# Time-Course Analysis of Brain Regional Expression Network Responses to Chronic Intermittent Ethanol and Withdrawal: Implications for Mechanisms Underlying Excessive Ethanol Consumption

**DOI:** 10.1371/journal.pone.0146257

**Published:** 2016-01-05

**Authors:** Maren L. Smith, Marcelo F. Lopez, Kellie J. Archer, Aaron R. Wolen, Howard C. Becker, Michael F. Miles

**Affiliations:** 1 Department of Pharmacology and Toxicology, Virginia Commonwealth University, Richmond, Virginia, United States of America; 2 Department of Human and Molecular Genetics, Virginia Commonwealth University, Richmond, Virginia, United States of America; 3 Department of Biostatistics, Virginia Commonwealth University, Richmond, Virginia, United States of America; 4 VCU Alcohol Research Center, Virginia Commonwealth University, Richmond, Virginia, United States of America; 5 Department of Psychiatry and Behavioral Sciences, Medical University of South Carolina, Charleston, South Carolina, United States of America; 6 Department of Neuroscience, Medical University of South Carolina, Charleston, South Carolina, United States of America; 7 RHJ Department of Veterans Affairs Medical Center, Charleston, South Carolina, United States of America; Coll. Medicine, UNITED STATES

## Abstract

Long lasting abusive consumption, dependence, and withdrawal are characteristic features of alcohol use disorders (AUD). Mechanistically, persistent changes in gene expression are hypothesized to contribute to brain adaptations leading to ethanol toxicity and AUD. We employed repeated chronic intermittent ethanol (CIE) exposure by vapor chamber as a mouse model to simulate the cycles of ethanol exposure and withdrawal commonly seen with AUD. This model has been shown to induce progressive ethanol consumption in rodents. Brain CIE-responsive expression networks were identified by microarray analysis across five regions of the mesolimbic dopamine system and extended amygdala with tissue harvested from 0-hours to 7-days following CIE. Weighted Gene Correlated Network Analysis (WGCNA) was used to identify gene networks over-represented for CIE-induced temporal expression changes across brain regions. Differential gene expression analysis showed that long-lasting gene regulation occurred 7-days after the final cycle of ethanol exposure only in prefrontal cortex (PFC) and hippocampus. Across all brain regions, however, ethanol-responsive expression changes occurred mainly within the first 8-hours after removal from ethanol. Bioinformatics analysis showed that neuroinflammatory responses were seen across multiple brain regions at early time-points, whereas co-expression modules related to neuroplasticity, chromatin remodeling, and neurodevelopment were seen at later time-points and in specific brain regions (PFC or HPC). In PFC a module containing *Bdnf* was identified as highly CIE responsive in a biphasic manner, with peak changes at 0 hours and 5 days following CIE, suggesting a possible role in mechanisms underlying long-term molecular and behavioral response to CIE. Bioinformatics analysis of this network and several other modules identified *Let-7* family microRNAs as potential regulators of gene expression changes induced by CIE. Our results suggest a complex temporal and regional pattern of widespread gene network responses involving neuroinflammatory and neuroplasticity related genes as contributing to physiological and behavioral responses to chronic ethanol.

## Introduction

Alcohol abuse and dependence have significant health and social consequences. Alcohol Use Disorder (AUD) is characterized by chronic excessive alcohol consumption, often alternating with periods of abstinence. Previous studies over the last two decades have suggested that neuroplasticity occurring in the brain’s reward and stress pathways contributes to the development of AUDs, and that changes in gene expression may be an important molecular mechanism underlying such neuroadaptations [1–4].

Genomic approaches involving microarrays or RNA-seq, together with scale-free network analyses, have recently shown that gene networks of highly correlated expression patterns are associated with acute or chronic ethanol exposure in brain tissue derived from animal models and human autopsies [5–7]. Such networks often have conserved biological functions or regulatory mechanisms [8, 9] providing novel mechanistic information about the neural actions of ethanol and other drugs of abuse [10]. Additionally, network topology analysis allows the identification of highly connected “hub genes” that have been shown to provide key regulatory functions over expression networks [6, 8]. Applying such approaches to animal models of alcohol dependence could thus provide new understanding of mechanisms underlying associated neuroplasticity, and identify new therapeutic targets for intervention in AUDs.

Although no animal model fully recapitulates the clinical characteristics of AUD, efforts to more accurately reflect development of AUD have recently shown considerable progress in providing predictive validation for new therapeutic targets [11, 12]. One such widely used model is the chronic intermittent ethanol vapor (CIE) paradigm where rodents are exposed intermittently to cycles of ethanol vapor such that they experience repeated cycles of exposure and withdrawal [13–15]. Cycles of heavy use and withdrawal are seen in alcoholics [16] and are thought to be an important component underlying the neuroplasticity that results in compulsive heavy abuse and frequent recidivism seen with AUD. The CIE model has been shown to produce lasting increases in ethanol consumption as well as neurochemical, physiological and synaptic structural changes [14, 17, 18]. However, the model obviously uses a much shorter time frame for exposure (weeks-months) than seen in AUD, and oftentimes requires inhibitors of alcohol metabolism so as to maintain higher blood alcohol levels [19]. Earlier genomic studies of CIE exposure in mice indicated brain regional and time-dependent changes in gene expression that may contribute to the behavioral and physiological plasticity evoked by chronic intermittent ethanol exposure [20]. However, a detailed network level analysis of gene expression adaptations with CIE has not been performed. Such an approach could identify key regulatory hubs that may play a significant role in mediating behavioral and physiological consequences of CIE treatment.

Here we use the Weighted Gene Correlated Network Analysis (WGCNA) scale-free network algorithm to analyze a detailed time-course study of CIE-evoked changes in gene expression across multiple brain regions comprising the mesolimbocortical dopamine and extended amygdala pathways. These neural pathways are thought to have a pivotal role in the development of excessive ethanol consumption associated with dependence [2, 3]. We found both conserved and region-specific rapid waves of expression network changes occur across multiple brain regions after ethanol withdrawal. However, following prolonged withdrawal (7 days), the hippocampus and the prefrontal cortex show persistent expression network alterations. The functional and network topology analysis of such networks provides key targets for future studies aimed at elucidating mechanisms of behavioral plasticity occurring with CIE. In particular, we implicate a *Bdnf*-containing network in prefrontal cortex as a potentially important contributor to the neurobiology of progressive ethanol consumption associated with dependence.

## Materials and Methods

### Ethics Statement

All animal studies were approved by the Institutional Animal Care and Use Committee at the Medical University of South Carolina (MUSC) and conducted in accordance with the guidelines outlined in the NIH Guide for the Care and Use of Laboratory Animals [21].

### Animals and Chronic Intermittent Ethanol Exposure

**Fig 1:**
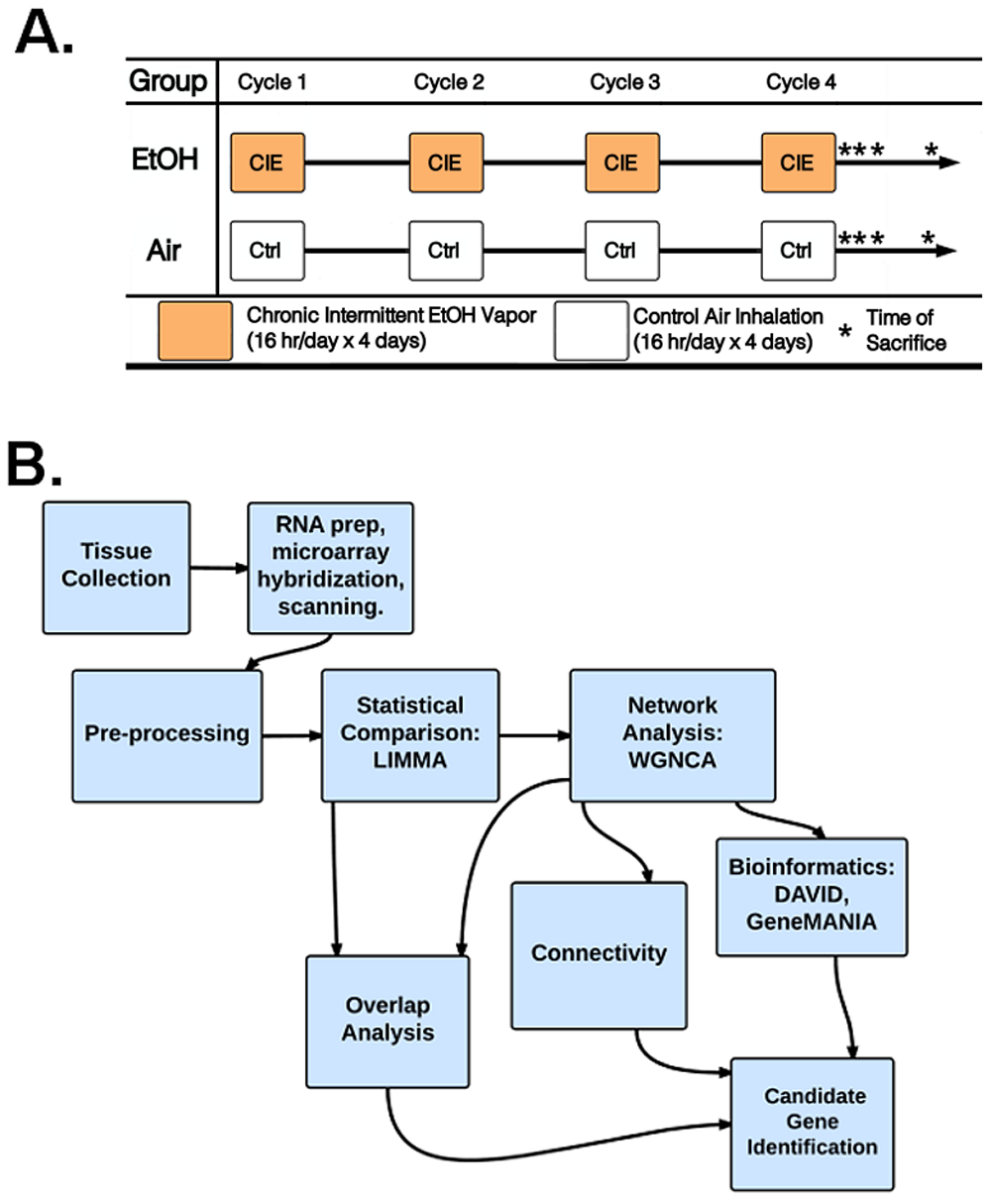
Schematic representation of experimental design and analysis.

Adult male C57BL/6J mice purchased from Jackson Laboratories (Bar Harbor, ME, USA) were individually housed in an AAALAC-accredited animal facility under a 12-hour light/dark cycle. Mice were given free access to food and water during all experimental procedures. After a 2-week acclimation period, mice (n = 48) were exposed to chronic intermittent ethanol (CIE) vapor or air in inhalation chambers, as previously described [14, 18, 19, 22]. Mice were divided into two groups of 24. One group (CIE) received ethanol vapor exposure for 16 hours/day for 4 days while the other group was similarly handled but received only air exposure in the inhalation chambers (Control; Ctrl). For CIE mice, ethanol was volatized by passing air through an air stone submerged in 95% ethanol. Chamber ethanol concentrations were monitored daily and air flow was adjusted to maintain ethanol concentrations within a range (10–13 mg/l air) that has been shown to yield stable blood ethanol concentrations (175–225 mg/dl) in C57BL/6J mice [14]. Before each chronic ethanol exposure cycle, intoxication was initiated in the CIE group by administration of ethanol (1.6 g/kg), and blood ethanol concentration was stabilized by injection of the alcohol dehydrogenase inhibitor pyrazole (1 mmol/kg). Both ethanol and pyrazole were administered intraperitoneally (i.p.) in a volume of 0.02 ml/g body weight. Ctrl mice were handled similarly, but administered saline and pyrazole (i.p.) prior to being placed in control chambers that delivered only air (no ethanol vapor). Thus, all mice received the same number and timing of pyrazole injections prior to final removal from the inhalation chambers. Following 4 days in the inhalation chamber, mice underwent 7 days of complete abstinence from ethanol. At the end of the abstinence period, mice were returned to the inhalation chamber to begin the next cycle of CIE. This pattern of 4 days CIE (or control air) exposure followed by 7 days abstinence was repeated for four complete cycles (Fig 1A). No animals had visible signs of ill health and there was no animal mortality during the experimental manipulations.

### Tissue Harvesting and RNA Isolation

Immediately following the last cycle of air or ethanol exposure as above, mice were removed from the inhalation chambers and euthanized at the appropriate time point by decapitation. Time points collected were 0, 8, and 72 hours (h) and 7 days (d), with n = 6 for each treatment/time group (Fig 1A). Following decapitation, mouse brains were immediately extracted from the skull, chilled on ice and dissected as described previously [20]. Tissue samples were frozen on dry ice and stored at -80°C until processed for RNA isolation. Total RNA was isolated using the RNeasy Mini Kit (Qiagen, Valencia, CA) exactly as described previously [20].

### Gene Expression Microarrays

The MUSC ProteoGenomics Core Facility processed RNA samples for microarray analysis using standard procedures as described by the manufacturer (Affymetrix, Santa Clara, CA). Samples were processed as a group by brain region with treatment groups and time points randomized to minimize batch effects. Gene expression was quantified with Affymetrix GeneChip^®^ Mouse Genome 430A 2.0 arrays. Scanning data was stored in CEL file format using Affymetrix Expression Console software, and these data files were transferred to Virginia Commonwealth University (VCU) for further analysis. Raw data files (CEL files) and RMA normalized expression values for all brain regions have been submitted to the Gene Expression Omnibus (GEO) database under accession number GSE5217.

### Microarray Analysis

Affymetrix GeneChip^®^ Mouse Genome 430 2.0 arrays were analyzed using The R Project for Statistical Computing (http://www.r-project.org/). RNA degradation, average background, and percent present probesets were used to assess array quality, and inspect for outlier arrays. Quality of each microarray was also assessed primarily by principal component analysis. Plots of first principal component by second principal component allowed for visual identification of outliers and batch effects between arrays. Background correction and normalization were performed using the affy package for R [23]. Due to batch effects noted in principal component plots, microarrays were separated by RNA hybridization batch for initial normalization. Each batch was background corrected with the Robust Multi-array Average (RMA) technique and normalized by quantile normalization [24]. The second step involved subjecting all microarrays together to another round of quantile normalization. Finally, ComBat with hybridization group as the batch effect was used to remove any remaining batch effects reflected in the data [25]. The only exception to this procedure was the prefrontal cortex where repeat group was used as only the batch effect correction factor.

### CIE Responsive Genes

CIE regulated genes were identified using the limma package for R [26]. Comparisons were made between CIE and Ctrl groups at each time point (0h, 8h, 72h, and 7d), and overall significance was determined by ANOVA. The Benjamini and Hochberg false discovery rate method (FDR) [27], was used to account for multiple testing. For the purposes of these studies, false discovery rates equal to or less than 0.01 were considered indicative of significant differences in gene expression between CIE and Ctrl mice.

### Weighted Gene Correlated Network Analysis

Weighted Gene Correlated Network Analysis (WGCNA) was used for scale-free network topology analysis of microarray expression data [8]. WGCNA was performed on each brain region independently with the WGCNA package for R [28]. Probesets were selected for WGCNA based on overall significance by ANOVA (FDRs equal to or less than 0.01). Any probeset found to be significant by ANOVA in any brain region was included, resulting in a total of 10,072 probesets used for WGCNA. Standard WGCNA parameters were used for analysis, with the exceptions of soft-thresholding power and deep split. A soft-thresholding power of 6 was used for all brain regions; this power was selected using methods described by Langfelder and Zhang [28]. WGCNA was performed with deep-split values of 0–3. Deep-split value was selected based on a multi-dimensional scaling (MDS) plot, which displayed first and second principal components. The criterion for deep-split value selection was that no modules showed overlap with each other by the MDS plot. Deep-split values of 3 were selected for all brain regions, except the nucleus accumbens, where a deep-split value of 2 was chosen. Modules were validated by bioinformatics analysis for over-represented biological functions (see below) and a statistical analysis based a permutation procedure outlined by Iancu et al. [29]. Briefly, the average topological overlap of probesets assigned to each module was compared to the average topological overlap of 100 bootstrapped modules comprised of randomly sampled probesets. Z-scores of average topological overlap between probesets assigned to the module, and modules comprised of random probesets were used to calculate p-values and false discovery rates (FDR). Modules with FDR values ≤ 0.2 were considered significant.

### Overlap Analysis

Overlap was determined between WGCNA modules and genes differentially expressed, as indicated by LIMMA FDR values equal to or less than 0.01 at each time-point. Fisher’s Test for Count Data [30] was then used to quantify the significance of overlap. WGCNA modules with Fisher’s Test for Count Data p-values ≤ 0.005 combined with odds ratios greater than 3 were determined to be significantly over-represented for differentially expressed genes at a certain time-point.

### Bioinformatics

Modules identified by WGCNA were examined for function using publicly available bioinformatics resources. The Functional Annotation Chart tool from DAVID (http://david.abcc.ncifcrf.gov/) [25] was used to identify biological pathways highly represented by genes grouped into each module. GeneMANIA (http://www.genemania.org/) was also utilized for functional analysis through use of constituent genes in each module as query lists for validation in GeneMANIA derived networks driven by previously published biological data sources (microarray, protein-protein interaction and others) [31]. The miRvestigator Framework application (http://mirvestigator.systemsbiology.net/) [32] was then used to identify microRNAs that may regulate modules that significantly overlap with differentially expressed genes at 0h and 7d in the PFC and HPC. The PFC and HPC were chosen for microRNA target analysis because these regions showed an appreciable level of regulation with CIE at 7d. A complete workflow of microarray analysis from tissue collection through bioinformatics is represented in Fig 1B.

### Candidate Gene Identification

The prefrontal cortex and hippocampus were chosen for detailed candidate gene characterization because these brain regions showed both immediate and long-term (7d) CIE induced changes in gene regulation (Table 1). Previous studies have shown a sustained increase in ethanol consumption at 7d post multiple CIE cycles [14], indicating that expression differences found at this time-point may contribute to the alteration in ethanol consumption. We reasoned that prolonged ethanol exposure-induced changes in gene expression (0h) might induce long-lasting structural or functional components of synaptic plasticity and contribute to elevated ethanol consumption, even if those genes mRNA expression patterns decayed to baseline over the 7d withdrawal period. Therefore, we restricted our detailed bioinformatics analysis and candidate gene identification to genes that were included in WGCNA, and showed significant differential gene expression by LIMMA at 0h or at 7d (FDRs equal to or less than 0.01). Genes fitting these criteria were then ranked by scaled within module connectivity (kIM) as described by Langfelder and Horvath [28].

**Table 1:** Linear Models for Microarray Analysis of Gene Expression.

Brain Region	CIE 0h vs. Air 0h	CIE 8h vs. Air 8h	CIE 72h vs. Air 72h	CIE 7d vs. Air 7d
PFC	3277	1527	238	427
NAC	717	28	0	0
HPC	865	967	3	604
BNST	1079	251	195	0
CEA	62	79	0	1

Number of significantly differentially expressed probesets at each time-point (LIMMA FDR ≤ 0.01).

## Results

### Time-Course Gene Expression with CIE

Gene expression analysis with LIMMA identified significant differential expression between CIE and Ctrl groups. The majority of gene regulation in all brain regions was observed during the first 8h after the final cycle of chronic intermittent ethanol. The prefrontal cortex (PFC) or hippocampus (HPC) showed the greatest number of CIE-regulated genes at any particular time point and they were the only regions to show expression changes at all time points. The contrasting response in PFC and HPC was particularly striking at the 7d time point where they each showed hundreds of post-CIE regulated genes while other brain regions were virtually quiescent (Table 1, S1–S5 Tables). Interestingly, the number of CIE-responsive genes at 7d in PFC and HPC were both greater than the numbers seen at 72h, suggesting a possible late withdrawal response or an unmasking of chronic CIE regulated genes following recovery from acute withdrawal. As an additional validation of the results from these current studies, we compared geneset membership of various time points and brain regions with those from a previously published initial analysis of expression responses to CIE [20]. In that analysis, PFC was also noted to have the most prominent responses at 0h following CIE, and HPC had significant withdrawal responses at 8h. Of these genesets, 48/284 genes overlapped with the PFC 0h responses in this current study (p = 0.00015) and 32/129 genes in HPC overlapped with the HPC 8h geneset (p = 6.01x10^-11^) in this current study (see S7 Table vs. S2 and S3 Tables in Melendez et al., 2011). This degree of cross-study validation is strong support for the importance of the network studies described below that were the major focus of this current work.

**Fig 2:**
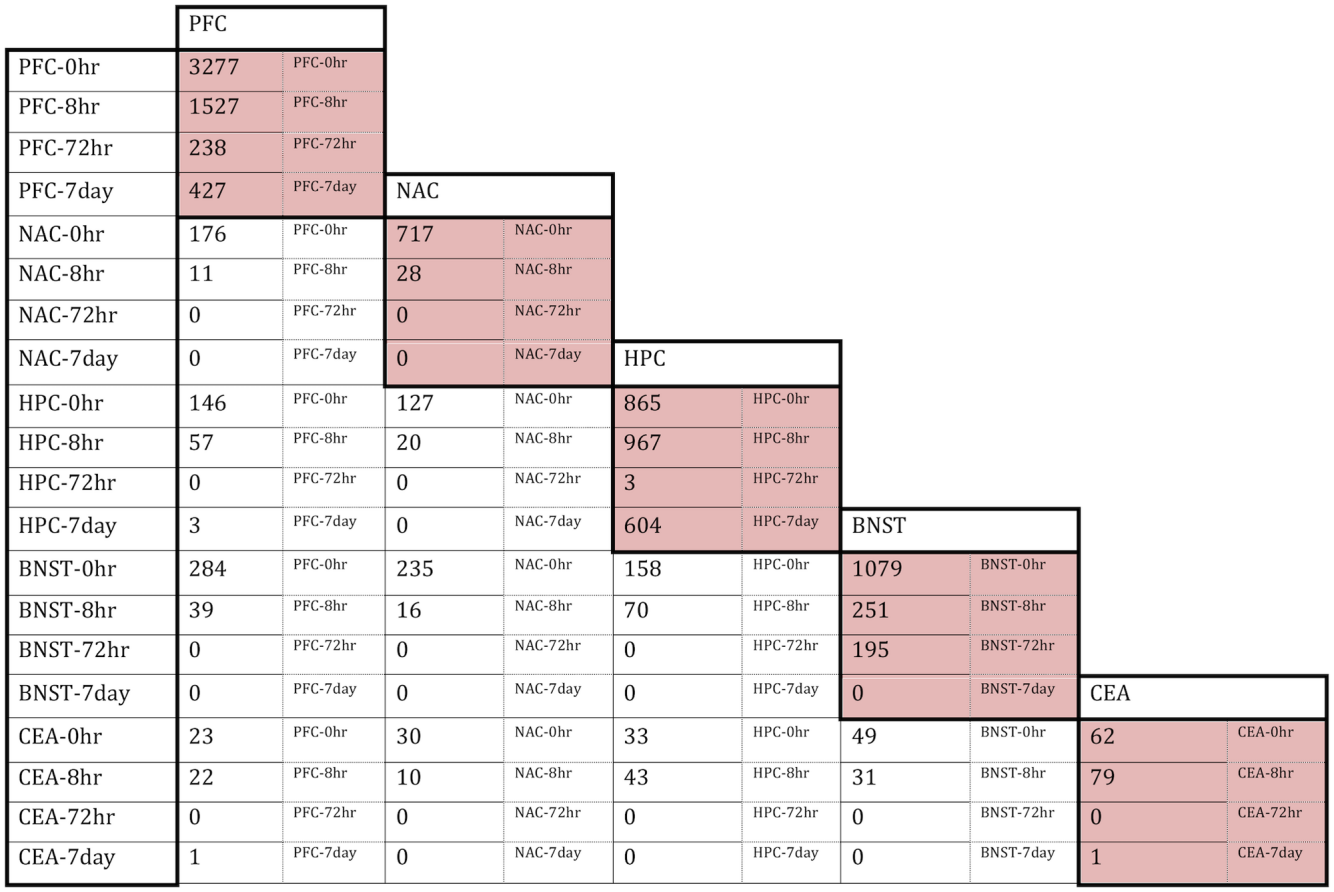
Overlap between CIE- regulated probesets at each time-point across brain regions. Table documents number of probesets significantly regulated by CIE (FDR ≤ 0.01) at each time point within individual brain regions (shaded cells) and overlap with same timepoints across other brain regions.

Comparison of overlaps in CIE-regulated gene sets either across time points within a brain region or across brain regions within a single time point revealed patterns of co-regulation. In all brain-regions, the greatest amount of overlap across time periods was seen between 0h and 8h, but these patterns largely decayed by 72h in most brain regions (S6 Table). Only PFC showed significant temporal overlaps across all time points. In both the PFC and HPC, there were over 100 probesets with overlapping regulation at both 0h and 7d, indicating that many genes responding to CIE showed persistent changes following a prolonged withdrawal period (Fig 2, S6 Table). Across brain regions, there was overlap in gene sets most prominently at the 0 and 8h time points (Fig 2). However, across brain-regions at 7d, only three genes overlapped between the PFC and HPC and one gene between PFC and CEA (Fig 2). This finding shows long-term gene regulation after CIE is specific to individual brain regions. There was also no overlap seen between the PFC and BNST at 72h, suggesting that gene expression changes during late withdrawal were also brain-region specific (Fig 2). Thus, four cycles of CIE induced robust changes in gene expression across multiple brain regions that largely decay over a 72h withdrawal period, except for PFC and HPC where region-specific persistent changes were seen.

### Weighted Gene Correlated Network Analysis

**Fig 3:**
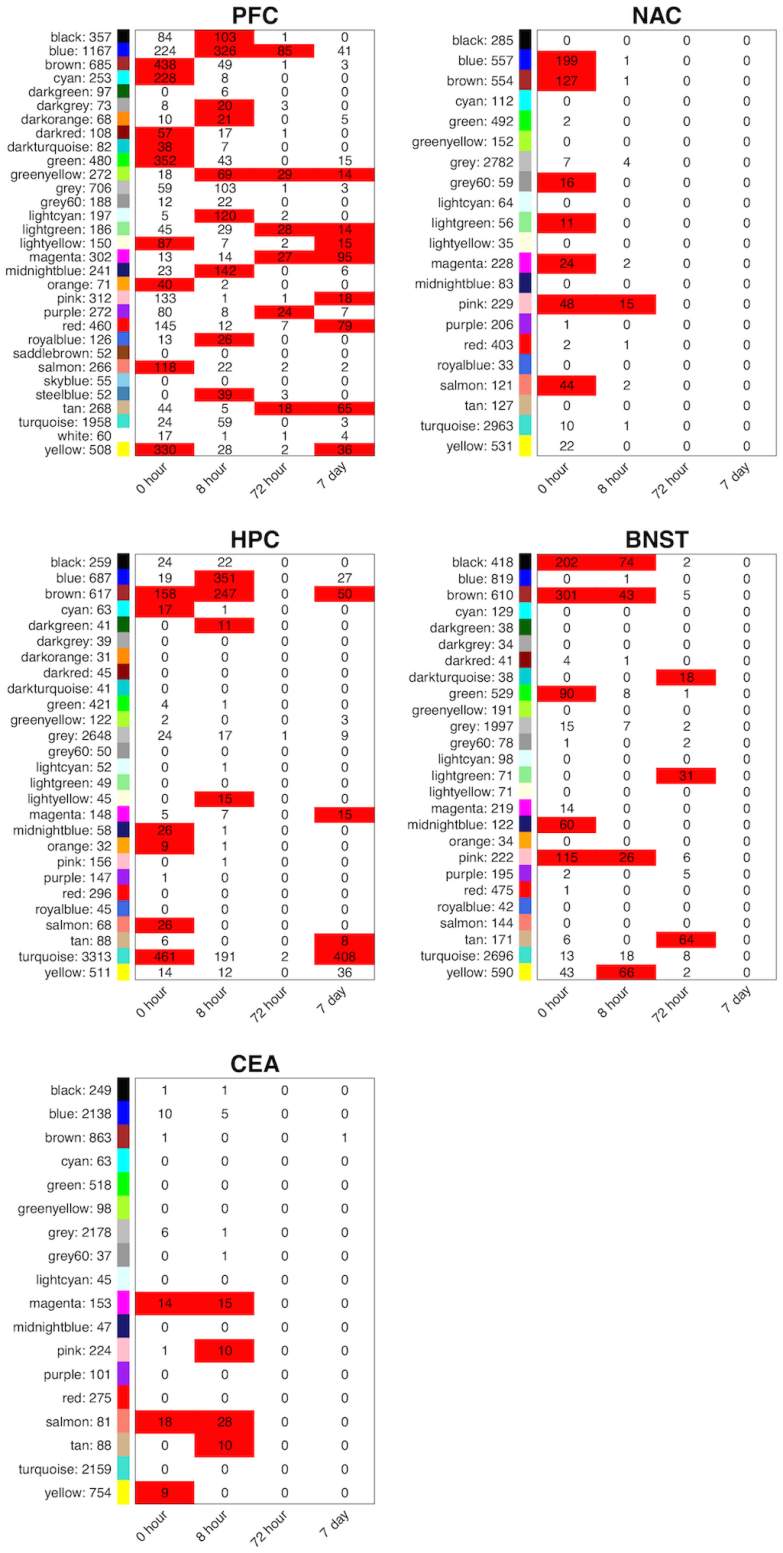
Overlap between CIE-regulated probesets and modules identified by WGCNA. Cell numbers indicate number of overlapping probe-sets, Cell color indicates significant overlap. Significant overlap: p-value ≤ 0.005 and Odds Ratio ≥ 3. Names and number of genes for each module are listed at far left columns within each brain region.

WGCNA identified expression modules in each of the 5 brain regions studied. Similar to differential gene expression analysis, the PFC (n = 31) and HPC (n = 27) had the largest number of modules and CEA (n = 18) the least (Fig 3 and S1–S5 Figs). Module sizes varied from over 3000 probesets to less than 35 (Fig 3, S7 and S13 Tables). The vast majority of these modules were validated by statistical comparison of topological overlap versus randomly permuted genesets of the same size as an individual module (see Methods; S13 Table) or by bioinformatics analysis for biological over-representation (see below). As expected, the “grey” module in each brain region showed no statistical significance since these modules only contain genes not gathered into other modules. However, HPC was an exception, where only 15/26 modules (excluding grey module) reached statistical significance at an FDR ≤0.2.

**Fig 4:**
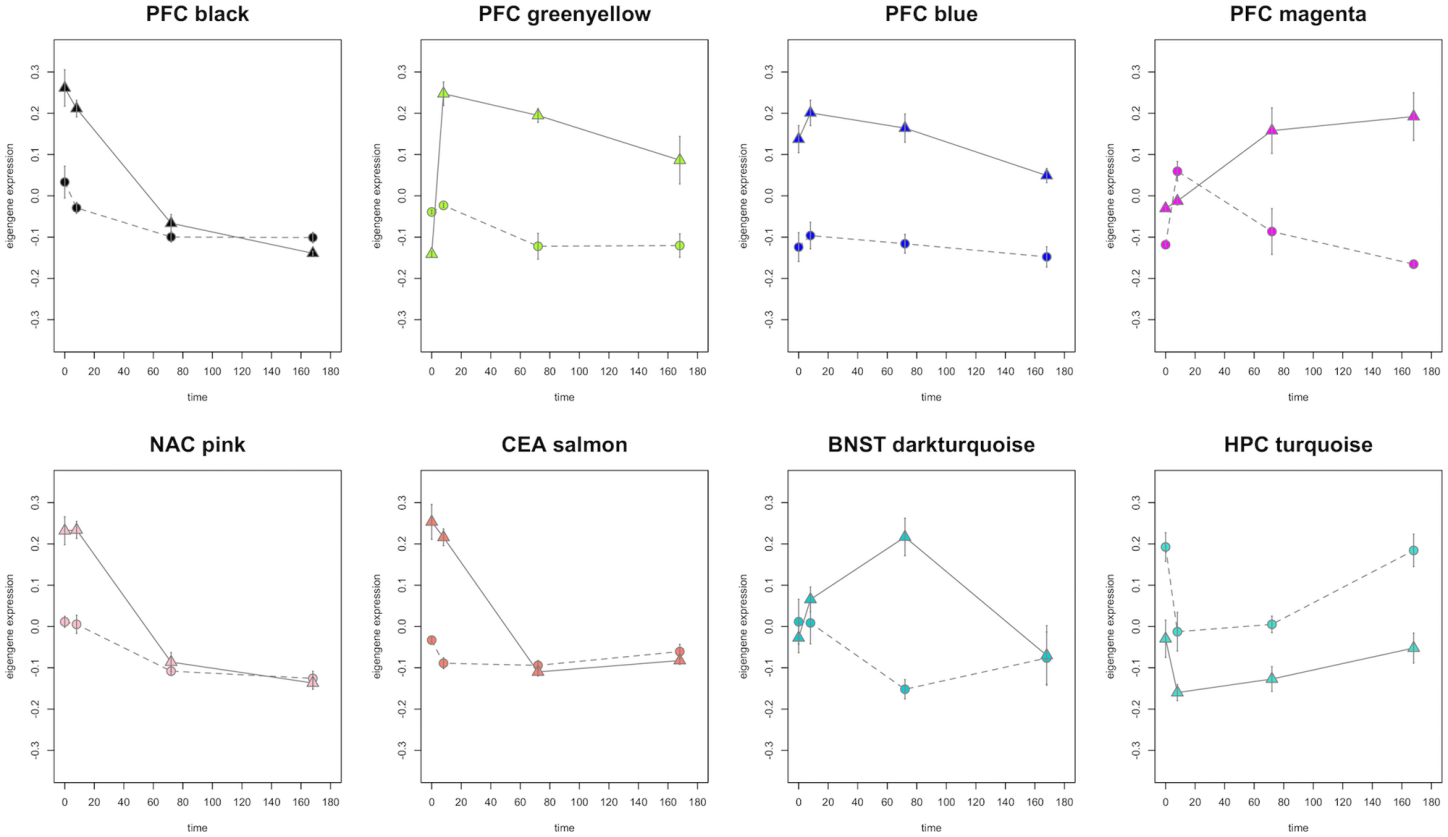
Representative kinetic profiles for module eigengenes. Module eigengene expression vs. time plots for the black, greenyellow, blue, and magenta modules in PFC, pink module in NAC, salmon module in CEA, darkturquoise module in BNST, and turquoise module in HPC. (detailed discussion of module functions in Results section). Triangle = CIE, Circle = Ctrl.

We next evaluated all modules for over-representation of genes regulated by CIE in the LIMMA analysis described above. Across all brain regions, 31 modules were significantly over-represented with genes regulated by CIE at 0h, 23 at 8h, 9 at 72h, and 13 at 7d (Fig 3, and S13 Table). Importantly, of the 13 modules in hippocampus that were over-represented with CIE-regulated genes at some time interval, only two (cyan and orange) had module topological overlap significance scores with FDR ≥ 0.2 (S13 Table). When genes within modules were summarized as “eigengenes” (first principal component of expression patterns for all genes in the module), a variety of temporal profiles were identified (Fig 4, S1–S4 Figs). The topology of kinetic profiles was most diverse in PFC and HPC while other brain regions mainly displayed module eigengene profiles that decayed to control levels by 8 or 72h post withdrawal (Fig 4). PFC and HPC were the exception with some modules displaying persistent or de novo expression changes at 7d in CIE-treated animals.

### Commonly Occurring Biological Processes

Modules that significantly overlapped with differentially expressed genes at the 0h and 7d time points were chosen to discuss further bioinformatics analyses because these time points represent the initial and sustained responses to chronic ethanol exposure (Fig 3, S7 Table). However, over-representation analysis of all modules for all brain regions is contained in S8–S12 Tables. At the 0h and 7d time points, a number of gene ontology categories showed significant over-representation (p ≤ 0.05) across modules in multiple brain regions. This suggests more global functional changes produced by CIE being elicited at those time points. Gene Ontology categories were considered “commonly occurring” if they showed significant overlap with 10% or more of all WGCNA modules across brain-regions (≥12 modules). 10 GO categories were represented in 12 or more modules, and all of these were represented in all brain-regions studied (Table 2, S14 Table). Functionally, these 10 fell into 4 general categories: RNA processing (GO:0006397~mRNA processing, GO:0008380~RNA splicing), DNA damage response (GO:0006511~ubiquitin-dependent protein catabolic process, GO:0010942~positive regulation of cell death, GO:0006974~cellular response to DNA damage stimulus, GO:0006457~protein folding), development and differentiation (GO:0045596~negative regulation of cell differentiation, GO:0048732~gland development, GO:0051301~cell division), and chromatin (GO:0000785~chromatin) (Table 2). Of note, ubiquitination and RNA-spicing were two gene ontology functional categories identified in our earlier global study of CIE-regulated gene expression [20]. The two categories related to RNA processing contained several DEAD box proteins (S8–S12 and S14 Tables). These proteins are known to function as RNA helicases [33]. Serine/arginine matrix proteins (*Srrm1*, *Srrm2*, *Srrm3*) were also represented in these categories. Functionally, serine/arginine matrix proteins are involved in mRNA splicing [34–36]. These three genes have also been found to be regulated by ethanol in multiple brain-regions in mice and human alcoholics or correlated with ethanol consumption in previous genomic studies [6, 7, 37]. Many genes were represented within in the categories related to DNA damage. *Usp1*, *Ube2d3*, and *Tecb1* are just a few examples of genes within these categories that have also been found to be regulated by ethanol in cultured cells, mice, and rats [5, 38–40]. These results may in part be indicative of the genotoxic effects seen with high-dose ethanol exposure [41].

**Table 2:** Commonly Occurring Gene Ontology Categories.

GO Number	Number Modules	Number Brain-regions
GO:0006397~mRNA processing	17	5
GO:0006457~protein folding	16	5
GO:0008380~RNA splicing	15	5
GO:0000785~chromatin	14	5
GO:0051301~cell division	14	5
GO:0006974~cellular response to DNA damage stimulus	13	5
GO:0010942~positive regulation of cell death	12	5
GO:0048732~gland development	12	5
GO:0006511~ubiquitin-dependent protein catabolic process	12	5
GO:0045596~negative regulation of cell differentiation	12	5

Gene Ontology categories seen in ≥ 10% of modules across all brain-regions.

#### Prefrontal Cortex

**Fig 5:**
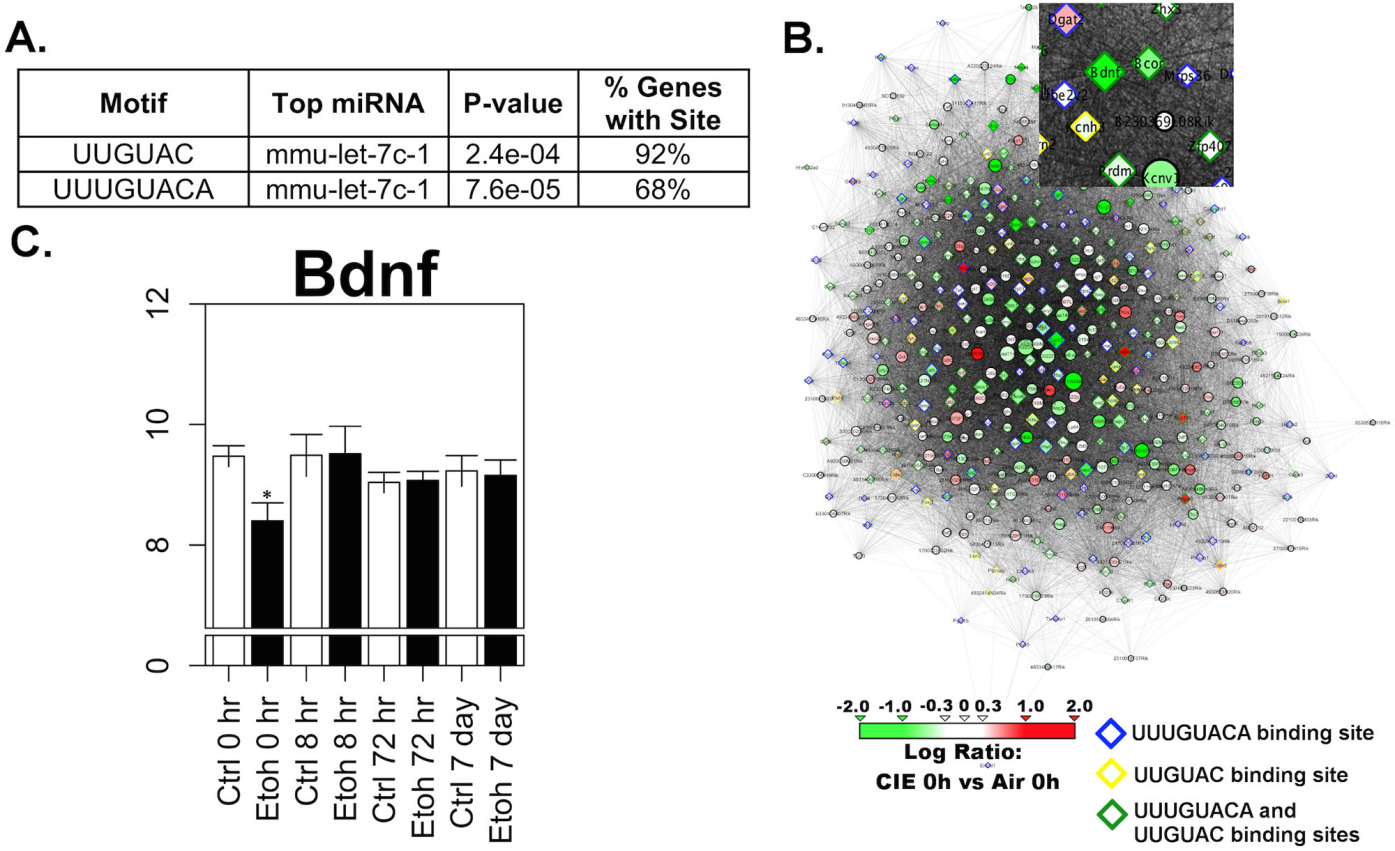
Bioinformatic analysis of PFC green module containing *Bdnf*. A) miRvestigator results of top miRNA motifs with complementary binding sequences in the PFC Green module. B) Network representation of the PFC Green module based on adjacency. Edge transparency indicates Pearson correlation coefficient. Node size reflects within-module connectivity determined by WGCNA. Node color indicates log-ratio of gene expression at 5 days CIE vs. Ctrl. Genes with mmu-let-7c-1 complementary sequences are highlighted. C) Average RMA value (log2 scaled, ±S.E.) expression of *Bdnf* at each time-point and treatment condition in the prefrontal cortex. (* = LIMMA FDR ≤ 0.05).

Overlap analysis between WGCNA modules and CIE-regulated gene sets revealed 9 modules enriched for genes regulated by chronic intermittent ethanol at 0h in PFC (Fig 3). Many of these modules contained genes involved in regulation of the cell cycle and apoptosis (S8 Table). The salmon and green modules showed several GO hits related to neuronal development, differentiation, and neuronal function. Genes within these GO categories included *Bdnf*, *c-fos*, *Bcor*, *Ppp2r3a*, *Hdac9* (green module), and *Notch1*, *Sox21*, *Sema3f*, *Gata2*, *Hdac2*, *Bmpr1a*, *Mkks* (salmon module) (S7 and S8 Tables). A highly significant number of genes in the green module contained potential base pairing motifs (68% with 8 base motif; 92% with 6 base-pairing match) for mmu-let-7c-1 (Fig 5a and 5b). Bdnf occupied a highly interconnected central position in the green module (Fig 5b), while showing significant expression changes only at the 0h time point (Fig 5c). The salmon module similarly had 68% of the genes with 8 base-pairing motifs for sequences within the miR-181 family and the let-7 family (S15 Table). These motifs were also contained in miR-543, miR-318, and miR-539-3p.

**Fig 6:**
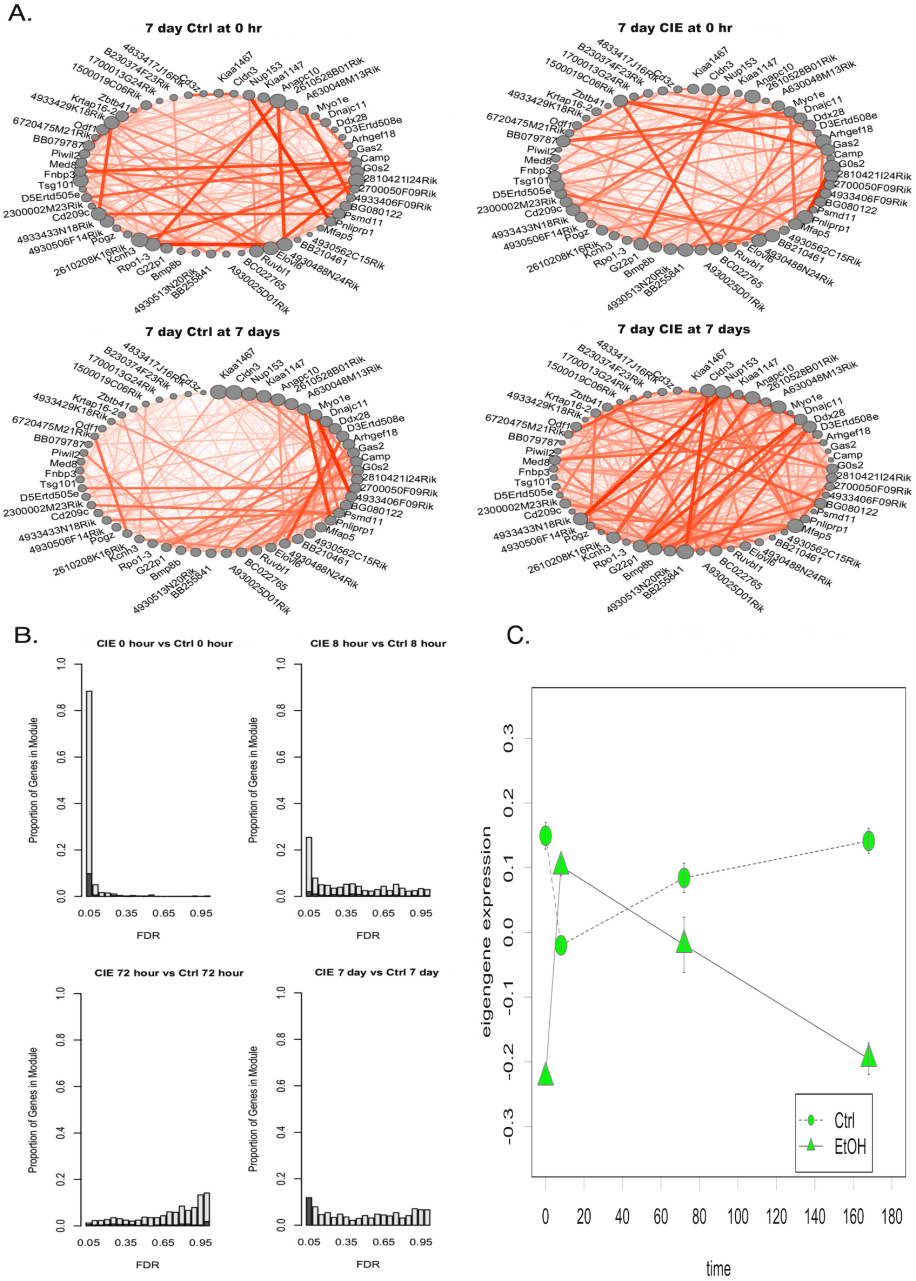
Network level analysis of PFC green module. A) Disruption of co-expression with CIE in genes regulated at 5 days (LIMMA FDR ≤ 0.05). Node size = within module connectivity. Ordered by within module connectivity at 5 days in Ctrl mice. B) Histograms for FDR of genes in PFC Green module at each time point. Dark grey = overlap of genes regulated at 5 days (LIMMA FDR ≤ 0.05). C) Eigengene expression time course for green module genes in control or ethanol (CIE) treated animals.

Given the potential role of Bdnf in mediating long-term plasticity underlying increased ethanol consumption after CIE [20, 42, 43], we performed further network analysis of the green module (Fig 6). Strikingly, while many genes in the green modules show significant changes in expression at the 0h time point (as seen with Bdnf—see Fig 5c), there was also a group of genes that showed changes at 0h and 7d (Fig 6b). Looking solely at genes within the green module that were significantly regulated at 7d, network connectivity analysis within control vs. CIE samples showed that this group had decreased a striking increase in connectivity in the CIE samples at 7d versus 0h, or compared to the control samples at 0h or 7d (Fig 6a). Module eigengene expression values for the green module genes significantly regulated at 7d reflected the bimodal pattern, with decreased expression in CIE samples versus control at 0h and 7d (Fig 6C).

Two additional modules, lightyellow and yellow, were enriched for genes that showed significant differential expression between CIE and Ctrl at both the 0h and 7d time-points, but not at 8h or 72h (Fig 3, S1 Fig). This functional overlap mirrors the overlap between the 0h and 7d time points seen with gene lists by LIMMA analysis (Fig 2, S6 Table). Contained in the lightyellow module were genes involved in cell cycle regulation, nerve cell development, and organization of cell projections (S8 Table). The yellow module also included several genes related to cell cycle regulation and vesicular trafficking. The latter group included *Syn2*, *Syn3*, *Syt7*, and *Syt11* (S6 Fig) [44–47]. These modules may thus include biological pathways relevant to both immediate and long-term neuroplasticity resulting from CIE exposure, but not the physiological effects of withdrawal, since there was no overlap with genes regulated at the 8h and 72h time points that cover the interval of peak withdrawal [48, 49]. The PFC yellow module also contained a high percentage of genes (70%) with 8 base-pairing motifs for mmu-let-7c-1-3p, another let-7 family microRNA (S15 Table).

A total of 13 modules from PFC were enriched for genes regulated at the peak withdrawal time-points of 8 and 72h post-CIE (Fig 3, S7 and S13 Tables). Only 3 of these modules were enriched for genes significantly regulated both at 8h and 72h. These findings indicate that gene expression functional patterns changed significantly as withdrawal progressed. Those modules enriched for genes regulated at both withdrawal time-points contained genes involved in regulating cell proliferation and cell death (S8 Table). The black module, one of the modules significantly enriched at 8h but not 72h, contained genes involved in stress hormone response and hypothalamic-pituitary-adrenal signaling such as *Sgk1*, *Sgk3*, and *Nfkbia*. These genes were also regulated by acute ethanol in our prior studies [5, 6]. Three modules, lightgreen, magenta, and tan, were over represented at both the 72h and 7d time-points. The tan and lightgreen modules showed significant overlap (p-value ≤ 0.05) with GO categories related to T-cell mediated immunity (S8 Table), including *Il2*, *Il4*, *Igh-6*, *IGH-VJ558* and *Cebpg*. Regulation of these genes by ethanol has been demonstrated in mice and humans previously [7, 37]. These modules may thus reflect biological processes having longer lasting regulation by withdrawal, or they may represent long-term functional adaptations to chronic ethanol exposure that are only apparent in the absence of ethanol. If the latter is the case, then such immunoregulatory-laden modules could have an important role in long-term behavioral consequences of CIE.

Finally, 8 modules in the PFC were significantly overrepresented for genes differentially regulated only at 7d after the final cycle of CIE (Fig 3, S7 Table). All these modules contained genes associated with neurodevelopment or neurotransmitter release (S8 Table). The greenyellow, lightyellow, pink, and red modules also had several gene ontology (GO) hits related to calcium binding, and cytoskeletal organization and control. Similarly, GO hits related to the cell cycle and cell proliferation were identified in the pink, red, tan, and yellow modules. Finally, biological processes related to immune response were identified in the greenyellow and lightgreen modules. The gene co-expression networks identified by WGCNA in PFC and regulated by CIE, therefore appear to represent both the lasting neuroplasticity and neuroinflammatory responses to chronic ethanol exposure.

#### Nucleus Accumbens

Significant differences in gene expression between CIE and Ctrl mice in NAc were only found immediately after the final cycle of CIE exposure (0h) and during acute withdrawal (8h) (Table 1). Seven WGCNA modules were enriched for genes expressed at the 0h time-point (Fig 3, S2 Fig). Several of these modules showed overlap with GO categories related to cellular stress response, metabolism, chromatin structure and regulation of gene expression (S9 Table). For example, the salmon module contained genes significantly differentially expressed at 0h in the NAC (Fig 3) and was over-represented for functions involved in chromatin structure (S9 Table). Several genes in this general functional group of the salmon module (*Bptf*, *Mysm1 and Ube2b*) all were previously shown to respond to acute ethanol in mice [6]. The brown module also showed significant expression changes at the 0h time point and had a striking enrichment for genes involved in RNA splicing and processing (S9 Table).

#### Hippocampus

Hippocampus showed the second greatest amount of differential gene expression between Ctrl and CIE mice. This brain region was also the only one, besides the PFC, to show significant differential gene expression at both 0h and 7d (Table 1). Furthermore, the HPC had the largest number of genes showing differential expression at 7d (604) with the vast majority of these residing within the turquoise module (408/604; see Fig 3). Overall, 27 modules were identified by WGCNA in the HPC, and 5 of these were significantly overrepresented for genes regulated by CIE at 7d (Fig 3, S7 and S13 Tables, S3 Fig). All 5 of these modules were statistically significant on topological overlap analysis (S13 Table). Furthermore, there was a highly significant overlap of genes regulated at 0h or 7d in HPC. The 0h and 7d time points showed 796 and 556 genes, respectively, significantly regulated by CIE at FDR≤ 0.01 (Fig 3). These gene sets showed an overlap of 104 genes (p≤ 2.2 x 10–16; Fisher’s Exact Test), with 89 of these residing in the turquoise module (Fig 3, S7 Table).

**Fig 7:**
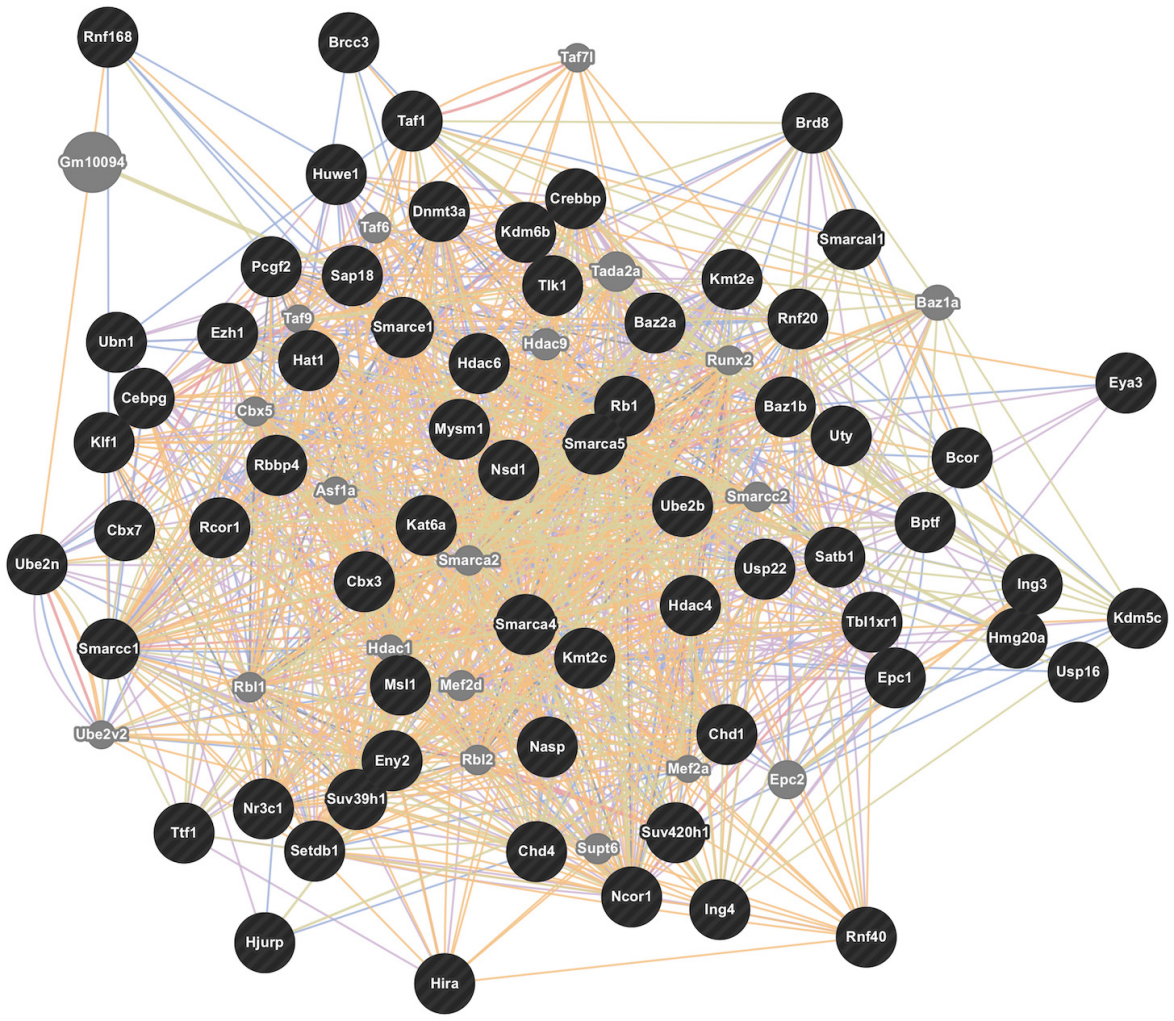
GeneMANIA analysis of genes from HPC turquoise module related to chromatin modification. Chromatin modification genes were identified from Gene Ontology analysis of the HPC turquoise module (S10 Table) and submitted to the GeneMANIA resource (www.genemania.org) for identification of network interactions using default criteria and databases.

The turquoise module in HPC was enriched for CIE-regulated genes at both the 0h and 7d time points and contained over 3000 genes, producing a complex bioinformatics analysis. Gene ontology analysis of the entire module showed strong over-representation for several functional groups potentially relevant to long term synaptic plasticity (S10 Table). These included extended groups of genes functioning in chromatin modification (Fig 7) such as histone acetylation (including *Baz2a*, *Brd8*, *Hdac4*, *Hdac6*, *and Myst3*), histone/DNA methylation (*Kdm6b*, *Kdm5c*, *Suv38H1*, *Suv420H1*, *and Dnmt3a*), chromatin remodeling (*Baz1b*, *Smarca4*, *Smarca5*, *SmarcaL1*, *SmarcC1*, *SmarcE1*), and histone/nuclear protein ubiquitination (*Ube2b*, *Ube2n*, *Ubn1*, *Usp16*, *and Usp22*). Similar results were found on over-representation analysis of only the genes showing CIE regulation (p≤ 0.05) at the 7d time point (S10 Table). Network connectivity analysis identified several highly connected hub genes in the HPC turquoise module, as discussed further in the Candidate Gene Identification section below.

Other HPC modules over-represented for genes regulated by CIE at 7d included the brown module, the only other module containing genes regulated at both 0h and 7d (Fig 3). This module contained genes related to immunity and cellular stress responses, including several genes encoding components of the major histocompatibility complex (S10 Table). Three HPC modules, magenta, tan, and yellow, were enriched for genes regulated by CIE at only the 7d time-point. The magenta module contained genes functioning in neurodevelopment, neuroplasticity, and synaptic transmission. These include *Vegfc*, *Notch1*, *Ppap2b*, *Scg2*, and several *Sox* family genes (S7 Fig) [50–54]. The yellow module also included genes known to be involved synaptic transmission such as glutamate receptors (*Gria1*, *Grik2*) and the D1 dopamine receptor gene (S7 and S10 Tables) [3, 55–57].

Of the HPC modules significantly overlapping with genes regulated by CIE only at 0h or 8h, most represented Gene Ontology hits seen in other brain regions such as immunity, cellular stress response, RNA splicing, transcription, and cell proliferation (S10 Table). Of note, our prior initial genomic analysis of CIE responses in hippocampus showed very prominent expression changes during acute withdrawal (8h) that included over-representation of genes involved in RNA splicing [20].

#### Bed Nucleus of the Stria Terminalis

The BNST was the only brain region other than PFC that showed significant gene regulation at 72h post-CIE (Fig 3, S4 Fig). Three modules were significantly overrepresented for genes regulated at only the 72h time-point. Two of these modules, tan and lightgreen, contained several genes related to neurodevelopment, and synaptic transmission (S7 and S8 Figs). These included *Ndrg1*, a myelin-related gene identified as an acute ethanol-responsive gene in our prior studies [5, 6, 58], (S7 and S11 Tables). The third 72h module, darkturquoise, contained genes related to the *Ras* GTPase intracellular signaling cascade. An additional 6 modules in the BNST (black, brown, green, midnightblue, pink, and yellow) were overrepresented for genes regulated by CIE at 0h, 8h, or both times (Fig 3, S4 Fig). Functionally, these modules contained genes overlapping with GO categories related to immune response, chromatin organization, transcription regulation, cell cycle control, and development (S11 Table).

#### Central Nucleus of the Amygdala

The CEA showed the least amount of differential expression between CIE and Ctrl mice at all time-points (Table 1) and, subsequently, fewer modules were identified by WGCNA than in the other brain regions (Fig 3, S7 Table). The CEA magenta and salmon modules were significantly enriched for genes expressed at both 0h and 8h post-CIE (Fig 3, S2 Fig). Bioinformatics analyses revealed that the magenta module contained genes related to immune response, particularly those encoding components of the major histocompatibility complex (S12 Table). Analysis of the salmon module identified several GO hits related to cell proliferation through negative regulation of programmed cell death. NF-κB was also identified as binding partner to multiple genes within the salmon module (S12 Table). Thus, NF-κB represents a possible target for network modulation in the CEA (S10 Fig).

The CEA yellow module was overrepresented for genes regulated at the 0h time point only. This module contained multiple genes related to neurodevelopment and synaptic transmission. Individually, only *Kif1b* was significantly regulated by CIE treatment in the CEA, but multiple other yellow module genes (including *Myo5a*, *Als2*, *Dlgap1*, *Egr3*, *Agtpbp1*, *Stx4a*, *Mecp2*, *Mylk2*, *Cacnb2*, *Lin7a*, *Psen1*, *Gria2*, *Trim9*, *Ssyn2*, *Chrna7*, *Ppp3ca*, *Bdnf*, *Grm5*, *Dlg4*, *Ncs1*, *Adra1a*, and *Lgi1*) were contained in 4 Gene Ontology categories related to synaptic transmission (S12 Table).

Two modules, pink and tan, were overrepresented for genes regulated by CIE only at the 8h time point, a time of peak withdrawal. The tan module was enriched with genes related to cellular stress response, many of which have been previously been associated with ethanol response in mice and humans (*Hsp5a*, *Cebpb*, *Dnajb9*, *Herpud1*, *Hes5*, *Creld2)* [5, 7, 37]. Analysis of the pink module also identified biological pathways representing cellular stress response, and included several genes previously identified as ethanol-responsive in brain, such as *Tsc22d3*, *Arrdc2*, *Htra1*, *Gclm*, and *Mt1* [7, 37] (S7 and S12 Tables).

### Candidate Gene Identification

To identify candidate genes for future study as major regulators of CIE-associated increased ethanol consumption, we focused attention on PFC and HPC where CIE-responsive genes (FDR≤0.05) were identified at 7d after removal from the vapor chambers. Furthermore, we identified hub genes having the highest scaled intramodular connectivity (kIM) (Tables 3 and 4, S17 Table), to focus on potential major regulators of network function [59].

**Table 3:** Prefrontal Cortex Candidate Genes.

ProbesetID	Gene Symbol	Within Module Connectivity	FDR CIE 7 days vs. Air 7 days
1439113_at	2410018L13Rik	1.000	0.003
1460202_at	Myoz1	1.000	0.003
1455946_x_at	Tmsb10	1.000	0.007
1422988_at	Sgsh	1.000	0.010
1436556_at	A930027H06Rik	1.000	0.011
1417711_at	0610012D09Rik	1.000	0.020
1418694_at	Kcmf1	1.000	0.033
1433996_at	Suv39h2	1.000	0.034
1425943_at	Nmur2	1.000	0.042
1432306_at	Rapgef5	0.985	0.013
1428006_at	Scfd1	0.979	0.044
1432615_at	Wdr37	0.976	0.001
1421837_at	Rps18	0.971	0.000
1430764_at	1700023F06Rik	0.931	0.014
1431466_at	4930553D19Rik	0.918	0.003
1446239_at	4921522A10Rik	0.908	0.001
1416893_at	Fam107b	0.898	0.033
1422166_at	Clec2i	0.895	0.005
1454088_at	5330411O13Rik	0.893	0.001
1443872_at	March2	0.892	0.034
1445578_at	Elovl6	0.887	0.005
1459149_at	Zfp809	0.881	0.013
1416154_at	Srp54	0.873	0.012
1459941_at	4933402J24Rik	0.871	0.017
1447850_x_at	Tex27	0.866	0.001
1432346_a_at	Cdh23	0.863	0.034
1423618_at	Bin1	0.857	0.005
1441289_at	C1orf54	0.842	0.000
1445973_at	C79461	0.839	0.001
1431332_a_at	Terf1	0.833	0.039

Top 30 most highly connected genes significantly differentially expressed 7 days (LIMMA FDR adjusted p-values ≤ 0.01) in the prefrontal cortex. Scaled module connectivity = within module connectivity/maximum number of connections possible as determined by WGCNA.

**Table 4:** Hippocampus Candidate Genes.

ProbesetID	Gene Symbol	Within Module Connectivity	FDR CIE 5 days vs. Air 7 days
1428941_at	Zmym2	1.000	0.027
1423065_at	Dnmt3a	1.000	0.007
1448940_at	Trim21	1.000	0.046
1426964_at	3110003A17Rik	1.000	0.034
1416897_at	Parp9	0.991	0.006
1429537_at	Srrp130	0.974	0.030
1420909_at	Vegfa	0.968	0.045
1451941_a_at	Fcgr2b	0.968	0.004
1447903_x_at	Ap1s2	0.967	0.027
1436343_at	Chd4	0.966	0.014
1439300_at	Chic1	0.962	0.020
1445499_at	Zc3h13	0.961	0.016
1460426_at	9430063L05Rik	0.957	0.018
1456316_a_at	Acbd3	0.956	0.008
1438069_a_at	Rbm5	0.955	0.018
1437638_at	Srrm2	0.950	0.004
1440375_at	5730419I09Rik	0.949	0.016
1456110_at	3010027A04Rik	0.947	0.018
1430599_at	Myt1l	0.943	0.006
1434055_at	Galnt9	0.942	0.038
1435477_s_at	Fcgr2b	0.942	0.004
1420402_at	Atp2b2	0.937	0.028
1438929_at	Actr1a	0.935	0.009
1423184_at	Itsn2	0.935	0.010
1451564_at	Parp14	0.935	0.041
1456262_at	Rbm5	0.934	0.023
1427401_at	Chrna5	0.933	0.005
1458147_at	Mamdc1	0.933	0.020
1438476_a_at	Chd4	0.926	0.005
1434020_at	Pdap1	0.924	0.010

Top 30 most highly connected genes significantly differentially expressed 7 days (LIMMA FDR adjusted p-values ≤ 0.01) in the hippocampus. Scaled module connectivity = within module connectivity/maximum number of connections possible as determined by WGCNA.

**Fig 8:**
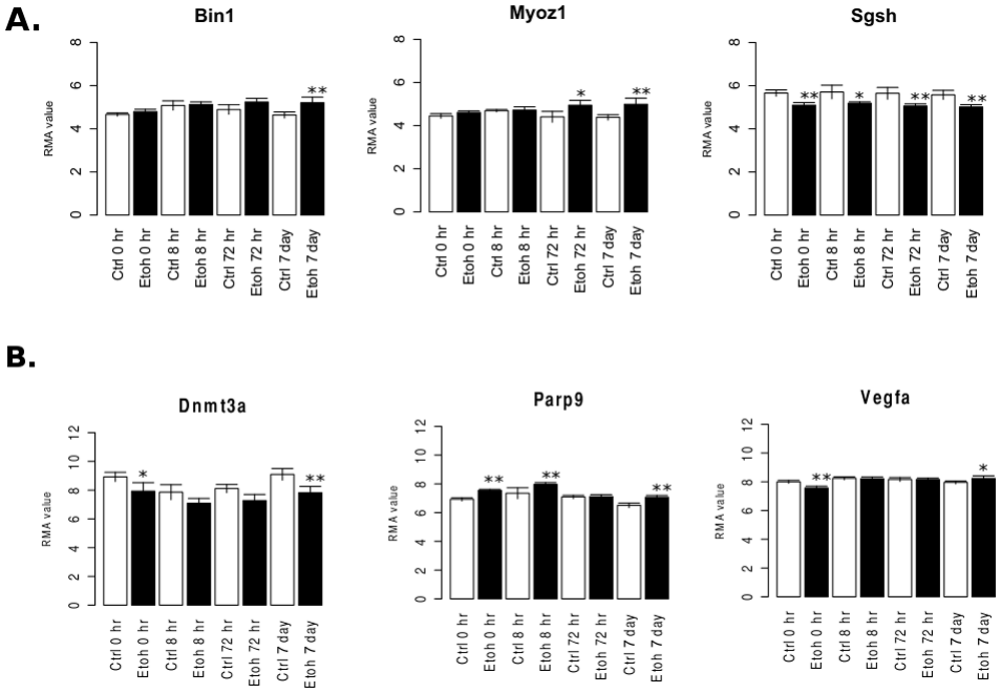
Expression patterns for representative candidate genes. A) Average RMA value (log2 scaled) expression of candidate genes at each time-point and treatment condition in the PFC cortex. (* = LIMMA FDR ≤ 0.05, ** = LIMMA FDR ≤ 0.01) B) Average RMA value (log2 scaled) expression of candidate genes at each time-point and treatment condition in the HPC. (* = LIMMA FDR ≤ 0.05, ** = LIMMA FDR ≤ 0.01).

Genes regulated by CIE in PFC at 7d and within the top 30 highest kIM scores, included *Myoz1* and *Sgsh* (S17 Table, Fig 8), with the former only becoming significantly different from Ctrl at the 72h and 7d time points. This strongly supports a possible role for *Myoz1* in longer term adaptations resulting from CIE. Previous studies from this laboratory have shown *Myoz1* expression correlates with individual variation in ethanol consumption in C57BL/6 mice [60]. *Myoz1* is most highly expressed in skeletal muscle but brain microarray databases suggest widespread lower expression in brain (www.genenetwork.org). The protein associates with the actin cytoskeleton and may play a role in determining cell shape [61]. *Sgsh* has also been correlated with ethanol behaviors in previous studies [37, 62] and found to have altered expression in alcoholic brain postmortem tissue [7]. *Sgsh* is involved in glycosaminoglycan degradation and mutations in the gene cause mucopolysaccharidosis IIIa. As two of the most highly connected genes within their respective modules, *Myoz1* and *Sgsh* may represent important regulatory proteins within a biological pathway induced by chronic ethanol exposure.

In the HPC, 1352 of the 10,072 probesets used for WGCNA were regulated by CIE (FDR≤ 0.05) at 7d. Interestingly, 60% (19/30) of the top 30 most highly connected genes in the HPC were within the turquoise module (S17 Table), even when within-module connectivity was scaled by the number of total genes in the module. The highly connected genes in the turquoise module represent a variety of biological functions from DNA processing to vesicle trafficking (Table 4, and S10 Table). Among the most highly connected genes in any HPC module were *Vegfa*, *Parp9*, and *Dnmt3a* (S17 Table, Table 4, Fig 8b). All these genes have previously been associated with ethanol responses in the literature [20, 62, 63]. Perhaps most strikingly regarding the highly interconnected turquoise module was the large subgroup of genes involved in chromatin modification (S10 Table). Fig 7 illustrates an external validation of this subnetwork, where the chromatin modification-related genes of the turquoise module were analyzed using the GeneMania bioinformatics tool (www.genemania.org) to illustrate connectivity between these genes using external data sources.

## Discussion

The investigation described in this manuscript employed a network-centric approach to identify brain region and time specific gene expression regulation by multiple cycles of chronic intermittent ethanol vapor exposure, an experimental model known to cause increased ethanol consumption [14, 15, 64]. Prior genomic studies have been conducted using similar vapor exposure models in mice and rats [20, 65], but this is first detailed network analysis to be performed on the time course of gene expression changes in this powerful behavioral model. Network analysis with WGCNA revealed modules of co-expressed genes regulated by CIE that showed remarkable time and brain-region specific expression patterns, with PFC and HPC showing the largest and most persistent expression changes. Functionally, chronic intermittent ethanol exposure and withdrawal caused time- and region-specific gene expression changes reflecting neuroplasticity, neuroimmunity, and neuroendocrine signaling responses to chronic ethanol. Additionally, our analysis suggests that possible mechanisms underlying persistent expression changes following chronic ethanol may involve regulation by miRNA and chromatin remodeling.

The prefrontal cortex and hippocampus were most affected by chronic ethanol, both in terms of number of differentially expressed genes at all 4 time-points, and as indicated by sustained gene expression changes at 7d post-CIE (Table 1; S1 and S17 Tables). Indeed, it was somewhat surprising that areas such as the BNST, CEA and particularly NAC did not show persistent changes induced by CIE. These regions did show strong responses at 0-8h after removal from the vapor chambers, particularly in regard to stress/inflammation-related functions (see below) and it is certainly possible that these mRNA expression changes evoked long-lasting translational or post-translational alterations that were relevant to long-lasting behaviors, but this will require additional study at the protein, structural or functional level to confirm such a hypothesis.

The findings presented in this study strongly implicate prefrontal cortex and hippocampus as brain regions most robustly influenced in terms of genomic regulation by CIE exposure, both at immediate time-points (0h) and after long-term abstinence (7d). The long-term changes in gene expression (Table 1, S1–S5 Tables) were of most interest because these possibly underlie behavioral responses to repeated chronic intermittent ethanol exposure, such as escalation of voluntary consumption observed in previous studies [14, 15]. Chronic heavy, and even moderate, ethanol intake has been shown to impair memory and hippocampal neurogenesis in humans and rodents [66, 67]. The hippocampus has also been implicated in withdrawal seizures, though there are mixed findings about the relationship between hippocampal atrophy with chronic heavy drinking, and onset and severity of withdrawal seizures [68–73]. Gene expression changes in the prefrontal cortex in response to both acute and chronic ethanol exposure have been demonstrated in mice and humans [5, 20, 65–67]. The prefrontal cortex's involvement in impulse control is hypothesized to underlie the ethanol seeking behaviors, increased consumption, and lack of control over intake associated with alcohol use disorders [74–76].

In contrast to the long lasting changes noted for PFC and HPC gene expression, chronic ethanol exposure and acute withdrawal, represented by tissue collected at 0-72h, affected all brain regions studied (Figs 2 and 3, Table 1). The greatest amount of overlap in differential gene expression, across all brain regions, also occurred at 0 and 8h (Fig 2). Functional over-representation studies showed, across all brain regions, an over-representation of genes involved in development, cell stress, programmed cell death, and immune responses at the 0h time point (S8–S12 Tables). The CIE magenta module, in particular, showed striking over-representation for genes related to MHC class 1 antigen responses with an over 2-fold up-regulation of H2-K1 and H2-L at 0h (S12 Table). The HPC brown module showed similar results (S10 Table). While many of these responses resolved as withdrawal proceeded to 72h and 7d, both HPC and PFC had persistent regulation of genes relating to immune responses at 7d. The strong presence of immune response genes across time points and brain regions in this study on CIE is consistent with observations from expression profiling of human autopsy brain material from alcoholics and subsequently validated in animal models [77]. Additionally, multiple recent studies have reported that intermittent ethanol exposure in adolescent animals can induce persistent changes in ethanol behaviors, including in adulthood, and that neuroinflammatory responses are a critical aspect of these responses to ethanol [78]. Together, these studies have suggested that ethanol-evoked activation of brain inflammatory responses may not just be a toxic response to ethanol, but could also play an important role in neuroadaptations leading to compulsive consumption. Neuroimmune responses have previously been implicated in other forms of experience-induced or developmental plasticity [79].

A priori, it was assumed that CIE would regulate networks of genes related to synaptic function, plasticity or development as part of the molecular events leading to progressive ethanol consumption following CIE. Indeed, gene modules over-represented with such functional groups were detected and showed regulation by CIE particularly at early time points (S8–S12 Tables). The PFC salmon module was significantly enriched for immediate early genes at the 0h time-point and several gene ontology hits related to neurodevelopment (S8 Table). *Notch1*, *Sox2*, and *Bmpr1a* are among the genes in the PFC salmon module with known roles in neurodevelopment. In particular, these genes have been shown to be important for the process of adult neurogenesis [50, 80–82]. Neurogenesis continues to occur into adulthood in the lateral ventricles and the dentate gyrus of the hippocampus [83, 84]. Studies examining adult neurogenesis occurring in other areas of the brain, including the medial prefrontal cortex (mPFC), have had mixed results [85–91]; but it has been shown that chronic stress and chronic alcohol exposure lead to observable structural and functional changes in the mPFC [92–98]. The PFC salmon module in this data set, therefore, may represent the effect of CIE on neurogenesis in the PFC of adult mice.

The PFC green module also contained genes related to neuroplasticity, notably *Bdnf*. *Bdnf* has previously been studied as a potential candidate gene for the genesis of alcohol use disorders. Previous studies have shown that *Bdnf* regulates neurodevelopment [99], synaptic plasticity [100], and is regulated by several drugs of addiction including ethanol [5, 101–105]. In looking more closely at the time-course of *Bdnf* expression in the PFC after CIE, *Bdnf* was significantly down regulated with CIE at 0h, in agreement with several prior studies on either CIE or intermittent oral ethanol consumption [20, 42, 43]. However, between 8h and 72h, *Bdnf* mRNA levels returned to control levels such that at 7 days, *Bdnf* gene expression was not significantly different between CIE and Ctrl mice (Fig 5c, S1 Table). This does not exclude the possibility that changes in BDNF protein might persist for more prolonged withdrawal periods [106]. Additionally, we found that a subgroup of genes in the PFC green module (not containing *Bdnf*) did show altered expression at both 0h and 7d post-CIE (Fig 6b). This subgroup of PFC green module genes also showed network level increases in connectivity at 7d post-CIE (Fig 6a). This may be further evidence for the role of a *Bdnf*-related gene network in the long-term neuroadaptive events leading to increased ethanol consumption following CIE exposure.

Studies by two separate laboratories using the vapor chamber CIE model in rats [43] or the intermittent ethanol consumption model in B6 mice [42], recently showed that chronic intermittent ethanol down-regulates mPFC *Bdnf* expression via increasing expression of select microRNA species, with resultant increases in ethanol consumption. Using a 7-week ethanol vapor exposure model, Tapocek et al. showed that reduced *Bdnf* expression in mPFC was accompanied by region-selective persistent increases in expression of *miR-206* and that viral vector over-expression of *miR-206* could, in itself, decrease mPFC *Bdnf*, with subsequent increases in ethanol consumption. Darcq et al. showed similar results in a mouse chronic intermittent binge ethanol model, including transient upregulation of *miR-1*. The *miR-1* miRNA family includes *miR-206*. However, Darcq et al. also found involvement of *miR-30a-5p*, including that inhibition of miR-30a-5p action could reverse the increased consumption caused by intermittent ethanol access [42]. In our own analysis of miRNA binding site over-representation among genes of the PFC green module, binding sites for both *miR-30a* (p = 0.003) and *mmu*-*miR-1a/mmu-miR-206* (p = 0.04) showed nominally significant potential binding motifs among genes in the PFC green module using MiRvestigator Framework (S15 Table), suggesting that these miRNA families may be involved in regulation of green module genes beyond *Bdnf* alone. Future direct studies will be needed to confirm such *in silico* findings.

Additionally, our studies suggested that the PFC green module was over-represented with binding sites for the *let-7c-1* group of miRNA, with 6 base motifs for *let-7c-1* being found in over 90% of the green module genes (p< 0.00024; Fig 5). MiRvestigator Framework web-software also revealed that 12 differentially regulated modules in the PFC and HPC were enriched for potential *let-7* family target genes (Fig 5; S15 and S16 Tables). *Let-7* was one of the earliest microRNA's discovered, and is highly conserved in function across species [107]. In the brain, in addition to being a key regulator of cell differentiation in early development, previous studies have shown that *let-7* expression is regulated by several types of neurodegenerative processes, from prion disease to ischemic brain injury [108–111]. We thus suggest that CIE exposure may increase long-term consumption through a miRNA-dependent regulation of the green module genes, including a role for the *let-7* miRNA family, which could impact CIE regulation of other modules as well. This hypothesis complements the prior direct work on *Bdnf* and suggests that mechanisms underlying regulation of the green module by chronic ethanol could be a novel target for future therapeutic approaches in treatment of alcohol use disorders. Confirmation that *let-7c-1* regulates genes of the PFC green module *in vitro* and *in vivo* is required before such a hypothesis could be tested and is the subject of ongoing studies.

Long-term gene expression and behavioral changes resulting from CIE exposure require a mechanism for persistence in the absence of further ethanol vapor exposure. Epigenetic mechanisms have lately been implicated as a causal factor for long-term functional and behavioral changes evoked by ethanol and other drugs of abuse [112, 113]. It is certainly possible that synaptic reorganization caused, for example, by miRNA-driven alterations in *Bdnf* expression, could subsequently produce persistent changes in synaptic function and behaviors. Our time course analysis of expression changes following CIE provided strong preliminary evidence for additional epigenetic mechanisms possibly influencing persistent changes in ethanol consumption following CIE exposure. The striking over-representation for chromatin modification (Fig 7) in hippocampal turquoise module genes regulated by ethanol, suggests a mechanism for long-lasting shifts in transcriptional adaptations to CIE in hippocampus. Our candidate gene analysis for hub genes further emphasized the potential importance of these chromatin modification genes in CIE-associated expression network structure (Fig 8, Table 4, S17 Table). Ongoing studies in our laboratories seek to identify such epigenetic signatures amongst hippocampal networks showing long-lasting expression changes following CIE.

The discussion above regarding our findings must be taken with several possible experimental confounds in mind. First, the studies here did not employ ethanol consumption as part of the design. While the CIE protocol of repeated ethanol vapor chamber exposure has been shown to increase subsequent ethanol consumption, we cannot definitively say that any particular expression network is related to ethanol consumption since this wasn’t tested. Secondly, there are several factors that could have influenced brain gene expression on their own or by interacting with ethanol. These include possible general metabolic factors triggered by the repeated ethanol vapor exposures. While there was no lethality or obvious toxicity from the treatment protocol for the animals in this experiment, we did not directly test factors such as possible hypothermia, circadian rhythm disturbances, stress hormone factors or metabolic abnormalities (e.g. liver dysfunction). The use of pyrazole to maintain stable blood alcohol levels has been widely reported with the CIE model in mice and control animals also had identical pyrazole exposure. However, we cannot eliminate the possibility that some interaction between pyrazole and ethanol could have contributed to the brain gene expression patterns we report. Finally, the expression networks identified here might influence or be related to behavioral aspects of chronic ethanol other than progressive consumption. Although prior reports [14] have suggested that increased ethanol consumption after CIE is not due to an ethanol-deprivation effect [114], neuroadaptations related to ethanol withdrawal, rather than chronic ethanol per se, might contribute both the expression networks observed here and the increased ethanol consumption seen following CIE. Overall, however, the characteristic time course of expression changes seen in this work strongly suggests that many of the gene networks highlighted here are directly related to chronic ethanol exposure and withdrawal.

In conclusion, differential gene expression and scale-free network analysis has revealed region-specific correlated changes in gene expression with chronic intermittent ethanol exposure in the mesolimbocortical dopamine and extended amygdala pathways. Our bioinformatics investigation has shown some conservation of functional groups, both across brain regions and time points, among the differentially regulated networks. In general, neuroinflammatory responses were seen across multiple brain regions at early time points, while genes involved in development, neuroplasticity, and chromatin remodeling where found to be over-represented at 3-7d post ethanol vapor. Remarkably, PFC and HPC were the only regions of the five surveyed that showed expression changes at 7d after removal from the vapor chamber model of chronic ethanol exposure. Since animals offered oral ethanol intake at that time will show increased consumption, these PFC and HPC networks may have a significant mechanistic role in the neuroplasticity underlying progressive ethanol consumption. The Bdnf-containing green network from PFC is a major target for future confirmatory studies since other investigators have previously implicated a miRNA-directed regulation of *Bdnf* consequent to chronic ethanol exposure in the mechanisms of progressive ethanol consumption. Importantly, however, our studies suggest that members of the green network other than *Bdnf* may also be involved in the long-lasting molecular mechanisms underlying increased ethanol consumption. Finally, our discovery of a striking subgroup of genes involved in chromatin modification having altered expression in HPC at 7d post ethanol vapor suggest future studies on chromatin structure as an important regulatory event contributing to long-term abusive ethanol consumption patterns as seen in alcoholism. Taken together, these findings provide novel and significant insight to the molecular neurobiology contributing to abusive alcohol consumption, and could thus eventually lead to development of new therapeutic strategies for AUD.

## Supporting Information

S1 Fig Multi-dimensional scale plots of the first and second principal component of each module identified by WGCNA in the prefrontal cortex (PFC); hierarchical cluster dendrogram using the module eigengenes (first principal component) of each PFC module; and line graphs of average module eigengene expression in Ctrl and CIE samples at each time-point. (PDF)

S2 Fig Multi-dimensional scale plots of the first and second principal component of each module identified by WGCNA in the nucleus accumbens (NAC); hierarchical cluster dendrogram using the module eigengenes (first principal component) of each NAC module; and line graphs of average module eigengene expression in Ctrl and CIE samples at each time-point. (PDF)

S3 Fig Multi-dimensional scale plots of the first and second principal component of each module identified by WGCNA in the hippocampus (HPC); hierarchical cluster dendrogram using the module eigengenes (first principal component) of each HPC module; and line graphs of average module eigengene expression in Ctrl and CIE samples at each time-point. (PDF)

S4 Fig Multi-dimensional scale plots of the first and second principal component of each module identified by WGCNA in the bed nucleus of the stria terminalis (BNST); hierarchical cluster dendrogram using the module eigengenes (first principal component) of each BNST module; and line graphs of average module eigengene expression in Ctrl and CIE samples at each time-point. (PDF)

S5 Fig Multi-dimensional scale plots of the first and second principal component of each module identified by WGCNA in the central nucleus of the amygdala (CEA); hierarchical cluster dendrogram using the module eigengenes (first principal component) of each CEA module; and line graphs of average module eigengene expression in Ctrl and CIE samples at each time-point. (PDF)

S6 Fig Network representation of the PFC Yellow module based on adjacency. Edge transparency = Pearson correlation coefficient. Node size = within module determined by WGCNA. Node color = Log-ratio of gene expression at 5 days CIE vs. Ctrl. Genes involved in neurotransmitter release at the synapse highlighted. Network representation built using the Cytoscape resource (http://www.cytoscape.org).
(PDF)

S7 Fig Network representation of HPC Magenta module based on adjacency. Edge transparency = Pearson correlation coefficient. Node size = within module determined by WGCNA. Node color = Log-ratio of gene expression at 5 days CIE vs. Ctrl. Highlighted genes indicate genes involved in neurodevelopment. Network representation built using the Cytoscape resource (http://www.cytoscape.org).
(PDF)

S8 Fig Network representation of BNST Lightgreen module based on adjacency. Edge transparency = Pearson correlation coefficient. Node size = within module determined by WGCNA. Node color = Log-ratio of gene expression at 72 hours CIE vs. Ctrl. Network representation built using the Cytoscape resource (http://www.cytoscape.org).
(PDF)

S9 Fig Network representation of BNST Tan module based on adjacency. Edge transparency = Pearson correlation coefficient. Node size = within module determined by WGCNA. Node color = Log-ratio of gene expression at 72 hours CIE vs. Ctrl. Network representation built using the Cytoscape resource (http://www.cytoscape.org).
(PDF)

S10 Fig Network representation of CEA Salmon module based on adjacency. Edge transparency = Pearson correlation coefficient. Node size = within module determined by WGCNA. Node color = Log-ratio of gene expression at 0 hours CIE vs. Ctrl. Highlighted genes indicate genes shown to interact with NF-κB. Network representation built using the Cytoscape resource (http://www.cytoscape.org).
(PDF)

S1 Table Detailed results of linear models for microarray analysis (LIMMA). Results include log-ratios (called coefficients), t-statistics, p-values, FDR adjusted p-values, F-statistics from ANOVA, F-statistic p-values, F-statistic FDR adjusted p-values, and RMA values. Data for PFC.
(XLSX)

S2 Table Detailed results of linear models for microarray analysis (LIMMA). Results include log-ratios (called coefficients), t-statistics, p-values, FDR adjusted p-values, F-statistics from ANOVA, F-statistic p-values, F-statistic FDR adjusted p-values, and RMA values. Data for NAC.
(XLSX)

S3 Table Detailed results of linear models for microarray analysis (LIMMA). Results include log-ratios (called coefficients), t-statistics, p-values, FDR adjusted p-values, F-statistics from ANOVA, F-statistic p-values, F-statistic FDR adjusted p-values, and RMA values. Data for HPC.
(XLSX)

S4 Table Detailed results of linear models for microarray analysis (LIMMA). Results include log-ratios (called coefficients), t-statistics, p-values, FDR adjusted p-values, F-statistics from ANOVA, F-statistic p-values, F-statistic FDR adjusted p-values, and RMA values. Data for BNST.
(XLSX)

S5 Table Detailed results of linear models for microarray analysis (LIMMA). Results include log-ratios (called coefficients), t-statistics, p-values, FDR adjusted p-values, F-statistics from ANOVA, F-statistic p-values, F-statistic FDR adjusted p-values, and RMA values. Data for CEA.
(XLSX)

S6 Table Time point comparisons of CIE-regulated genes within brain regions. Columns display the number of genes overlapping for the indicated timepoint comparisons within brain regions.
(DOCX)

S7 Table Connectivity measures, RMA-values, LIMMA log-ratios, WGCNA module assignments, and LIMMA FDR adjusted p-values of 10,072 probesets used for WGCNA. (XLSX)

S8 Table DAVID bioinformatics results obtained from each WGCNA module identified in the PFC. (XLSX)

S9 Table DAVID bioinformatics results obtained from each WGCNA module identified in the NAC. (XLSX)

S10 Table DAVID bioinformatics results obtained from each WGCNA module identified in the HPC. (XLSX)

S11 Table DAVID bioinformatics results obtained from each WGCNA module identified in the BNST. (XLSX)

S12 Table DAVID bioinformatics results obtained from each WGCNA module identified in the CEA. (XLSX)

S13 Table Results of overlap analysis between WGCNA modules and LIMMA significant results including number of overlapping genes, p-values, odds ratios, and representation factor. Also contains statistical verification of all modules by topological overlap analysis versus permuted random modules.
(XLSX)

S14 Table Count of number of WGCNA modules in each brain-region significantly overlapping with Gene Ontology categories (DAVID p-value ≤ 0.05 and number of overlapping genes between 3 and 300). (XLSX)

S15 Table miRvestigator results for PFC modules significantly overlapping with genes significantly differentially expressed at 0 hours or 7 days (significantly differentially expressed = LIMMA FDR ≤ 0.01, significantly overlapping = Fisher’s Exact test, p-value ≤ 0.005 and odds ratio ≥ 3). (XLSX)

S16 Table miRvestigator results for HPC modules significantly overlapping with genes significantly differentially expressed at 0 hours or 7 days (significantly differentially expressed = LIMMA FDR ≤ 0.01, significantly overlapping = Fisher’s Exact test, p-value ≤ 0.005 and odds ratio ≥ 3). (XLSX)

S17 Table Connectivity measures, RMA-values, LIMMA log-ratios, WGCNA module assignments, and LIMMA FDR adjusted p-values of probesets significant at 7 days (FDR ≤ 0.01) in the PFC (tab 1) and HPC (tab 2). (XLSX)
