# Correction: Time-Course Analysis of Brain Regional Expression Network Responses to Chronic Intermittent Ethanol and Withdrawal: Implications for Mechanisms Underlying Excessive Ethanol Consumption

**DOI:** 10.1371/journal.pone.0218700

**Published:** 2019-06-18

**Authors:** Maren L. Smith, Marcelo F. Lopez, Kellie J. Archer, Aaron R. Wolen, Howard C. Becker, Michael F. Miles

The Data Availability statement for this paper contains an incorrect accession number. The correct statement is: All relevant data are within the paper and its Supporting Information files. Additional data from microarray studies are available from the Gene Expression Omnibus (GEO) database, under accession number GSE72517.
