# Supplementary material for: Time-Course Analysis of Brain Regional Expression Network Responses to Chronic Intermittent Ethanol and Withdrawal: Implications for Mechanisms Underlying Excessive Ethanol Consumption
Source: PLoS One. 2016 Jan 5;11(1):e0146257. doi: 10.1371/journal.pone.0146257 (PMC4701666; doi:10.1371/journal.pone.0146257)
Supplement: S1 Fig — (PDF) [file pone.0146257.s001.pdf]

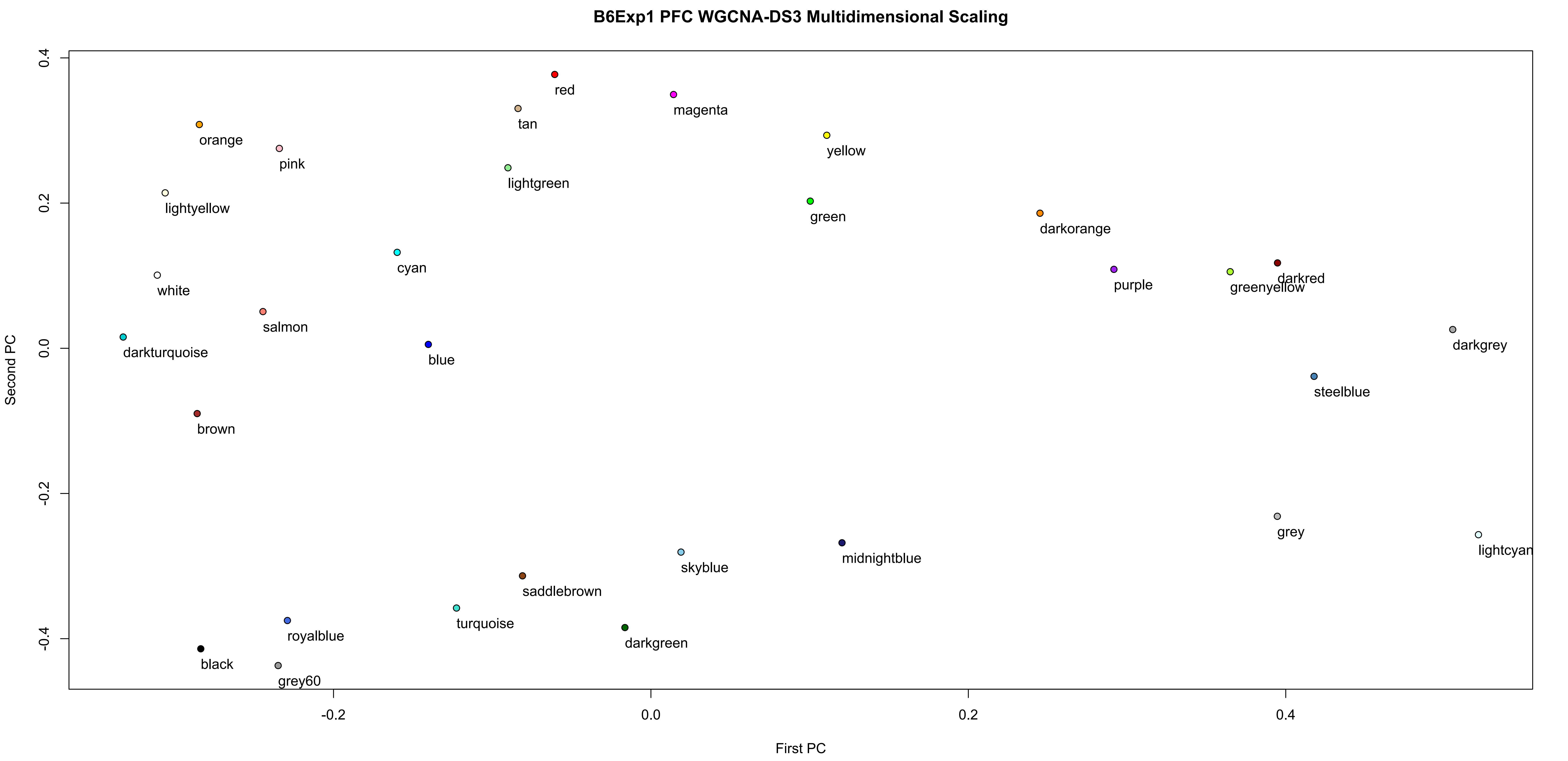
B6Exp1 PFC WGCNA-DS3 Multidimensional Scaling
First PC
Second PC
-0.2
0.0
0.2
0.4
-0.4
red
magenta
tan
orange
pink
yellow
lightgreen
lightyellow
green
darkorange
cyan
white
purple
greenyellow
darkred
salmon
darkturquoise
blue
darkgrey
steelblue
brown
grey
lightcyan
midnightblue
skyblue
saddlebrown
turquoise
royalblue
darkgreen
black
grey60

B6Exp1 PFC WGCNA-DS3 Module Eigengene Cluster Dendrogram

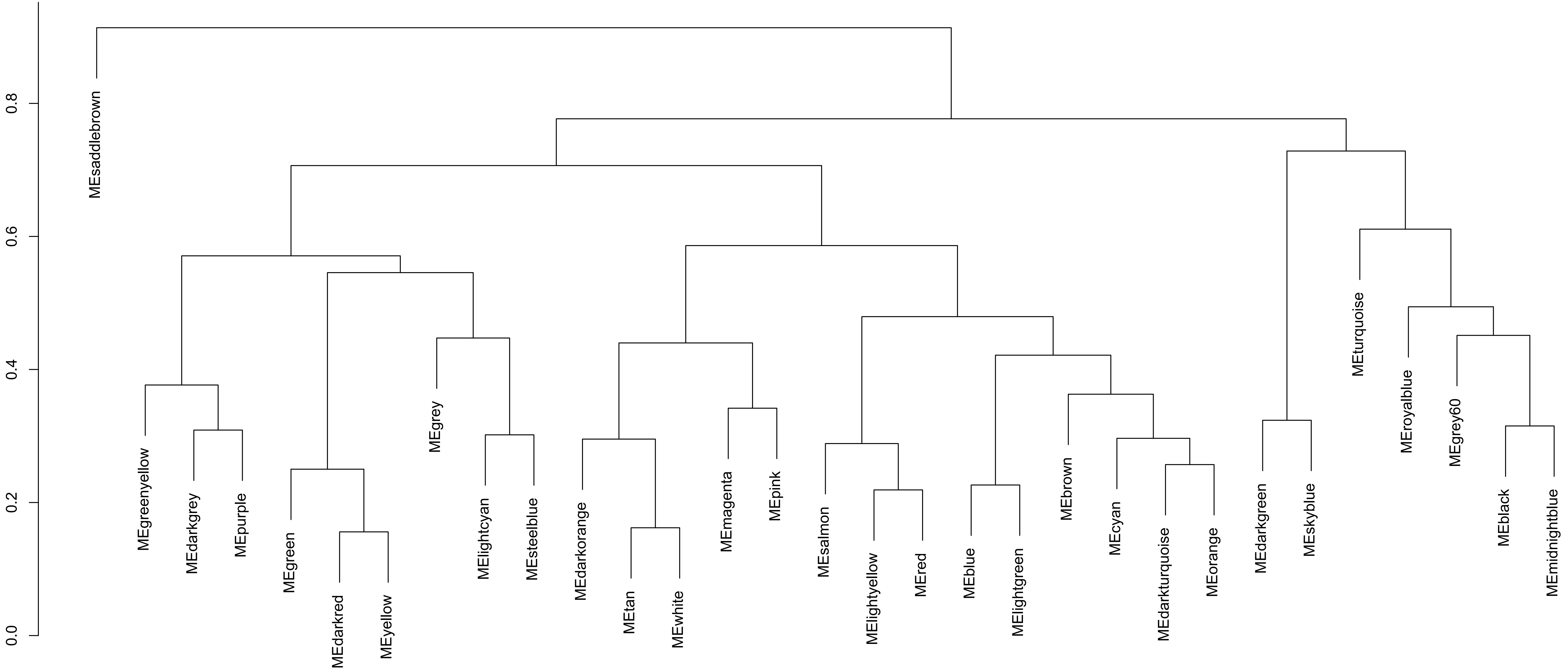

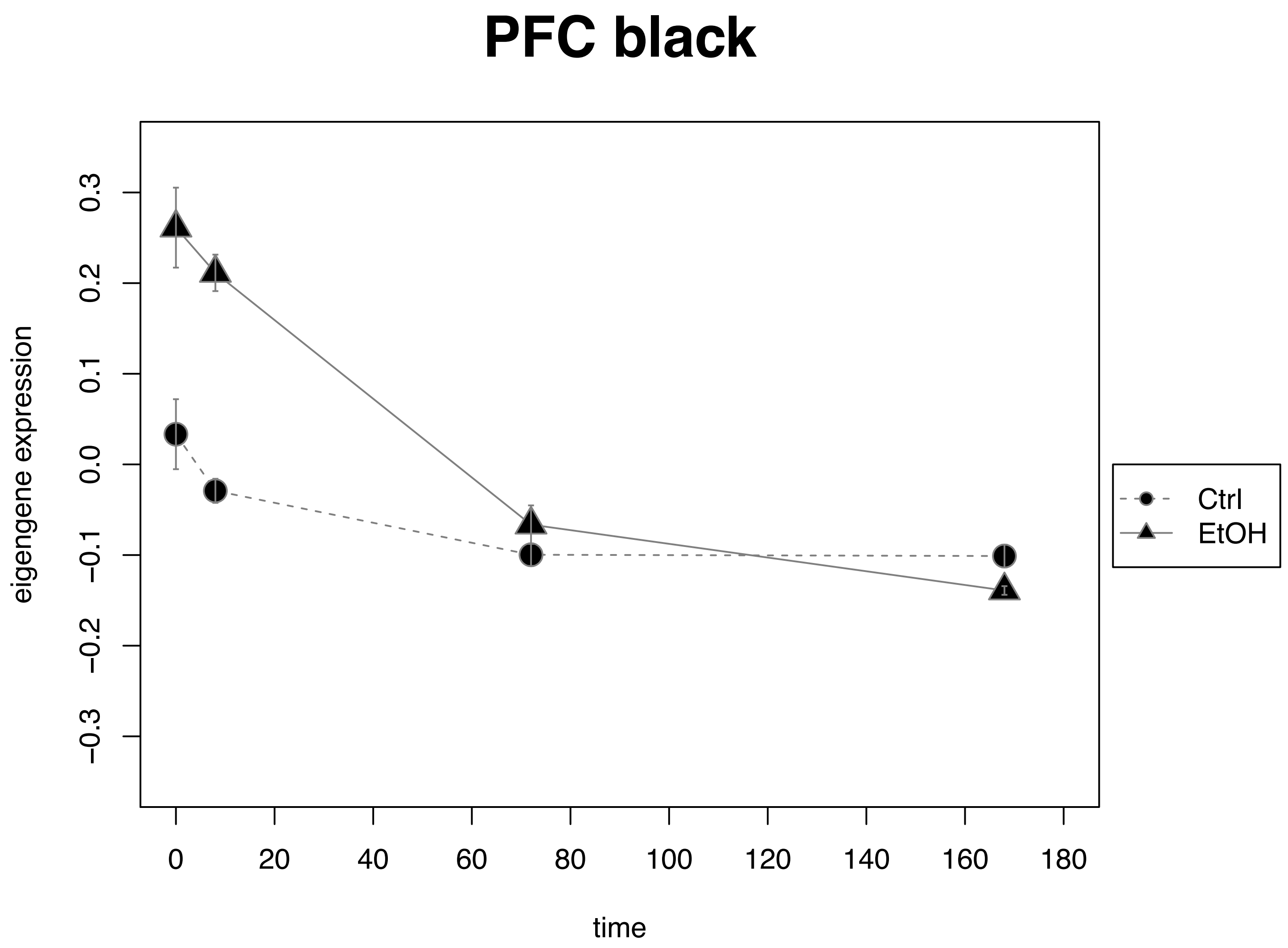
PFC black
eigengene expression
0.3
0.2
0.1
0.0
−0.1
−0.2
−0.3
0
20
40
60
80
100
120
140
160
180
time
Ctrl
EtOH

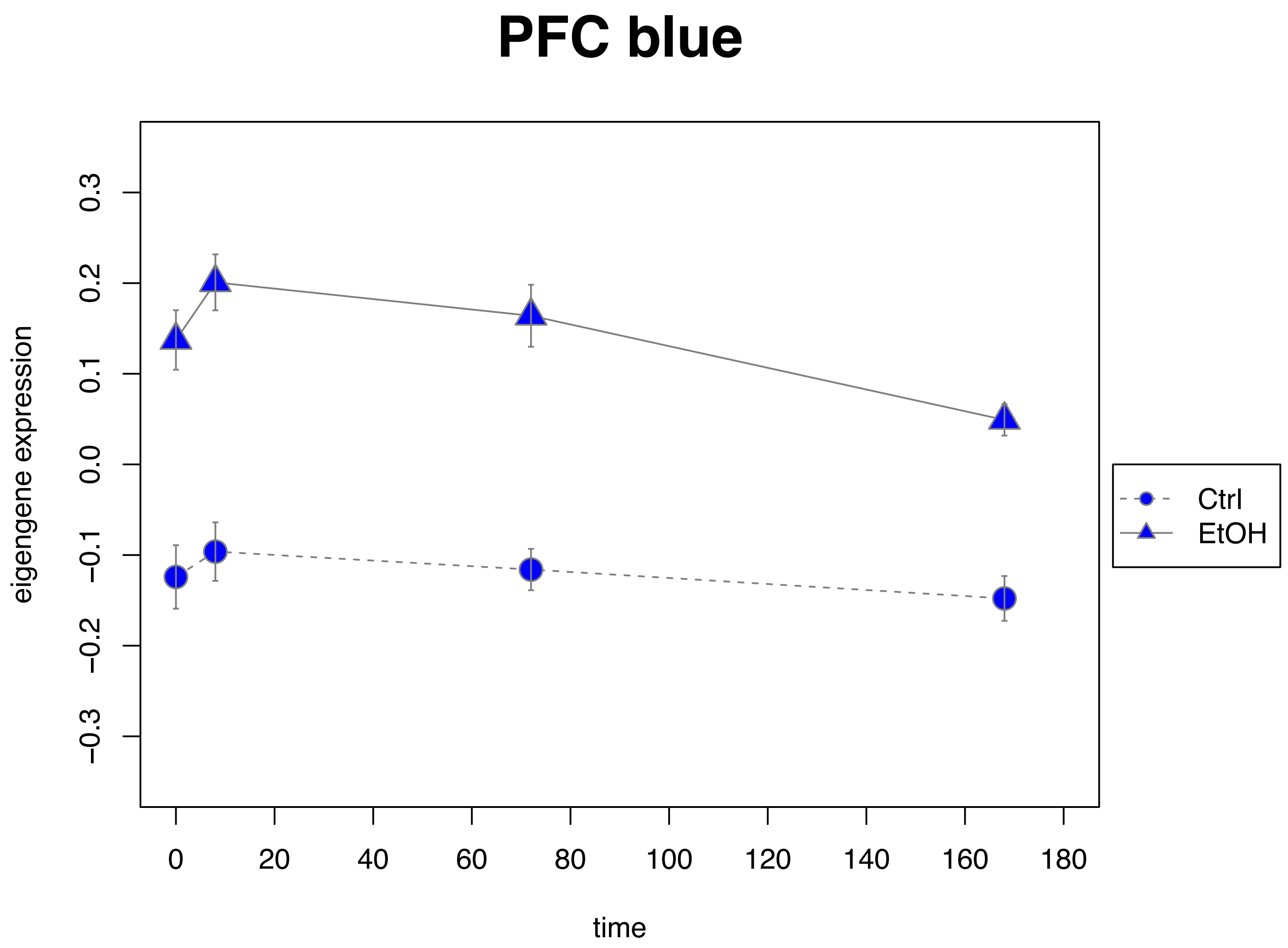
PFC blue
eigengene expression
time
Ctrl
EtOH
0.3
0.2
0.1
0.0
−0.1
−0.2
−0.3
0
20
40
60
80
100
120
140
160
180

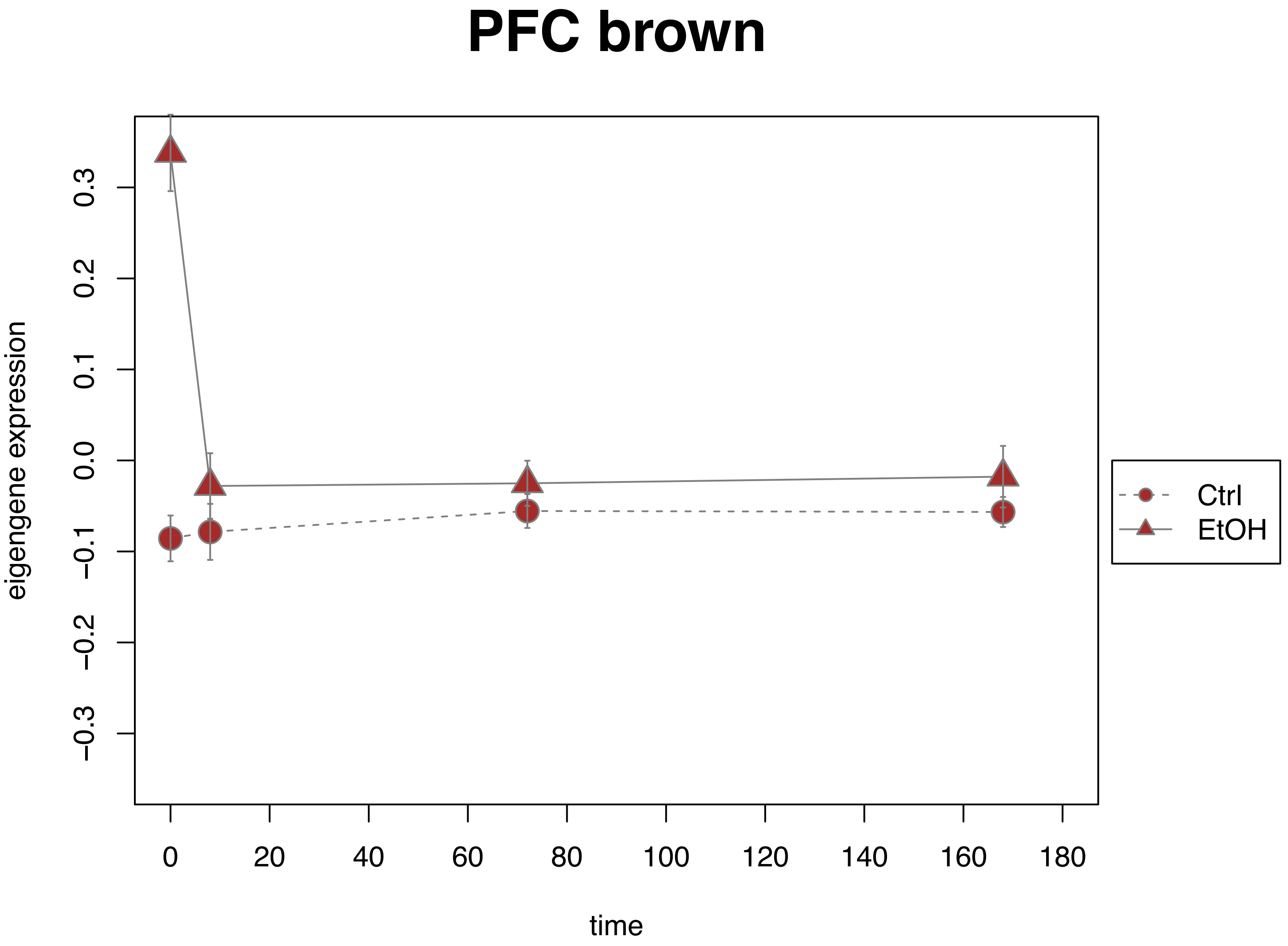

PFC brown
eigengene expression
time
Ctrl
EtOH
0.3
0.2
0.1
0.0
−0.1
−0.2
−0.3
0
20
40
60
80
100
120
140
160
180

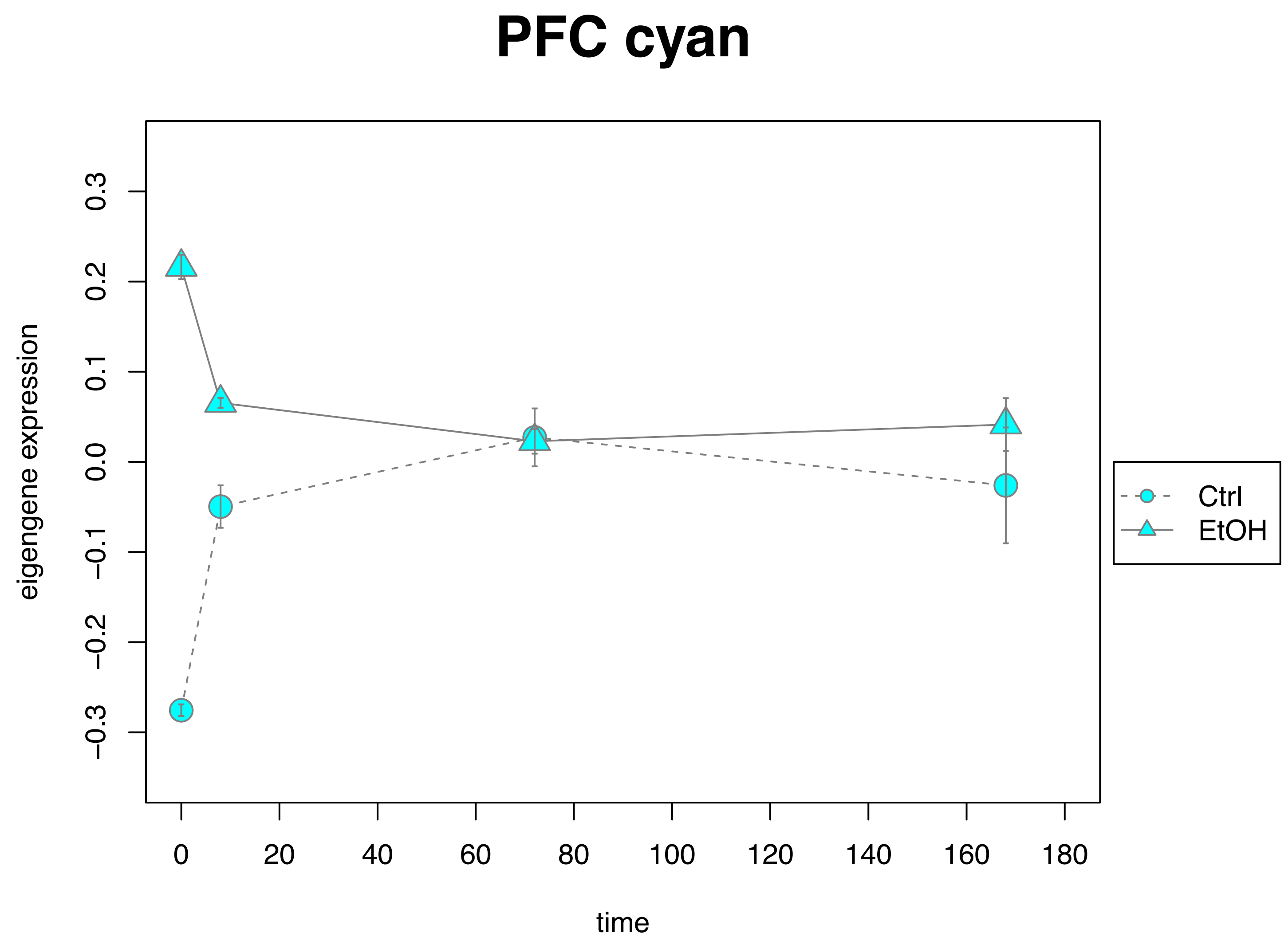
PFC cyan
eigengene expression
0.3
0.2
0.1
0.0
−0.1
−0.2
−0.3
0
20
40
60
80
100
120
140
160
180
time
Ctrl
EtOH

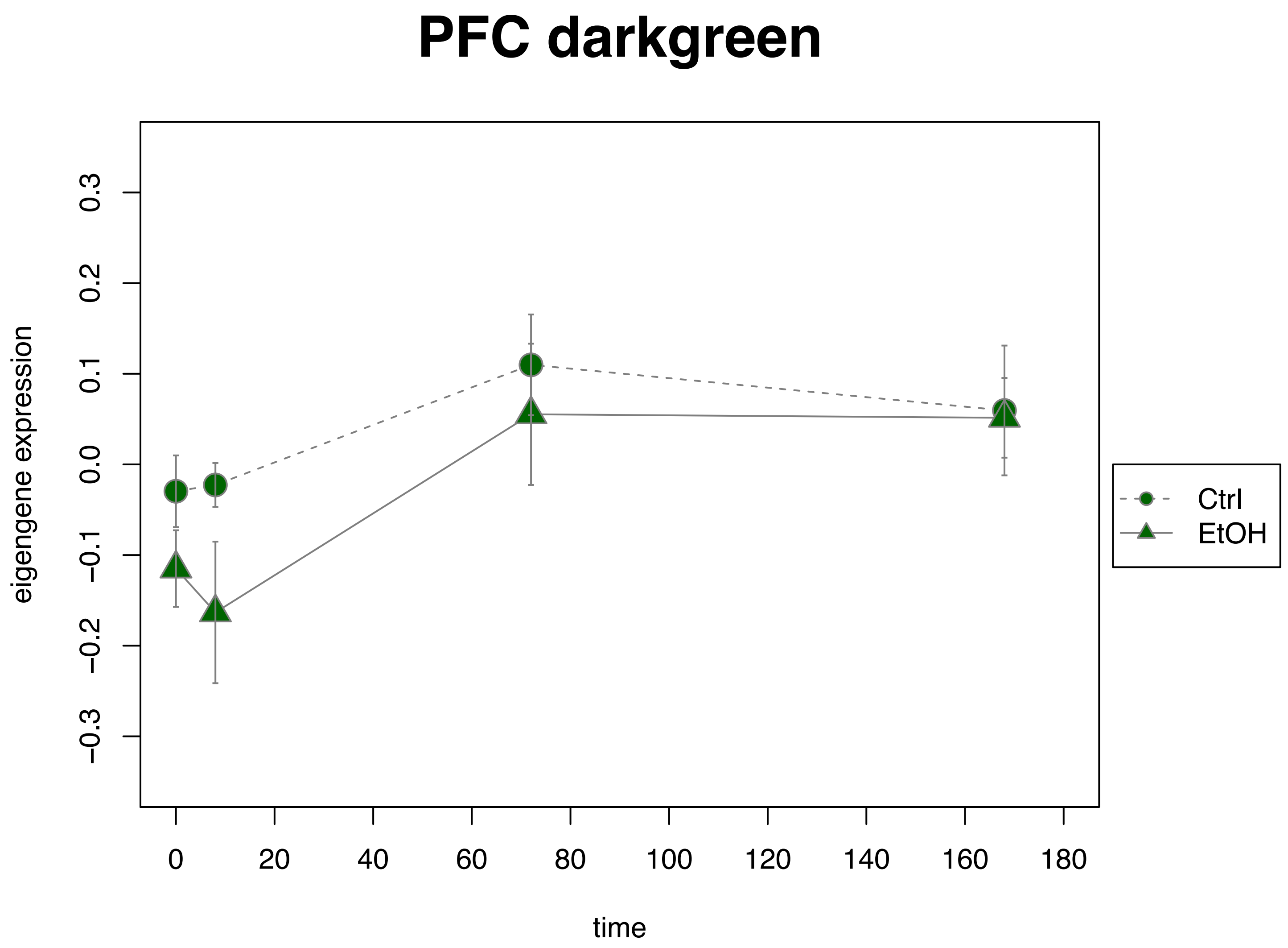

PFC darkgreen
eigengene expression
0.3
0.2
0.1
0.0
−0.1
−0.2
−0.3
0
20
40
60
80
100
120
140
160
180
time
Ctrl
EtOH

PFC darkgrey

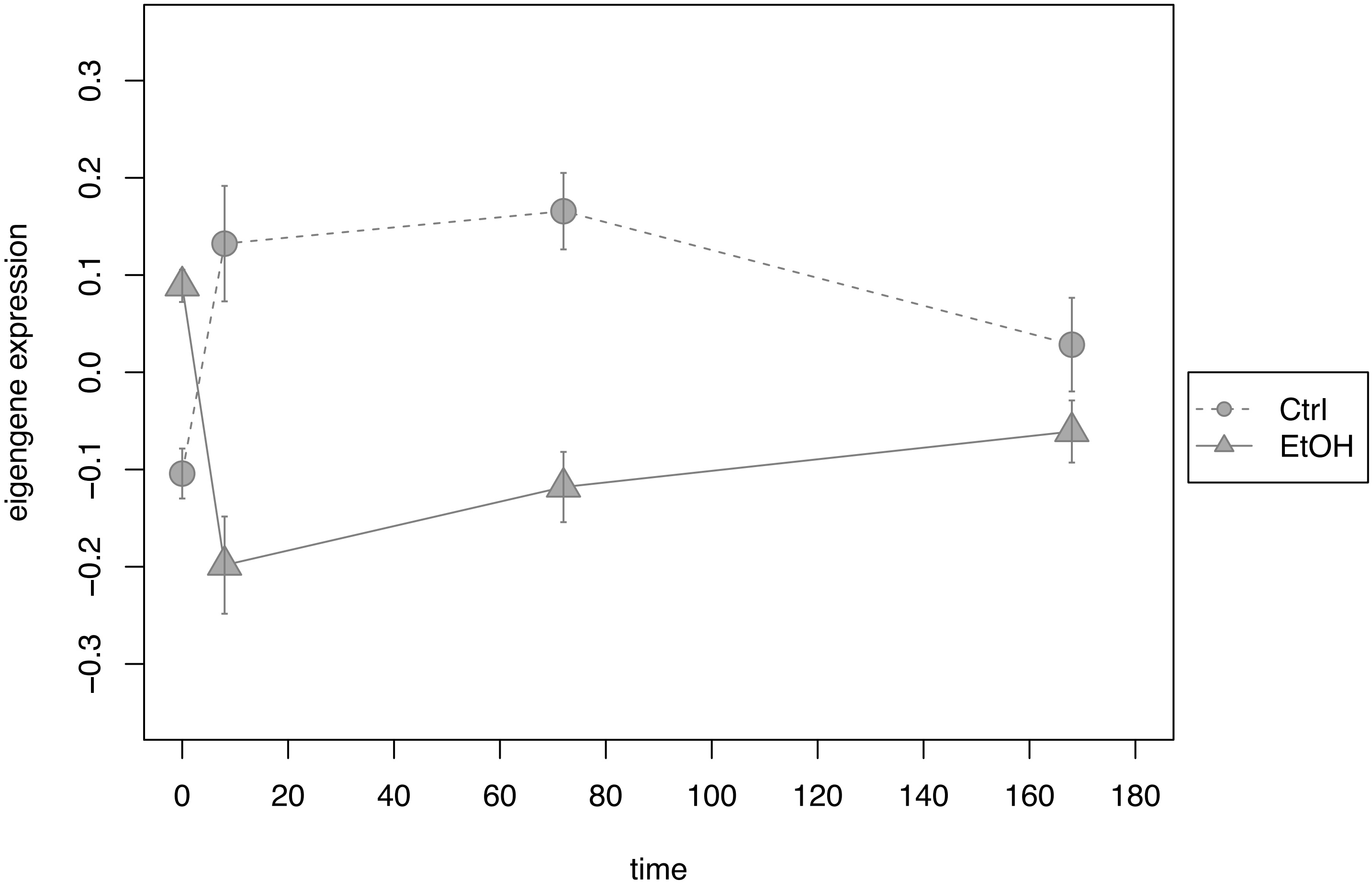

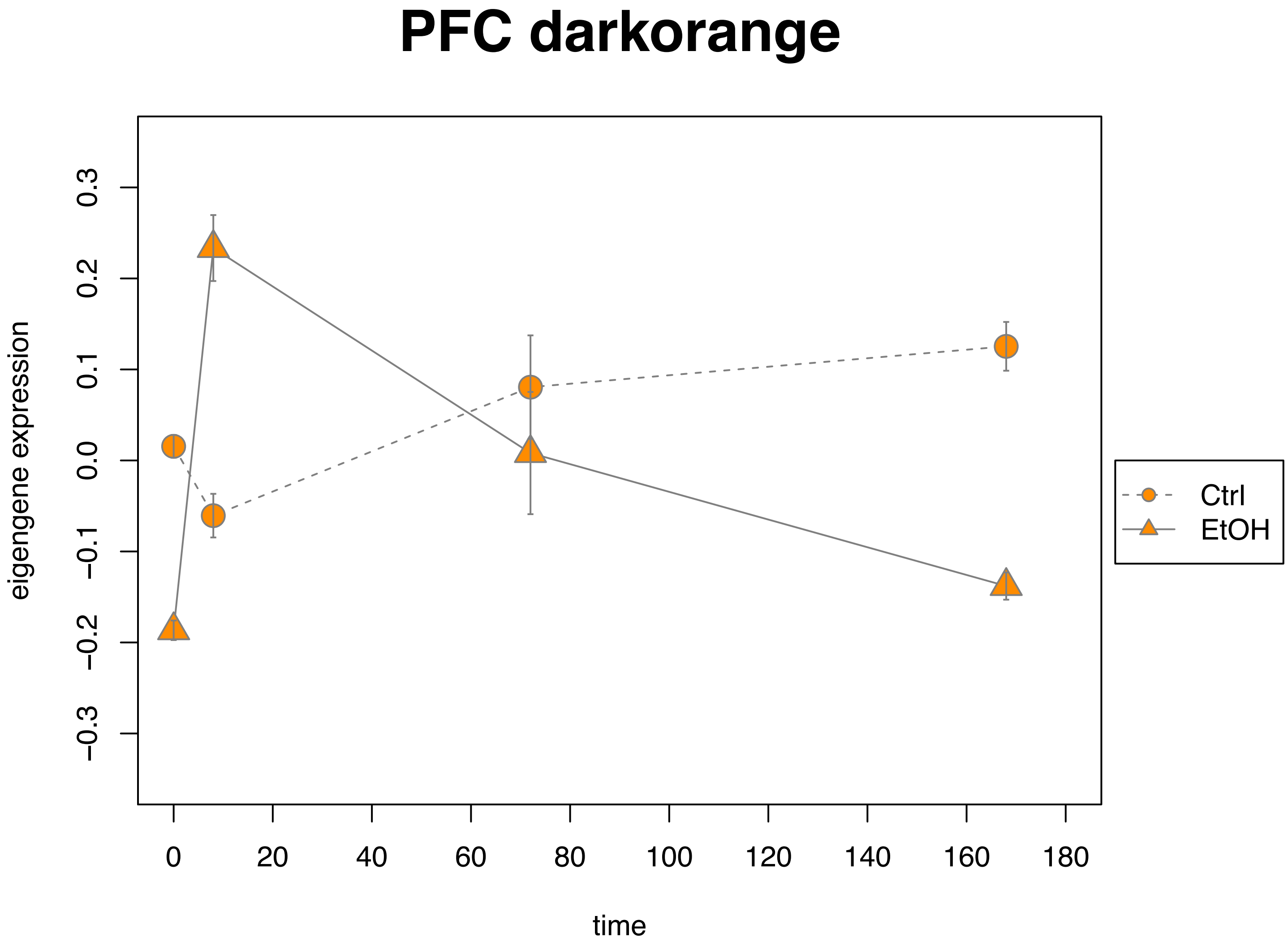
PFC darkorange
eigengene expression
0.3
0.2
0.1
0.0
−0.1
−0.2
−0.3
0
20
40
60
80
100
120
140
160
180
time
Ctrl
EtOH

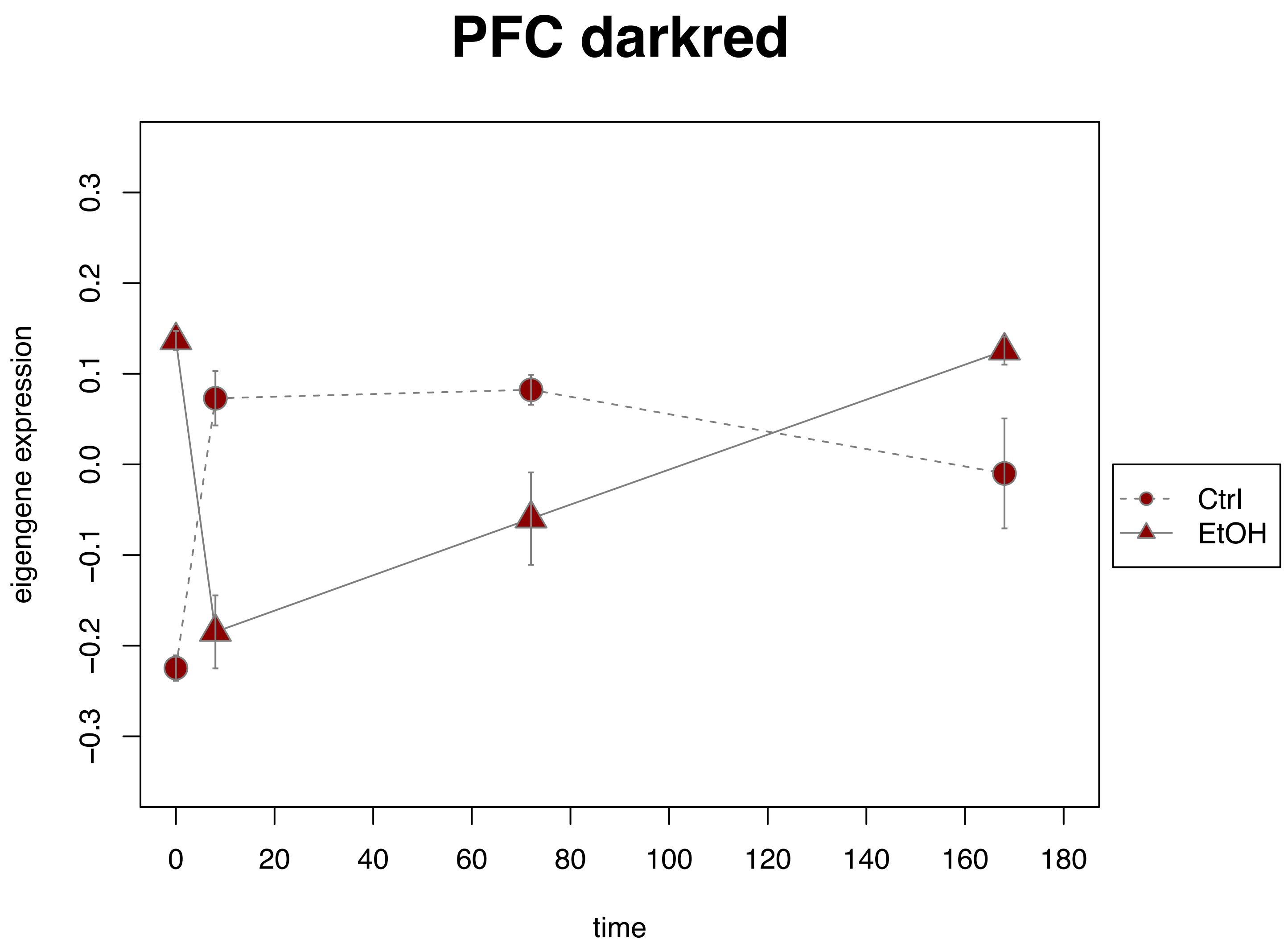

PFC darkred
eigengene expression
time
0.3
0.2
0.1
0.0
−0.1
−0.2
−0.3
0
20
40
60
80
100
120
140
160
180
Ctrl
EtOH

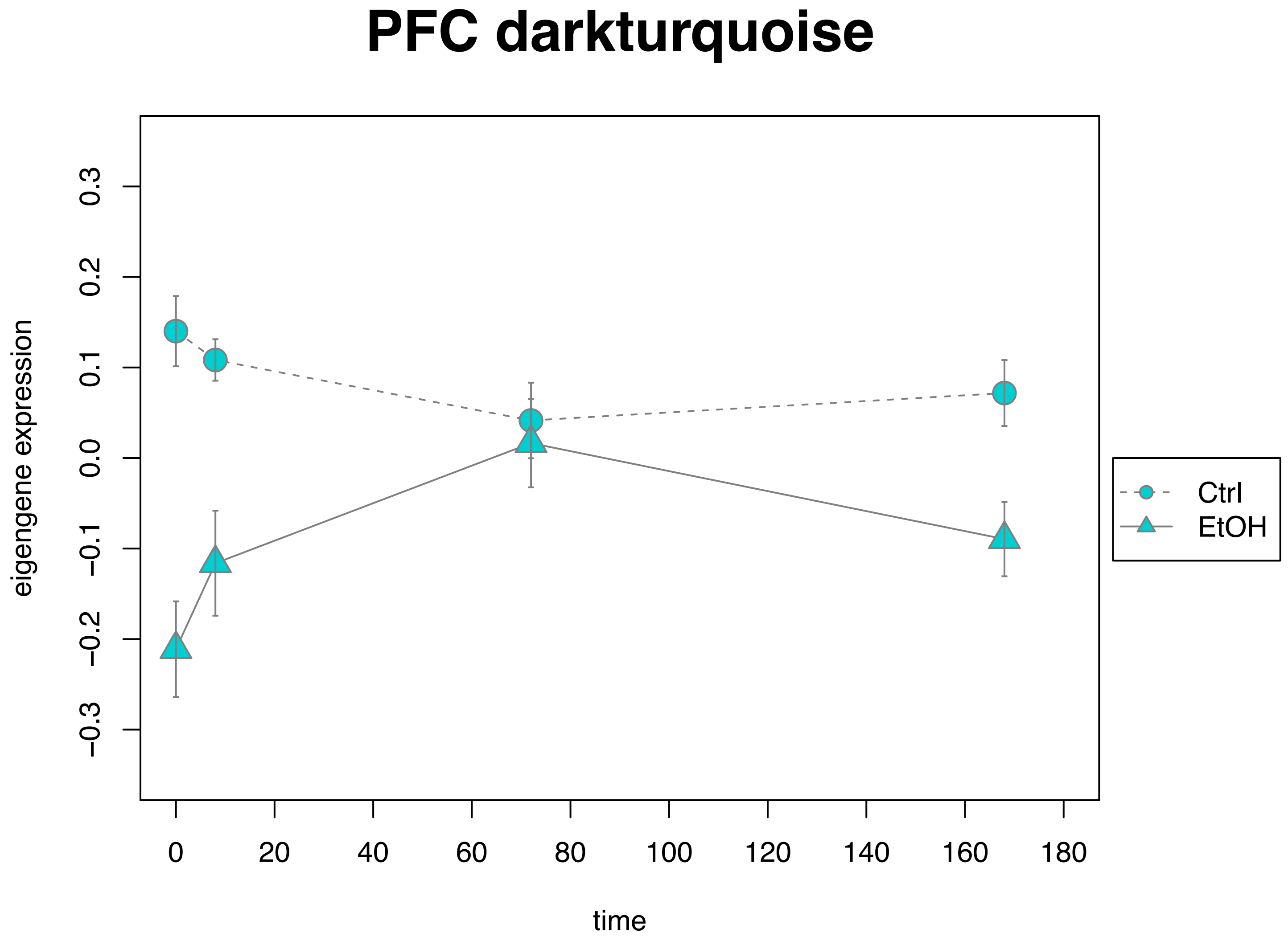
PFC darkturquoise
eigengene expression
0.3
0.2
0.1
0.0
−0.1
−0.2
−0.3
0
20
40
60
80
100
120
140
160
180
time
Ctrl
EtOH

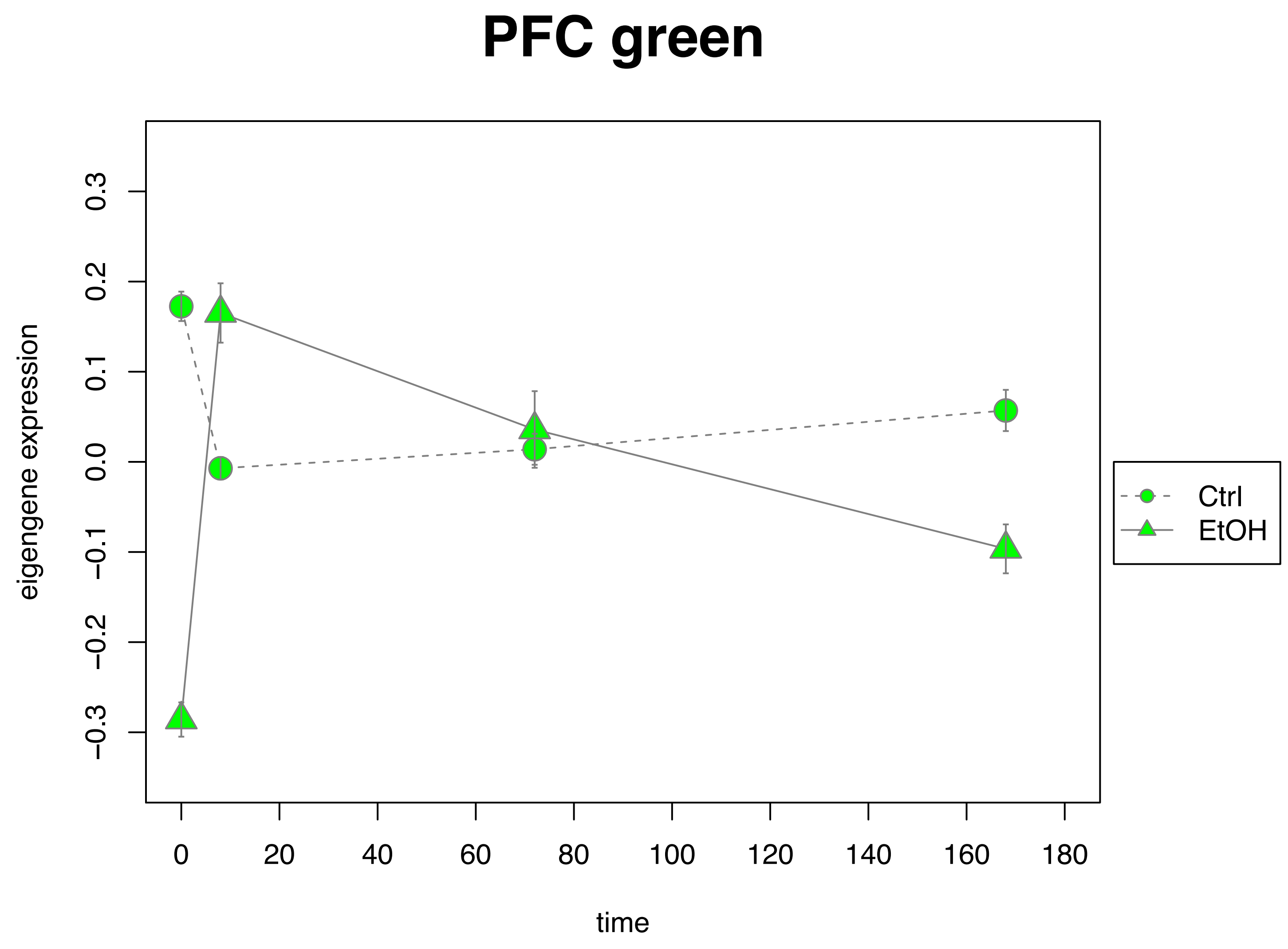
PFC green
eigengene expression
time
0.3
0.2
0.1
0.0
−0.1
−0.2
−0.3
0
20
40
60
80
100
120
140
160
180
Ctrl
EtOH

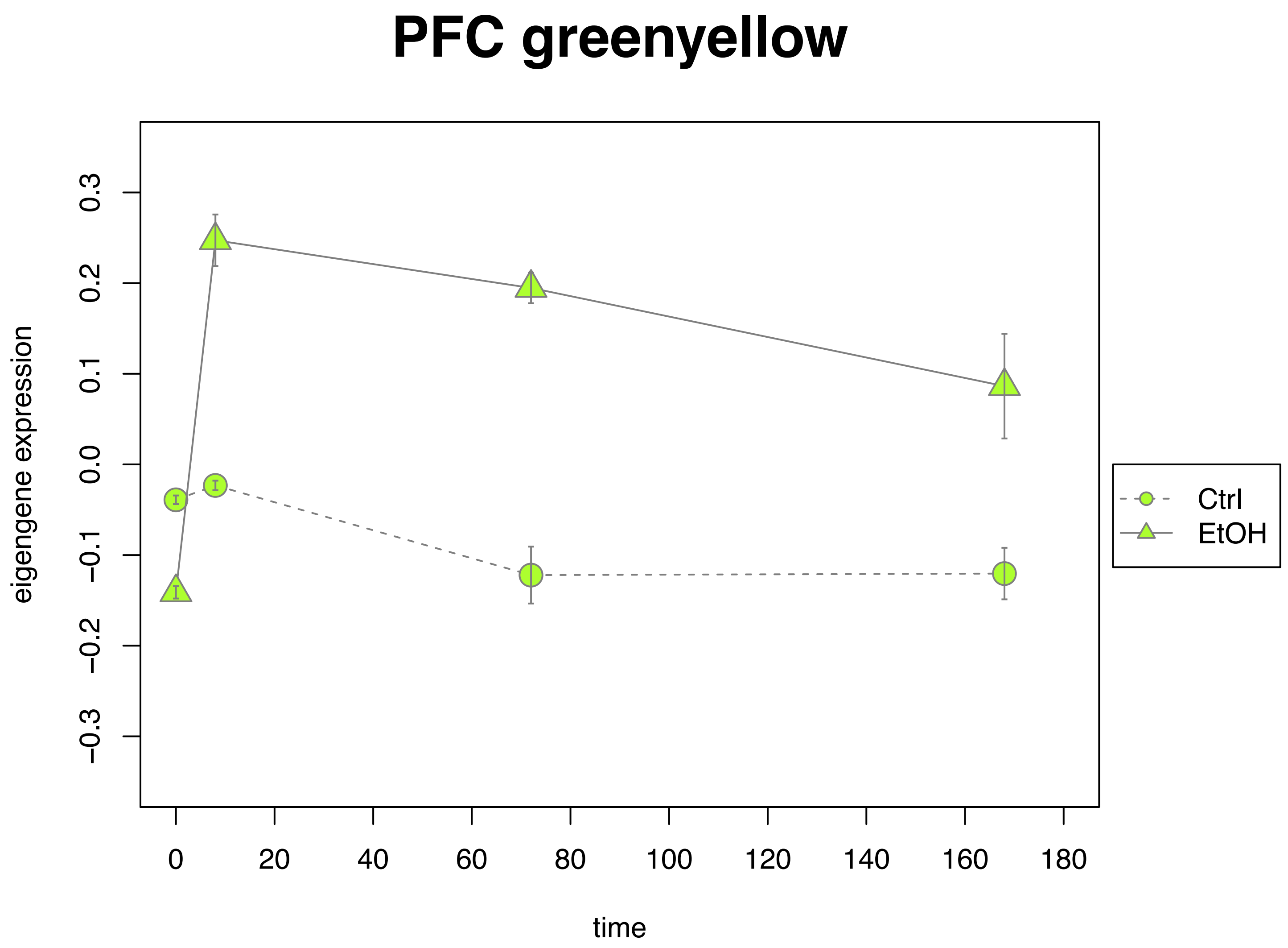
PFC greenyellow
eigengene expression
time
0.3
0.2
0.1
0.0
−0.1
−0.2
−0.3
0
20
40
60
80
100
120
140
160
180
Ctrl
EtOH

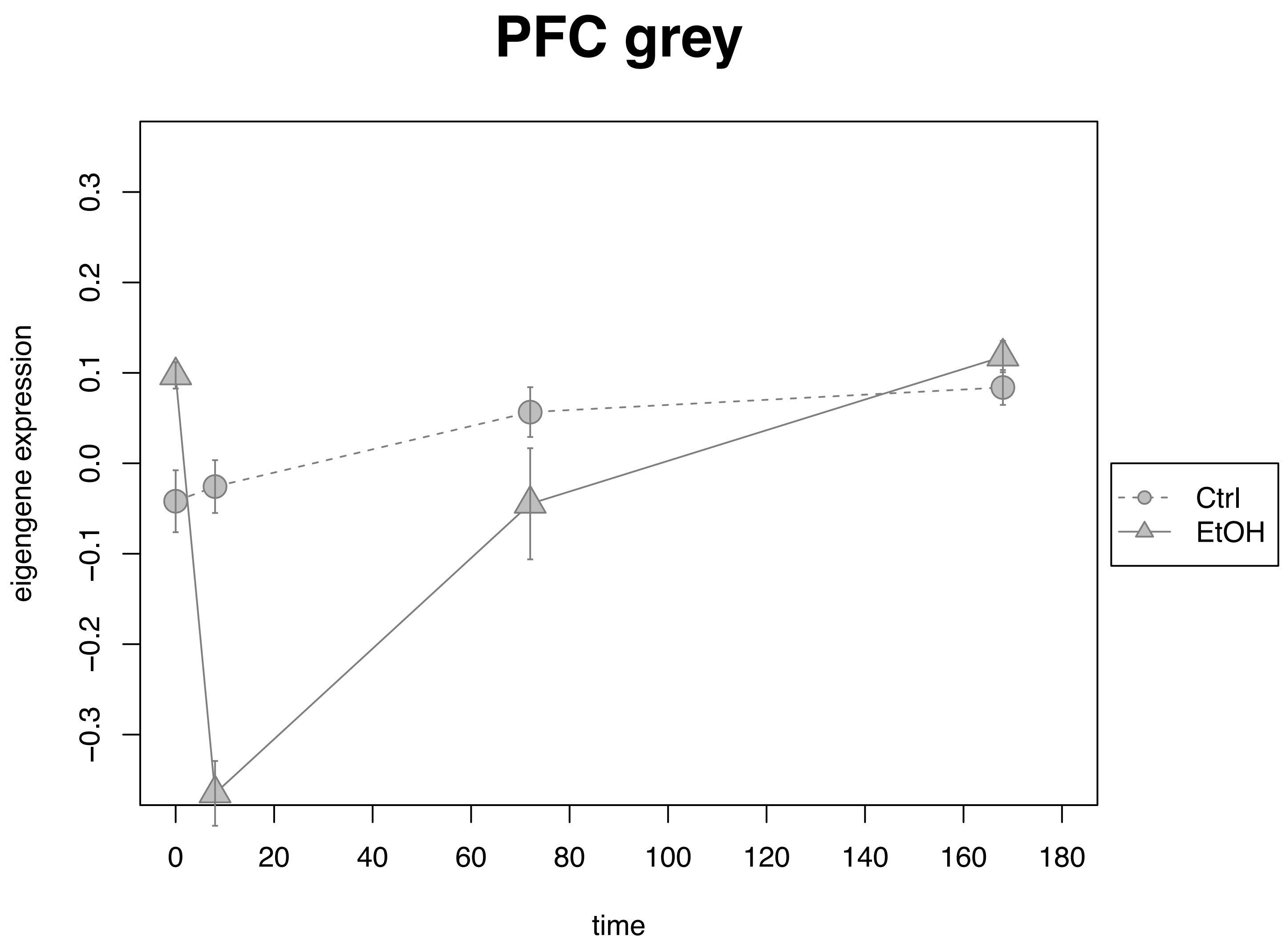
PFC grey
eigengene expression
0.3
0.2
0.1
0.0
−0.1
−0.2
−0.3
0
20
40
60
80
100
120
140
160
180
time
Ctrl
EtOH

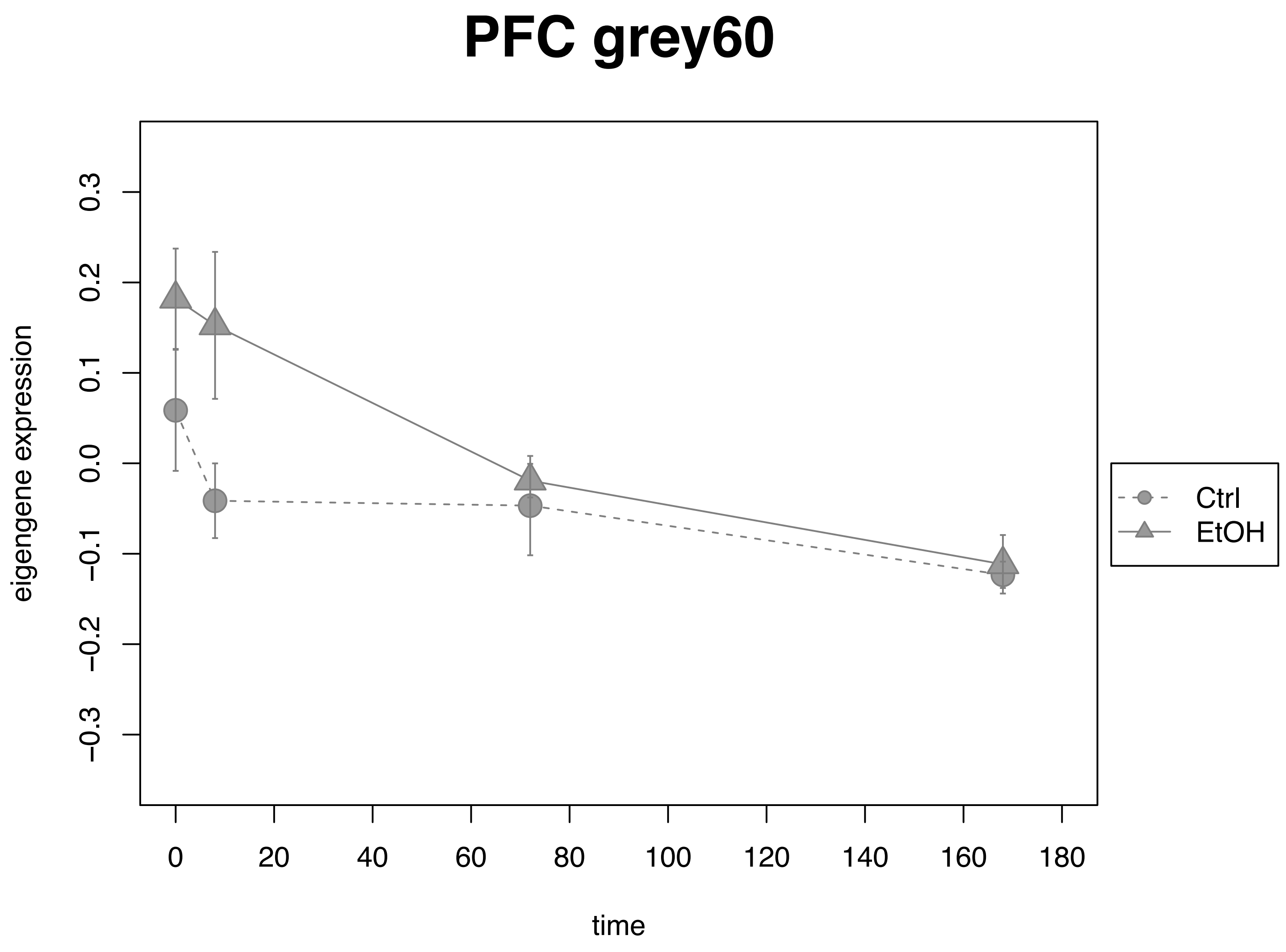

PFC grey60
eigengene expression
0.3
0.2
0.1
0.0
−0.1
−0.2
−0.3
0
20
40
60
80
100
120
140
160
180
time
Ctrl
EtOH

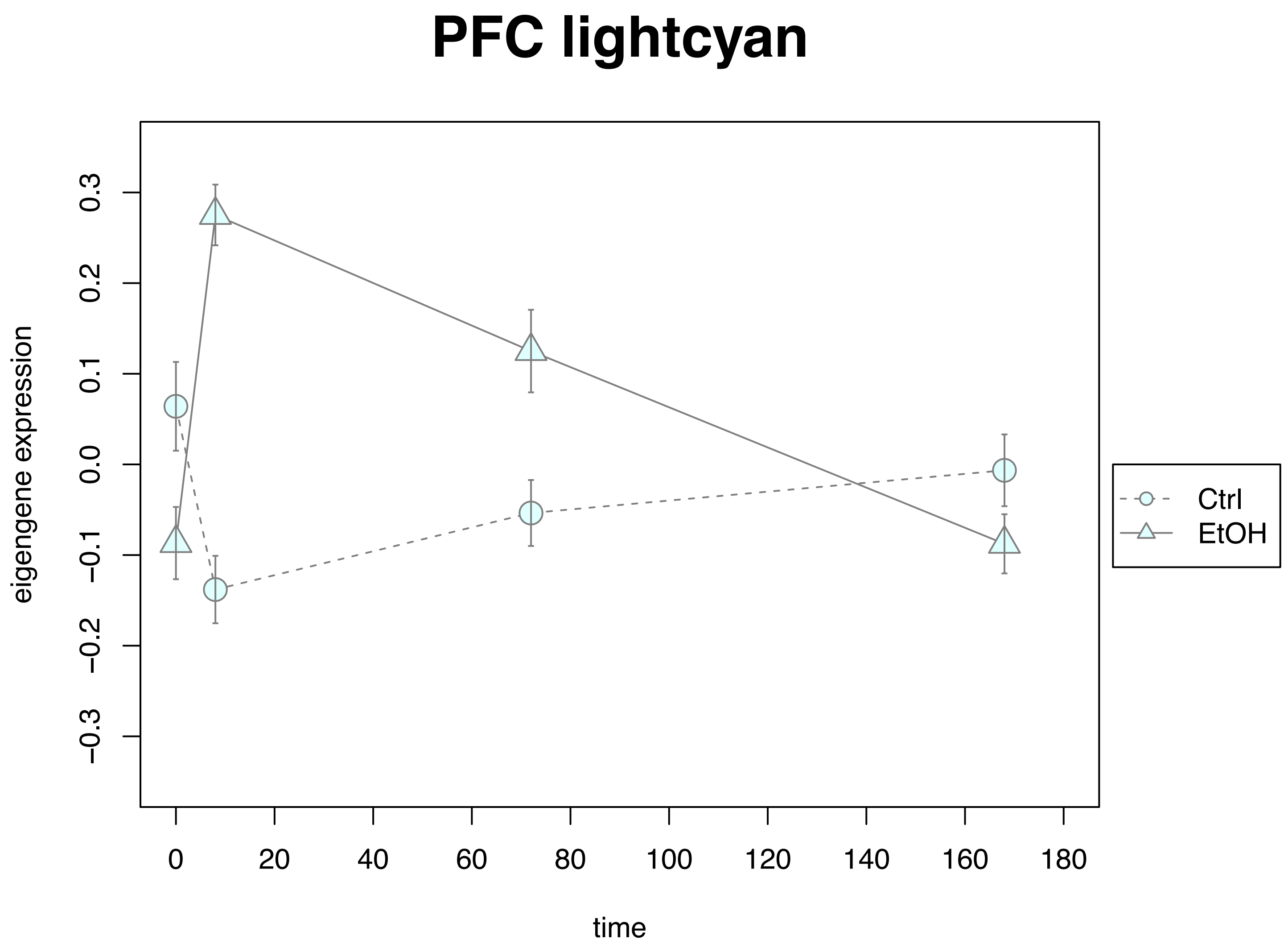
PFC lightcyan
eigengene expression
0.3
0.2
0.1
0.0
−0.1
−0.2
−0.3
0
20
40
60
80
100
120
140
160
180
time
Ctrl
EtOH

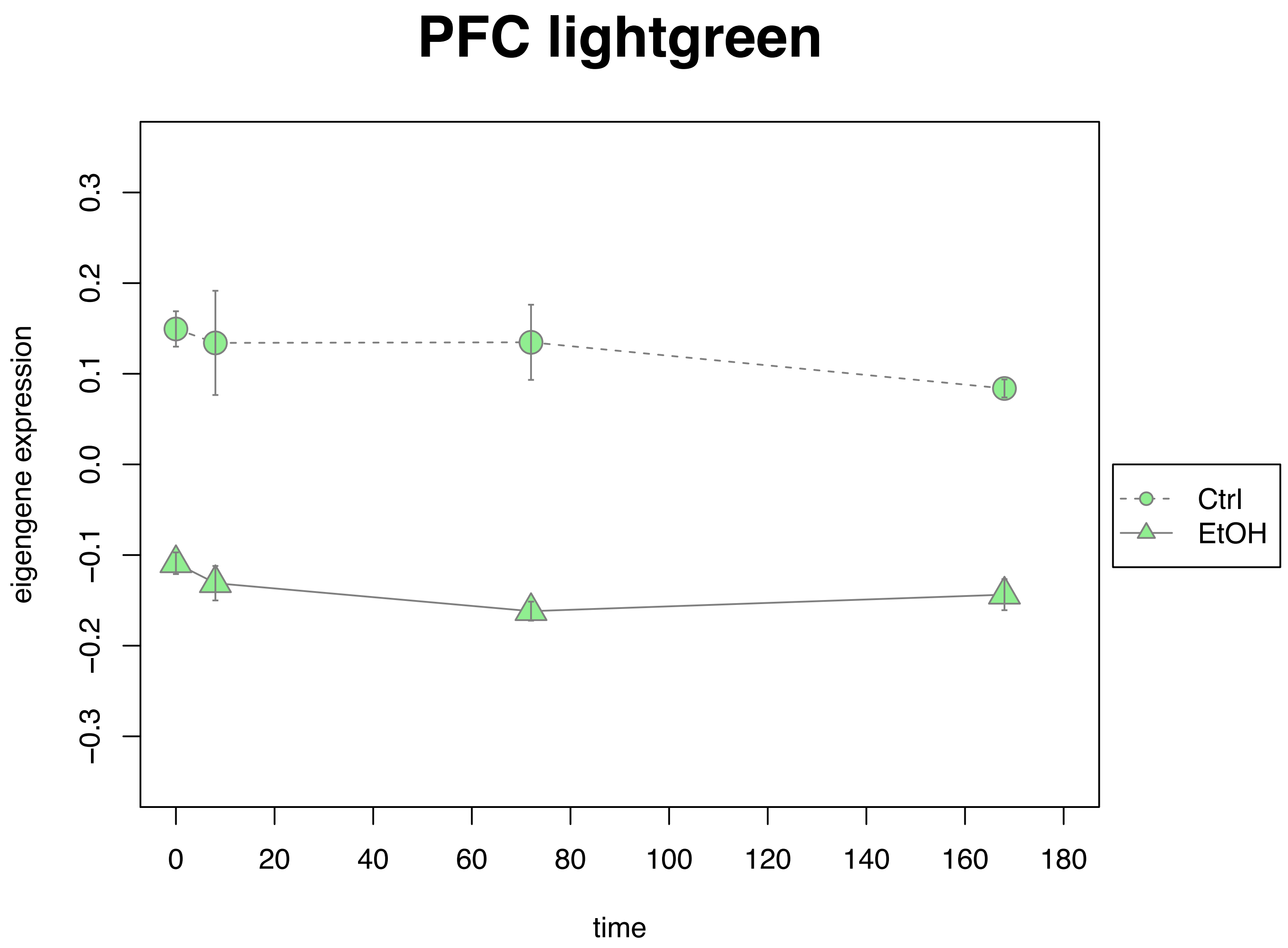
PFC lightgreen
eigengene expression
0.3
0.2
0.1
0.0
−0.1
−0.2
−0.3
0
20
40
60
80
100
120
140
160
180
time
Ctrl
EtOH

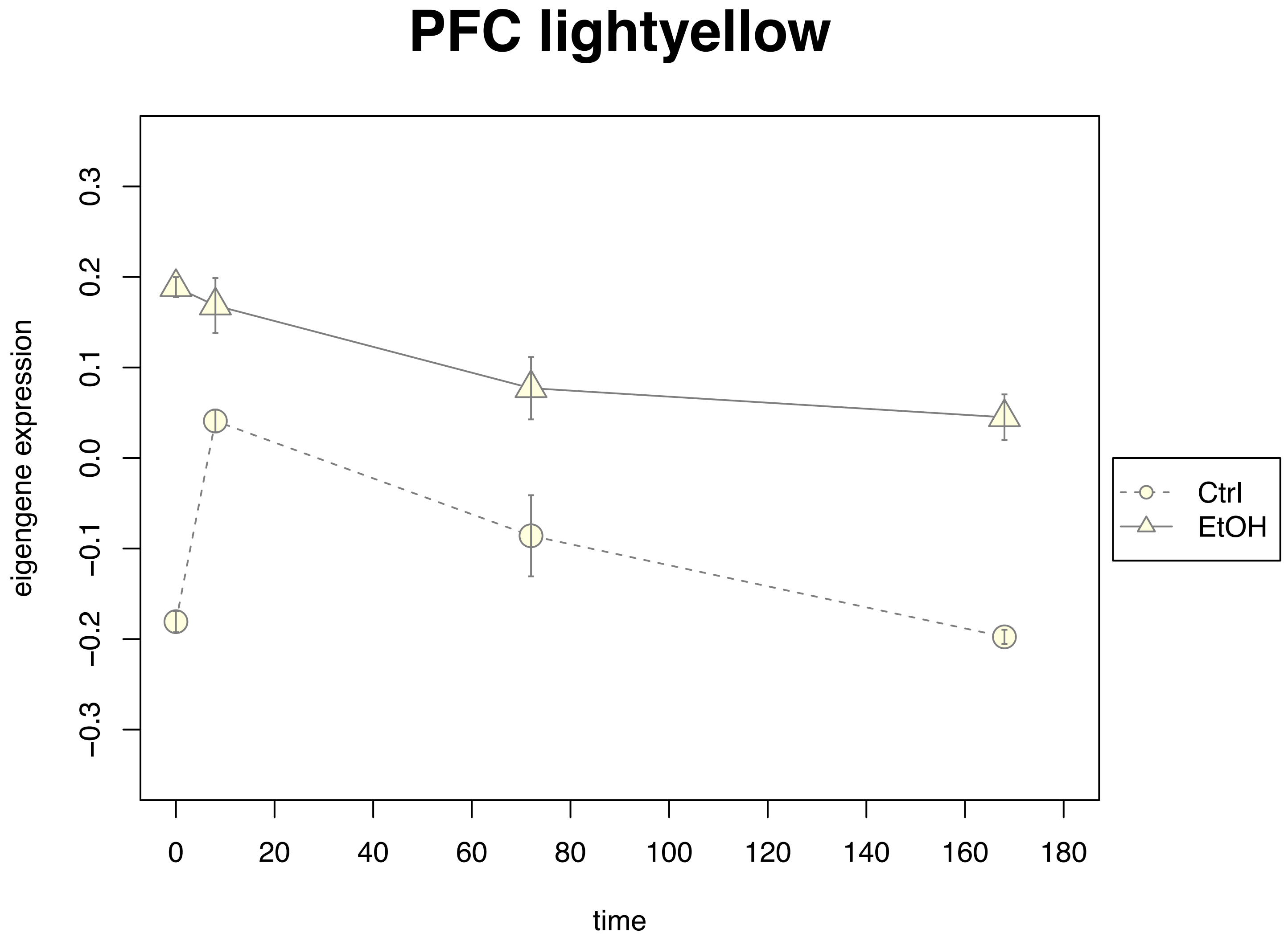
PFC lightyellow
eigengene expression
0.3
0.2
0.1
0.0
−0.1
−0.2
−0.3
0
20
40
60
80
100
120
140
160
180
time
Ctrl
EtOH

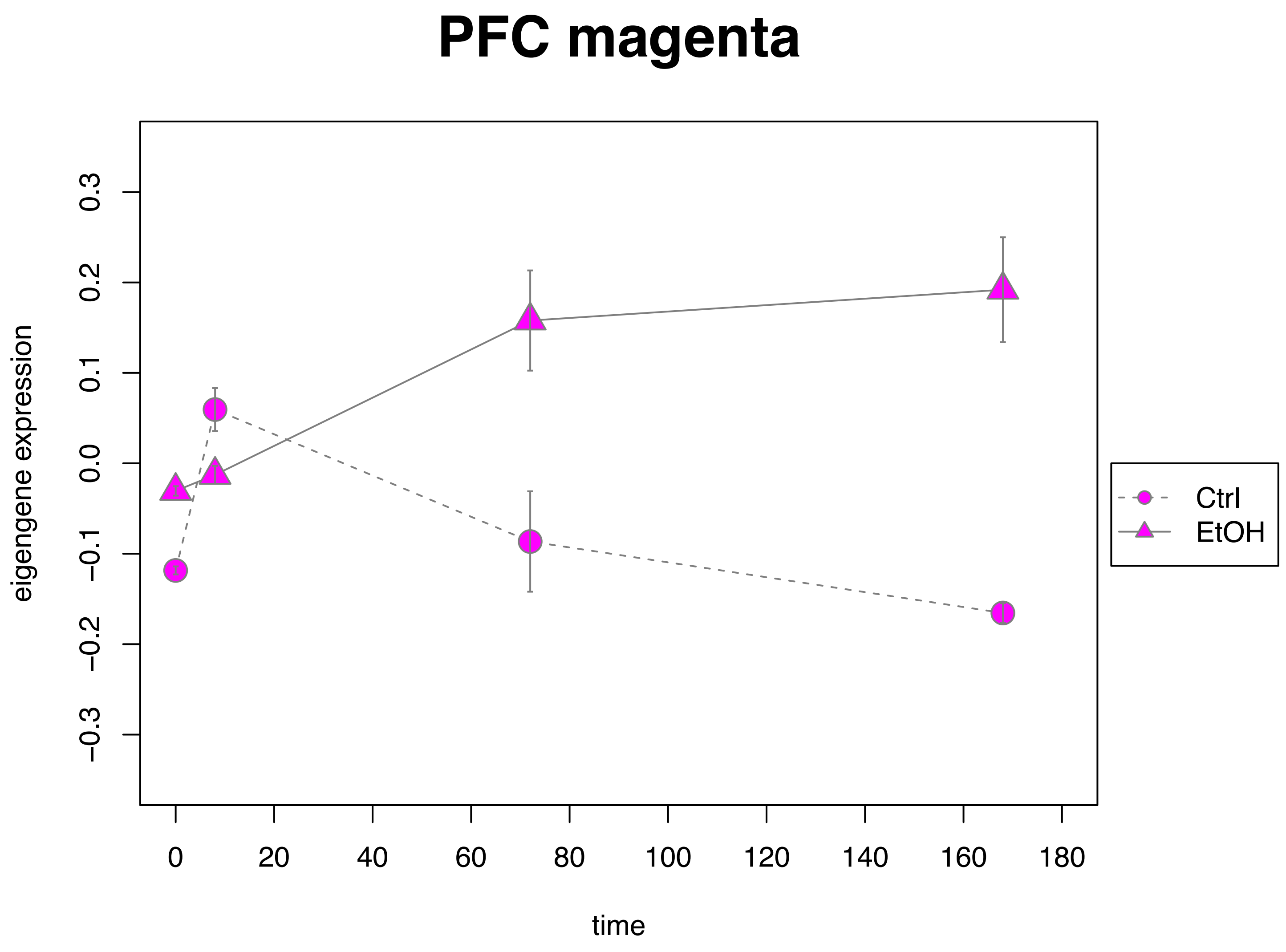
PFC magenta
eigengene expression
0.3
0.2
0.1
0.0
−0.1
−0.2
−0.3
0
20
40
60
80
100
120
140
160
180
time
Ctrl
EtOH

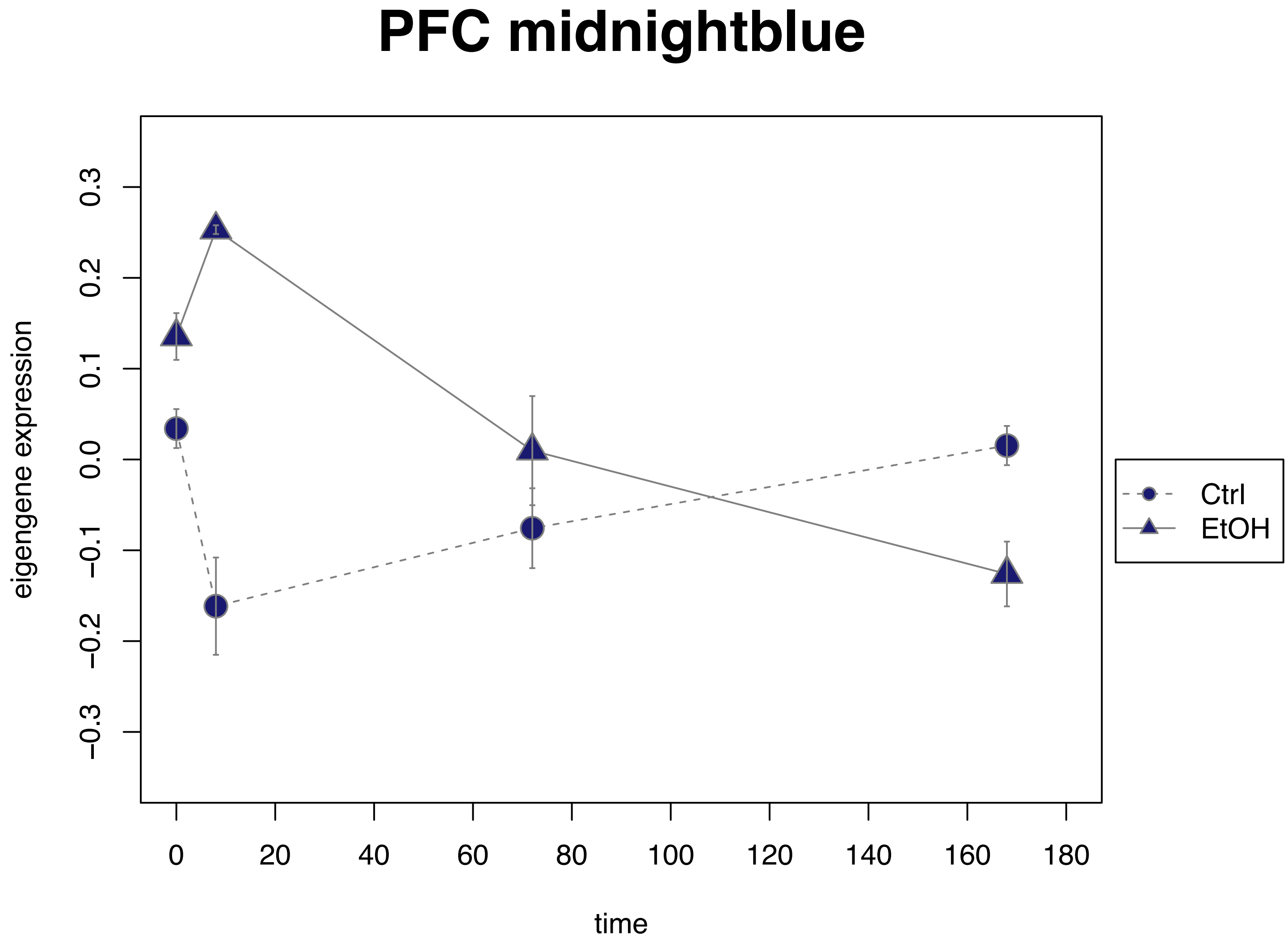

PFC midnightblue
eigengene expression
time
0.3
0.2
0.1
0.0
−0.1
−0.2
−0.3
0
20
40
60
80
100
120
140
160
180
Ctrl
EtOH

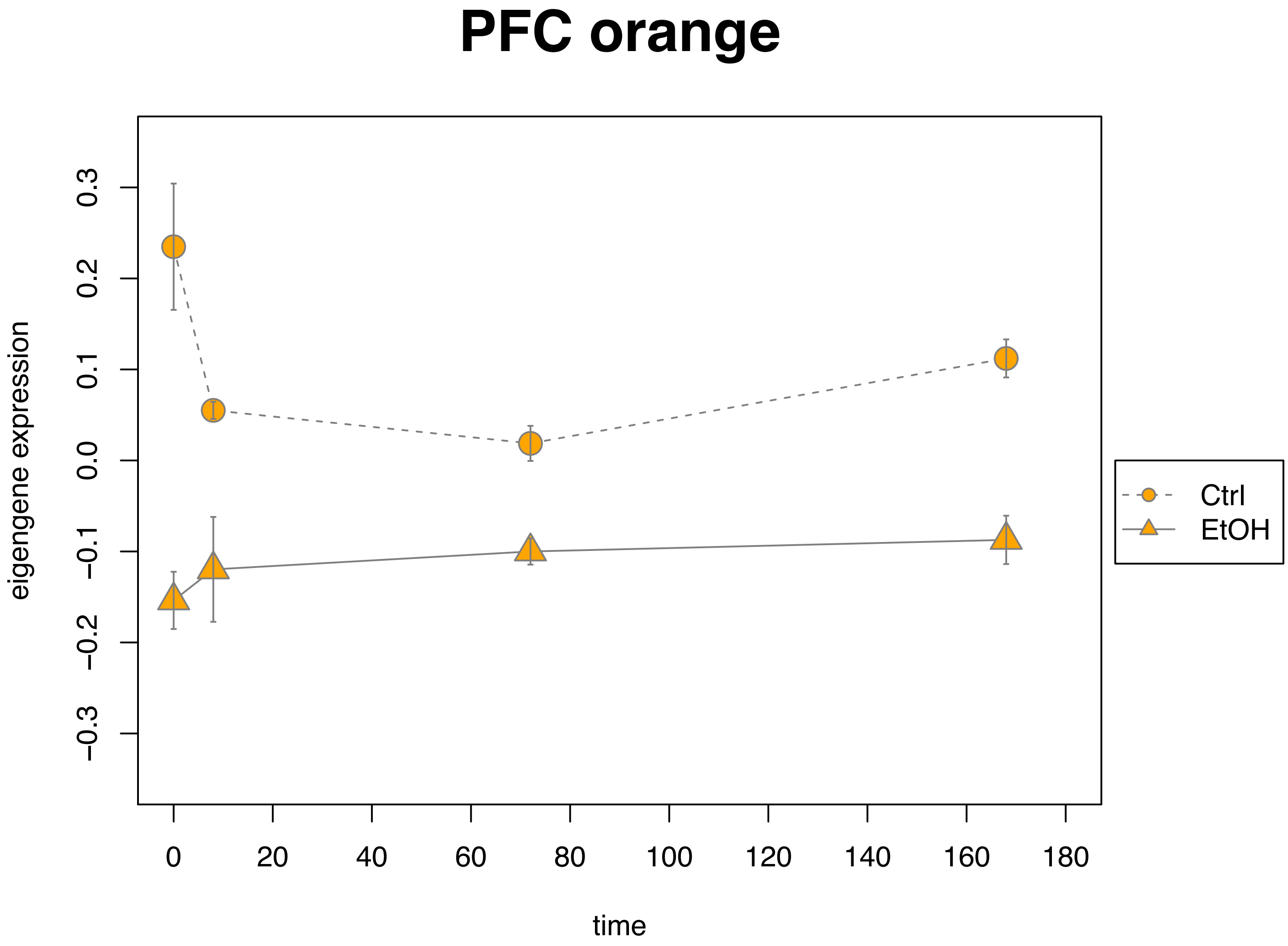

PFC orange
eigengene expression
0.3
0.2
0.1
0.0
−0.1
−0.2
−0.3
0
20
40
60
80
100
120
140
160
180
time
Ctrl
EtOH

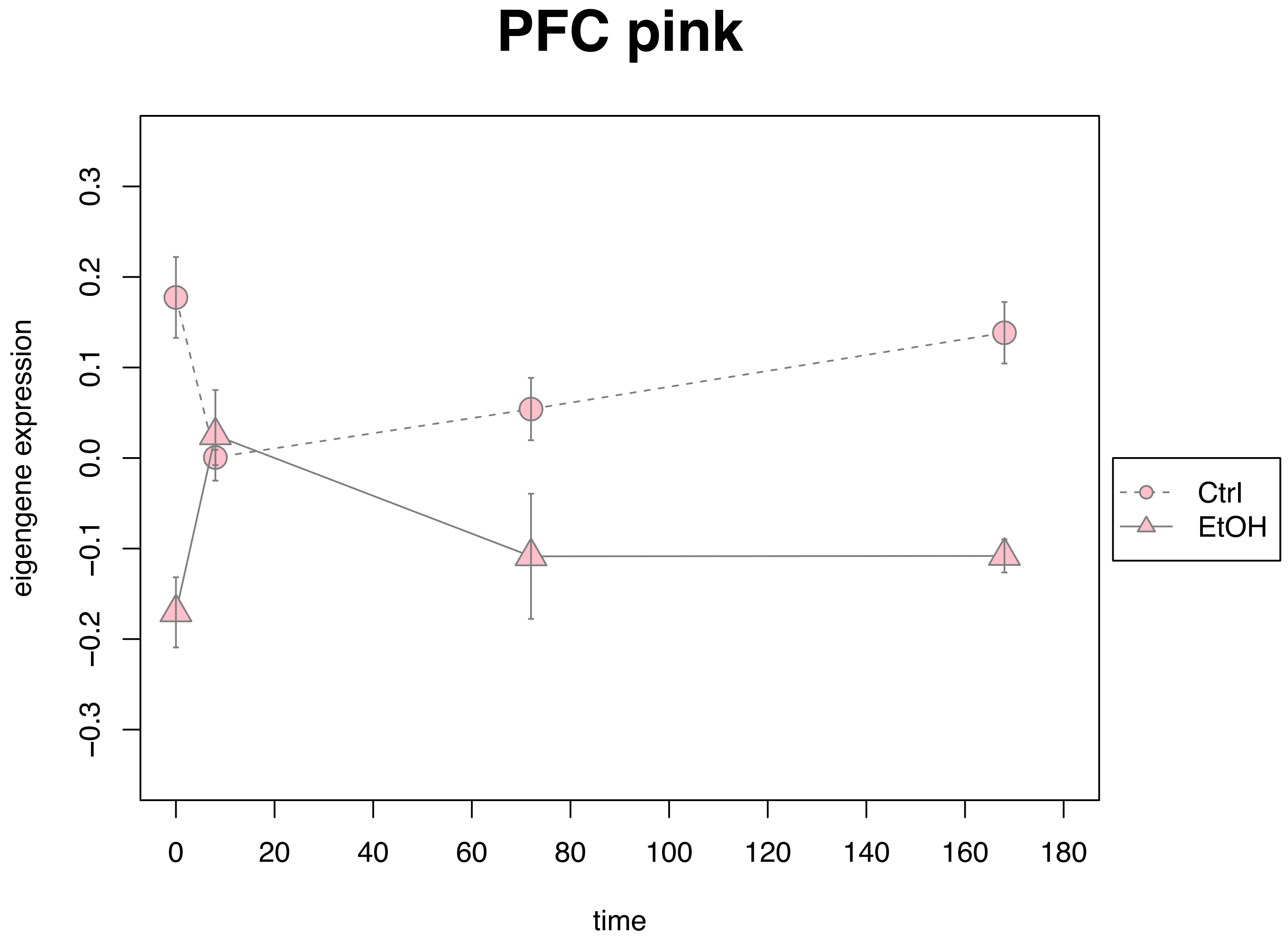
PFC pink
eigengene expression
0.3
0.2
0.1
0.0
−0.1
−0.2
−0.3
0
20
40
60
80
100
120
140
160
180
time
Ctrl
EtOH

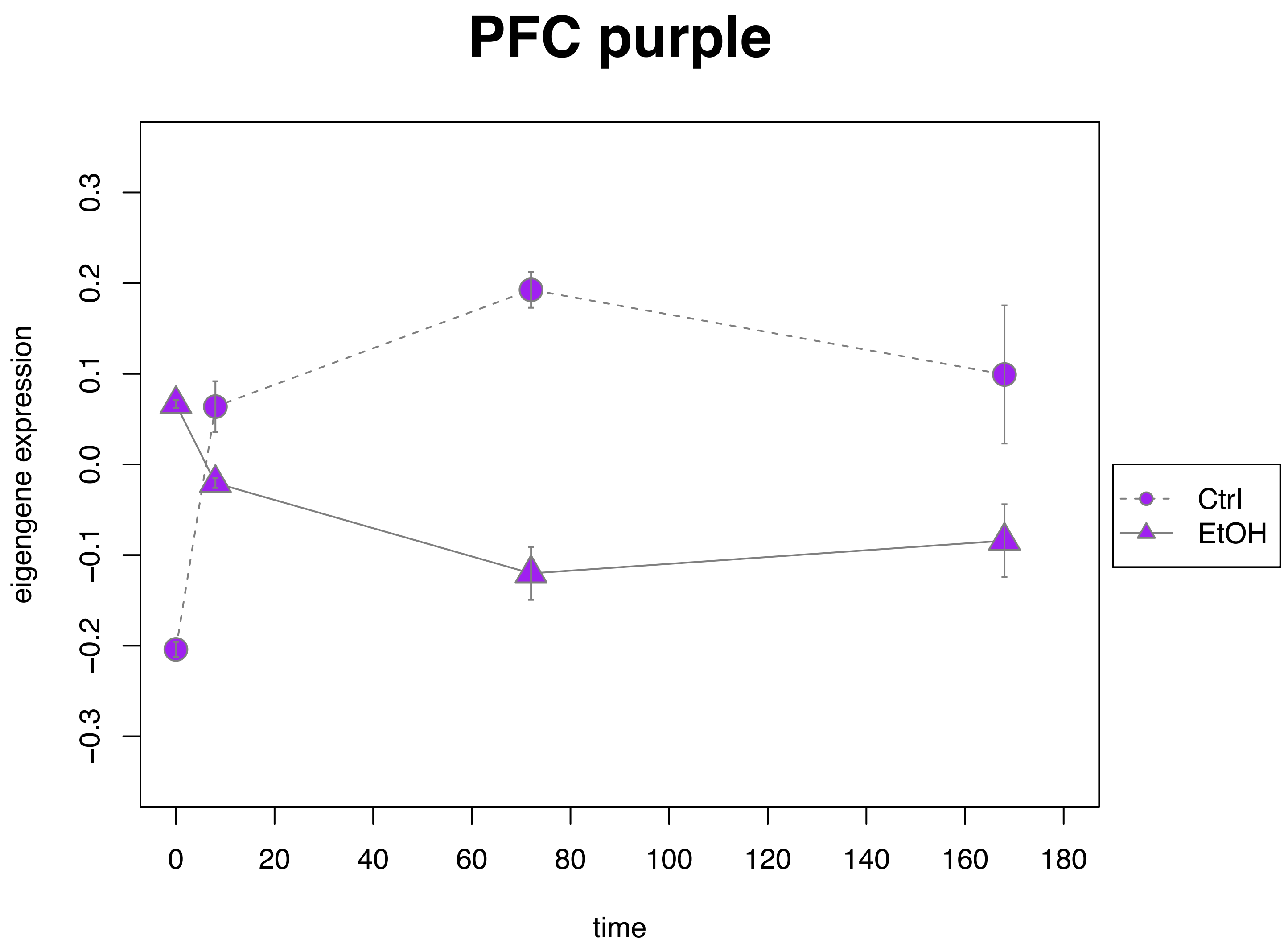
PFC purple
eigengene expression
0.3
0.2
0.1
0.0
−0.1
−0.2
−0.3
0
20
40
60
80
100
120
140
160
180
time
Ctrl
EtOH

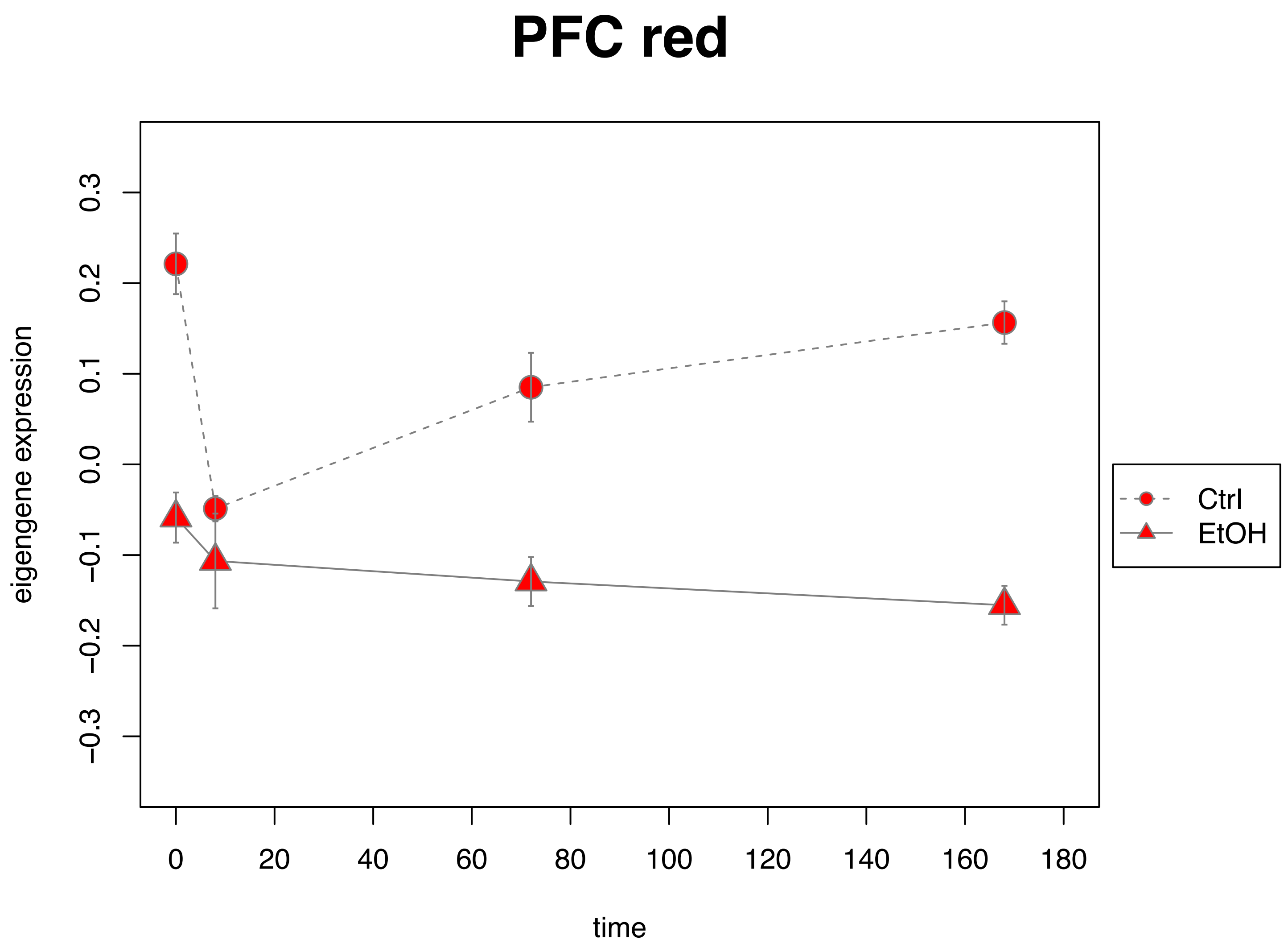
PFC red
eigengene expression
0.3
0.2
0.1
0.0
−0.1
−0.2
−0.3
0
20
40
60
80
100
120
140
160
180
time
Ctrl
EtOH

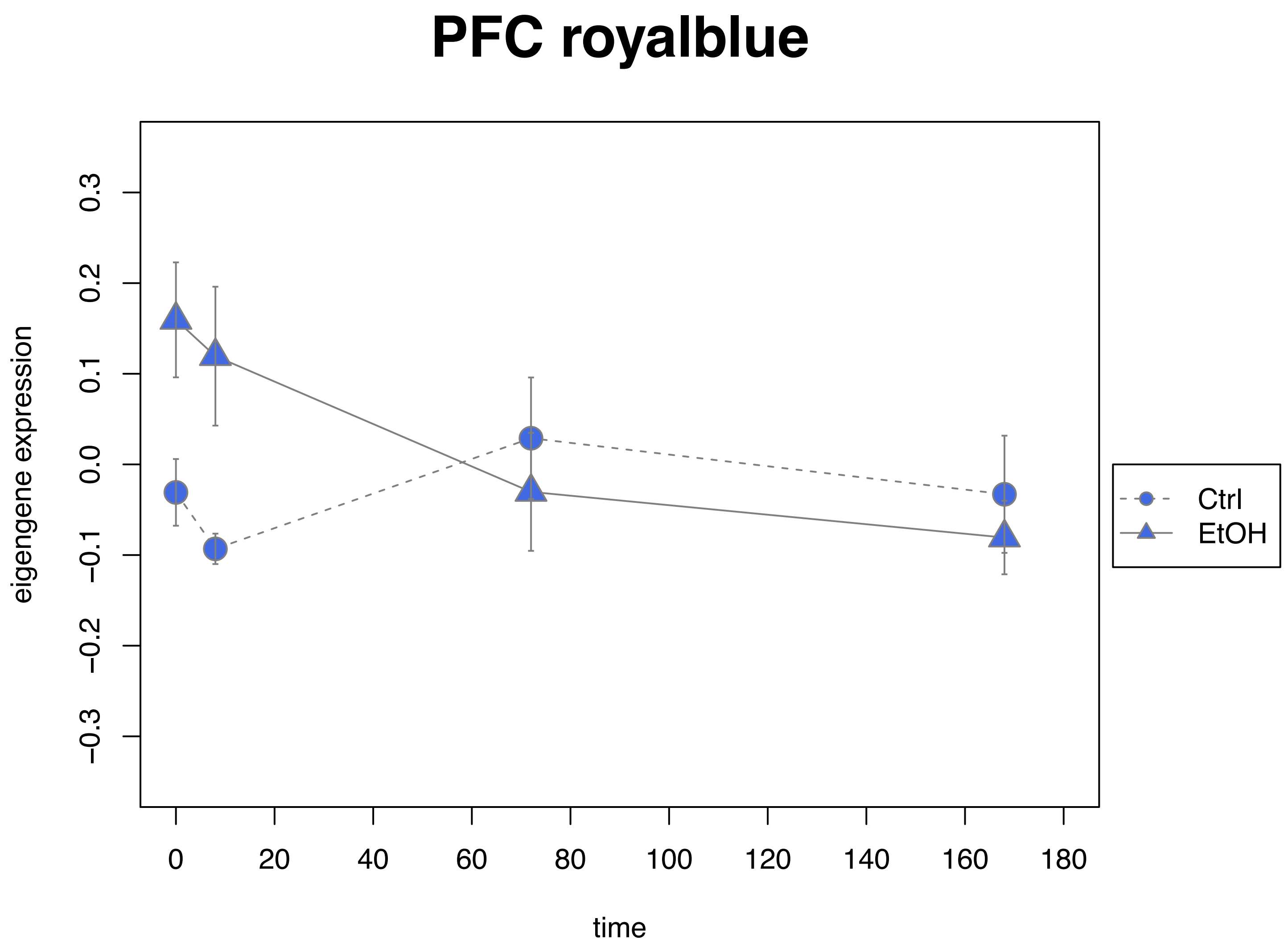

PFC royalblue
eigengene expression
0.3
0.2
0.1
0.0
−0.1
−0.2
−0.3
0
20
40
60
80
100
120
140
160
180
time
Ctrl
EtOH

PFC saddlebrown

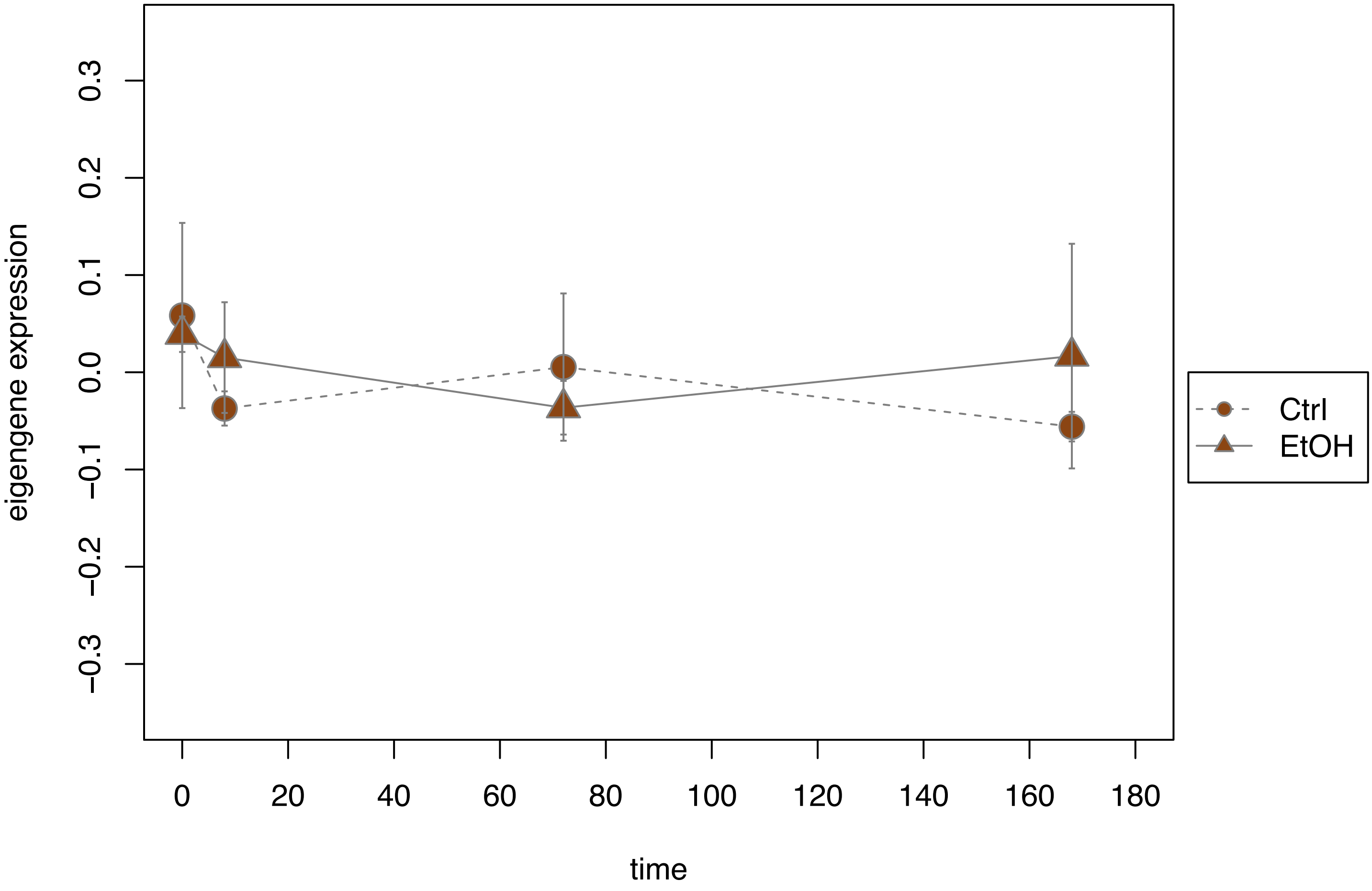

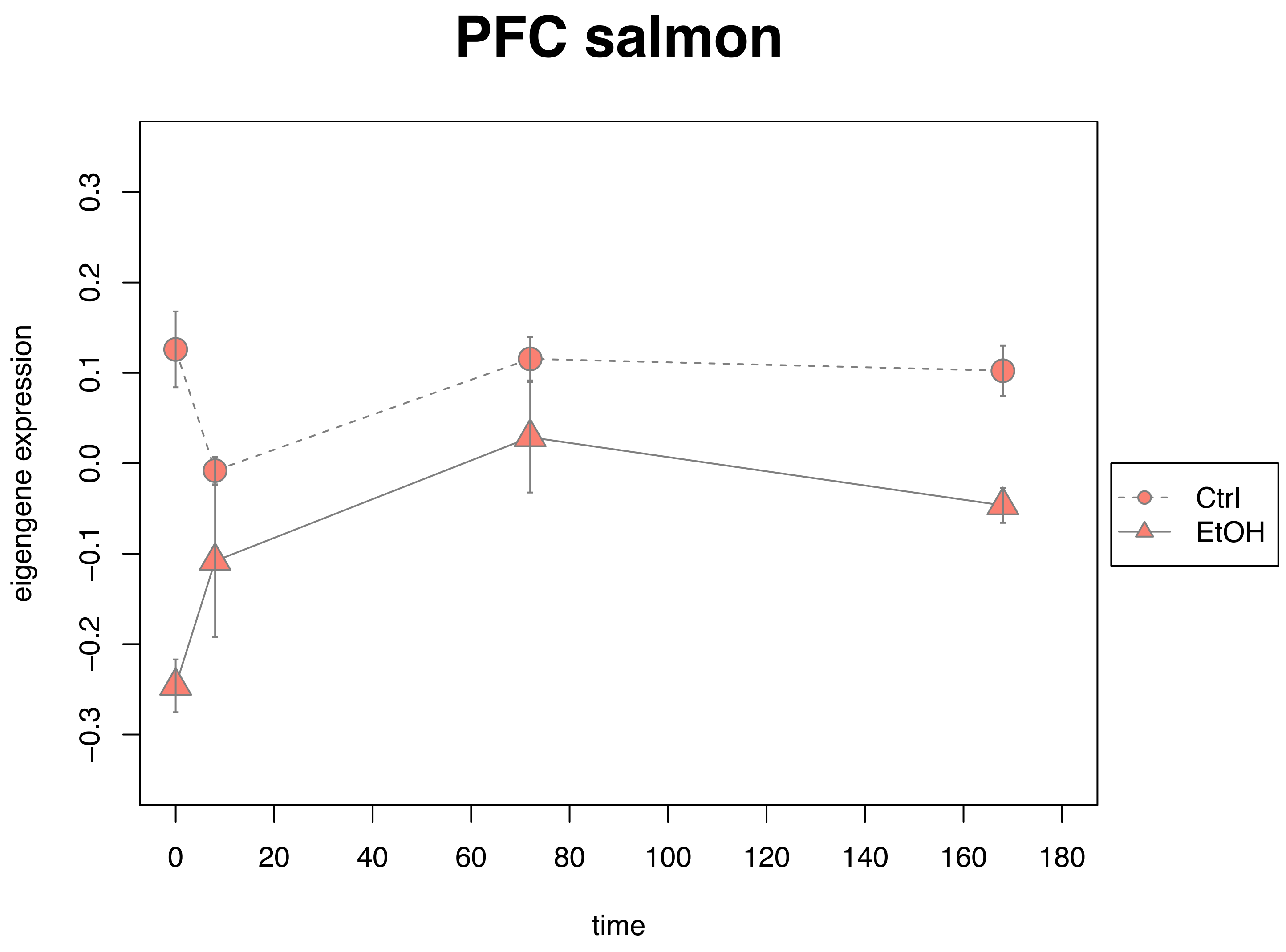
PFC salmon
eigengene expression
0.3
0.2
0.1
0.0
−0.1
−0.2
−0.3
0
20
40
60
80
100
120
140
160
180
time
Ctrl
EtOH

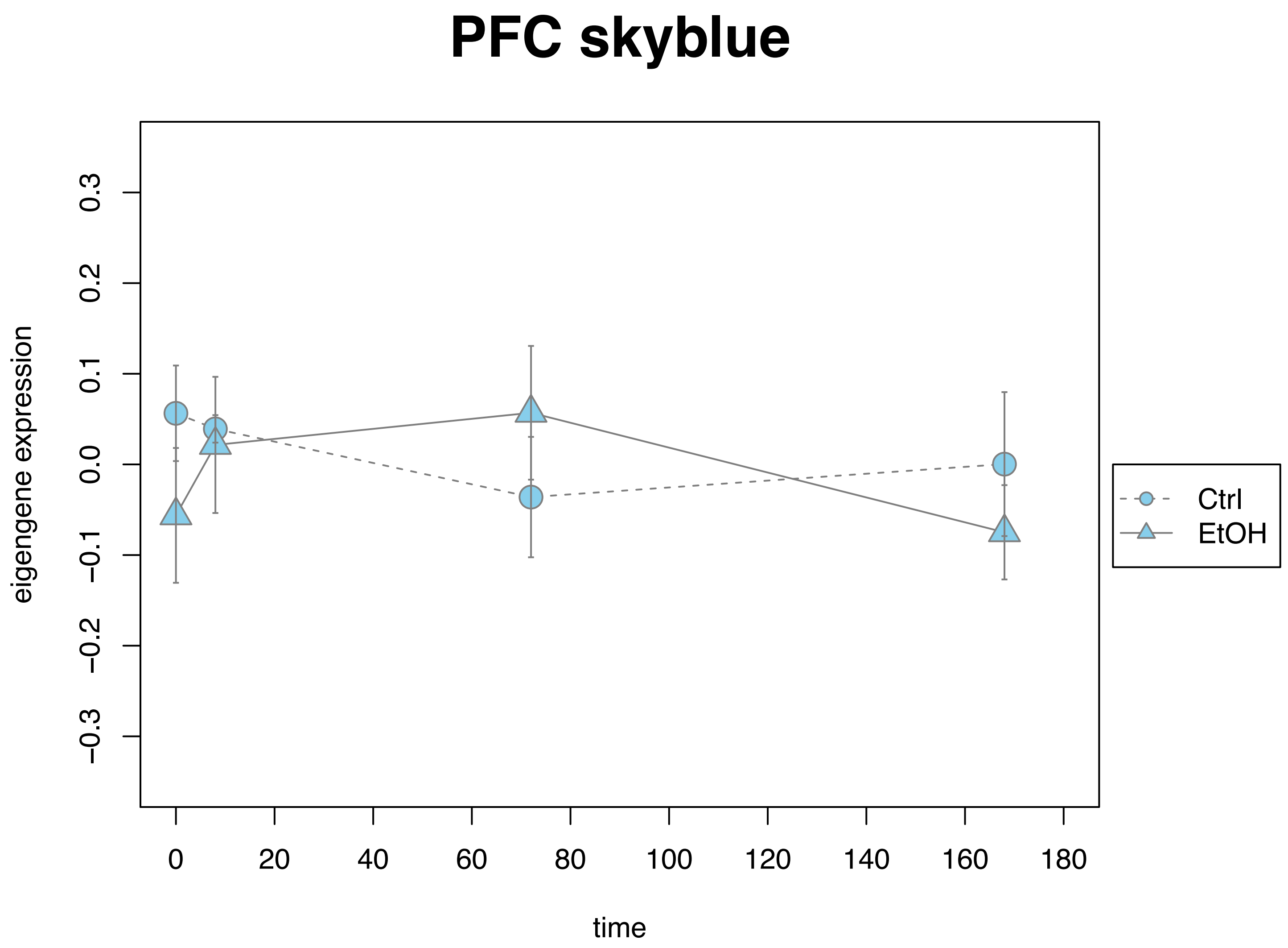

PFC skyblue
eigengene expression
0.3
0.2
0.1
0.0
−0.1
−0.2
−0.3
0
20
40
60
80
100
120
140
160
180
time
Ctrl
EtOH

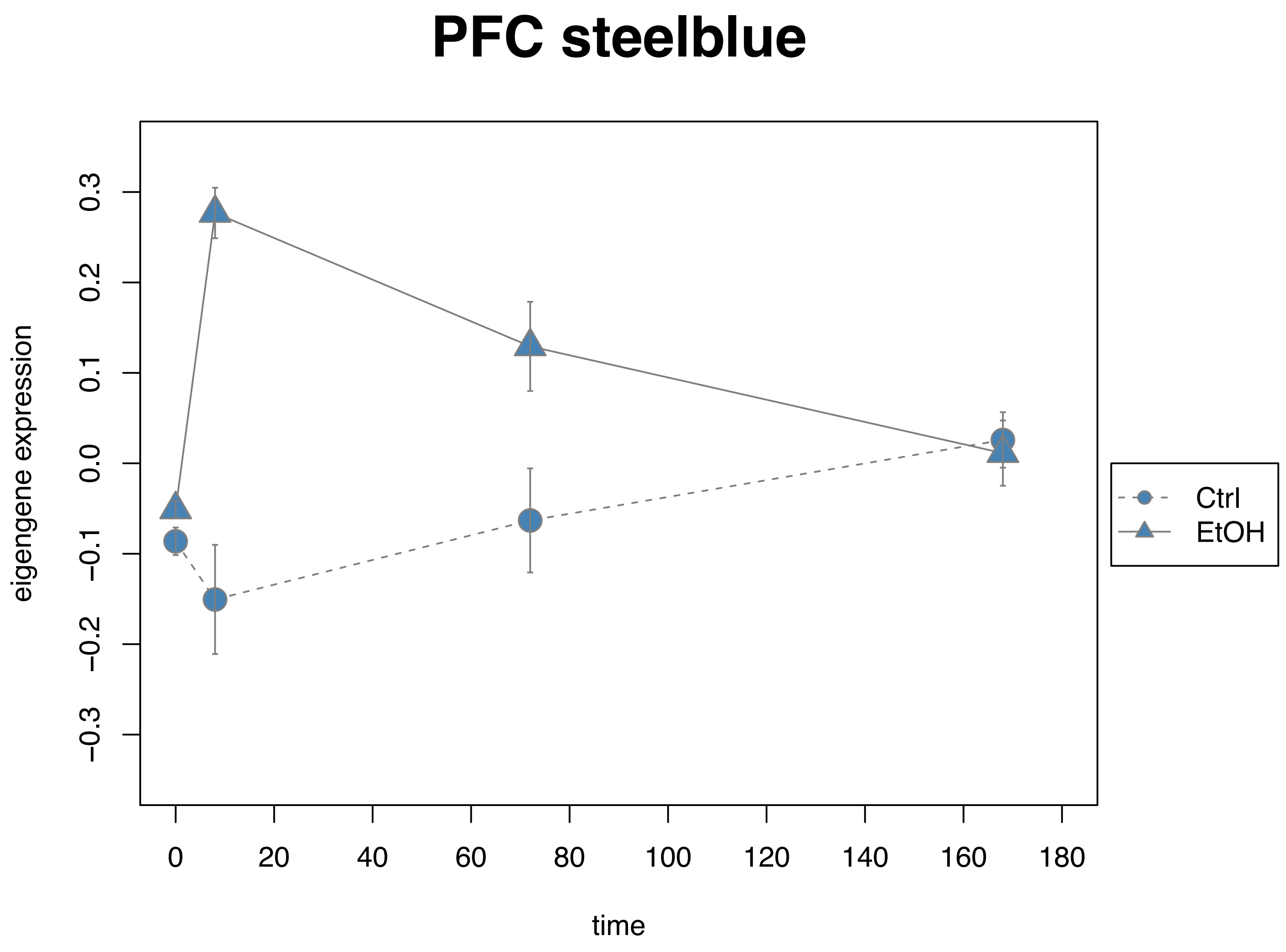
PFC steelblue
eigengene expression
0.3
0.2
0.1
0.0
−0.1
−0.2
−0.3
0
20
40
60
80
100
120
140
160
180
time
Ctrl
EtOH

PFC tan

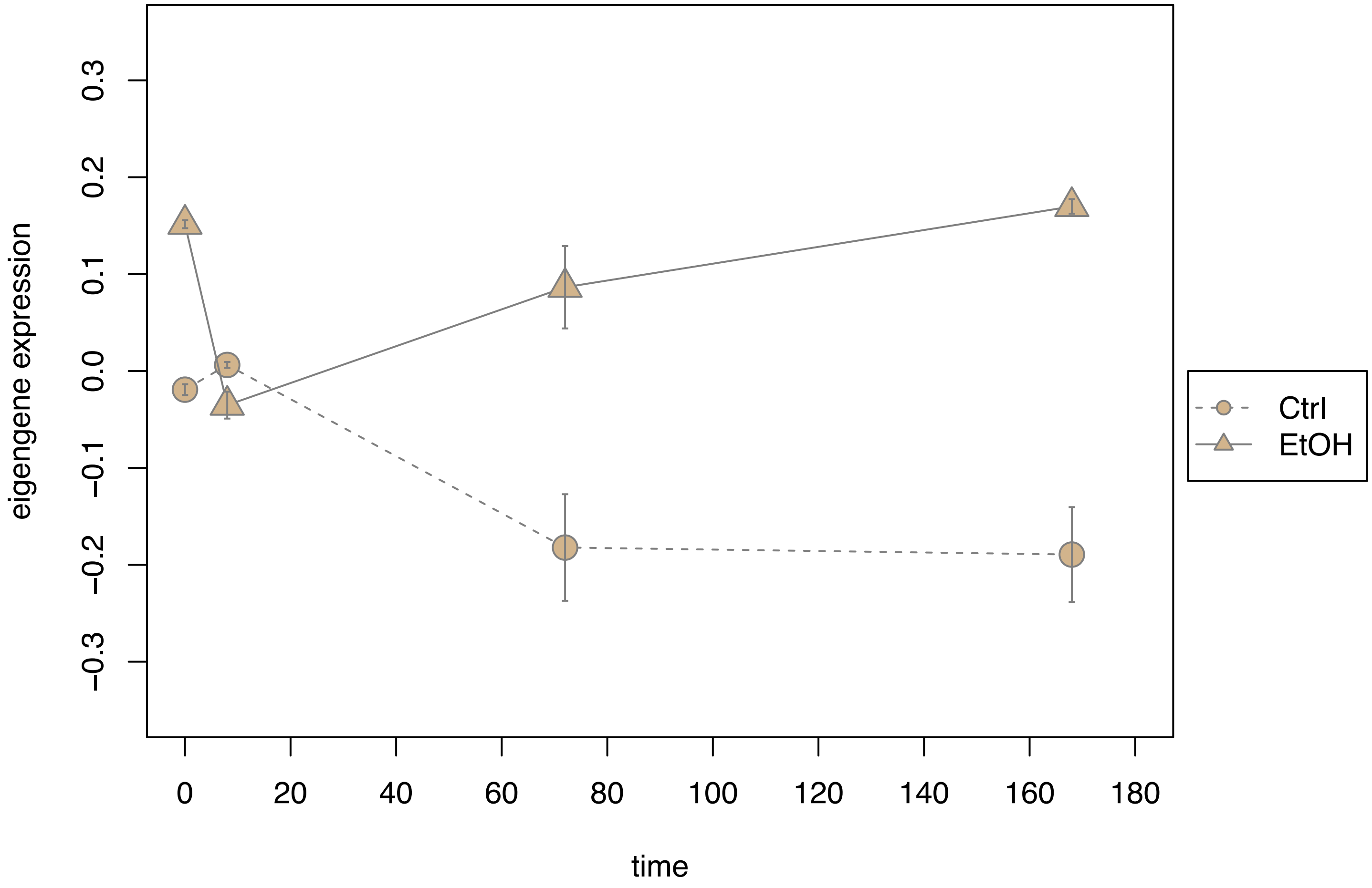

eigengene expression
0.3
0.2
0.1
0.0
−0.1
−0.2
−0.3
0
20
40
60
80
100
120
140
160
180
time
Ctrl
EtOH

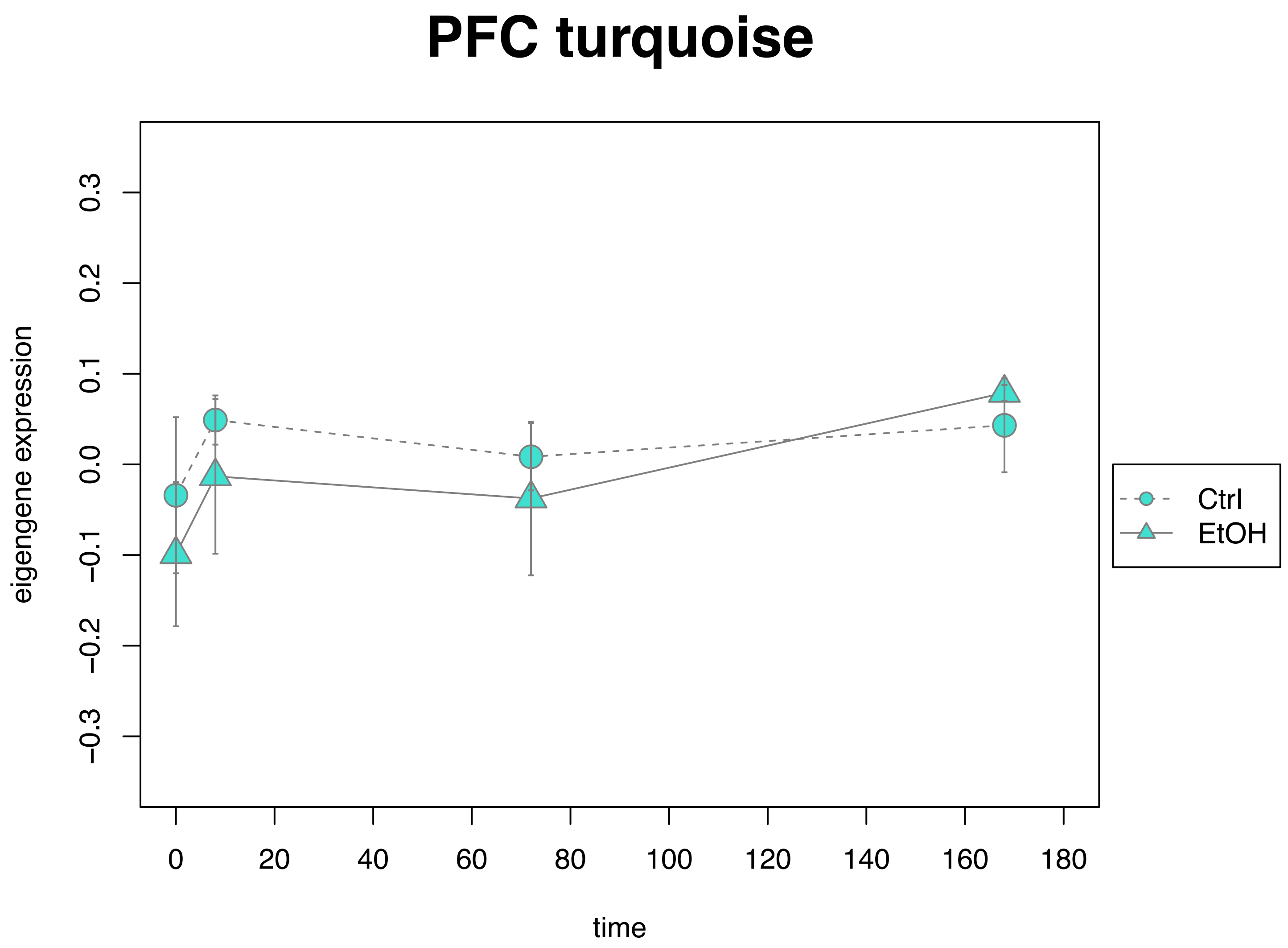
PFC turquoise
eigengene expression
0.3
0.2
0.1
0.0
−0.1
−0.2
−0.3
0
20
40
60
80
100
120
140
160
180
time
Ctrl
EtOH

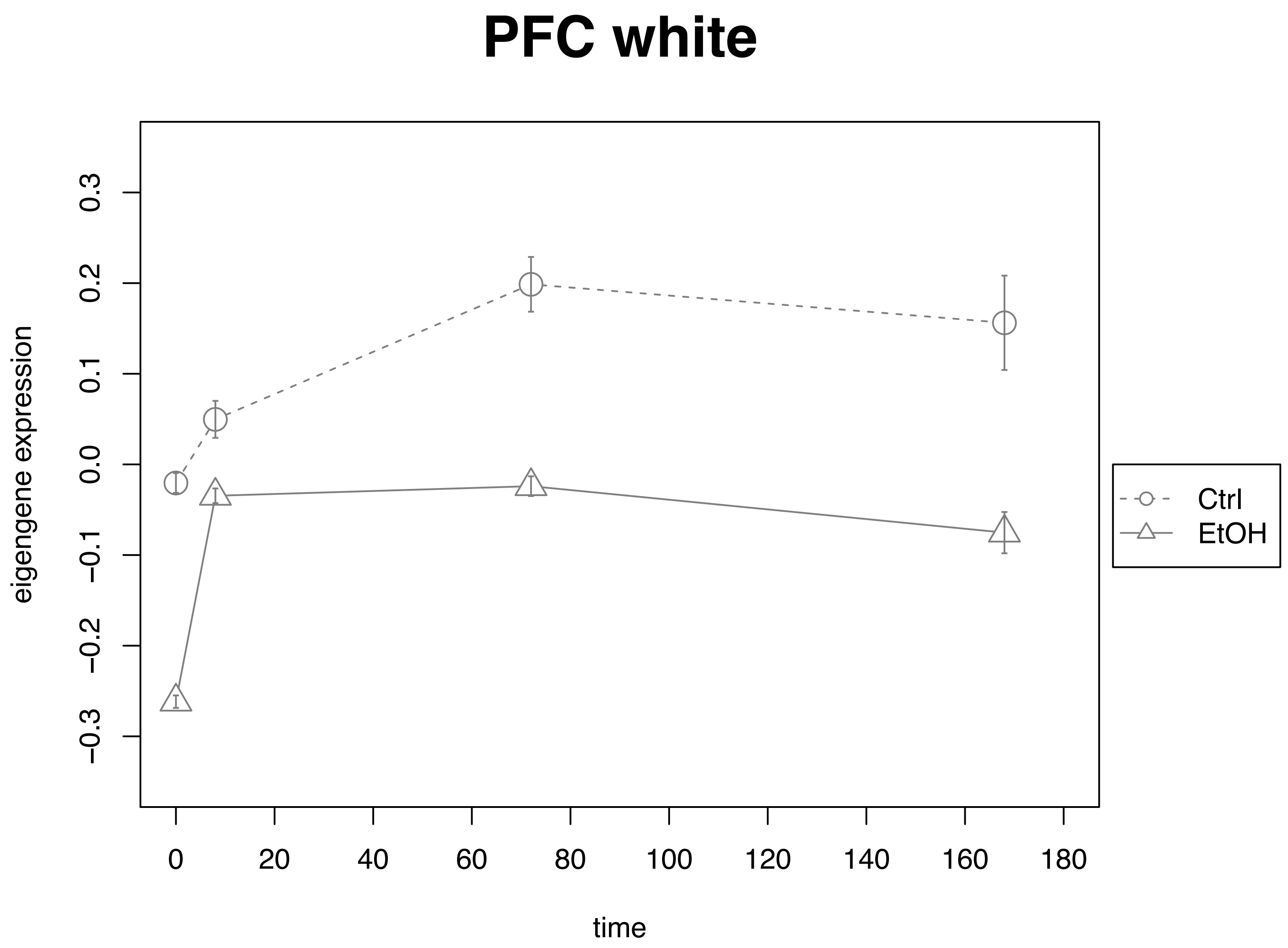

PFC white
eigengene expression
0.3
0.2
0.1
0.0
−0.1
−0.2
−0.3
0
20
40
60
80
100
120
140
160
180
time
Ctrl
EtOH

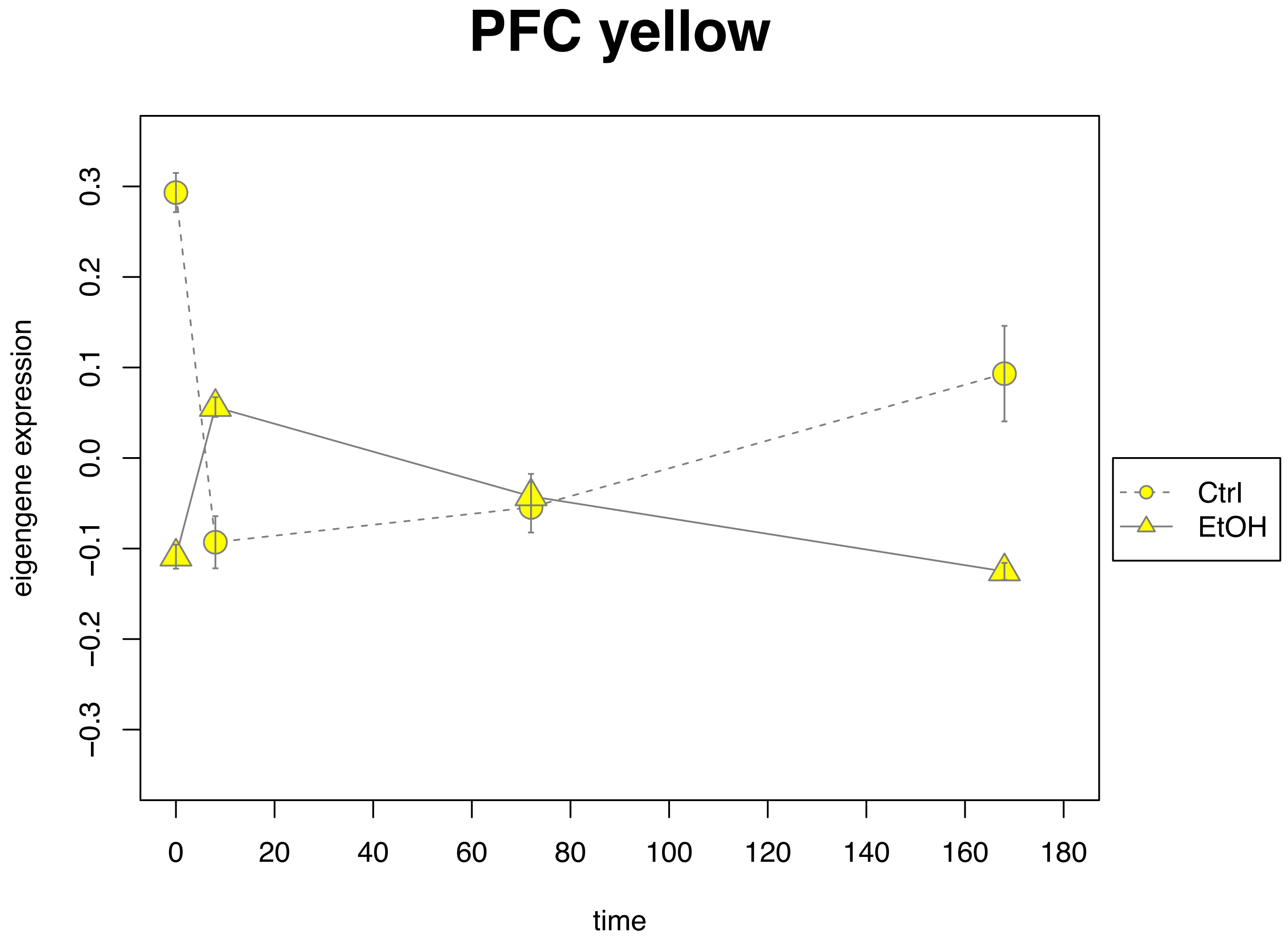
PFC yellow
eigengene expression
0.3
0.2
0.1
0.0
−0.1
−0.2
−0.3
0
20
40
60
80
100
120
140
160
180
time
Ctrl
EtOH
