# Supplementary material for: Time-Course Analysis of Brain Regional Expression Network Responses to Chronic Intermittent Ethanol and Withdrawal: Implications for Mechanisms Underlying Excessive Ethanol Consumption
Source: PLoS One. 2016 Jan 5;11(1):e0146257. doi: 10.1371/journal.pone.0146257 (PMC4701666; doi:10.1371/journal.pone.0146257)
Supplement: S2 Fig — (PDF) [file pone.0146257.s002.pdf]

B6Exp1 NAC WGCNA-DS2 Multidimensional Scaling

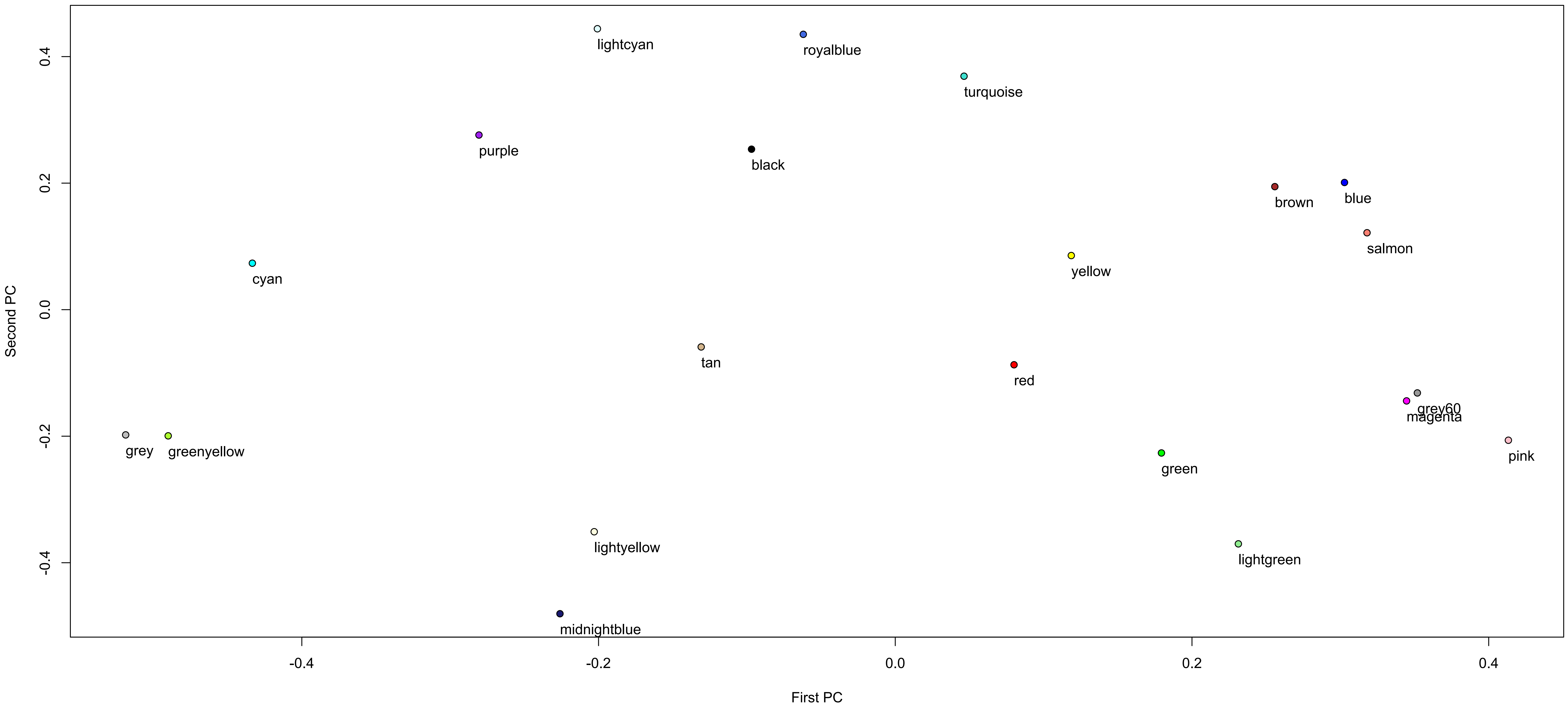

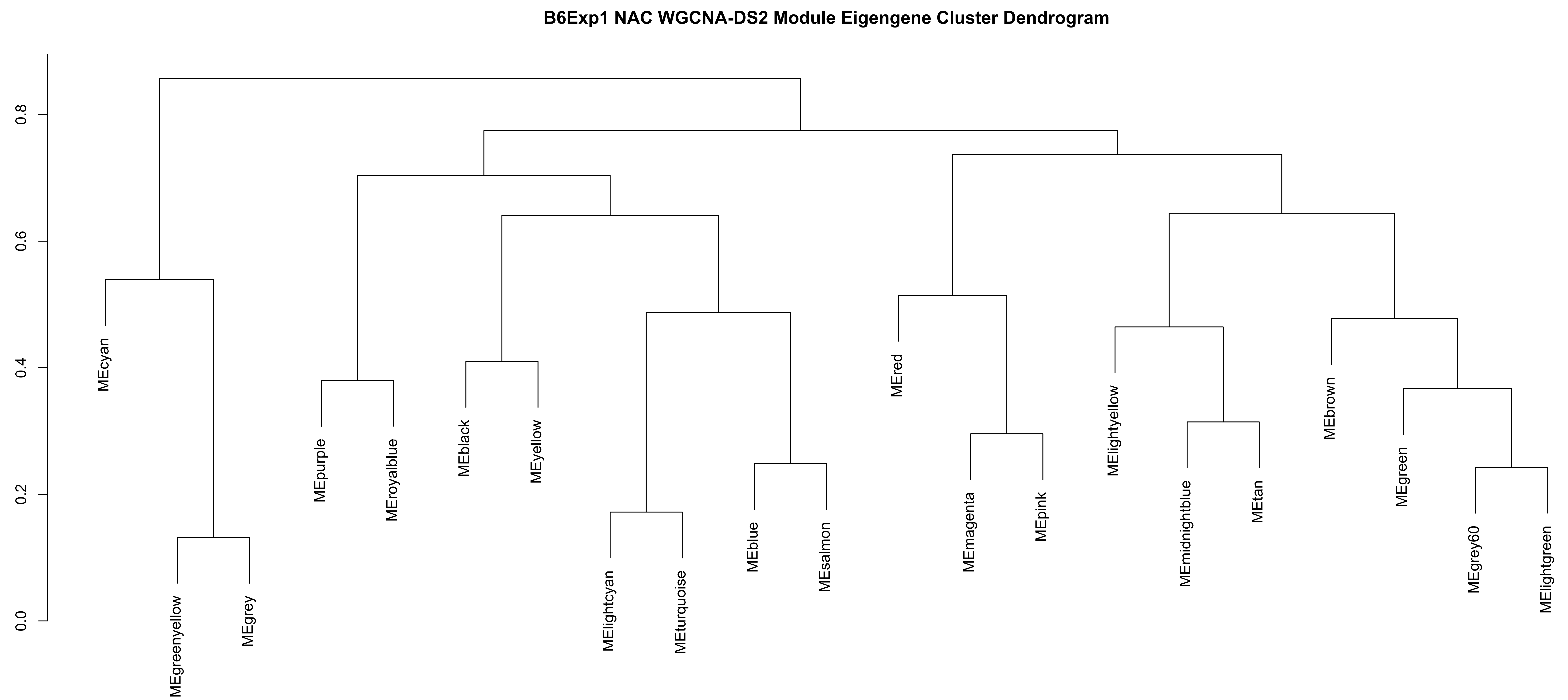

B6Exp1 NAC WGCNA-DS2 Module Eigengene Cluster Dendrogram
0.0
0.2
0.4
0.6
0.8
MEcyan
MEgreenyellow
MEgrey
MEpurple
MEroyalblue
MEblack
MEyellow
MElightcyan
MEturquoise
MEblue
MEsalmon
MEred
MEmagenta
MEpink
MElightyellow
MEmidnightblue
MEtan
MEbrown
MEgreen
MEgrey60
MElightgreen

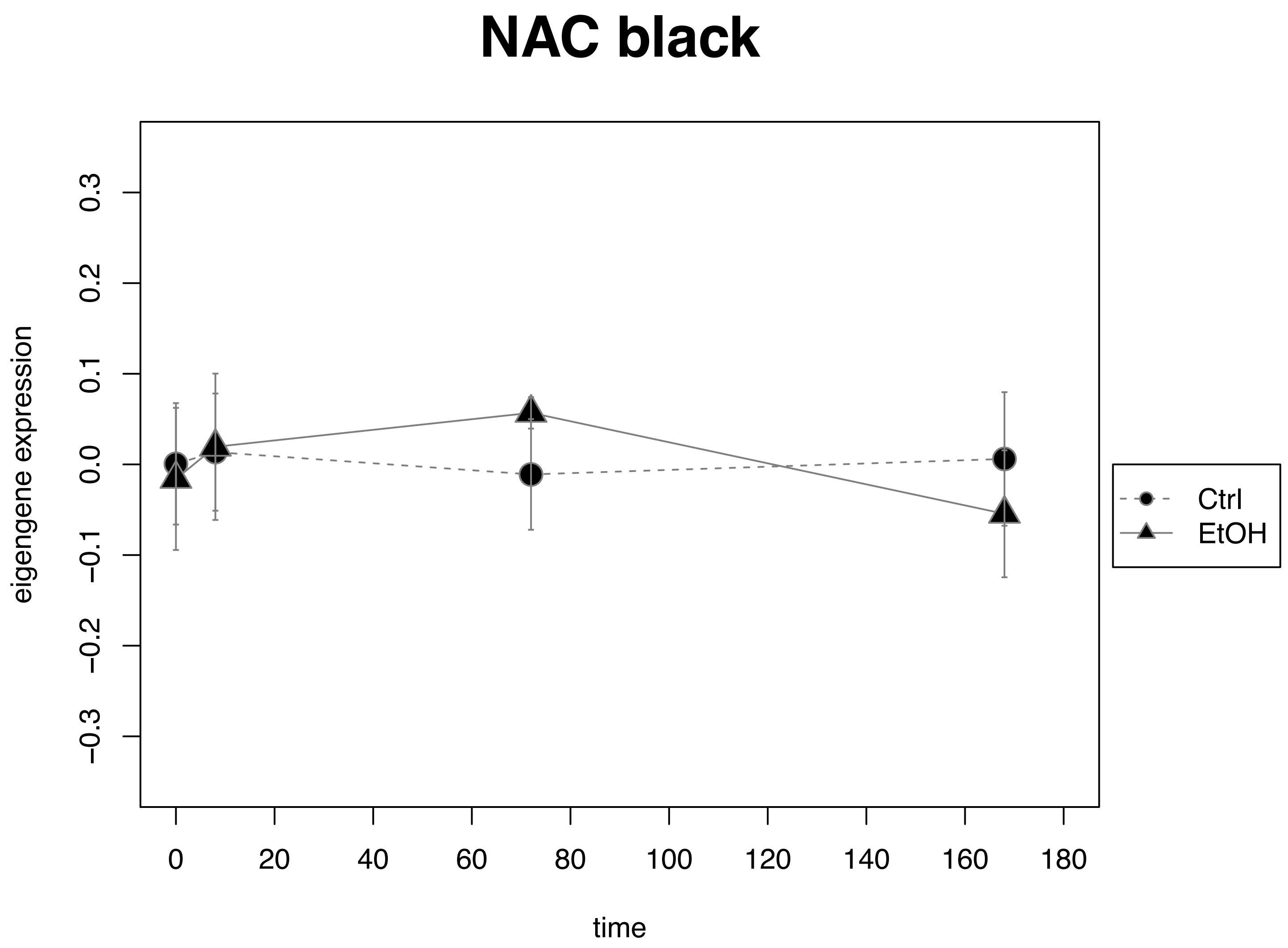

NAC black
eigengene expression
0.3
0.2
0.1
0.0
−0.1
−0.2
−0.3
0
20
40
60
80
100
120
140
160
180
time
Ctrl
EtOH

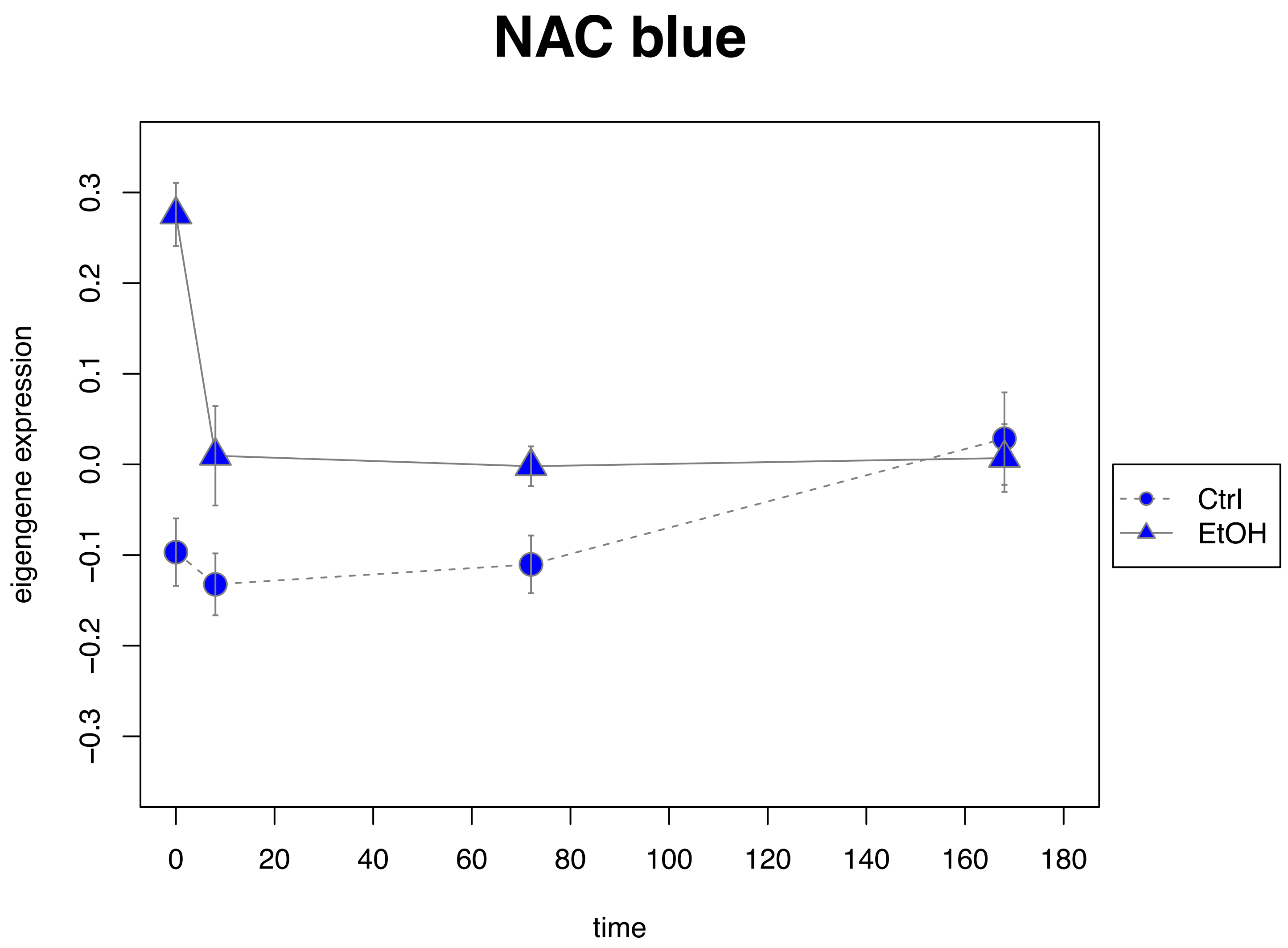

NAC blue
eigengene expression
time
0.3
0.2
0.1
0.0
−0.1
−0.2
−0.3
0
20
40
60
80
100
120
140
160
180
Ctrl
EtOH

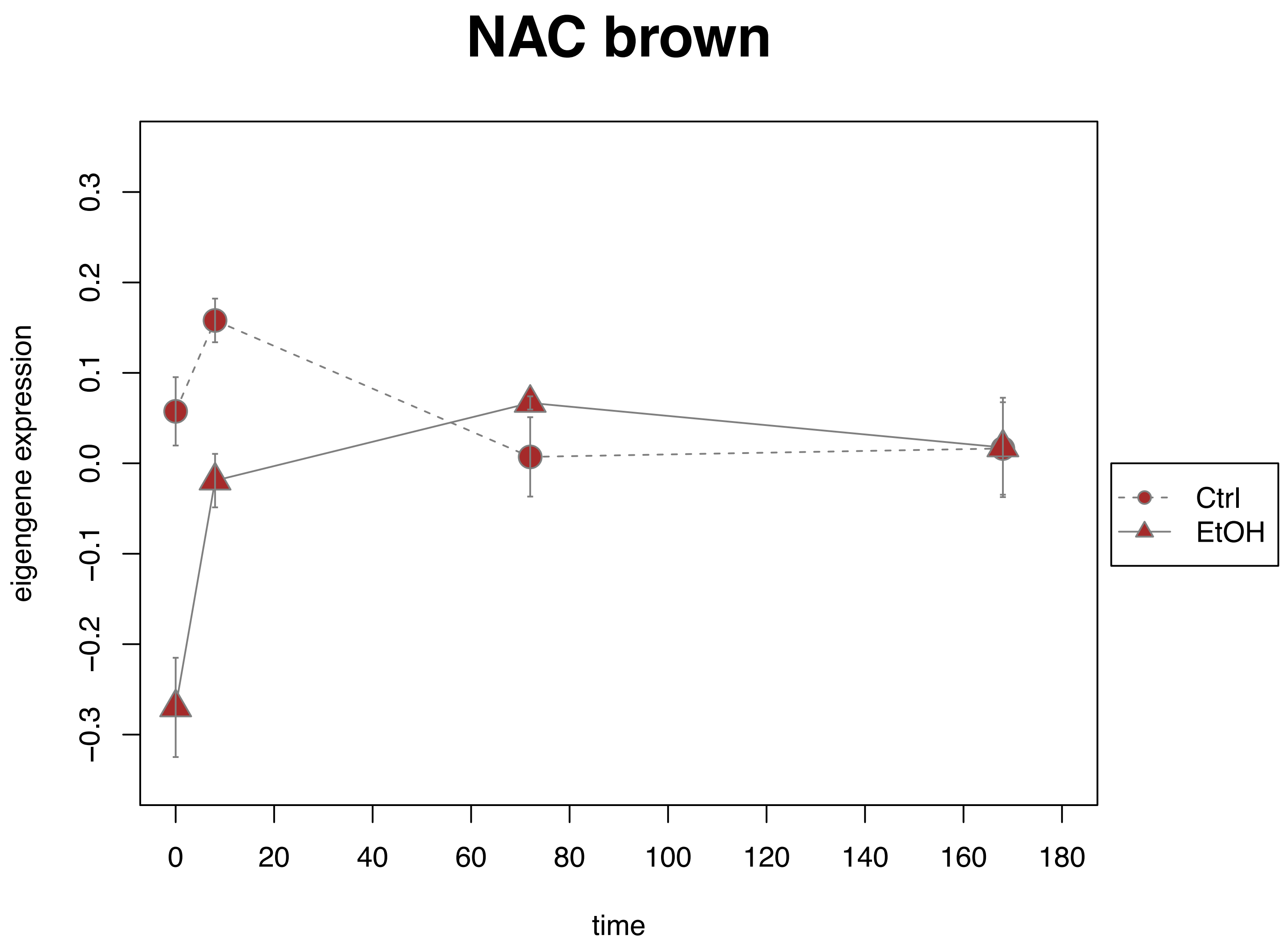
NAC brown
eigengene expression
0.3
0.2
0.1
0.0
−0.1
−0.2
−0.3
0
20
40
60
80
100
120
140
160
180
time
Ctrl
EtOH

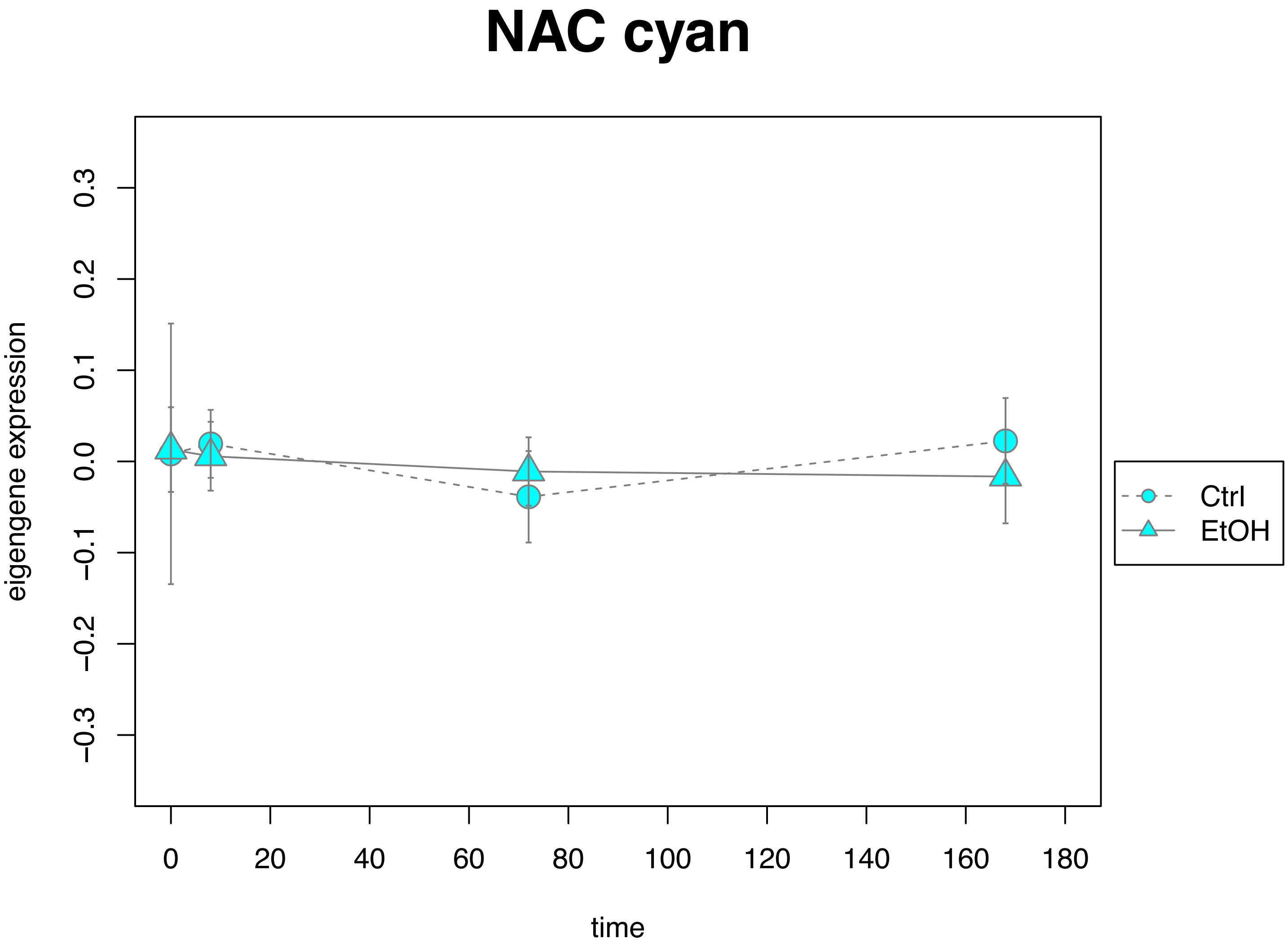

NAC cyan
eigengene expression
0.3
0.2
0.1
0.0
−0.1
−0.2
−0.3
0
20
40
60
80
100
120
140
160
180
time
Ctrl
EtOH

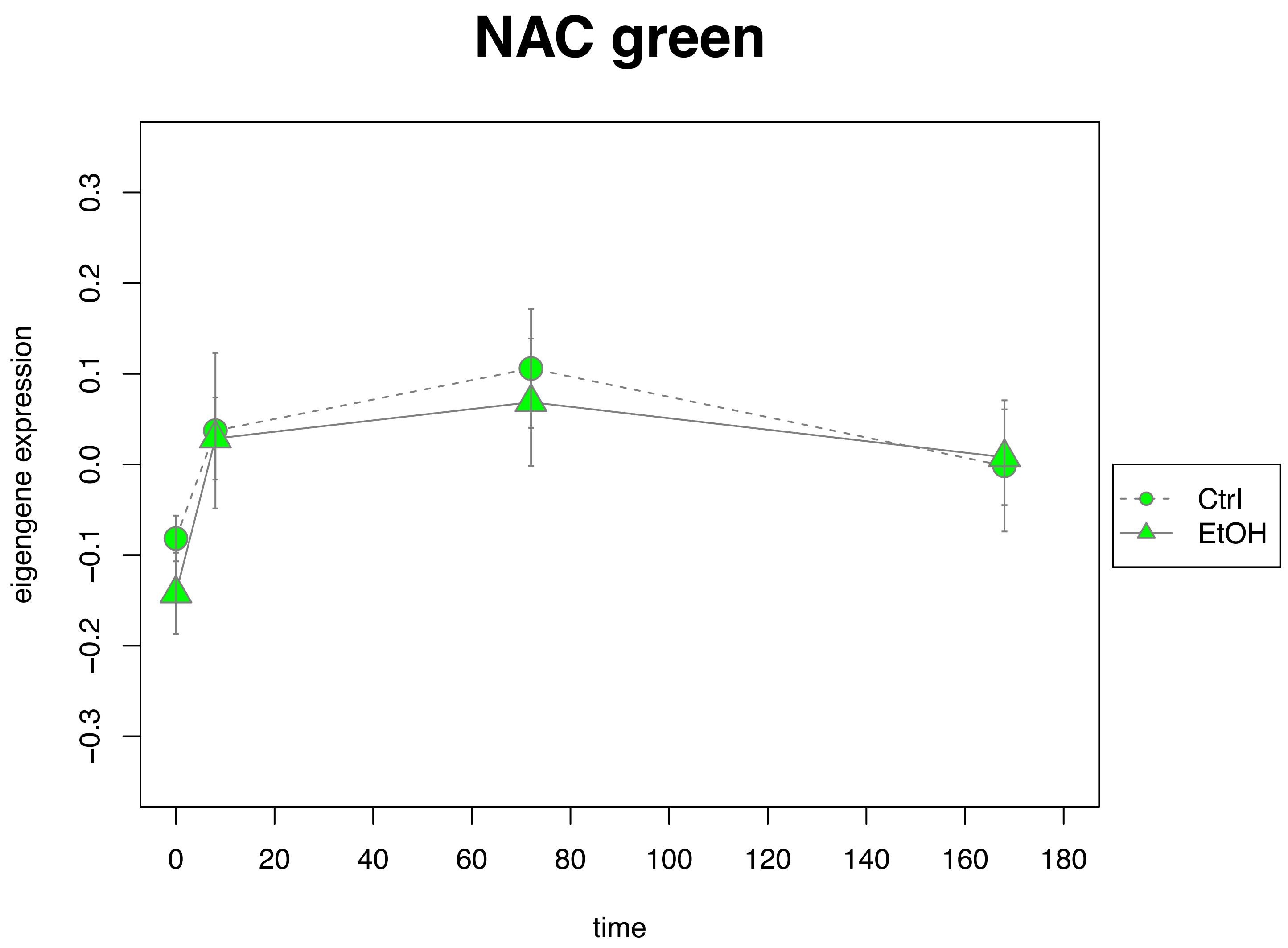
NAC green
eigengene expression
0.3
0.2
0.1
0.0
−0.1
−0.2
−0.3
0
20
40
60
80
100
120
140
160
180
time
Ctrl
EtOH

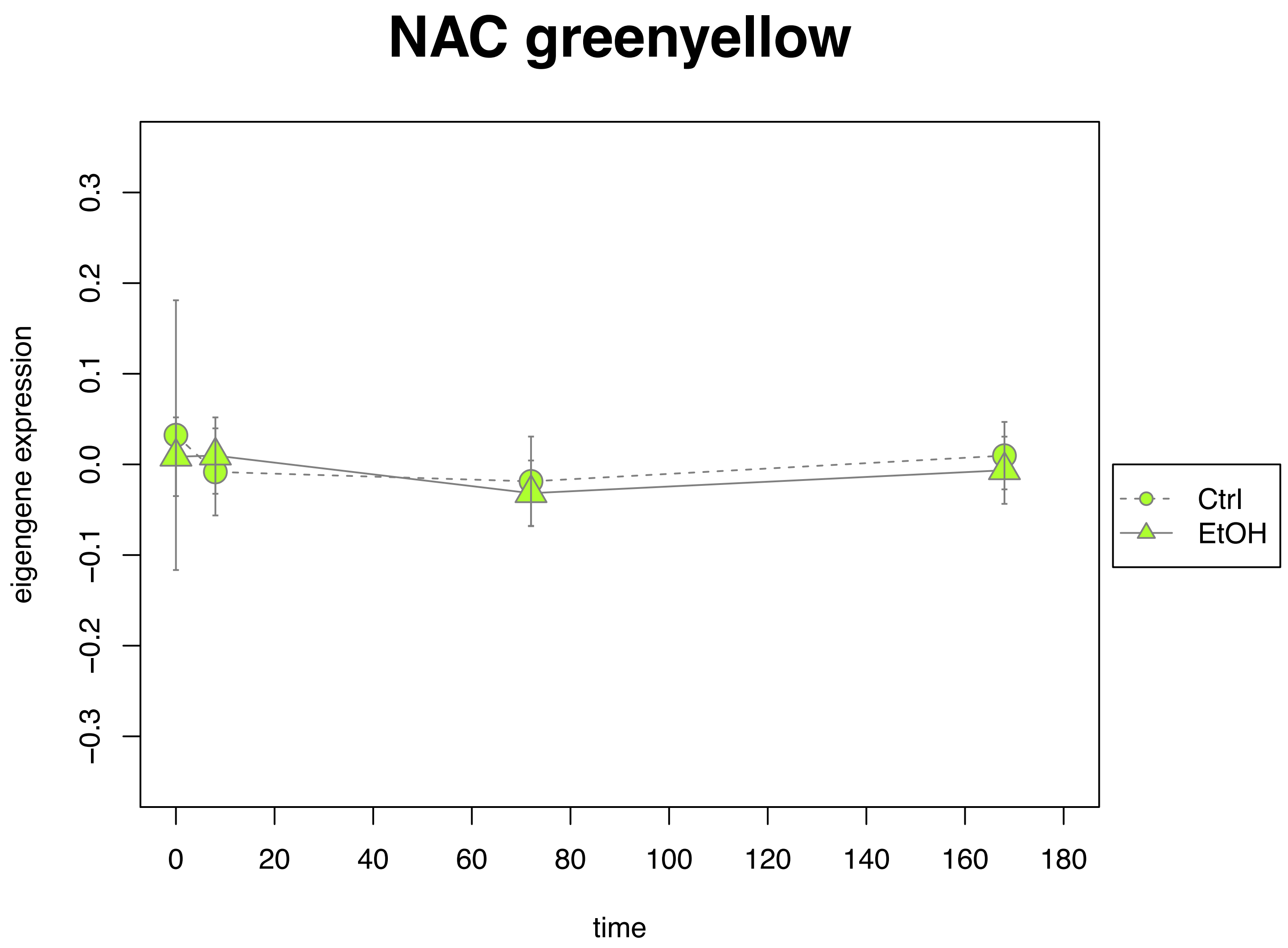

NAC greenyellow
eigengene expression
time
0.3
0.2
0.1
0.0
−0.1
−0.2
−0.3
0
20
40
60
80
100
120
140
160
180
Ctrl
EtOH

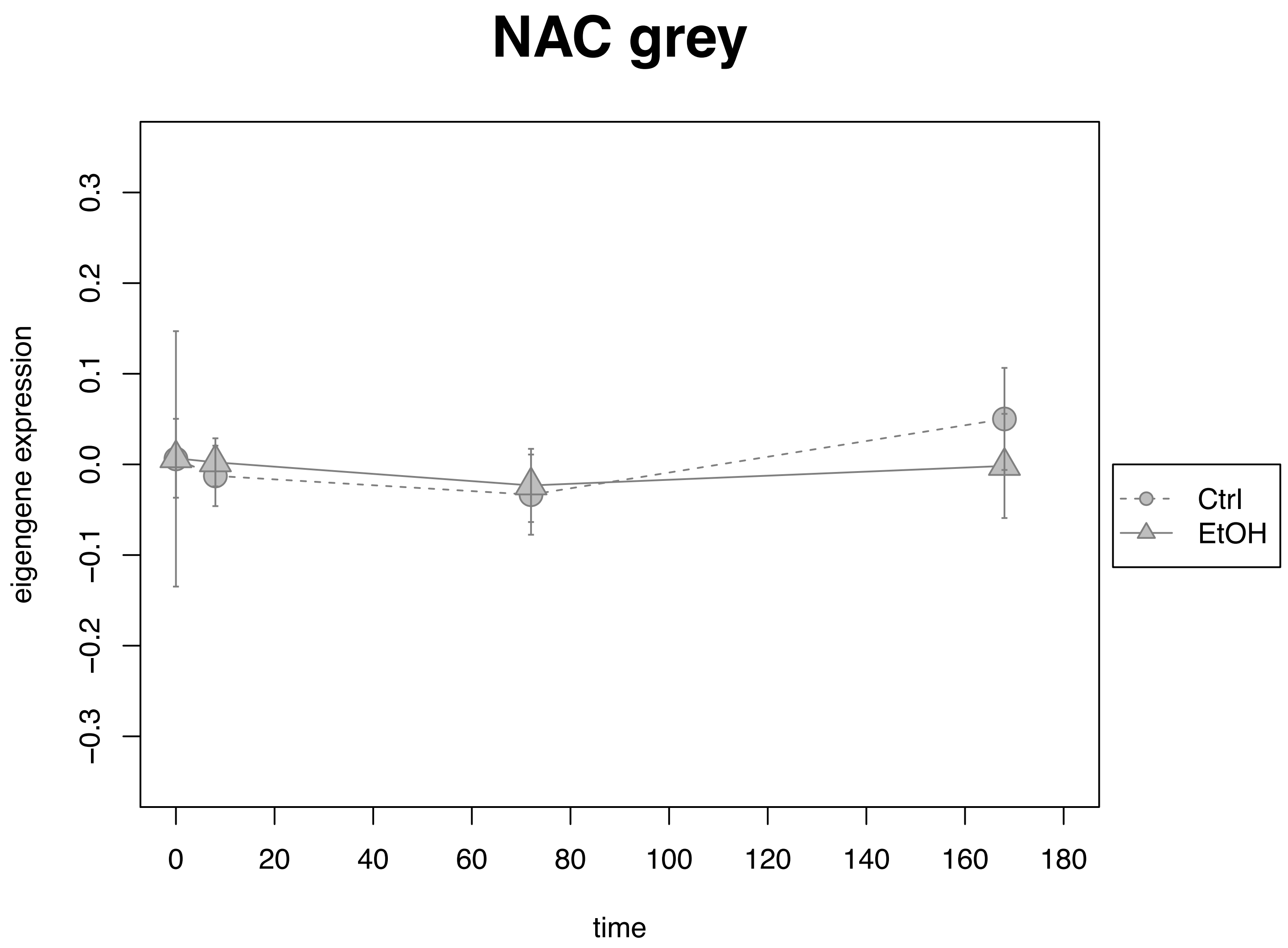

NAC grey
eigengene expression
0.3
0.2
0.1
0.0
−0.1
−0.2
−0.3
0
20
40
60
80
100
120
140
160
180
time
Ctrl
EtOH

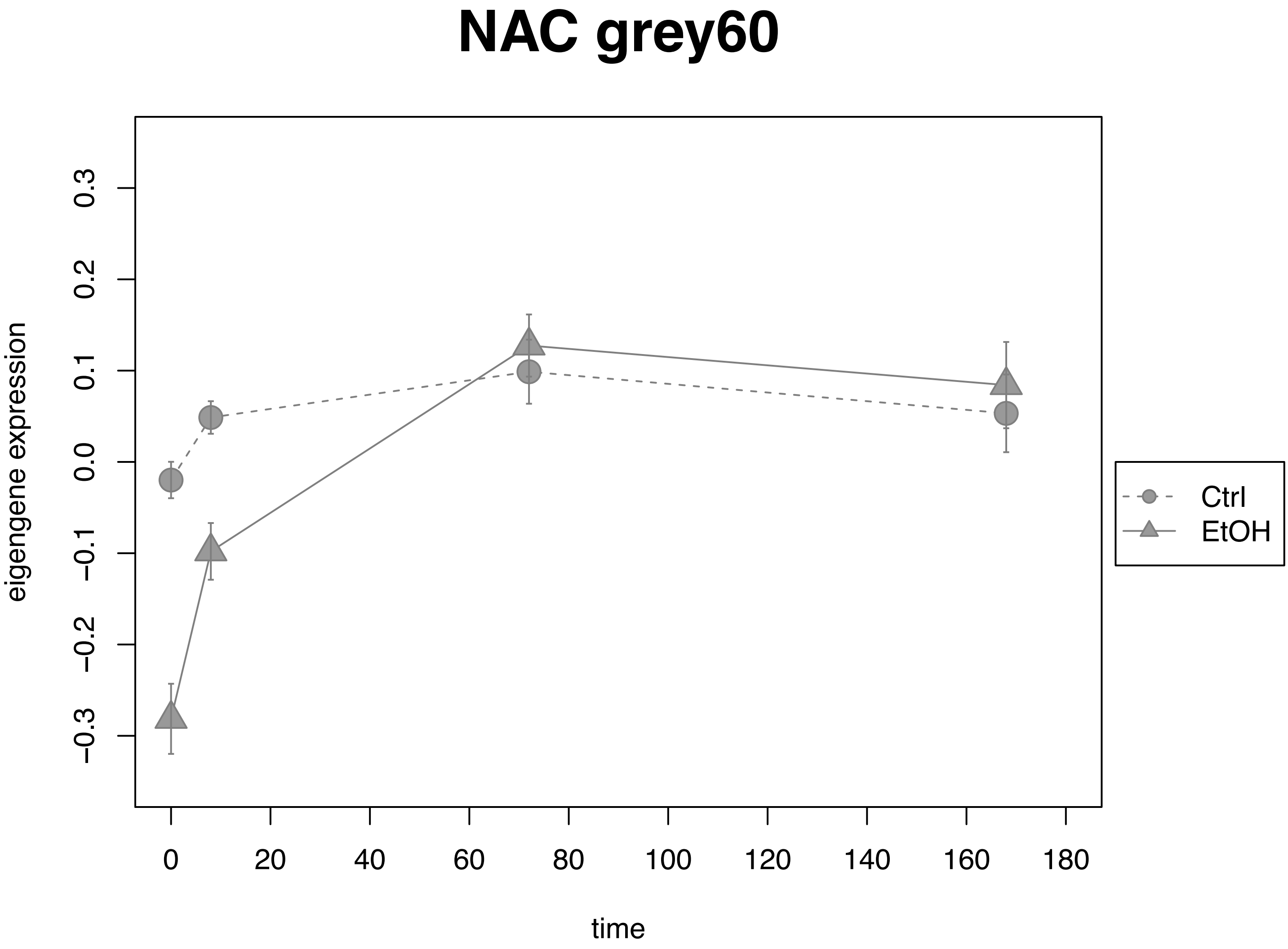
NAC grey60
eigengene expression
time
0.3
0.2
0.1
0.0
−0.1
−0.2
−0.3
0
20
40
60
80
100
120
140
160
180
Ctrl
EtOH

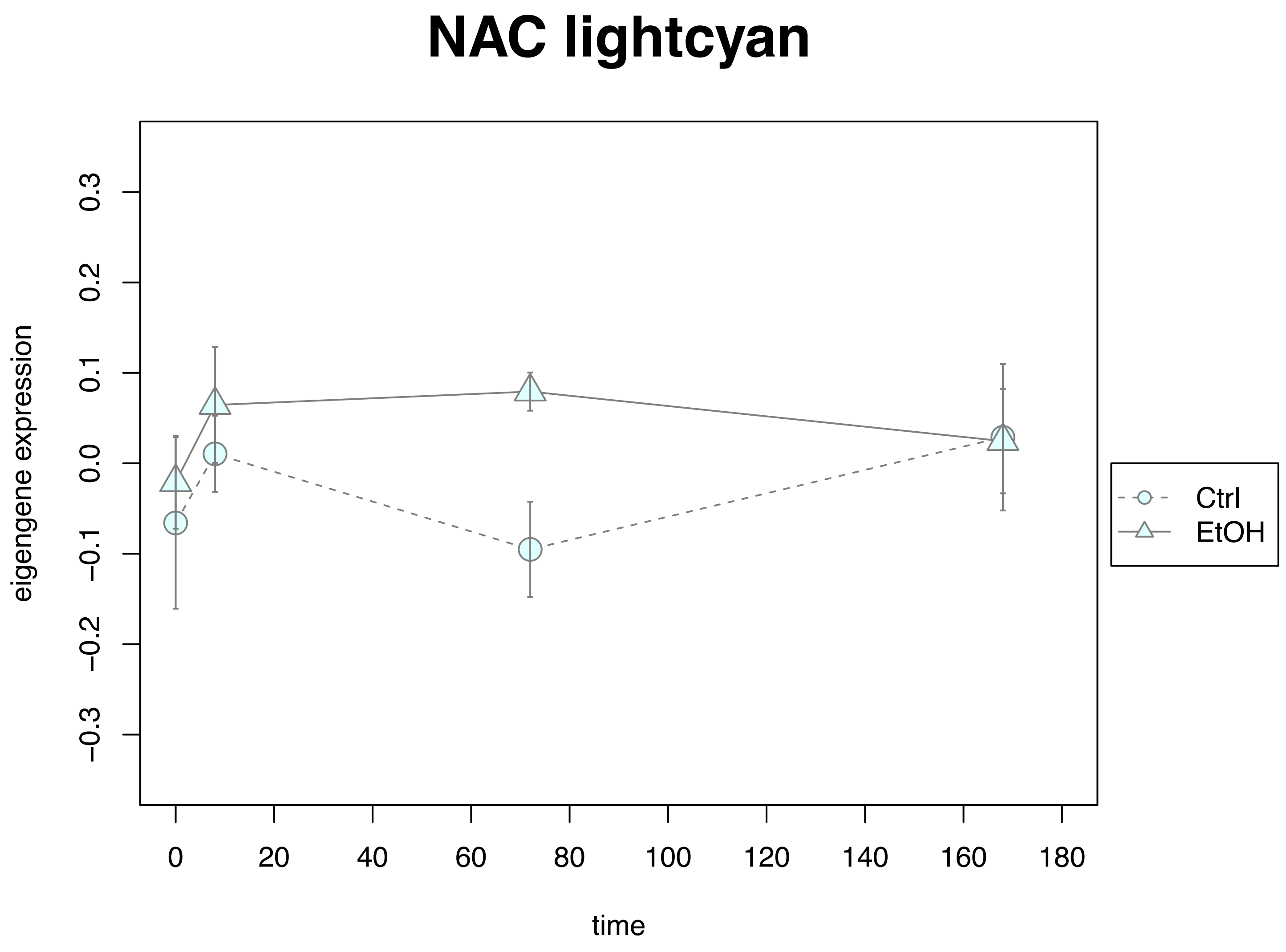
NAC lightcyan
eigengene expression
time
0.3
0.2
0.1
0.0
−0.1
−0.2
−0.3
0
20
40
60
80
100
120
140
160
180
Ctrl
EtOH

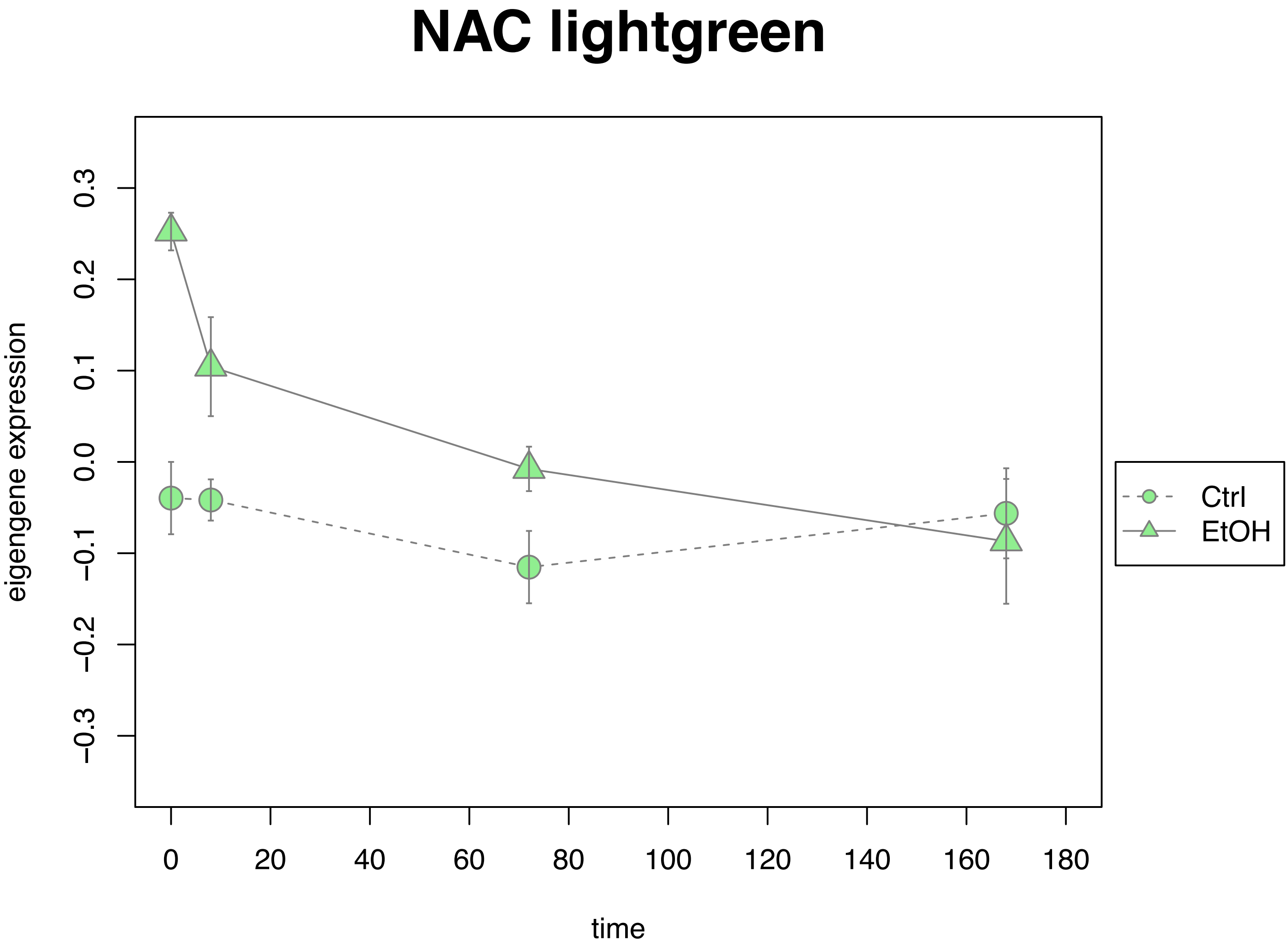
NAC lightgreen
eigengene expression
0.3
0.2
0.1
0.0
−0.1
−0.2
−0.3
0
20
40
60
80
100
120
140
160
180
time
Ctrl
EtOH

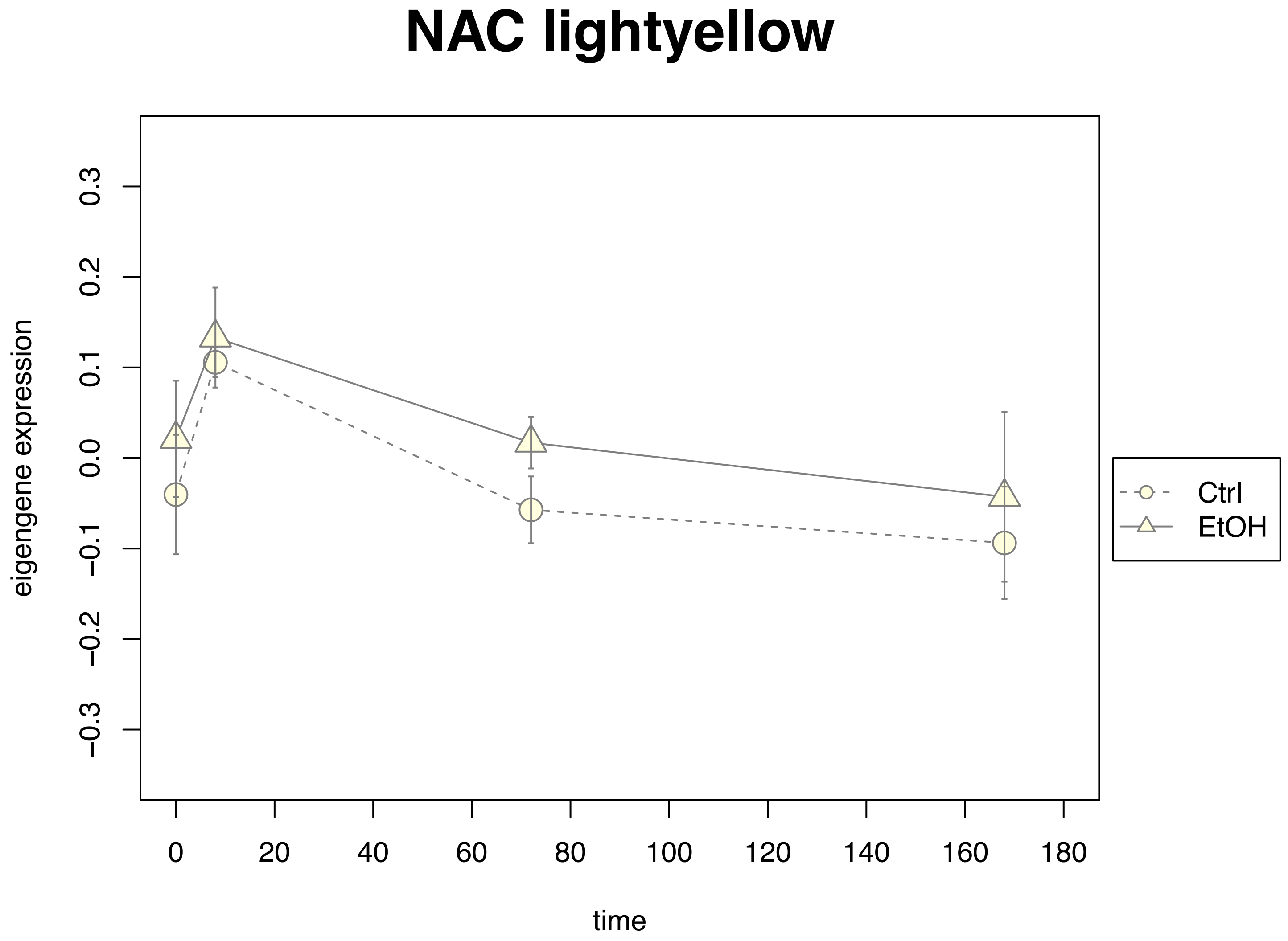
NAC lightyellow
eigengene expression
0.3
0.2
0.1
0.0
−0.1
−0.2
−0.3
0
20
40
60
80
100
120
140
160
180
time
Ctrl
EtOH

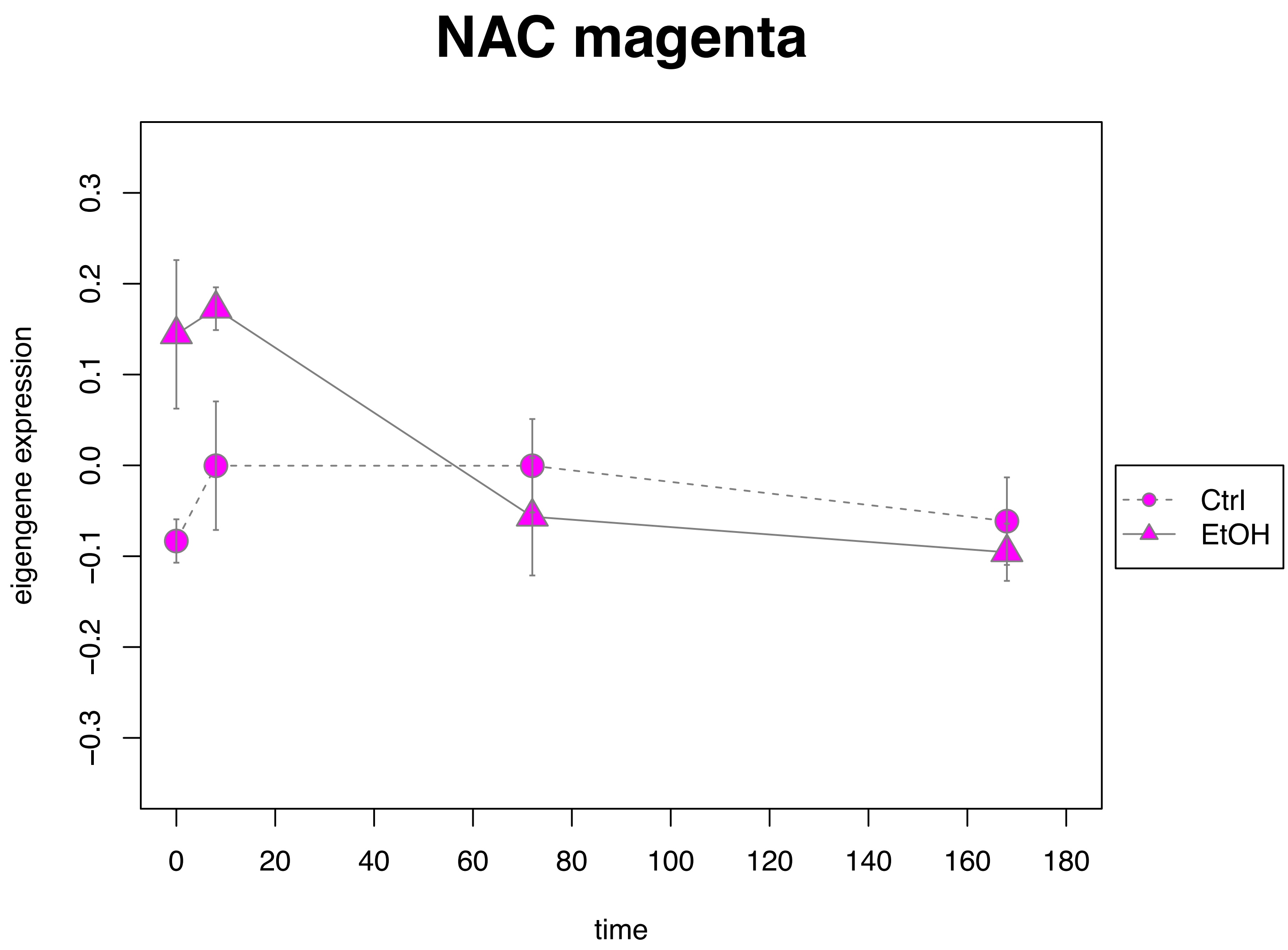
NAC magenta
eigengene expression
0.3
0.2
0.1
0.0
−0.1
−0.2
−0.3
0
20
40
60
80
100
120
140
160
180
time
Ctrl
EtOH

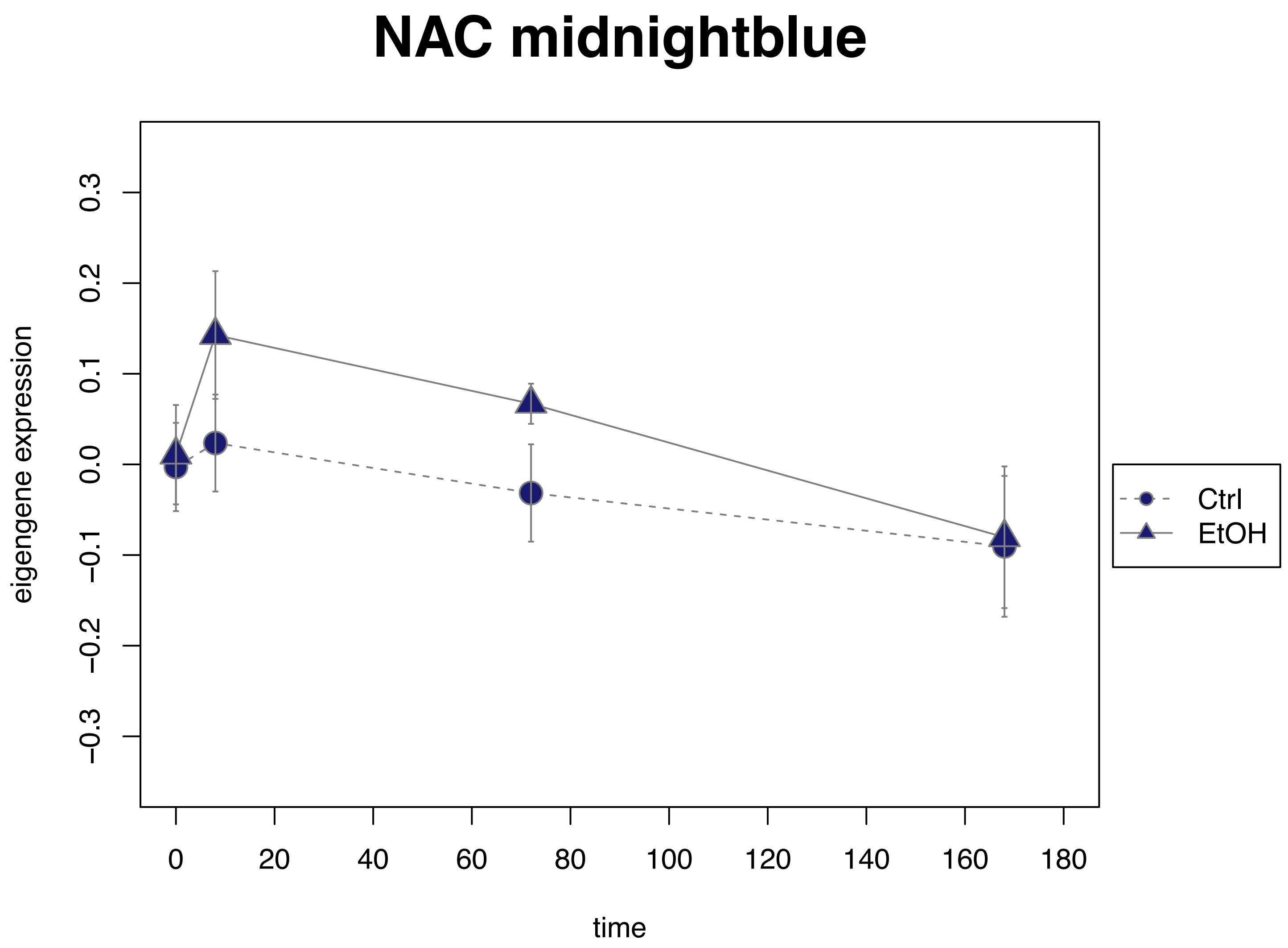
NAC midnightblue
eigengene expression
0.3
0.2
0.1
0.0
−0.1
−0.2
−0.3
0
20
40
60
80
100
120
140
160
180
time
Ctrl
EtOH

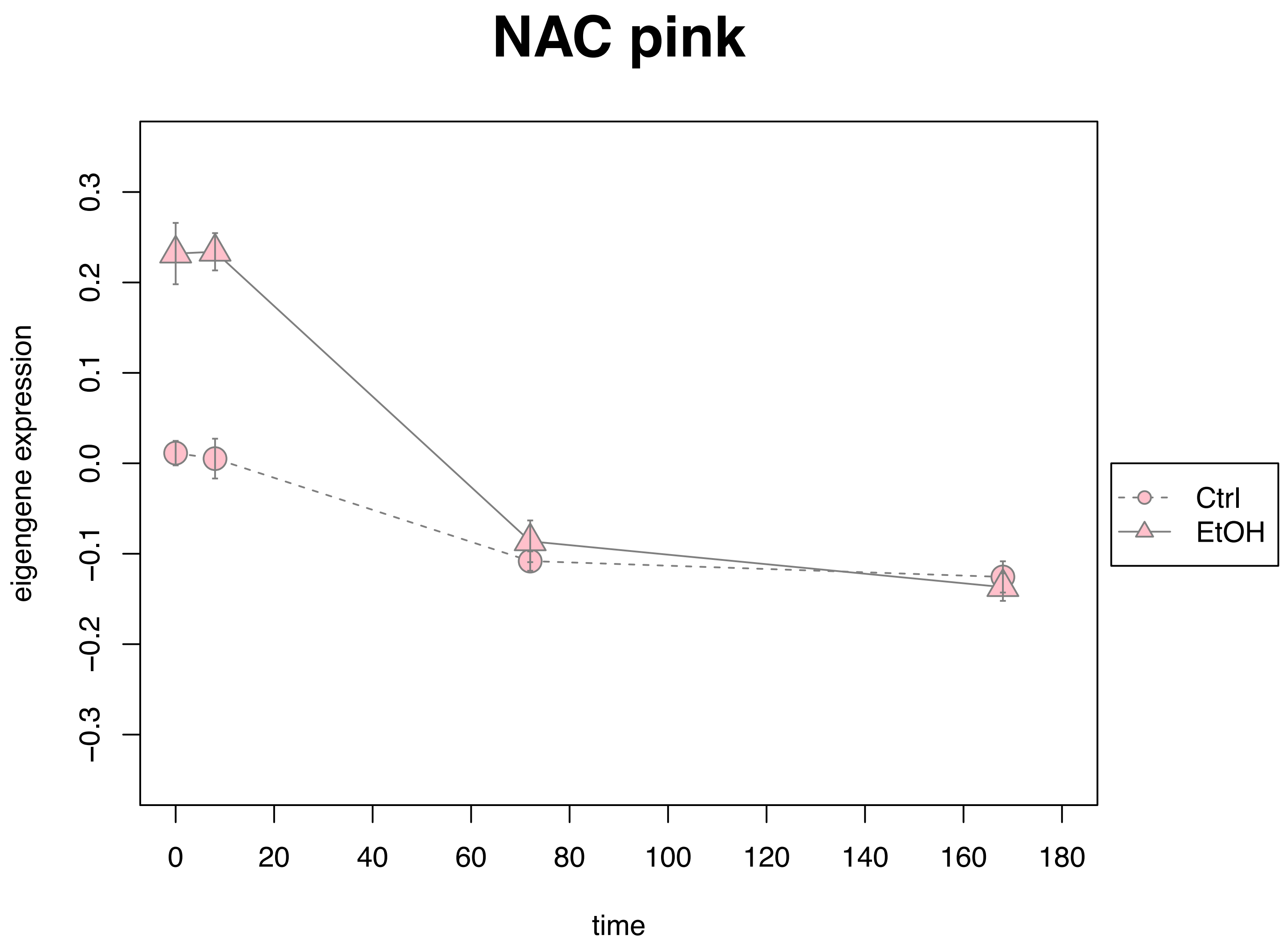
NAC pink
eigengene expression
0.3
0.2
0.1
0.0
−0.1
−0.2
−0.3
0
20
40
60
80
100
120
140
160
180
time
Ctrl
EtOH

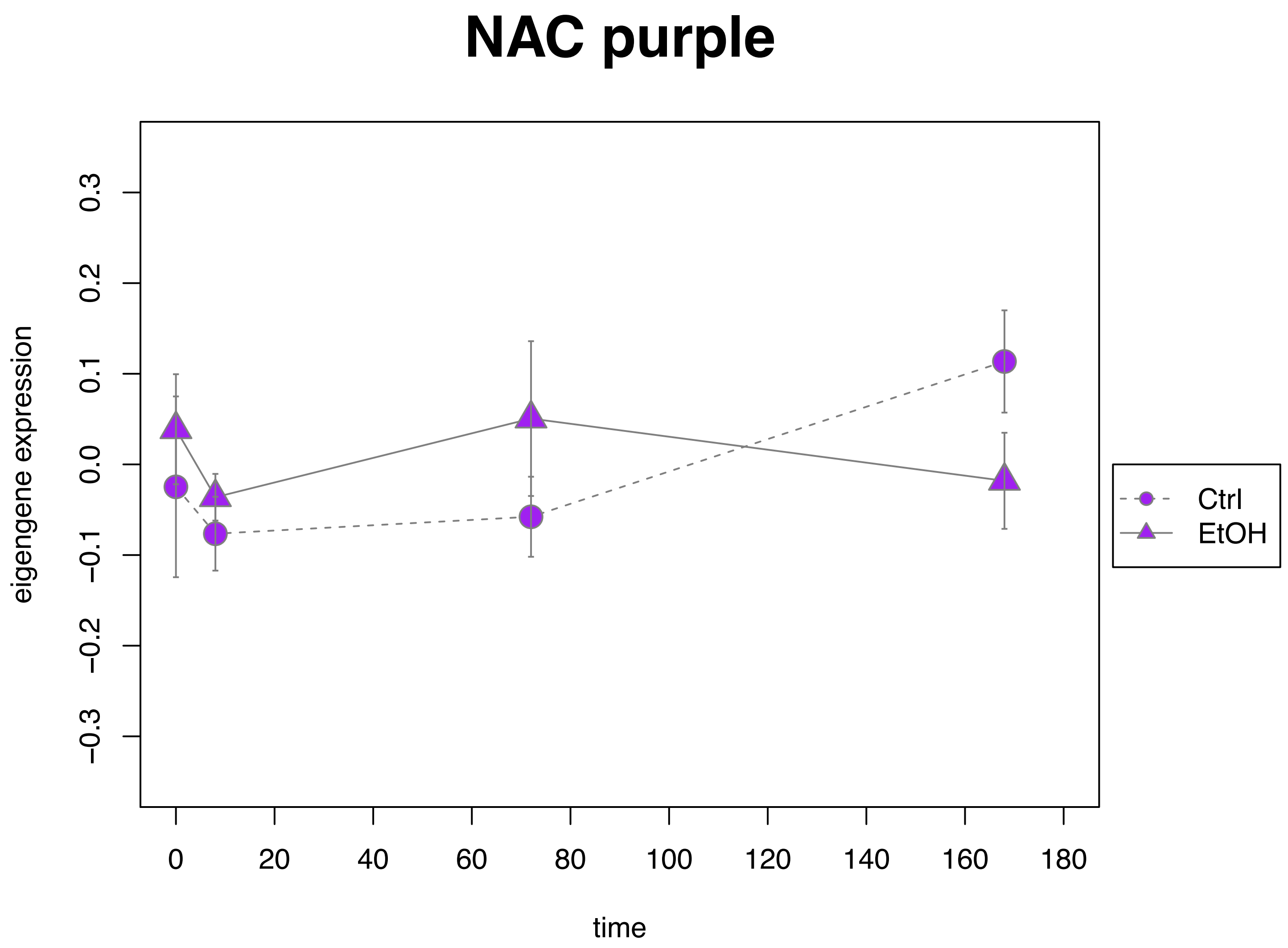

NAC purple
eigengene expression
0.3
0.2
0.1
0.0
−0.1
−0.2
−0.3
0
20
40
60
80
100
120
140
160
180
time
Ctrl
EtOH

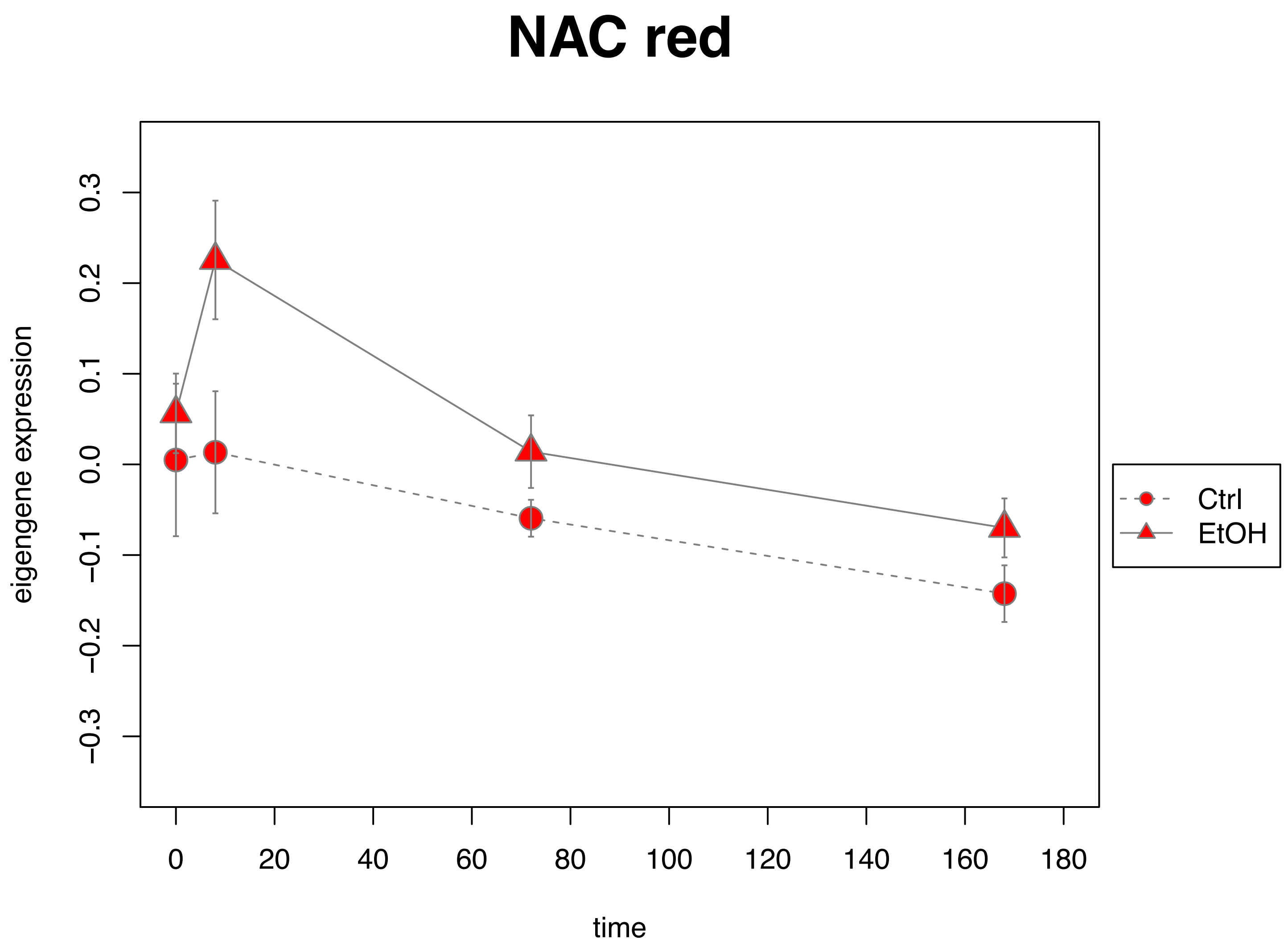
NAC red
eigengene expression
0.3
0.2
0.1
0.0
−0.1
−0.2
−0.3
0
20
40
60
80
100
120
140
160
180
time
Ctrl
EtOH

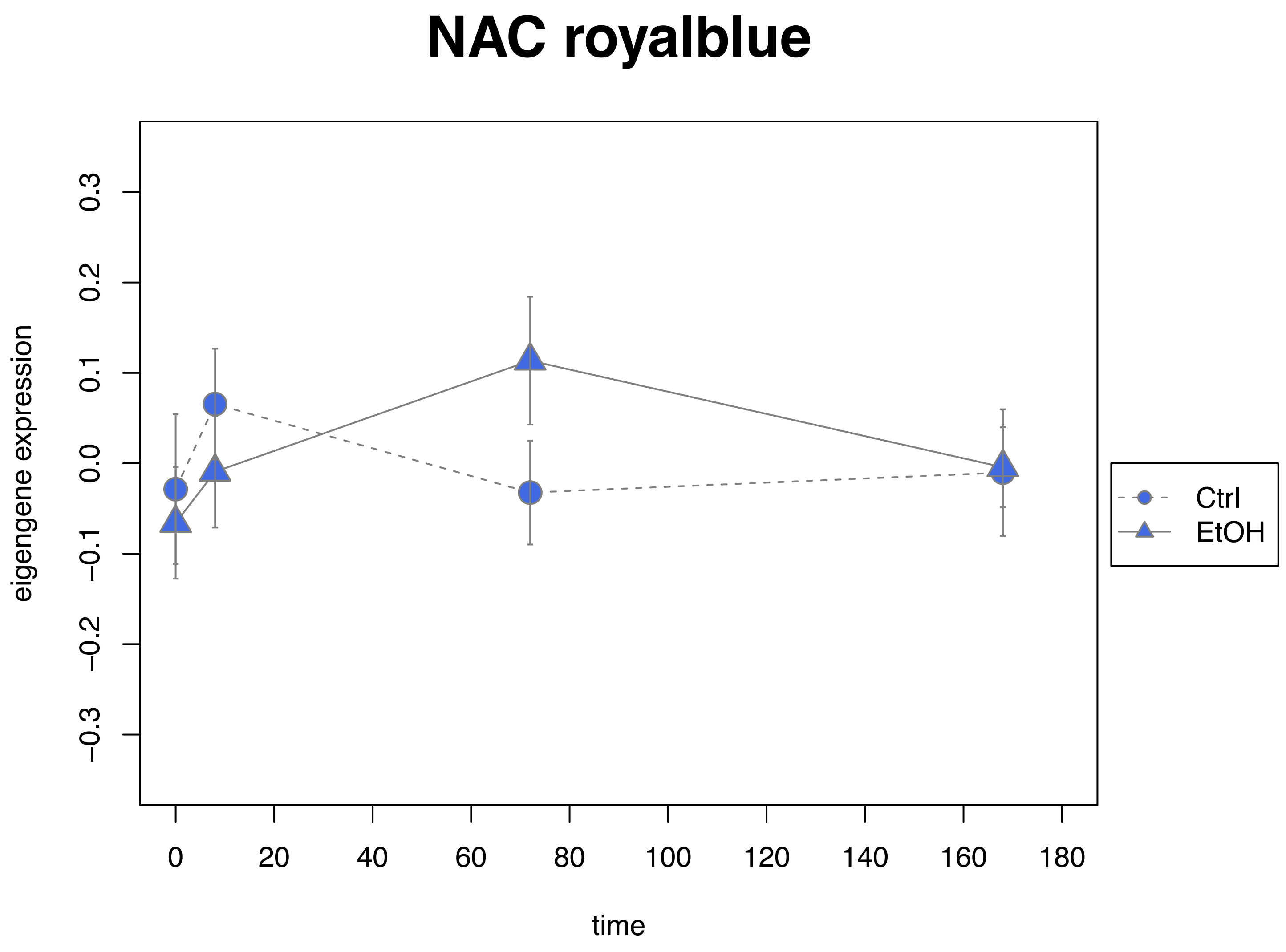
NAC royalblue
eigengene expression
time
0.3
0.2
0.1
0.0
−0.1
−0.2
−0.3
0
20
40
60
80
100
120
140
160
180
Ctrl
EtOH

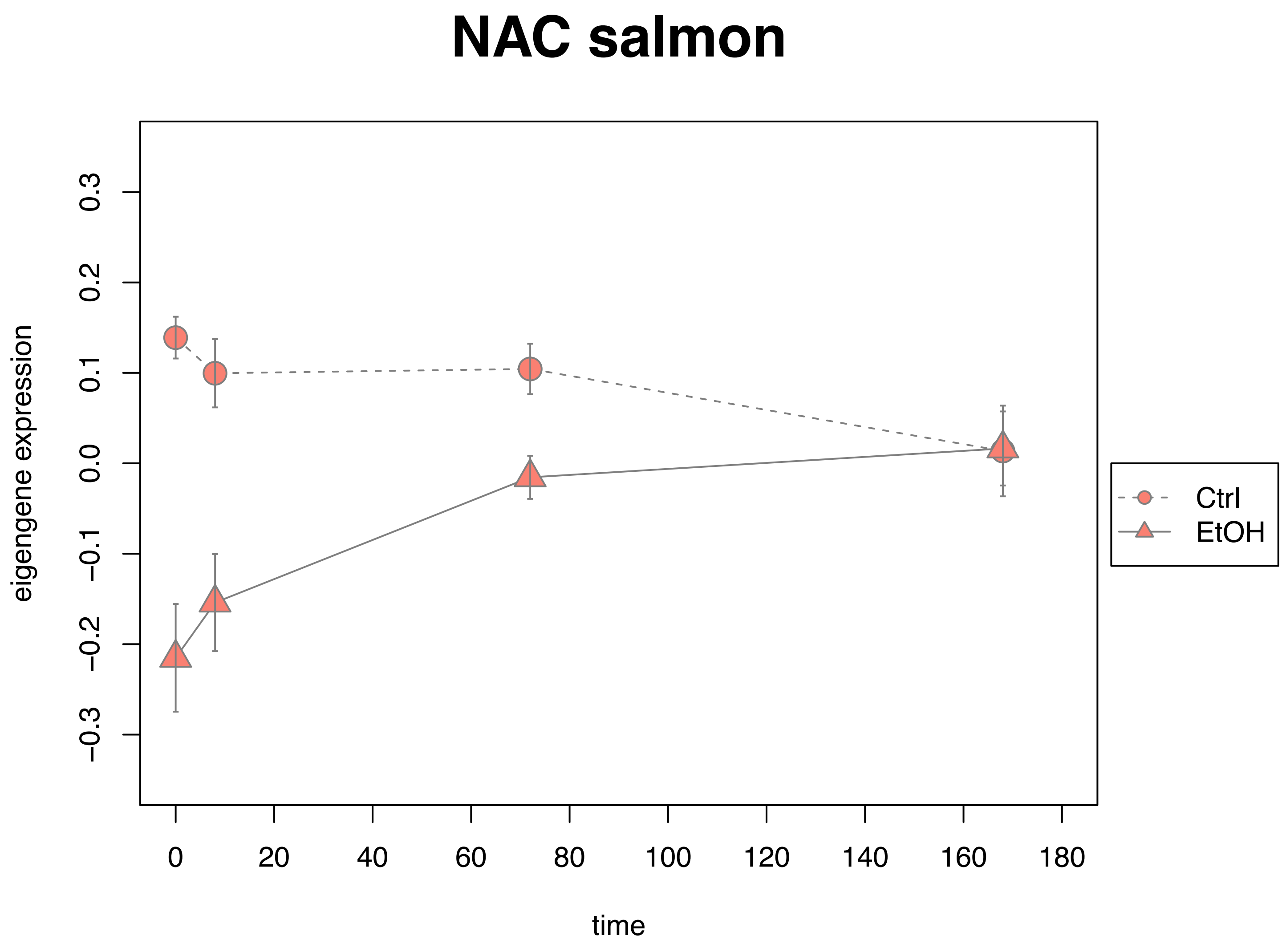
NAC salmon
eigengene expression
0.3
0.2
0.1
0.0
−0.1
−0.2
−0.3
0
20
40
60
80
100
120
140
160
180
time
Ctrl
EtOH

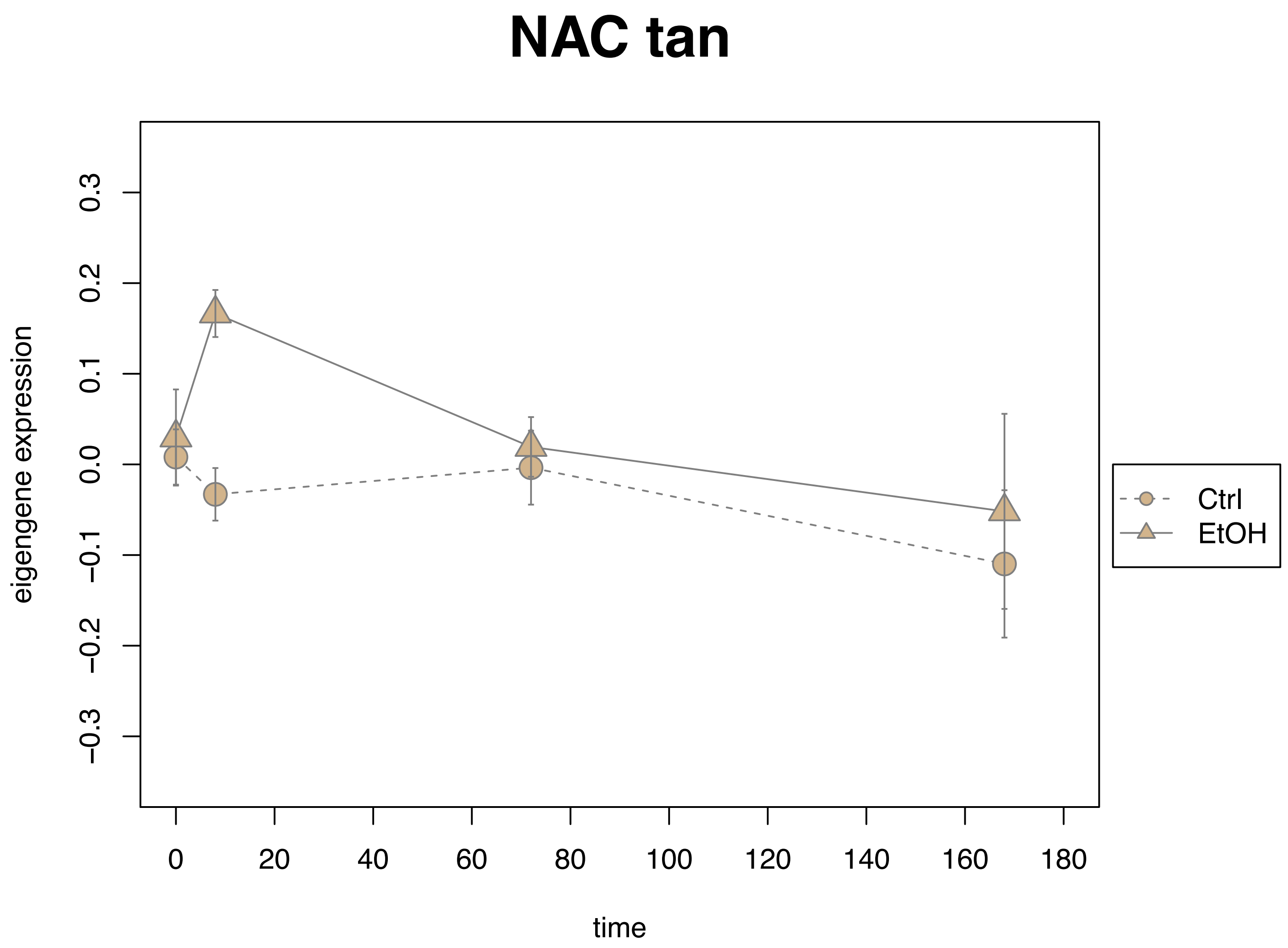
NAC tan
eigengene expression
0.3
0.2
0.1
0.0
−0.1
−0.2
−0.3
0
20
40
60
80
100
120
140
160
180
time
Ctrl
EtOH

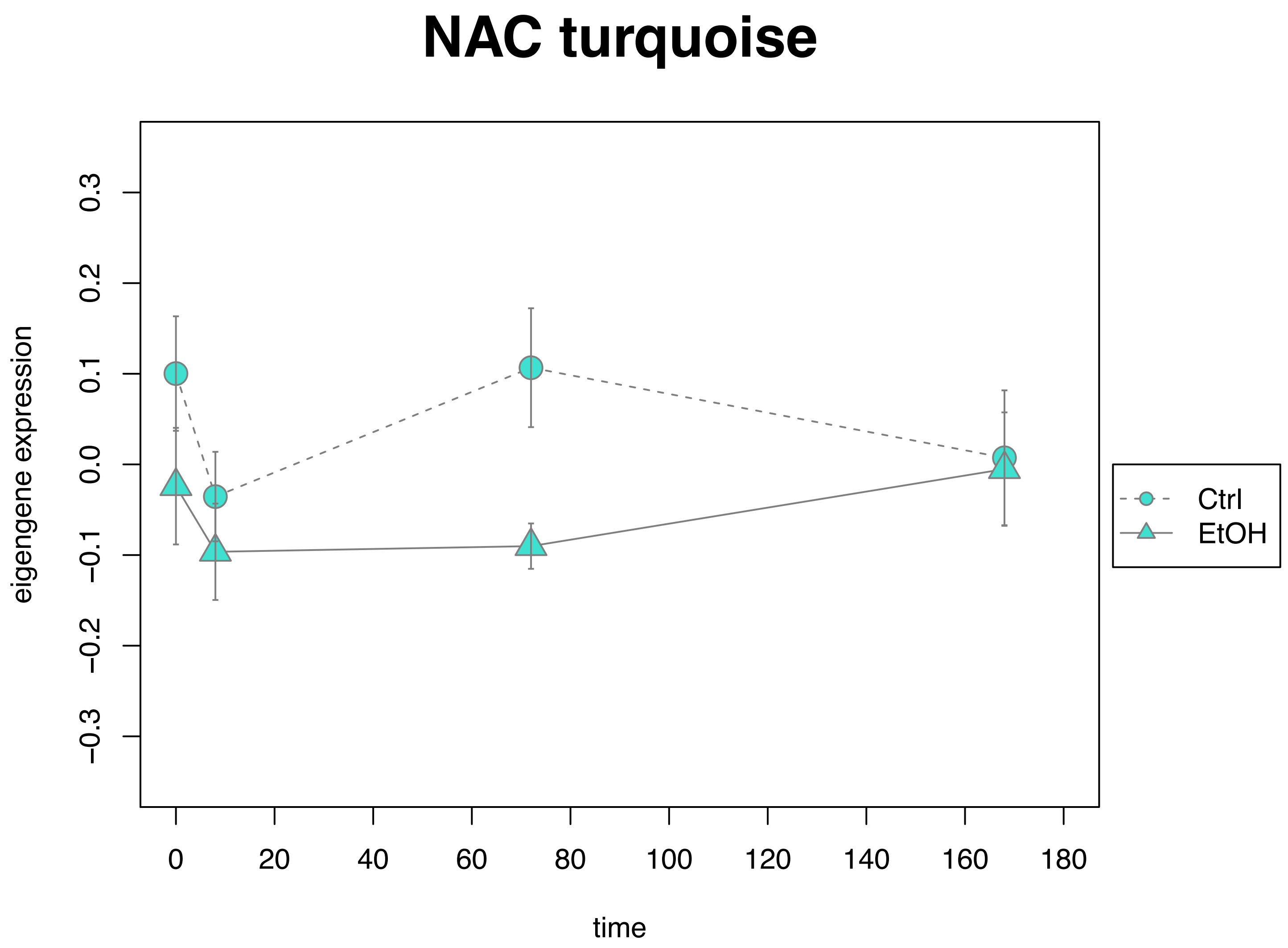

NAC turquoise
eigengene expression
0.3
0.2
0.1
0.0
−0.1
−0.2
−0.3
0
20
40
60
80
100
120
140
160
180
time
Ctrl
EtOH

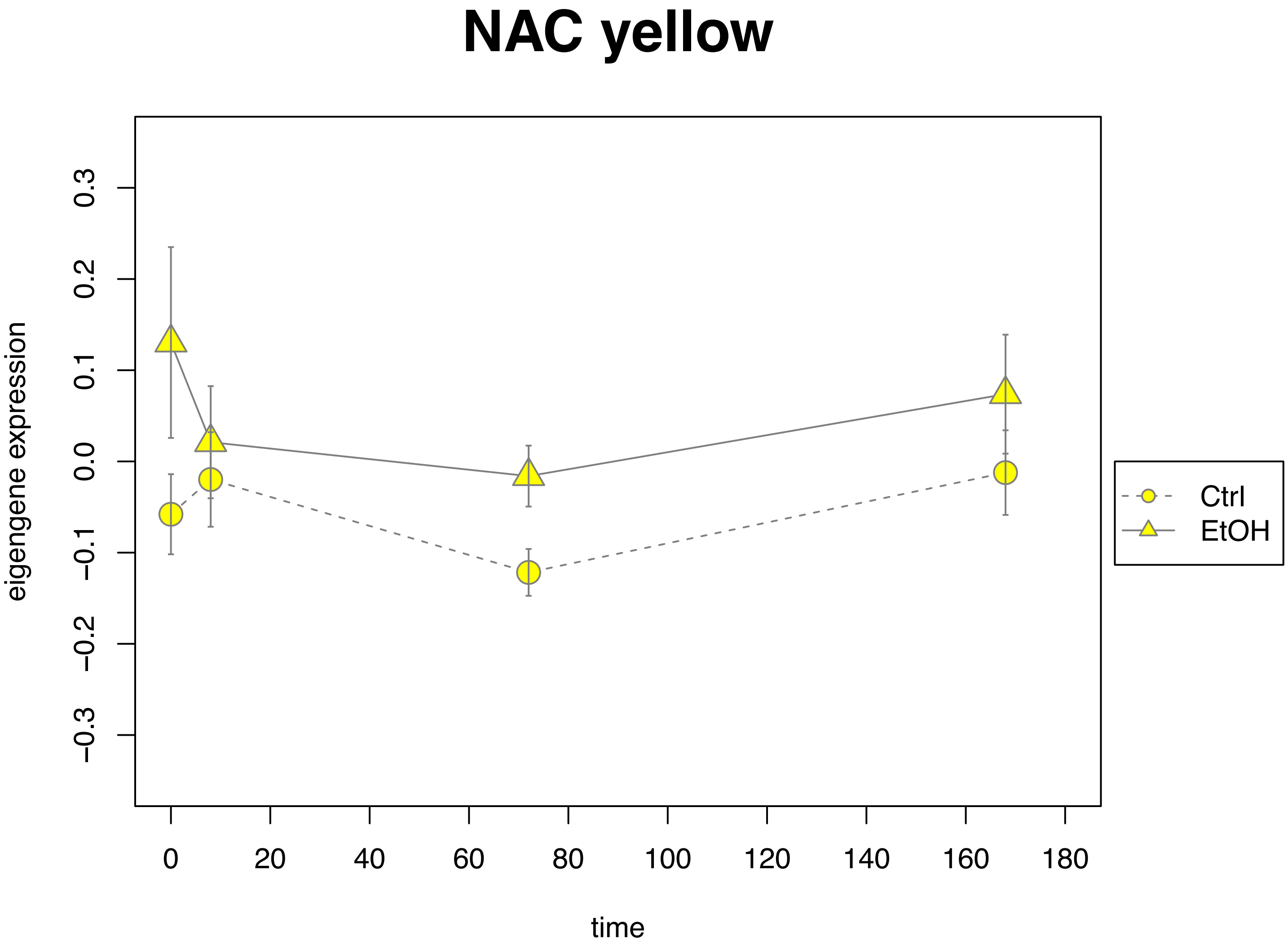
NAC yellow
eigengene expression
0.3
0.2
0.1
0.0
−0.1
−0.2
−0.3
0
20
40
60
80
100
120
140
160
180
time
Ctrl
EtOH
