# Supplementary material for: Time-Course Analysis of Brain Regional Expression Network Responses to Chronic Intermittent Ethanol and Withdrawal: Implications for Mechanisms Underlying Excessive Ethanol Consumption
Source: PLoS One. 2016 Jan 5;11(1):e0146257. doi: 10.1371/journal.pone.0146257 (PMC4701666; doi:10.1371/journal.pone.0146257)
Supplement: S3 Fig — (PDF) [file pone.0146257.s003.pdf]

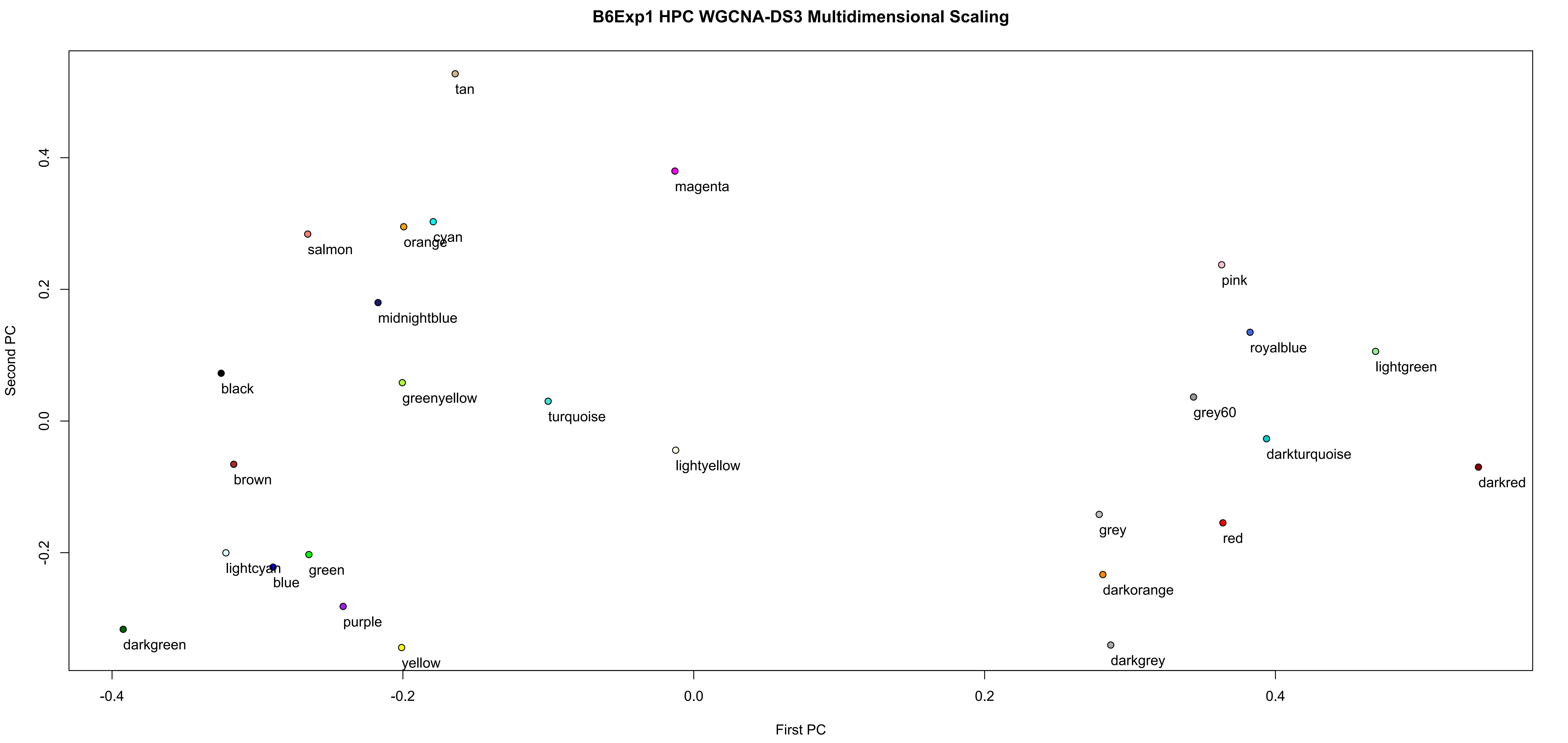
B6Exp1 HPC WGCNA-DS3 Multidimensional Scaling
Second PC
First PC
0.4
0.2
0.0
-0.2
-0.4
-0.2
0.0
0.2
0.4
tan
magenta
salmon
orange
cyan
pink
midnightblue
royalblue
lightgreen
black
greenyellow
turquoise
grey60
darkturquoise
lightyellow
brown
darkred
grey
red
lightcyan
blue
green
darkorange
purple
darkgreen
yellow
darkgrey

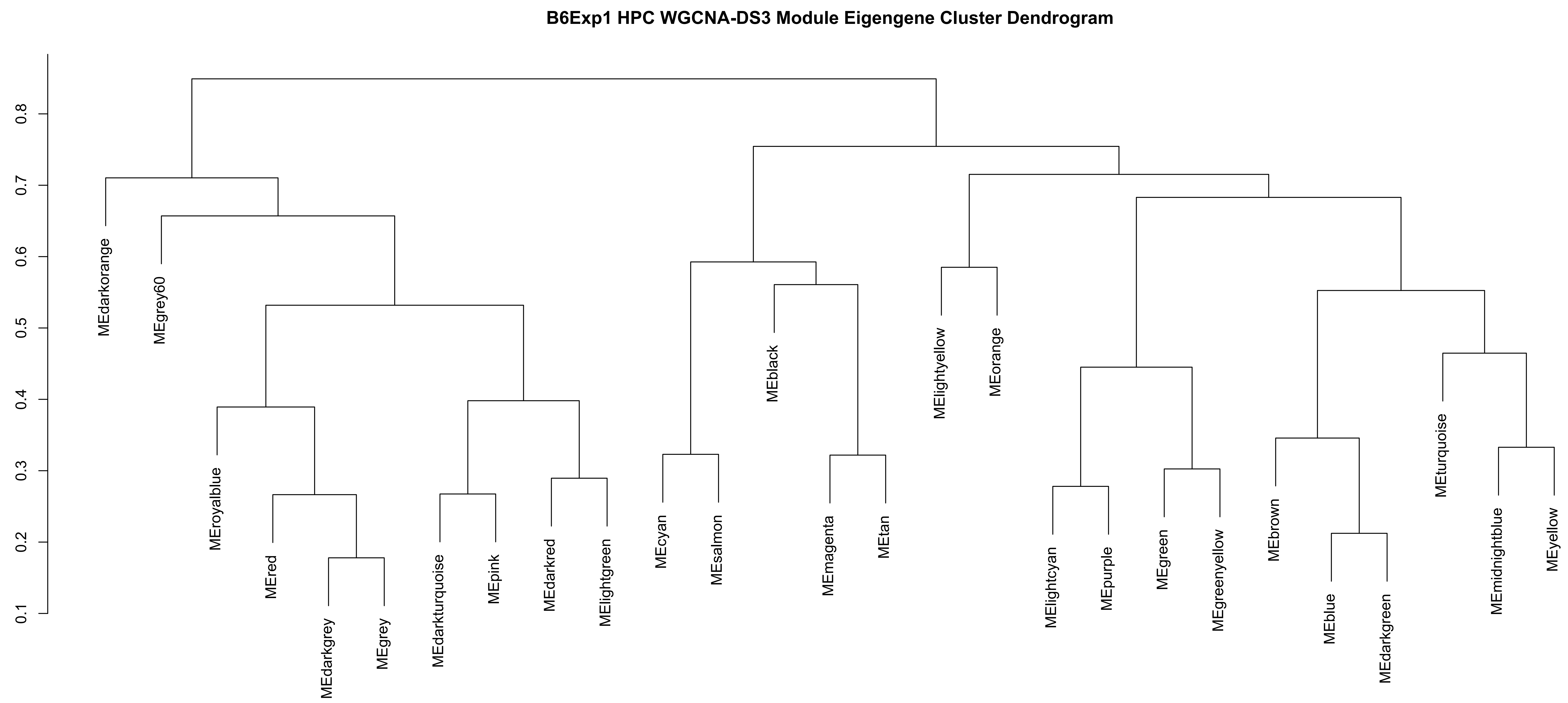
B6Exp1 HPC WGCNA-DS3 Module Eigengene Cluster Dendrogram
0.1
0.2
0.3
0.4
0.5
0.6
0.7
0.8
MEdarkorange
MEgrey60
MEroyalblue
MEred
MEdarkgrey
MEgrey
MEdarkturquoise
MEpink
MEdarkred
MElightgreen
MEcyan
MEsalmon
MEblack
MEmagenta
MEtan
MElightyellow
MEorange
MElightcyan
MEpurple
MEgreen
MEgreenyellow
MEbrown
MEblue
MEdarkgreen
MEturquoise
MEmidnightblue
MEyellow

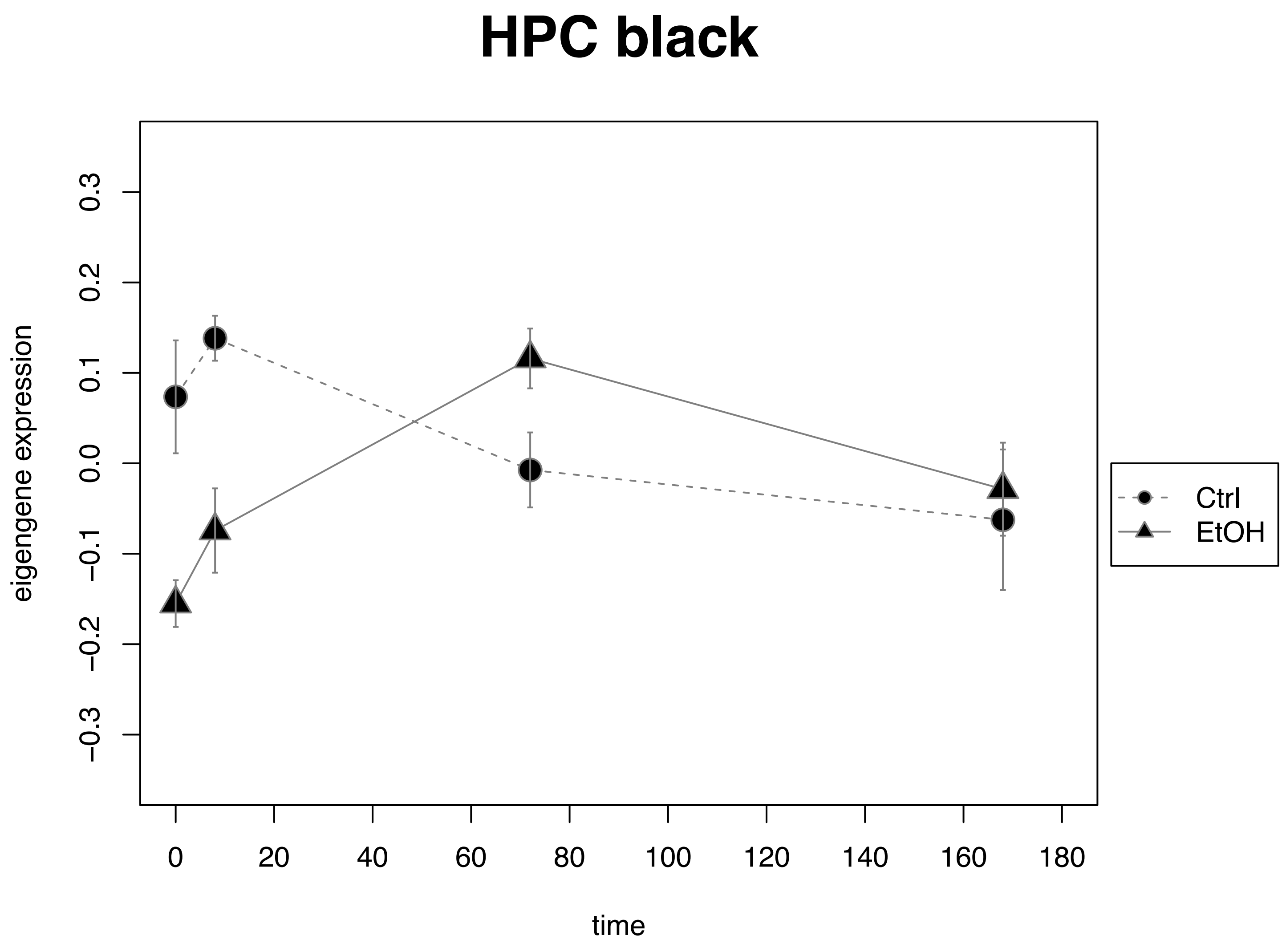
HPC black
eigengene expression
0.3
0.2
0.1
0.0
−0.1
−0.2
−0.3
0
20
40
60
80
100
120
140
160
180
time
Ctrl
EtOH

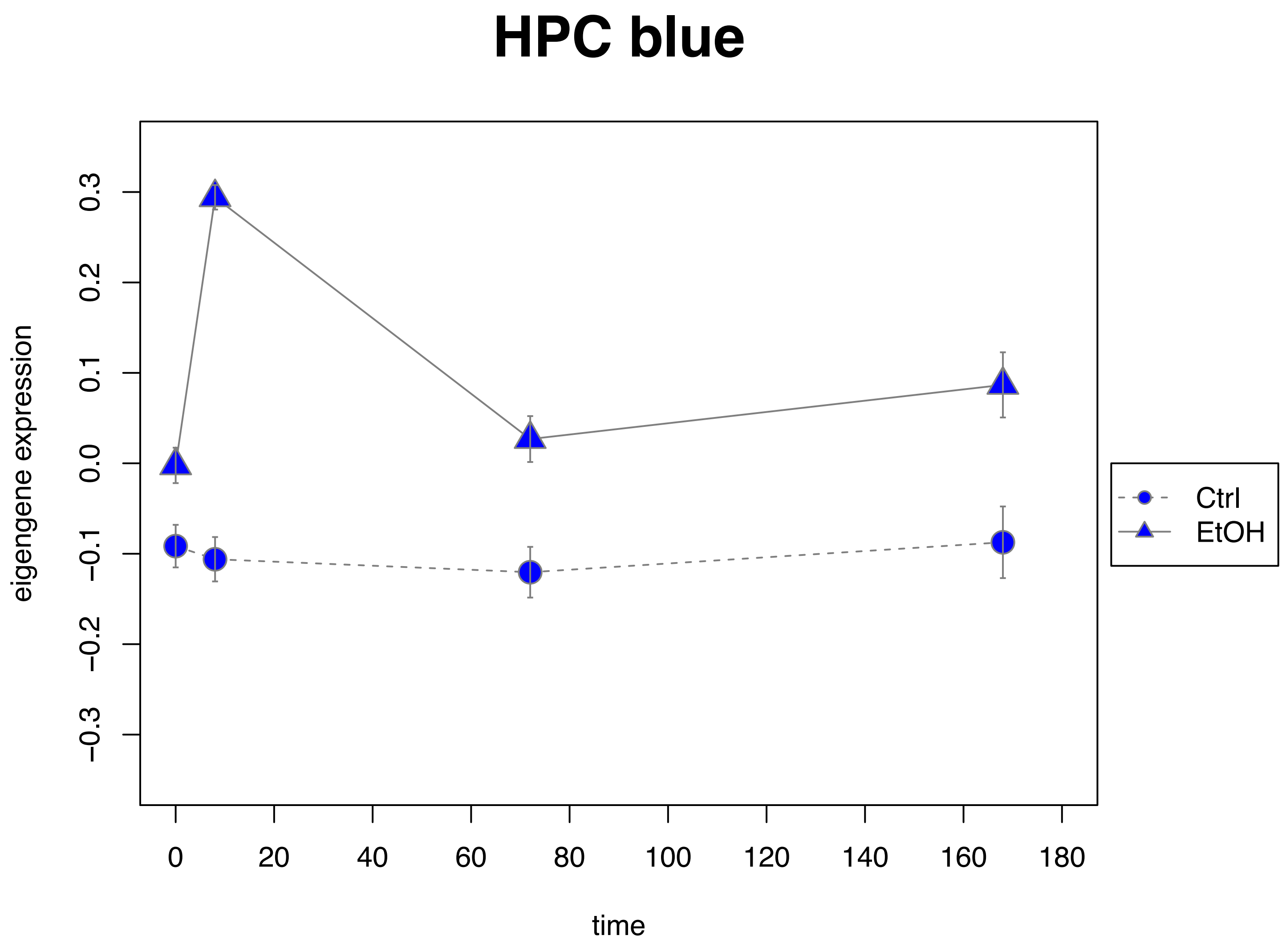
HPC blue
eigengene expression
time
0.3
0.2
0.1
0.0
−0.1
−0.2
−0.3
0
20
40
60
80
100
120
140
160
180
Ctrl
EtOH

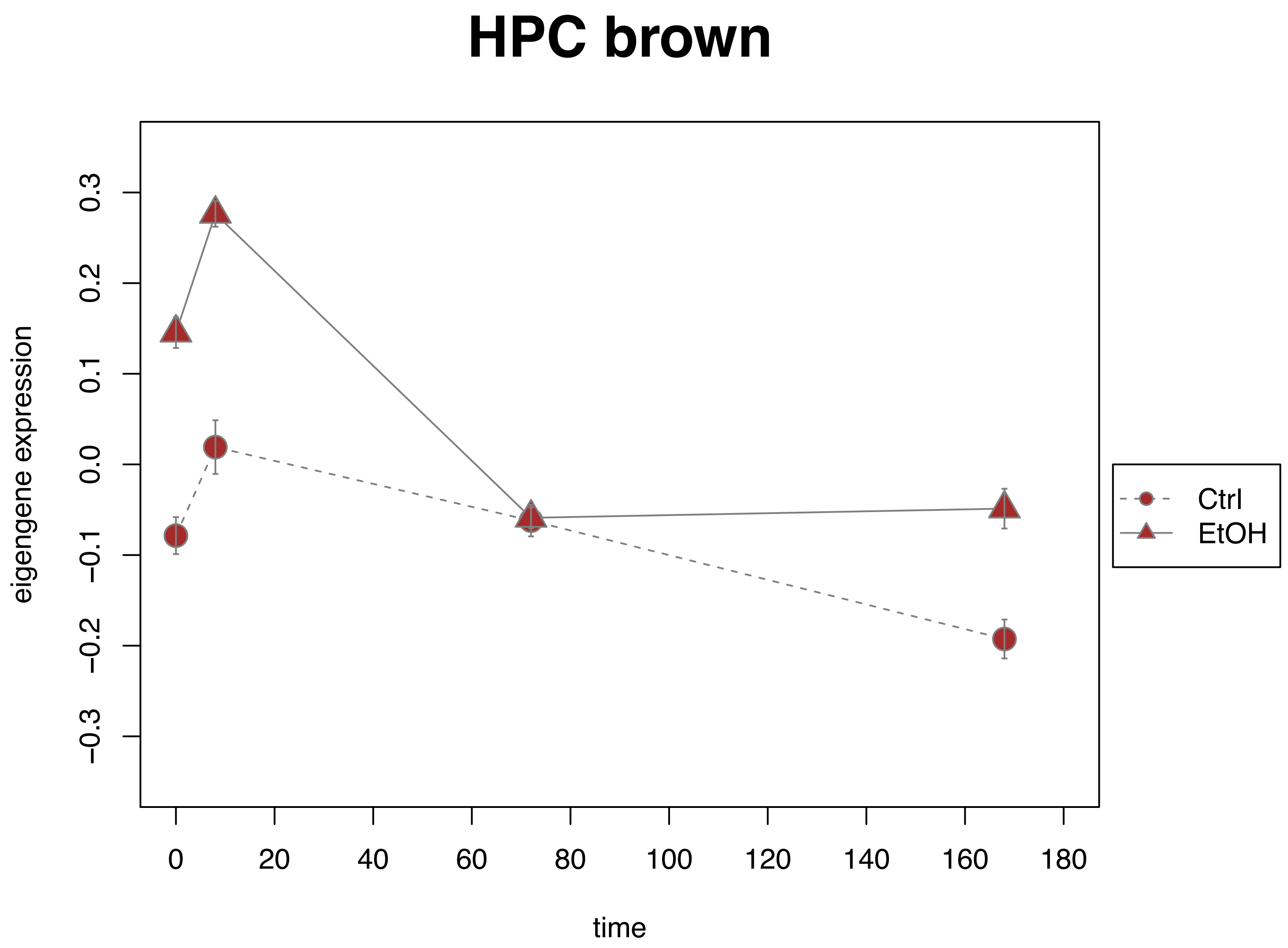
HPC brown
eigengene expression
time
Ctrl
EtOH
0.3
0.2
0.1
0.0
−0.1
−0.2
−0.3
0
20
40
60
80
100
120
140
160
180

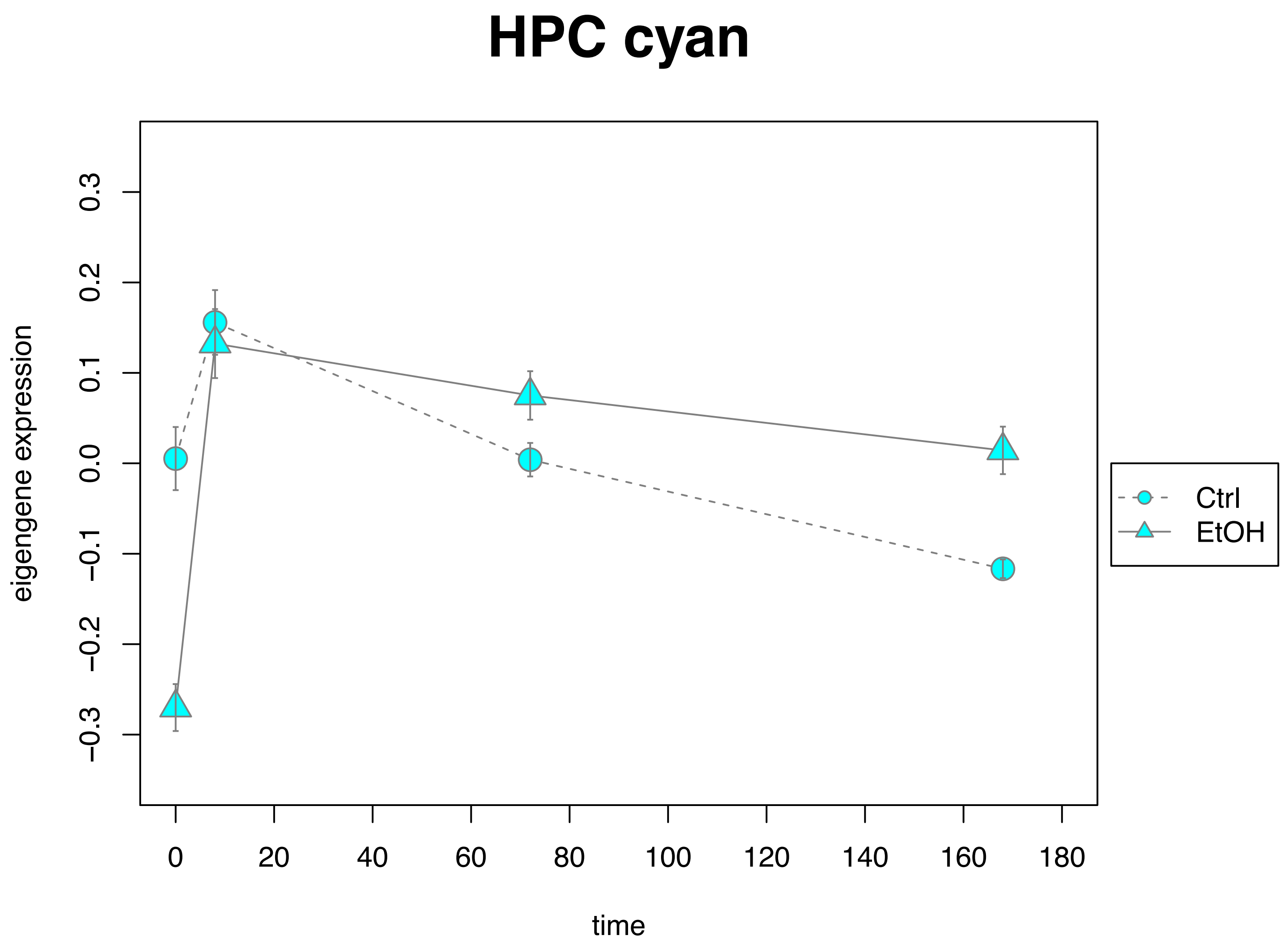
HPC cyan
eigengene expression
time
0.3
0.2
0.1
0.0
−0.1
−0.2
−0.3
0
20
40
60
80
100
120
140
160
180
Ctrl
EtOH

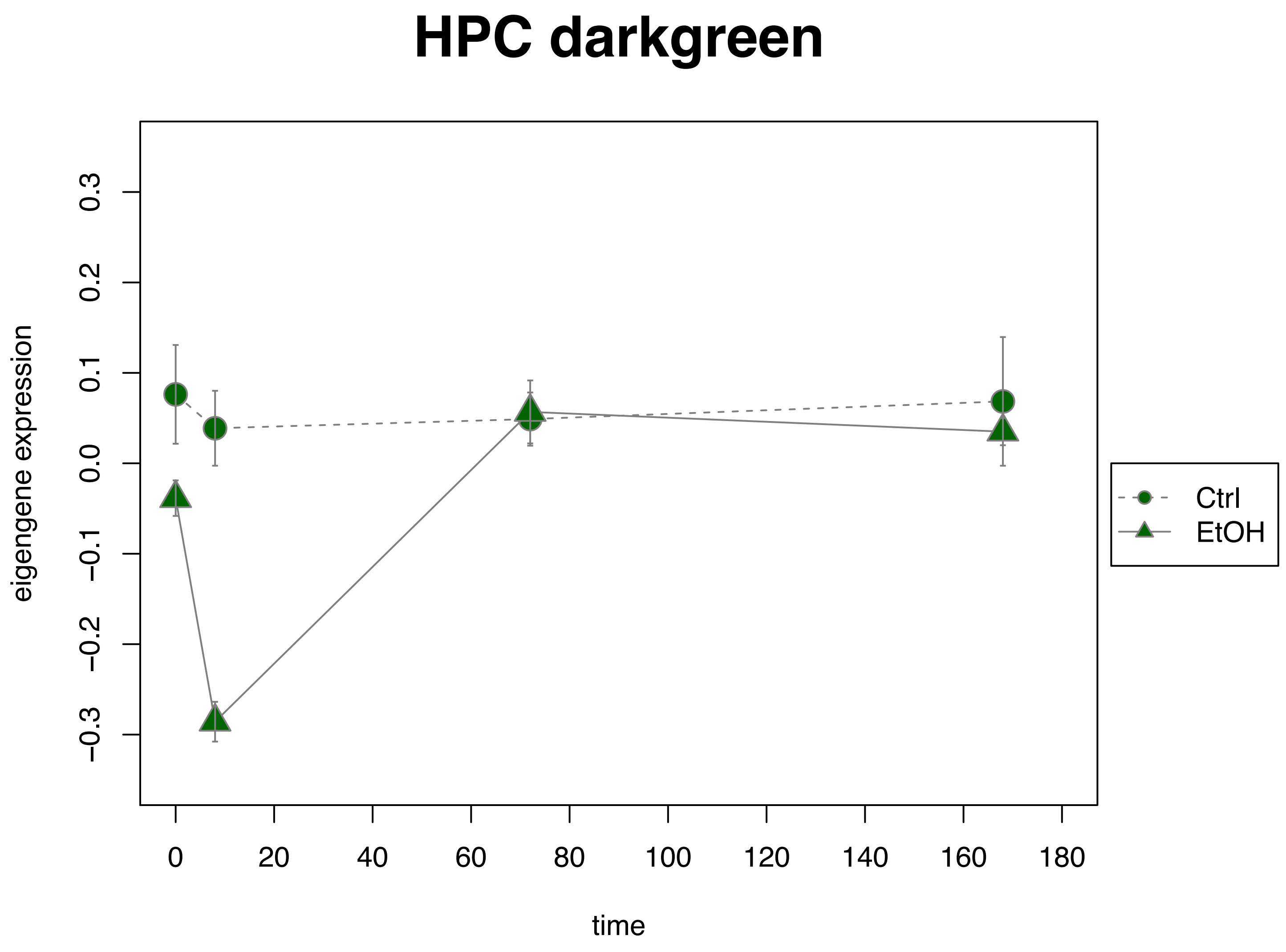
HPC darkgreen
eigengene expression
0.3
0.2
0.1
0.0
−0.1
−0.2
−0.3
0
20
40
60
80
100
120
140
160
180
time
Ctrl
EtOH

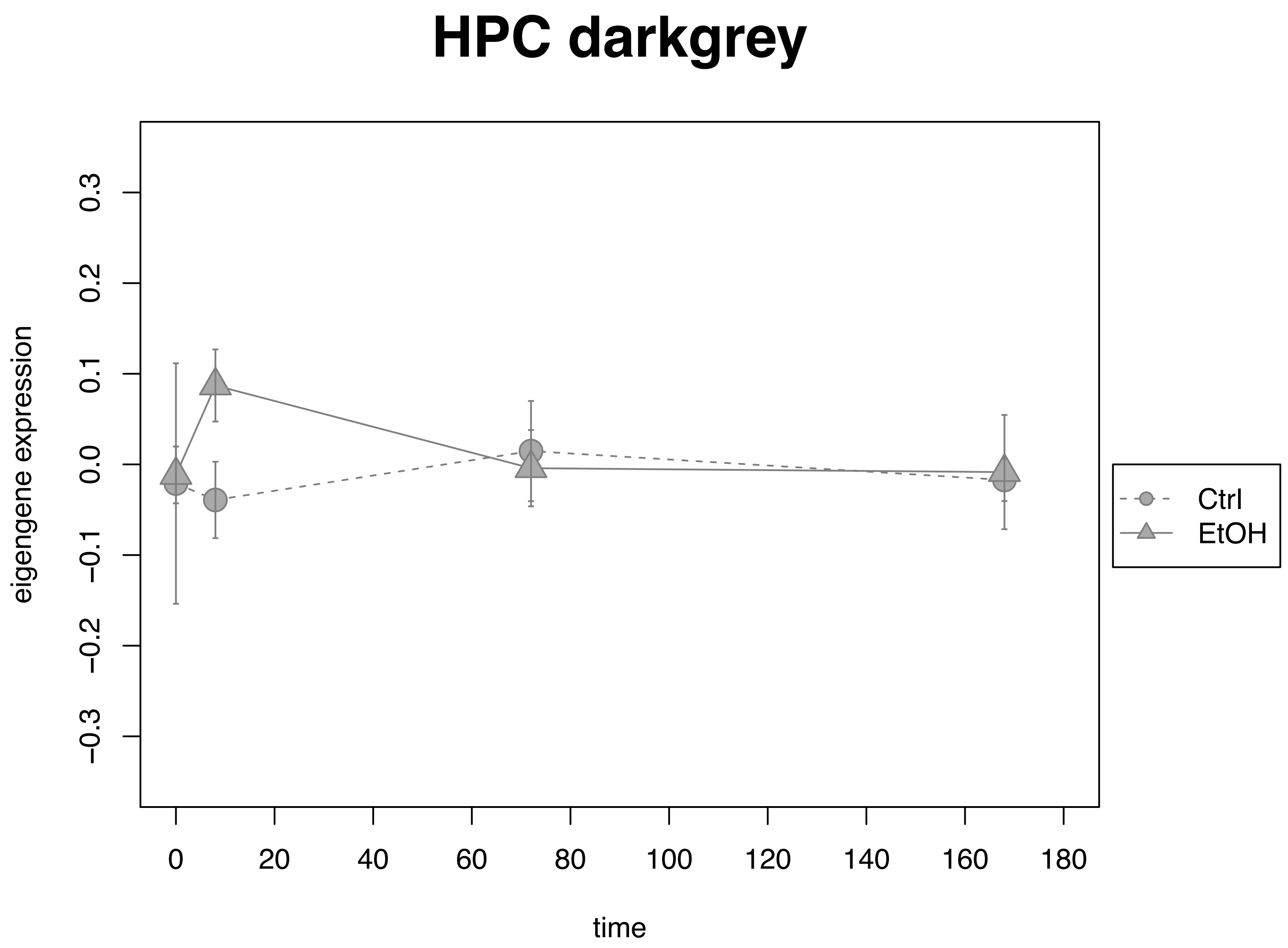
HPC darkgrey
eigengene expression
0.3
0.2
0.1
0.0
−0.1
−0.2
−0.3
0
20
40
60
80
100
120
140
160
180
time
Ctrl
EtOH

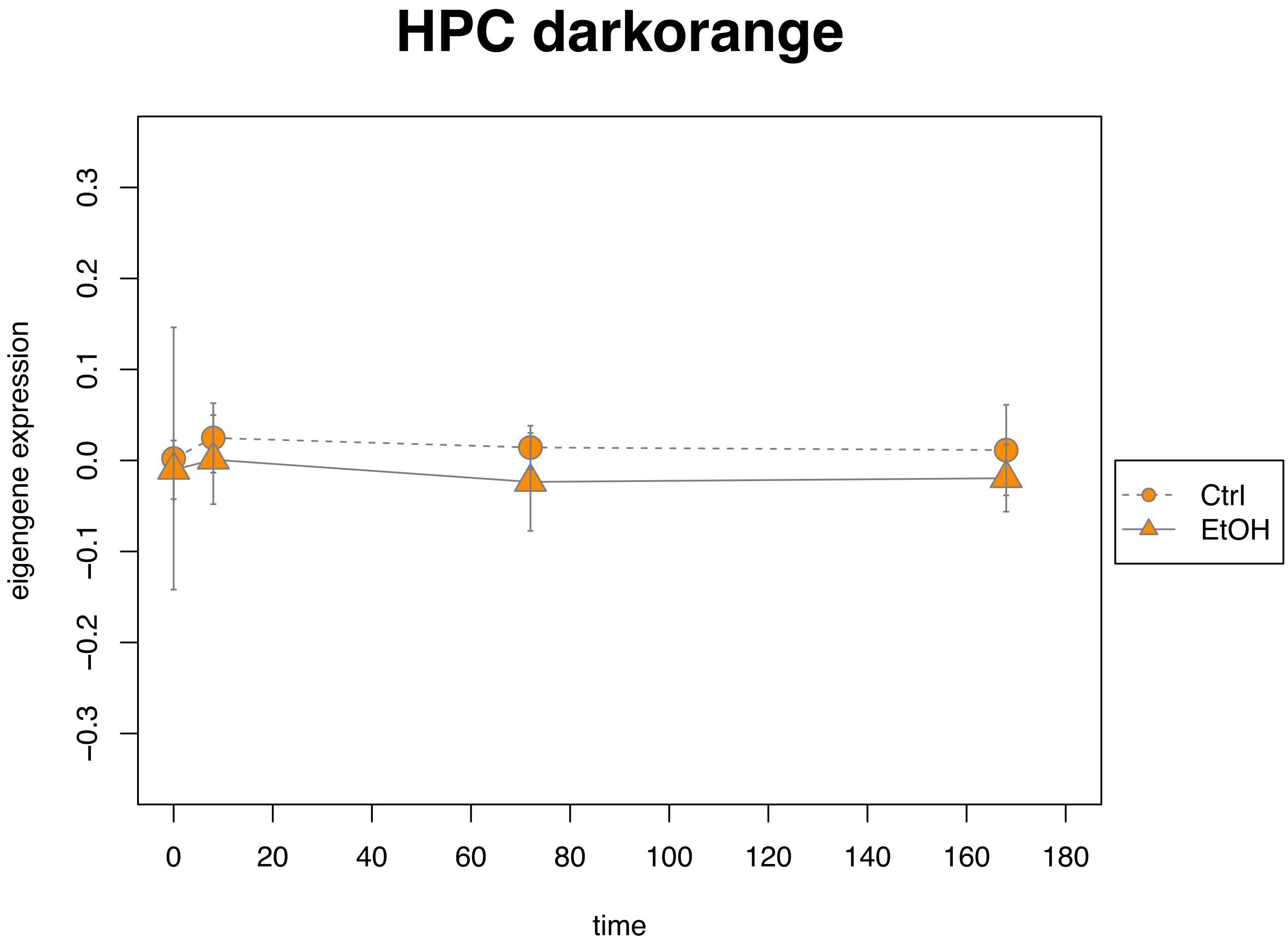
HPC darkorange
eigengene expression
0.3
0.2
0.1
0.0
−0.1
−0.2
−0.3
0
20
40
60
80
100
120
140
160
180
time
Ctrl
EtOH

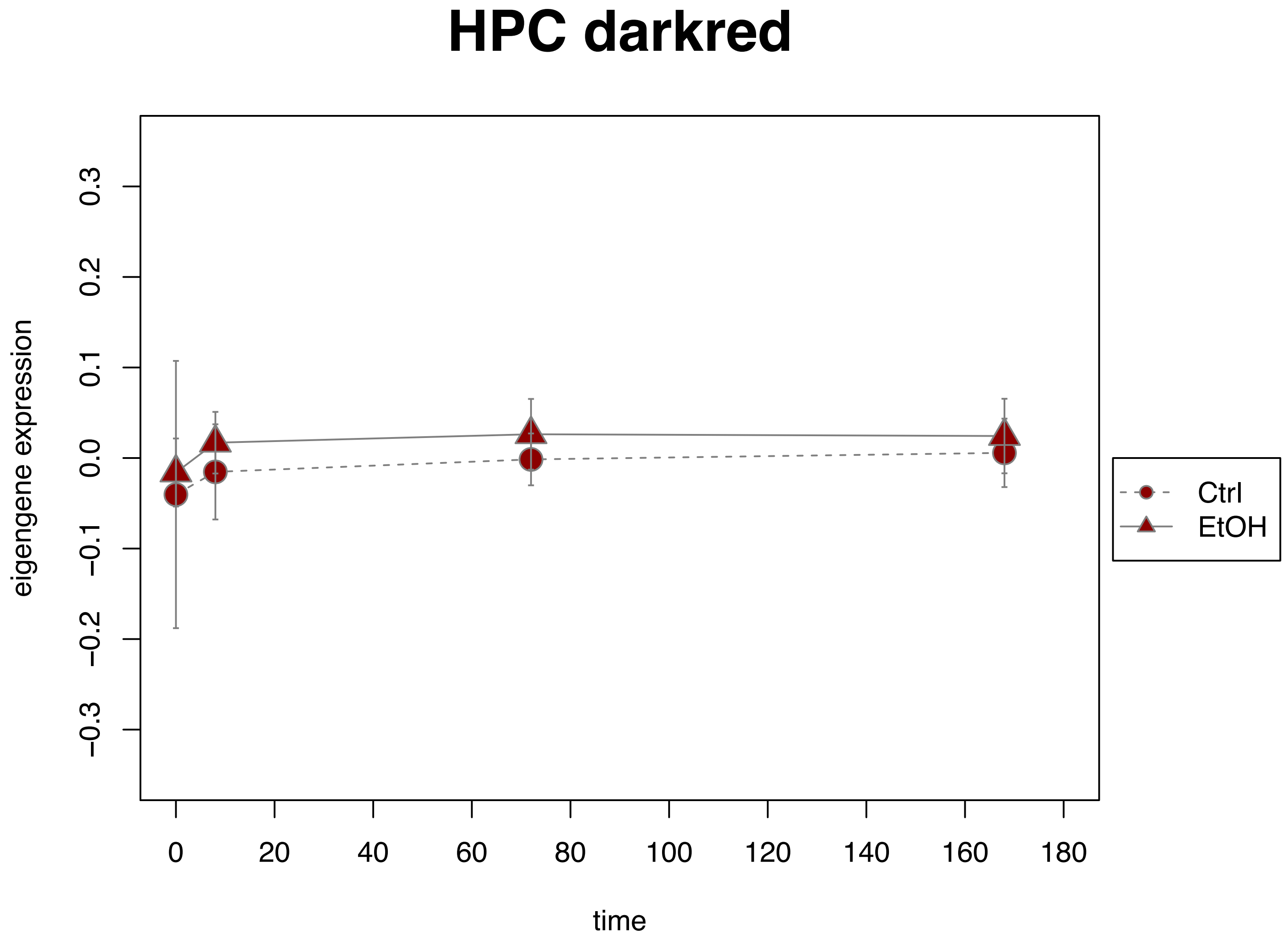

HPC darkred
eigengene expression
0.3
0.2
0.1
0.0
−0.1
−0.2
−0.3
0
20
40
60
80
100
120
140
160
180
time
Ctrl
EtOH

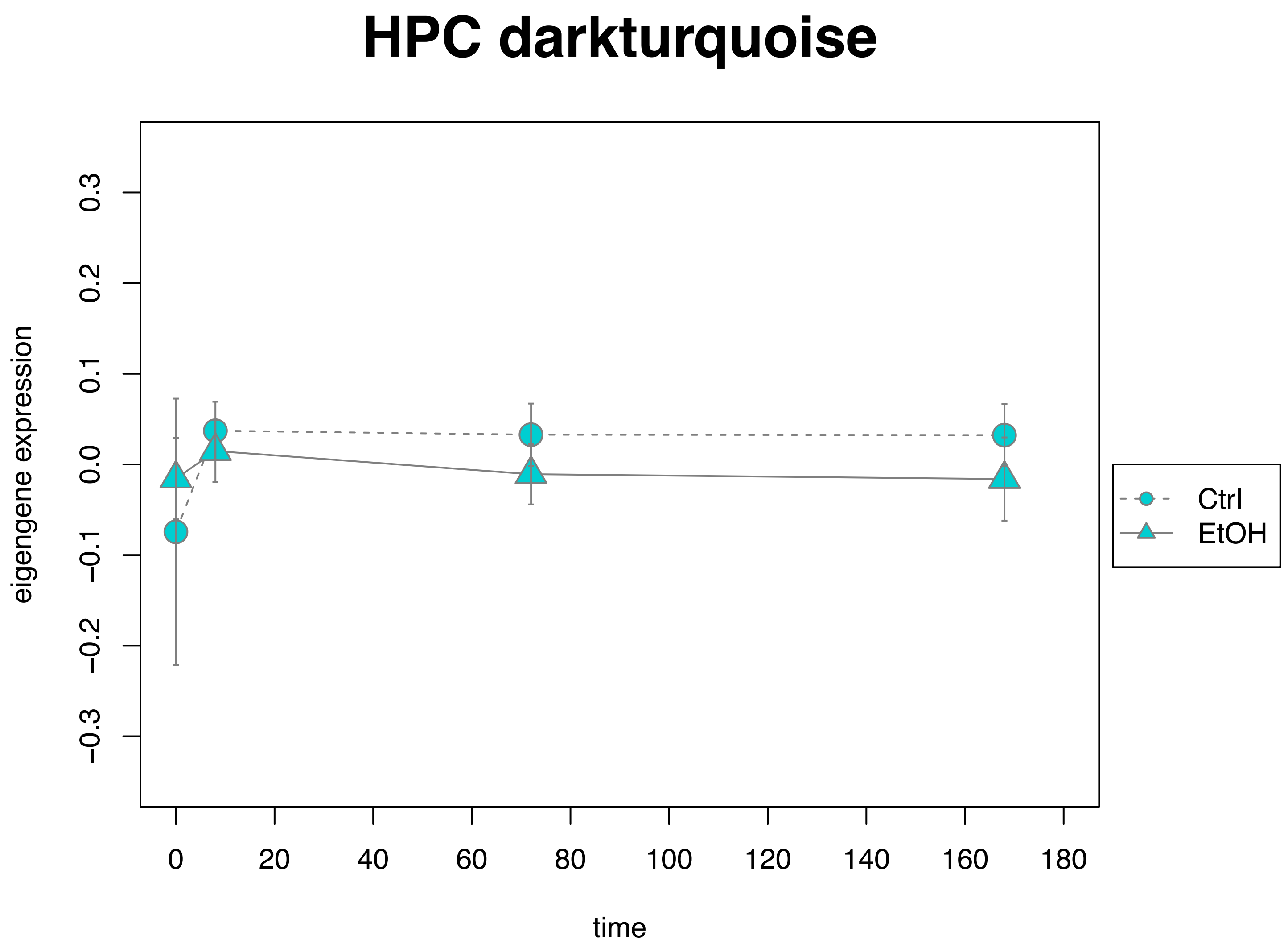

HPC darkturquoise
eigengene expression
0.3
0.2
0.1
0.0
−0.1
−0.2
−0.3
0
20
40
60
80
100
120
140
160
180
time
Ctrl
EtOH

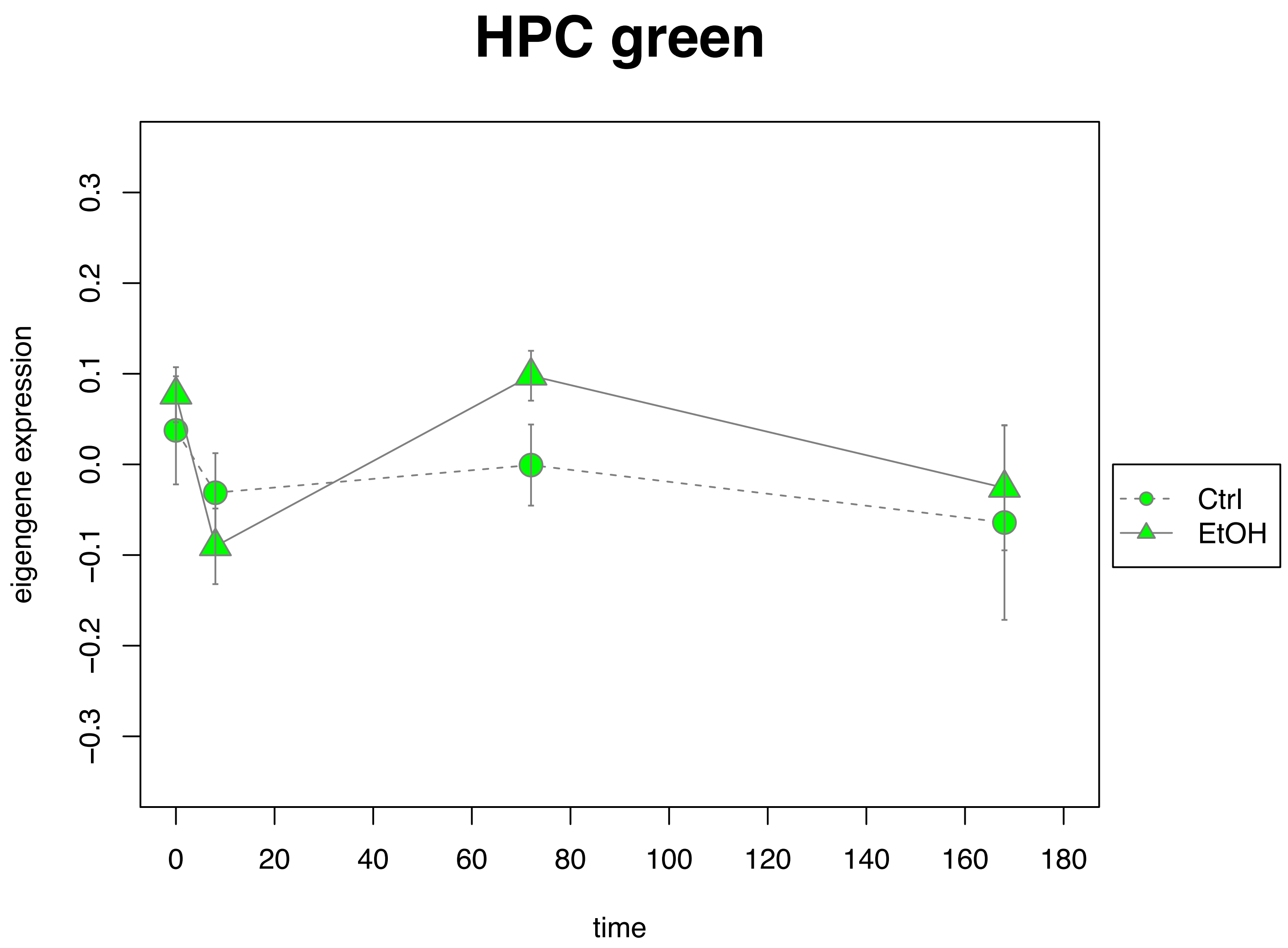
HPC green
eigengene expression
0.3
0.2
0.1
0.0
−0.1
−0.2
−0.3
0
20
40
60
80
100
120
140
160
180
time
Ctrl
EtOH

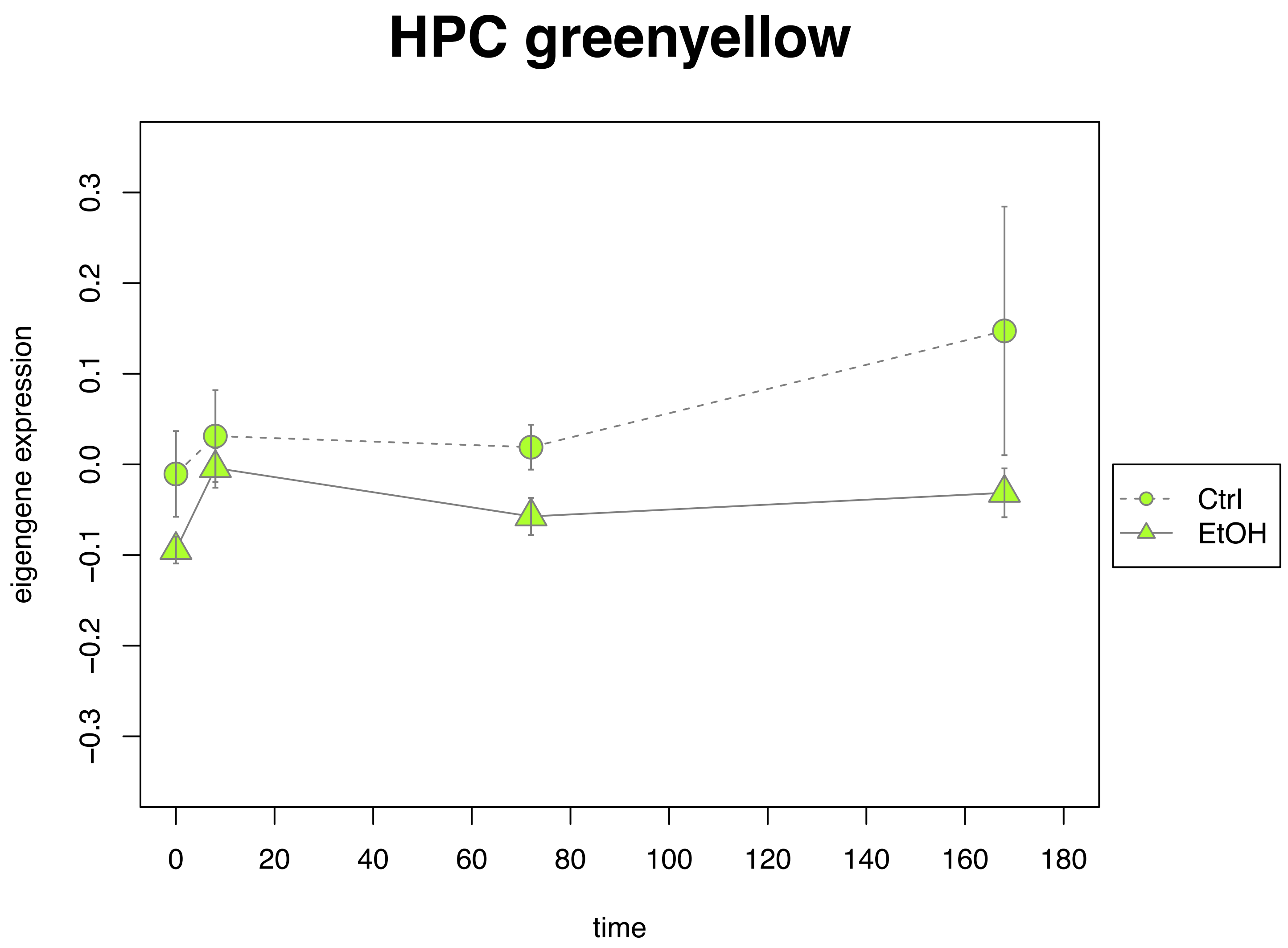
HPC greenyellow
eigengene expression
0.3
0.2
0.1
0.0
−0.1
−0.2
−0.3
0
20
40
60
80
100
120
140
160
180
time
Ctrl
EtOH

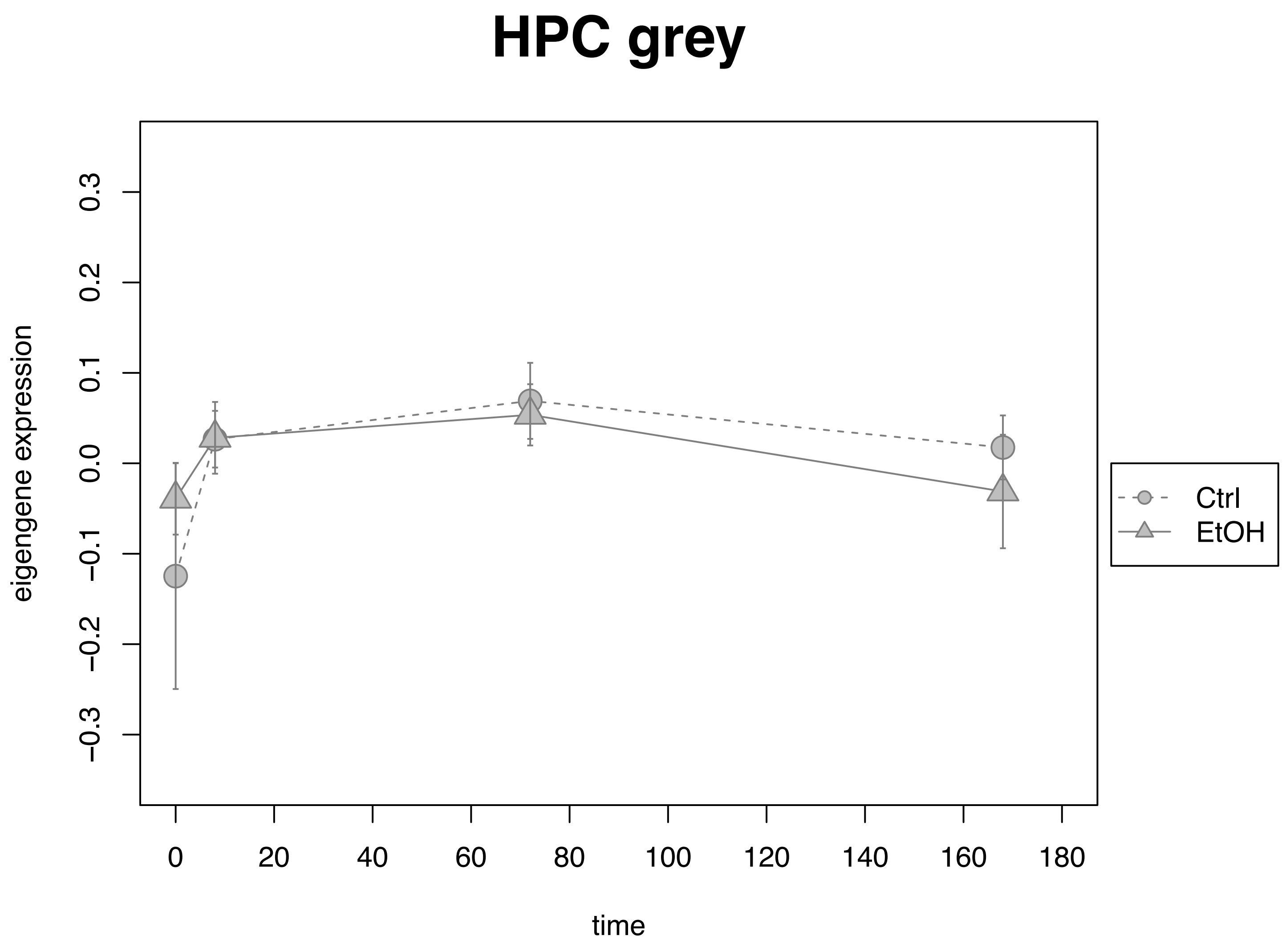

HPC grey
eigengene expression
0.3
0.2
0.1
0.0
−0.1
−0.2
−0.3
time
0
20
40
60
80
100
120
140
160
180
Ctrl
EtOH

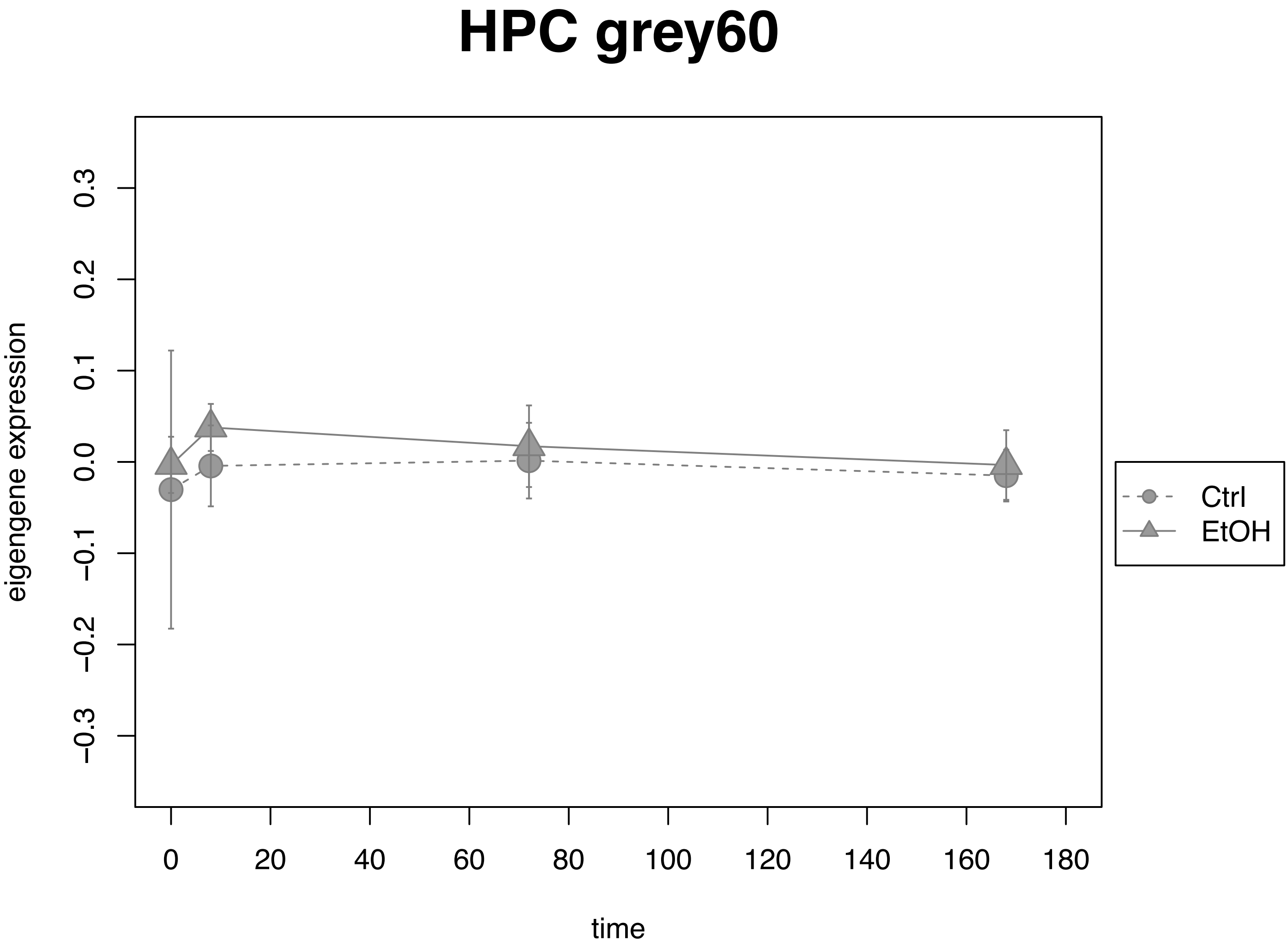

HPC grey60
eigengene expression
0.3
0.2
0.1
0.0
−0.1
−0.2
−0.3
time
0
20
40
60
80
100
120
140
160
180
Ctrl
EtOH

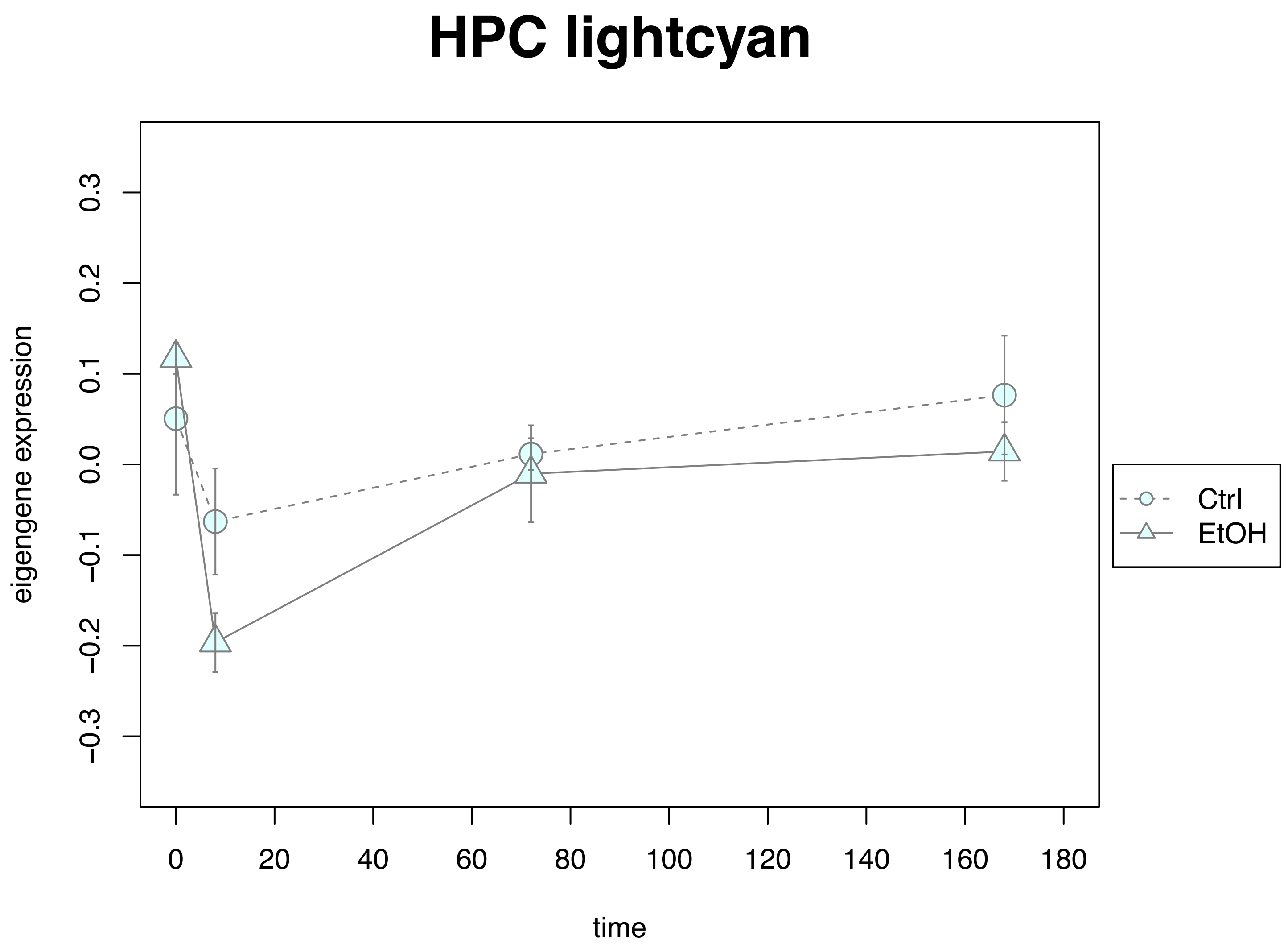
HPC lightcyan
eigengene expression
time
0.3
0.2
0.1
0.0
−0.1
−0.2
−0.3
0
20
40
60
80
100
120
140
160
180
Ctrl
EtOH

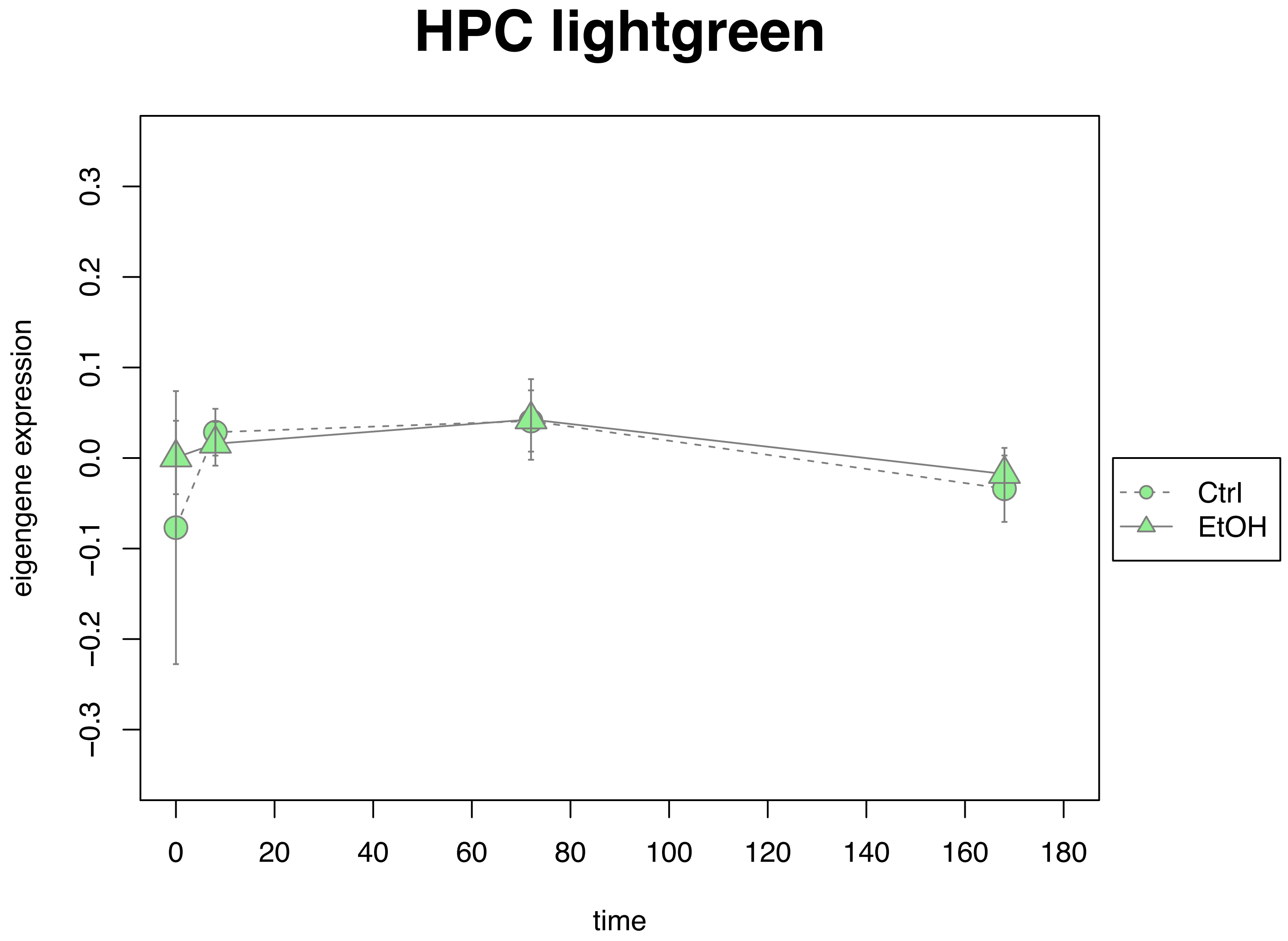

HPC lightgreen
eigengene expression
0.3
0.2
0.1
0.0
−0.1
−0.2
−0.3
0
20
40
60
80
100
120
140
160
180
time
Ctrl
EtOH

HPC lightyellow

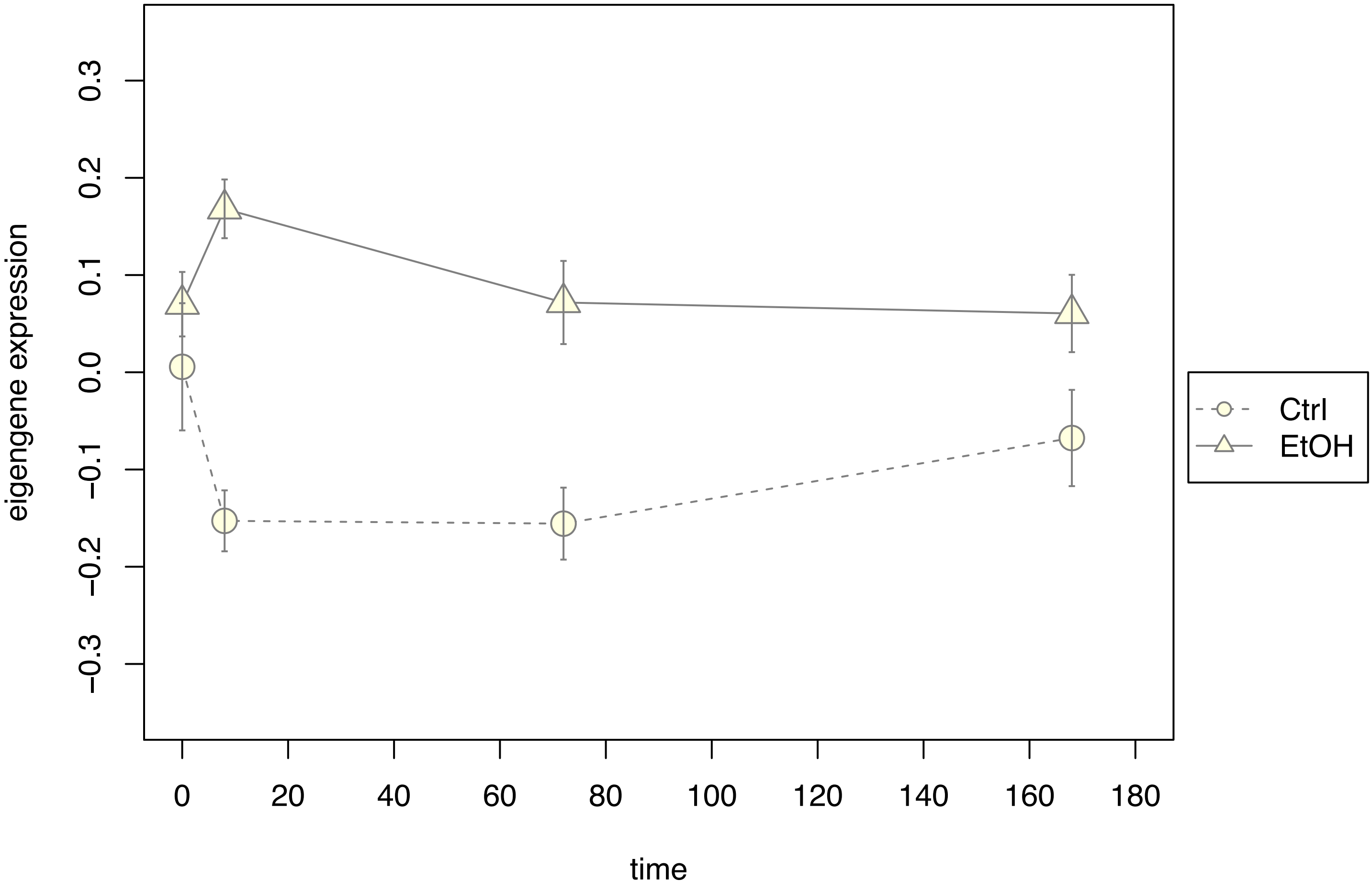

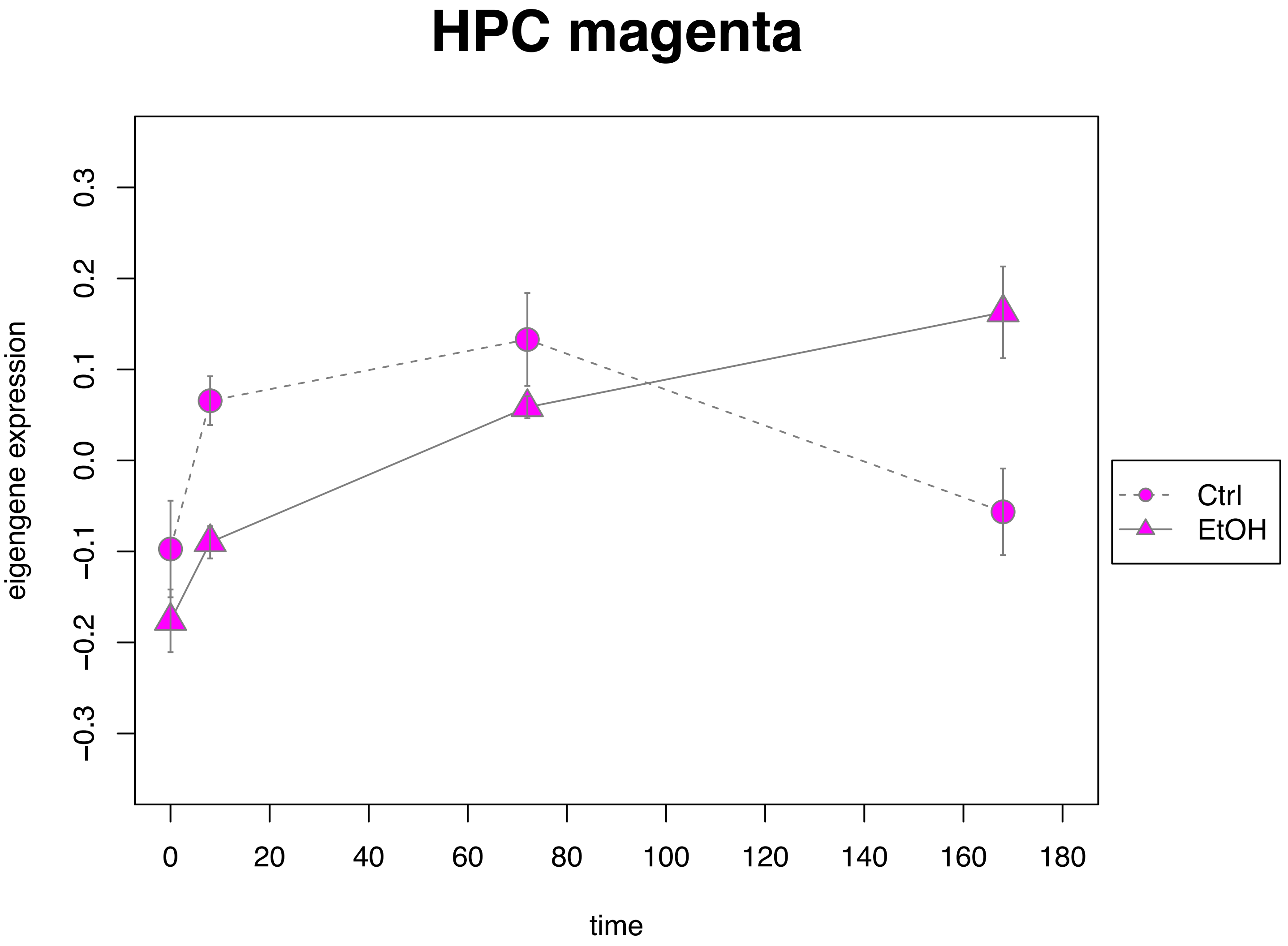
HPC magenta
eigengene expression
time
0.3
0.2
0.1
0.0
−0.1
−0.2
−0.3
0
20
40
60
80
100
120
140
160
180
Ctrl
EtOH

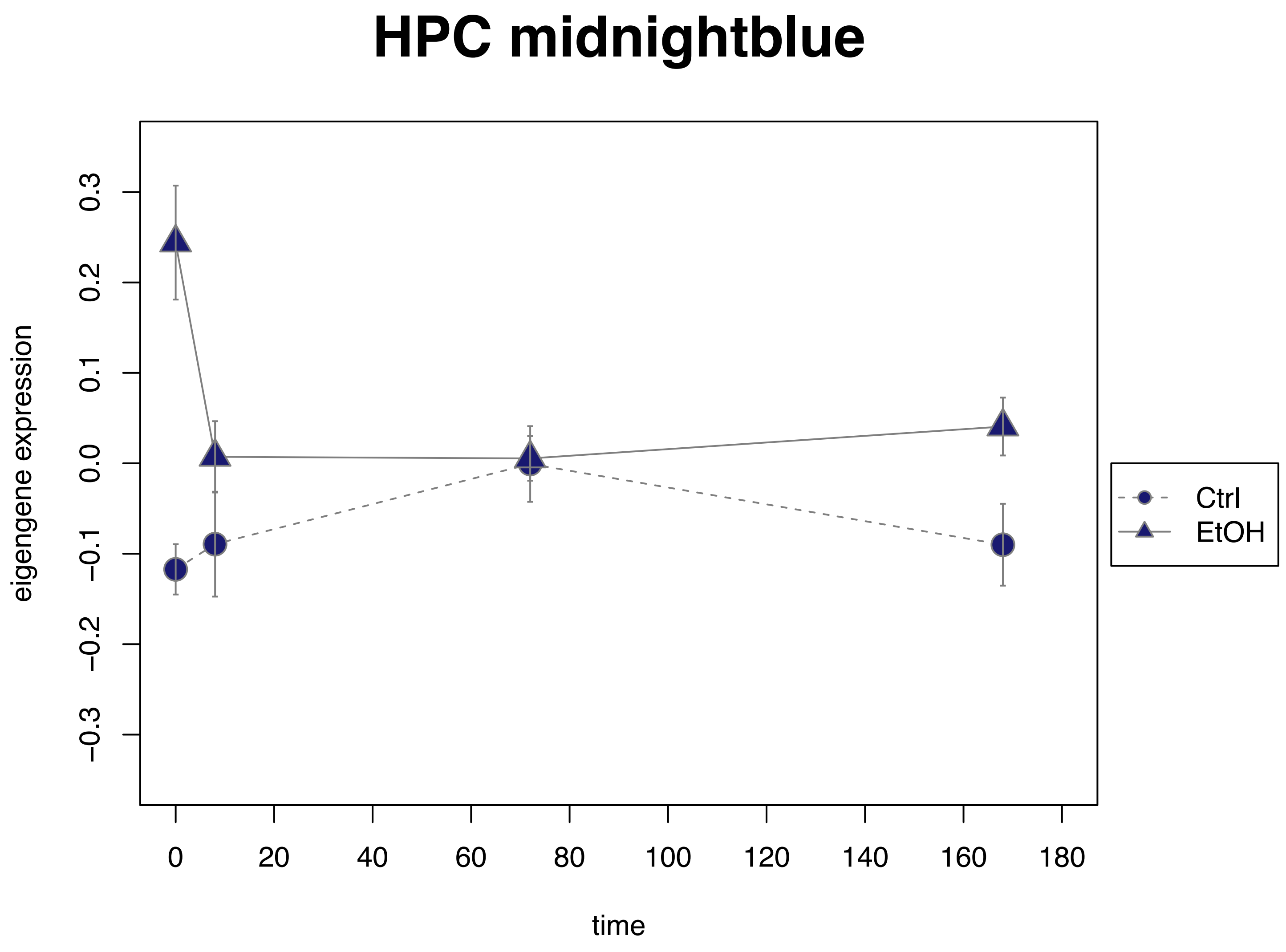
HPC midnightblue
eigengene expression
0.3
0.2
0.1
0.0
−0.1
−0.2
−0.3
0
20
40
60
80
100
120
140
160
180
time
Ctrl
EtOH

HPC orange

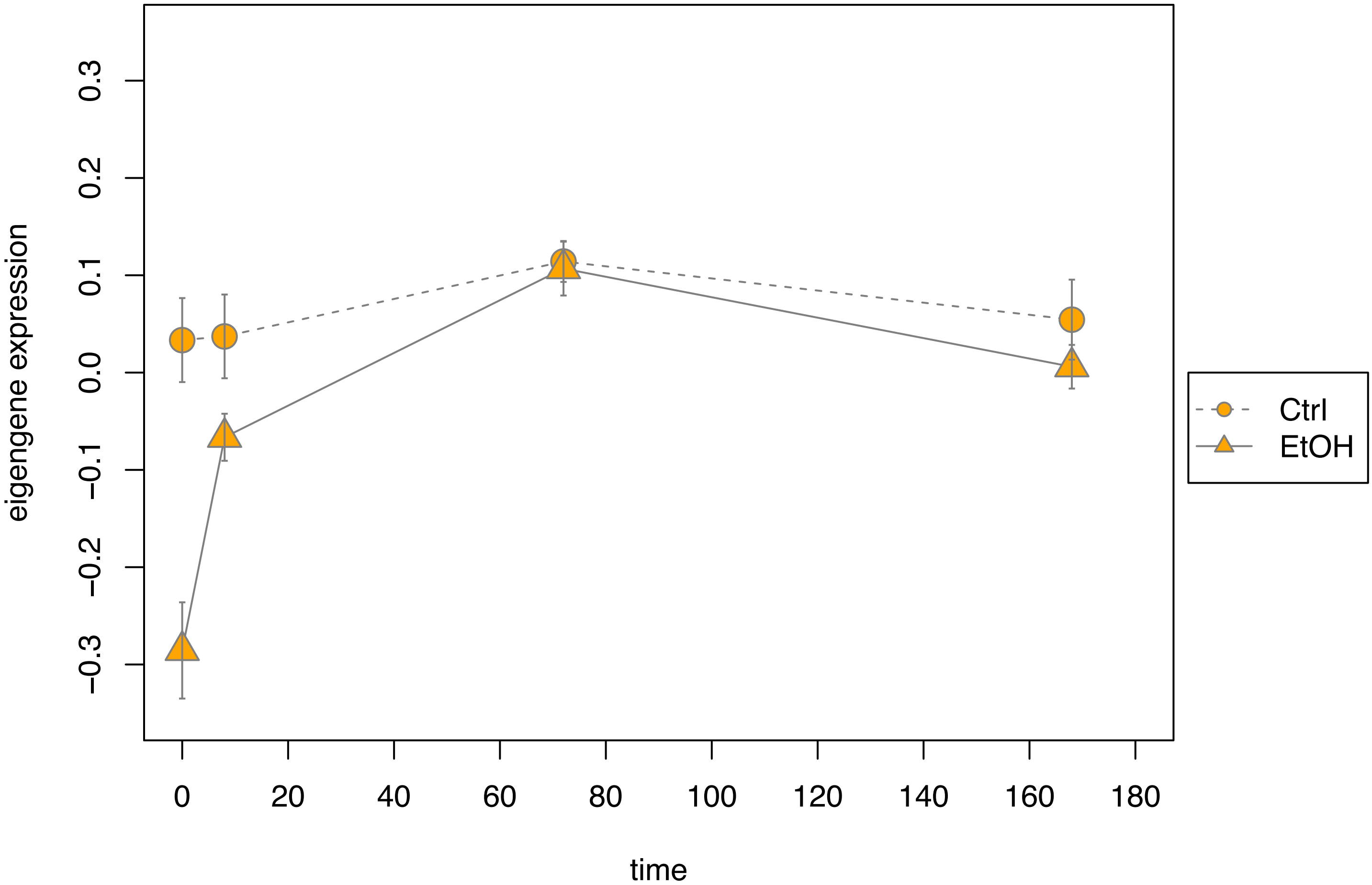

eigengene expression
0.3
0.2
0.1
0.0
−0.1
−0.2
−0.3
0
20
40
60
80
100
120
140
160
180
time
Ctrl
EtOH

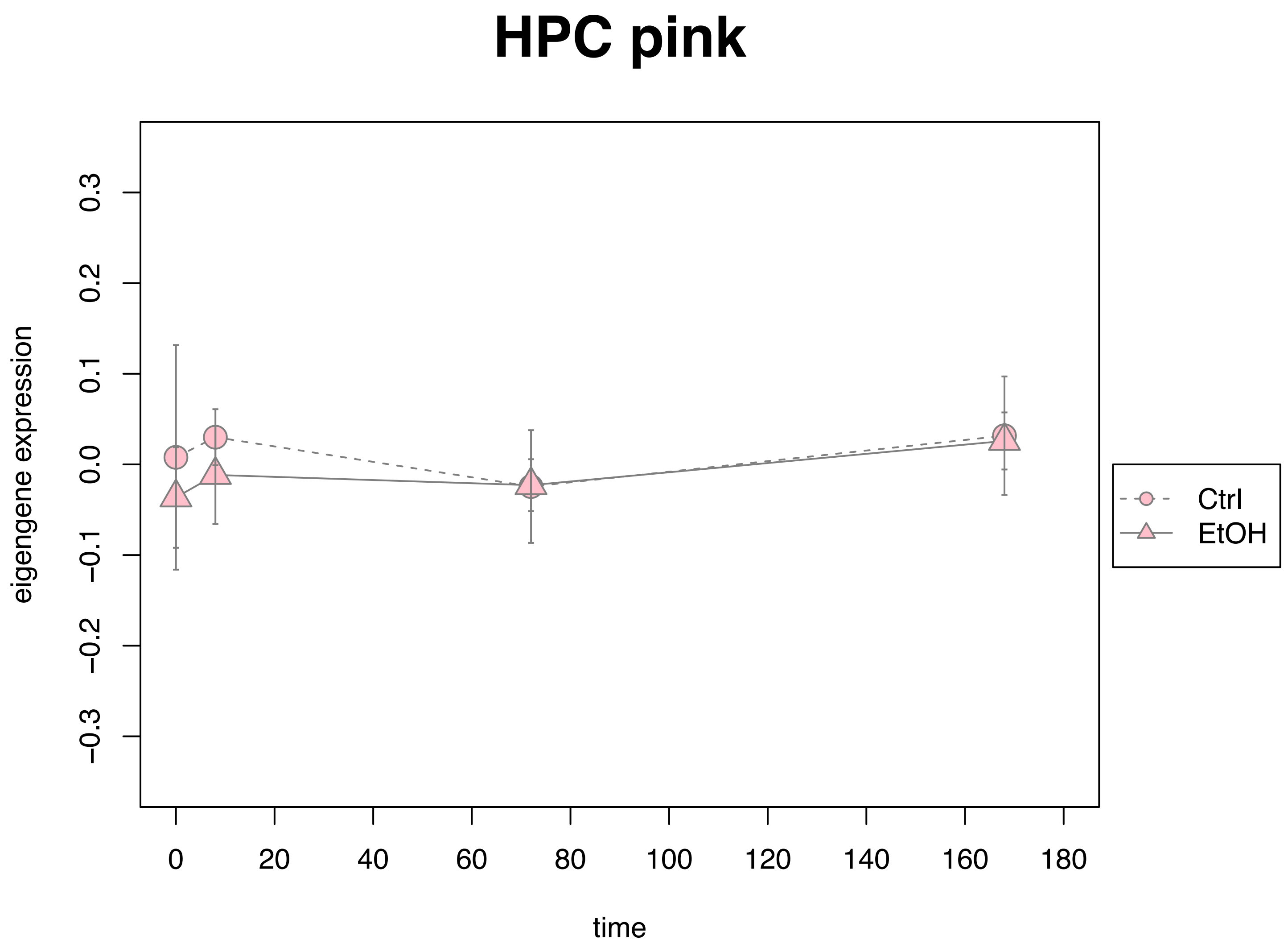

HPC pink
eigengene expression
−0.3
−0.2
−0.1
0.0
0.1
0.2
0.3
0
20
40
60
80
100
120
140
160
180
time
Ctrl
EtOH

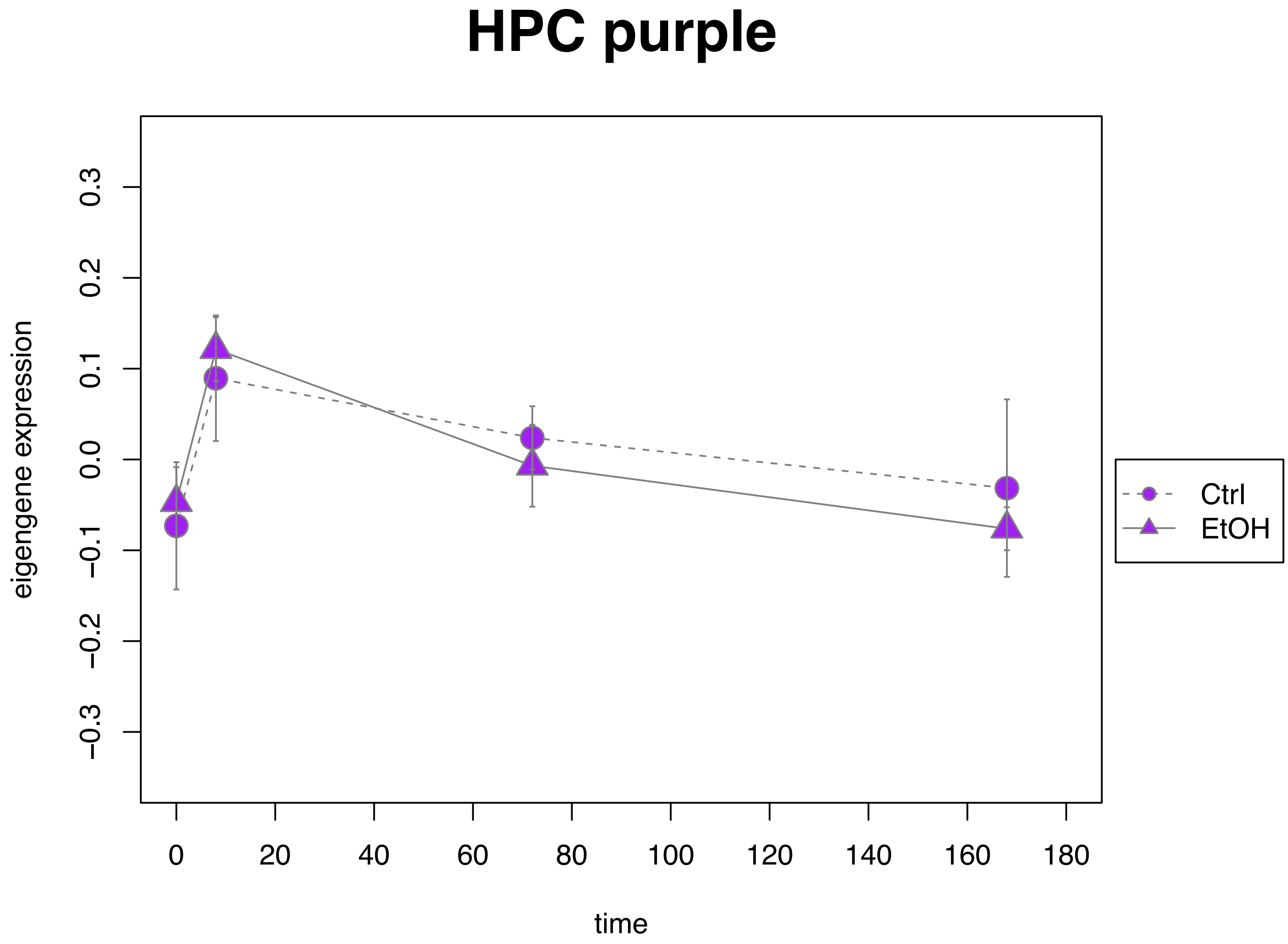
HPC purple
eigengene expression
0.3
0.2
0.1
0.0
−0.1
−0.2
−0.3
0
20
40
60
80
100
120
140
160
180
time
Ctrl
EtOH

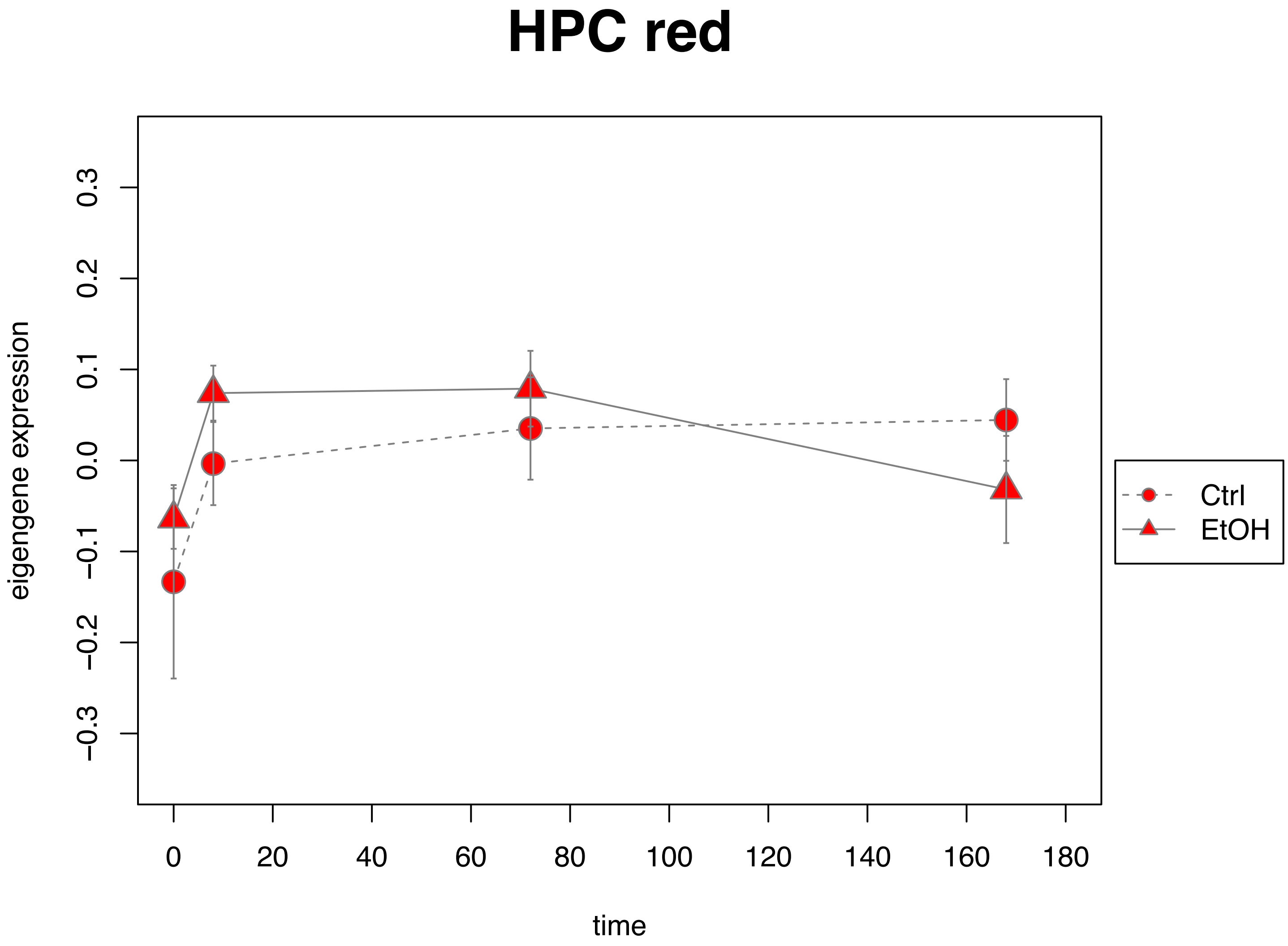

HPC red
eigengene expression
0.3
0.2
0.1
0.0
−0.1
−0.2
−0.3
0
20
40
60
80
100
120
140
160
180
time
Ctrl
EtOH

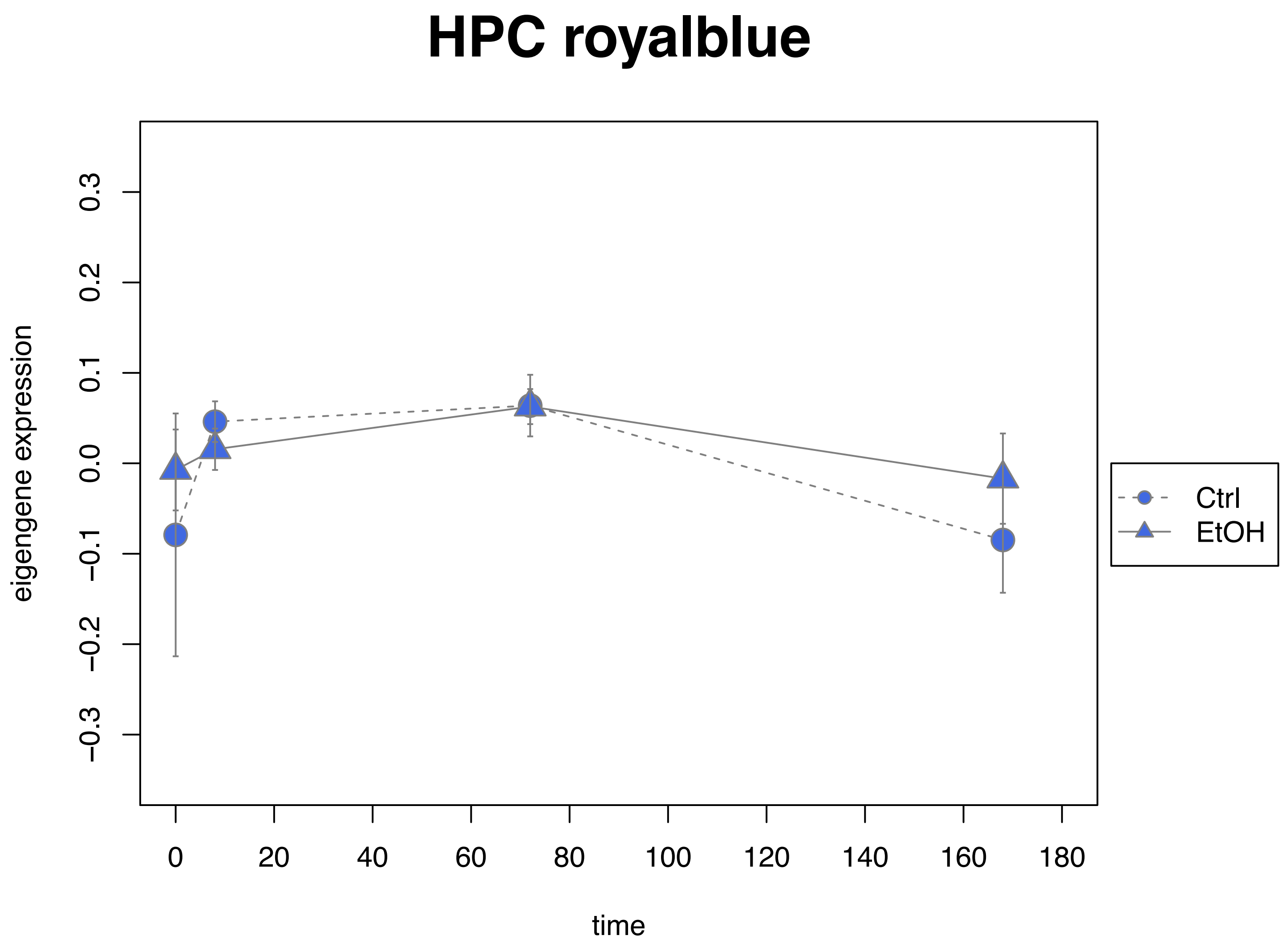

HPC royalblue
eigengene expression
0.3
0.2
0.1
0.0
−0.1
−0.2
−0.3
0
20
40
60
80
100
120
140
160
180
time
Ctrl
EtOH

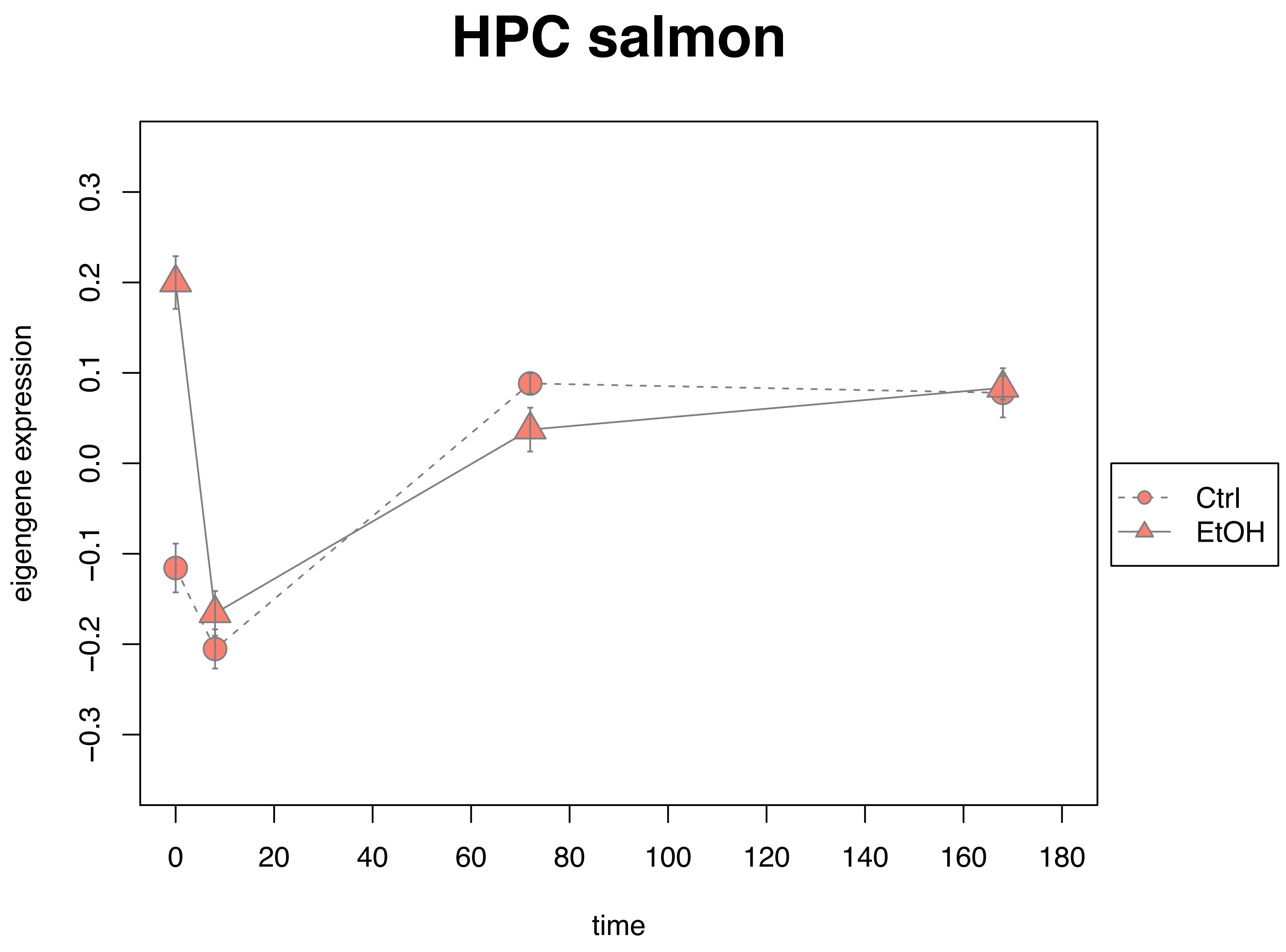
HPC salmon
eigengene expression
time
0.3
0.2
0.1
0.0
−0.1
−0.2
−0.3
0
20
40
60
80
100
120
140
160
180
Ctrl
EtOH

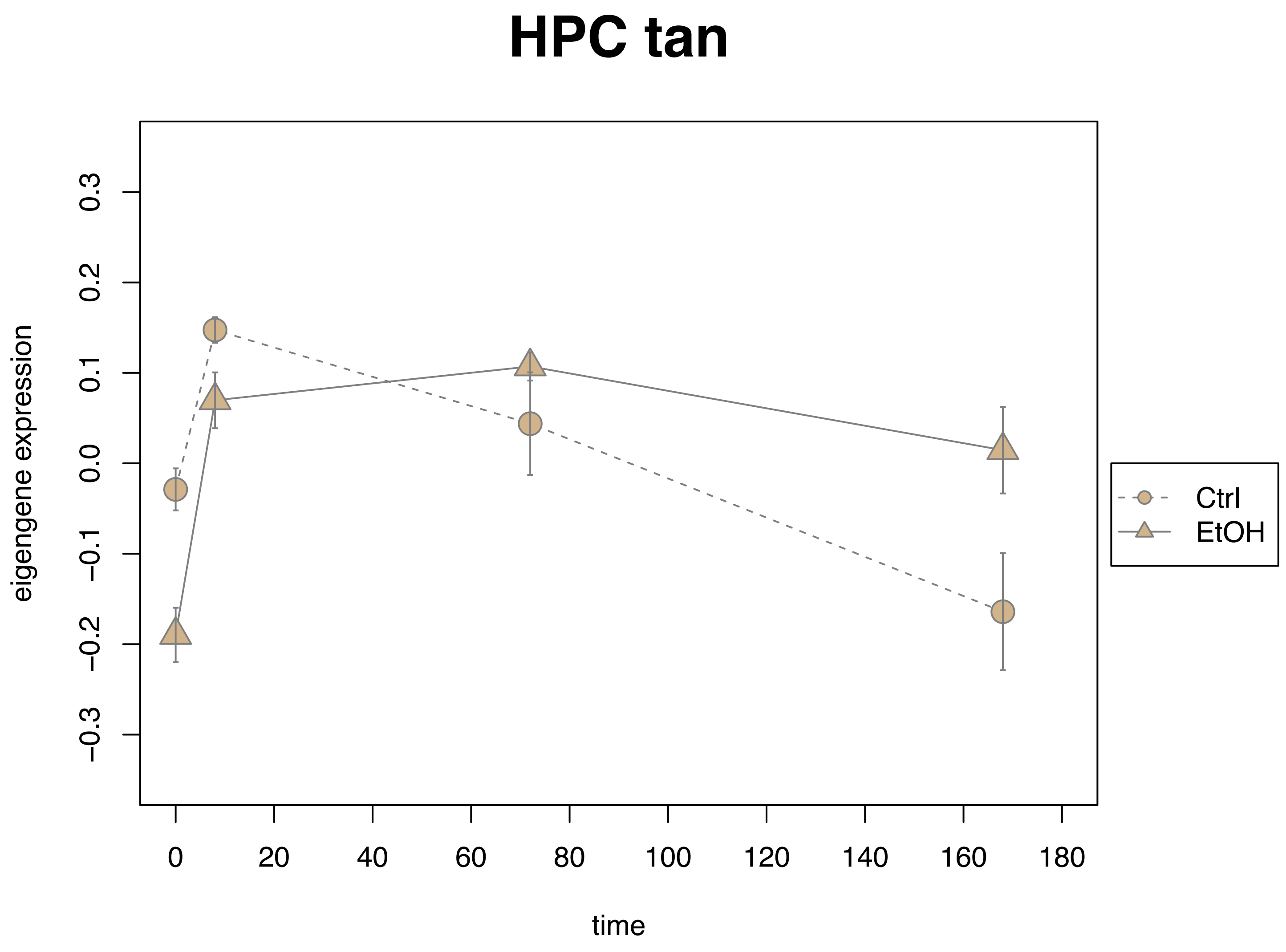
HPC tan
eigengene expression
time
0.3
0.2
0.1
0.0
−0.1
−0.2
−0.3
0
20
40
60
80
100
120
140
160
180
Ctrl
EtOH

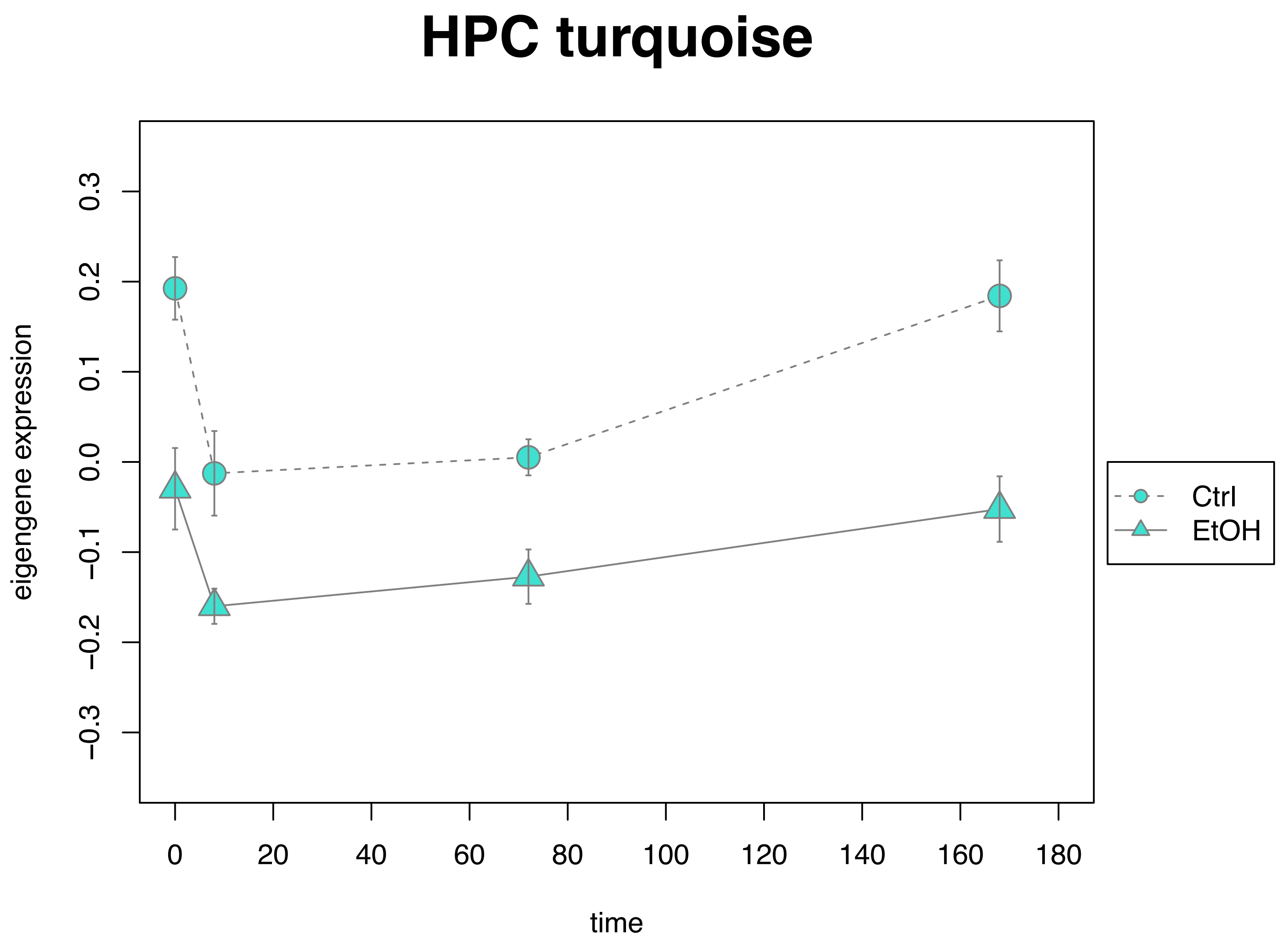

HPC turquoise
eigengene expression
0.3
0.2
0.1
0.0
−0.1
−0.2
−0.3
0
20
40
60
80
100
120
140
160
180
time
Ctrl
EtOH

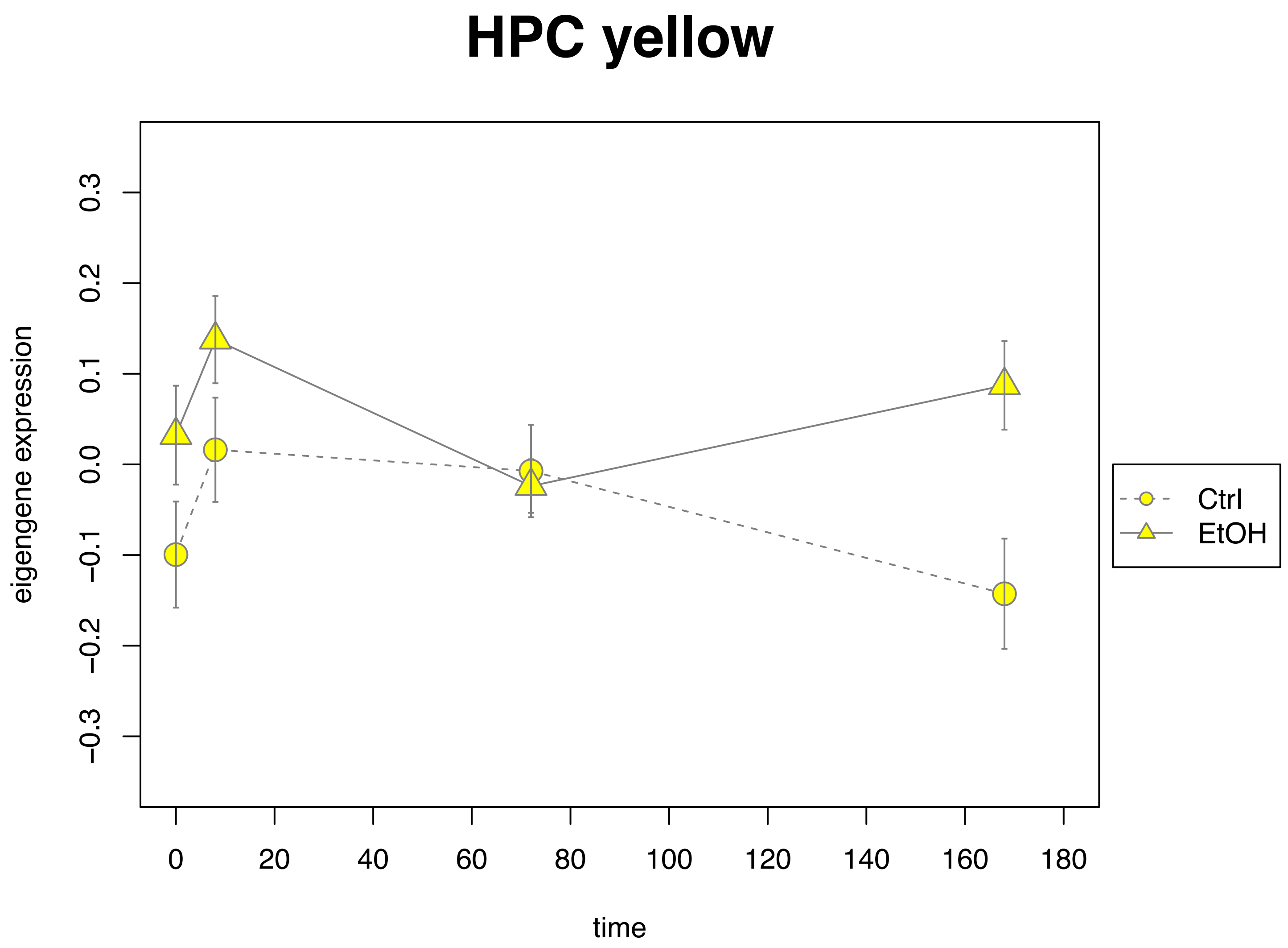

HPC yellow
eigengene expression
0.3
0.2
0.1
0.0
−0.1
−0.2
−0.3
0
20
40
60
80
100
120
140
160
180
time
Ctrl
EtOH
