# Supplementary material for: Time-Course Analysis of Brain Regional Expression Network Responses to Chronic Intermittent Ethanol and Withdrawal: Implications for Mechanisms Underlying Excessive Ethanol Consumption
Source: PLoS One. 2016 Jan 5;11(1):e0146257. doi: 10.1371/journal.pone.0146257 (PMC4701666; doi:10.1371/journal.pone.0146257)
Supplement: S4 Fig — (PDF) [file pone.0146257.s004.pdf]

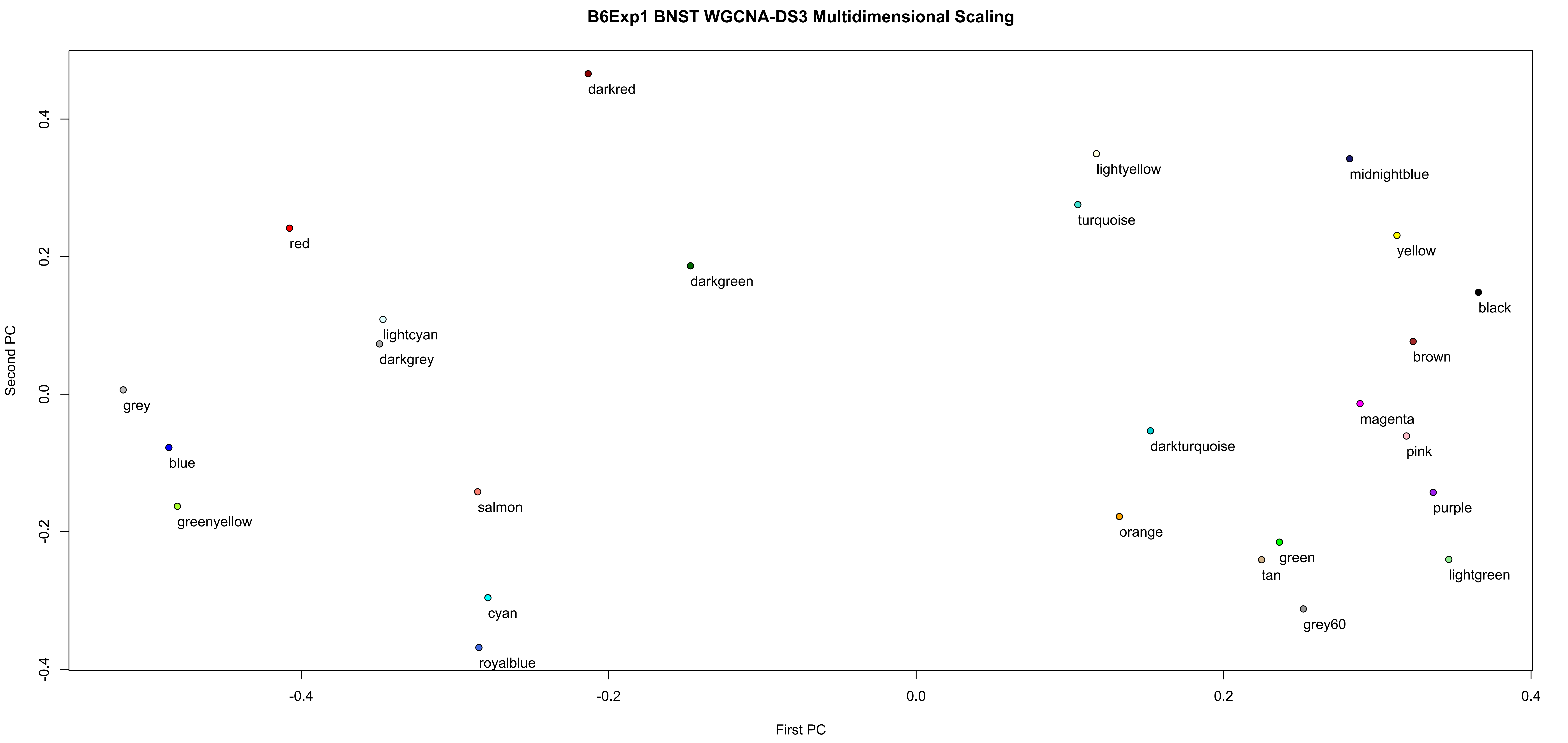
B6Exp1 BNST WGCNA-DS3 Multidimensional Scaling
First PC
Second PC
-0.4
-0.2
0.0
0.2
0.4
darkred
lightyellow
midnightblue
turquoise
yellow
red
darkgreen
black
lightcyan
darkgrey
brown
grey
magenta
darkturquoise
pink
blue
salmon
purple
greenyellow
orange
green
tan
lightgreen
cyan
grey60
royalblue

B6Exp1 BNST WGCNA-DS3 Module Eigengene Cluster Dendrogram

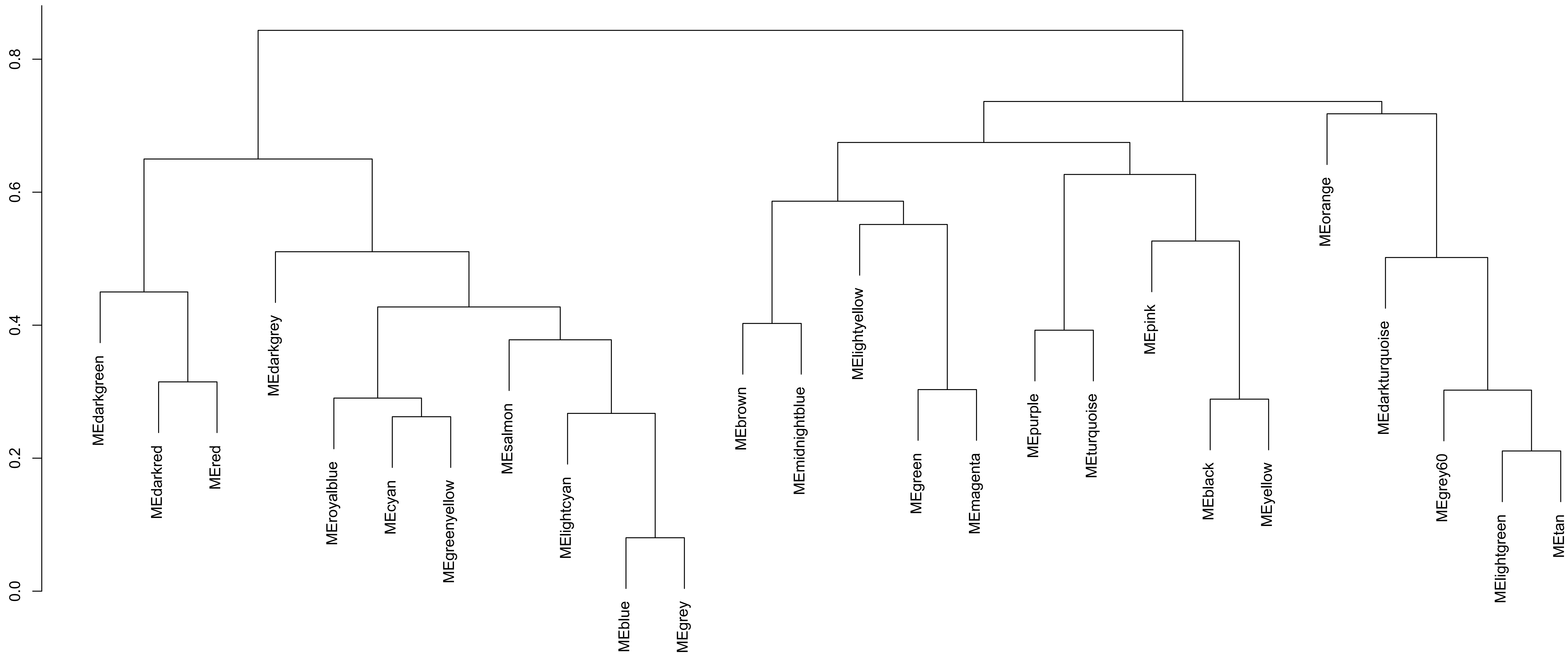

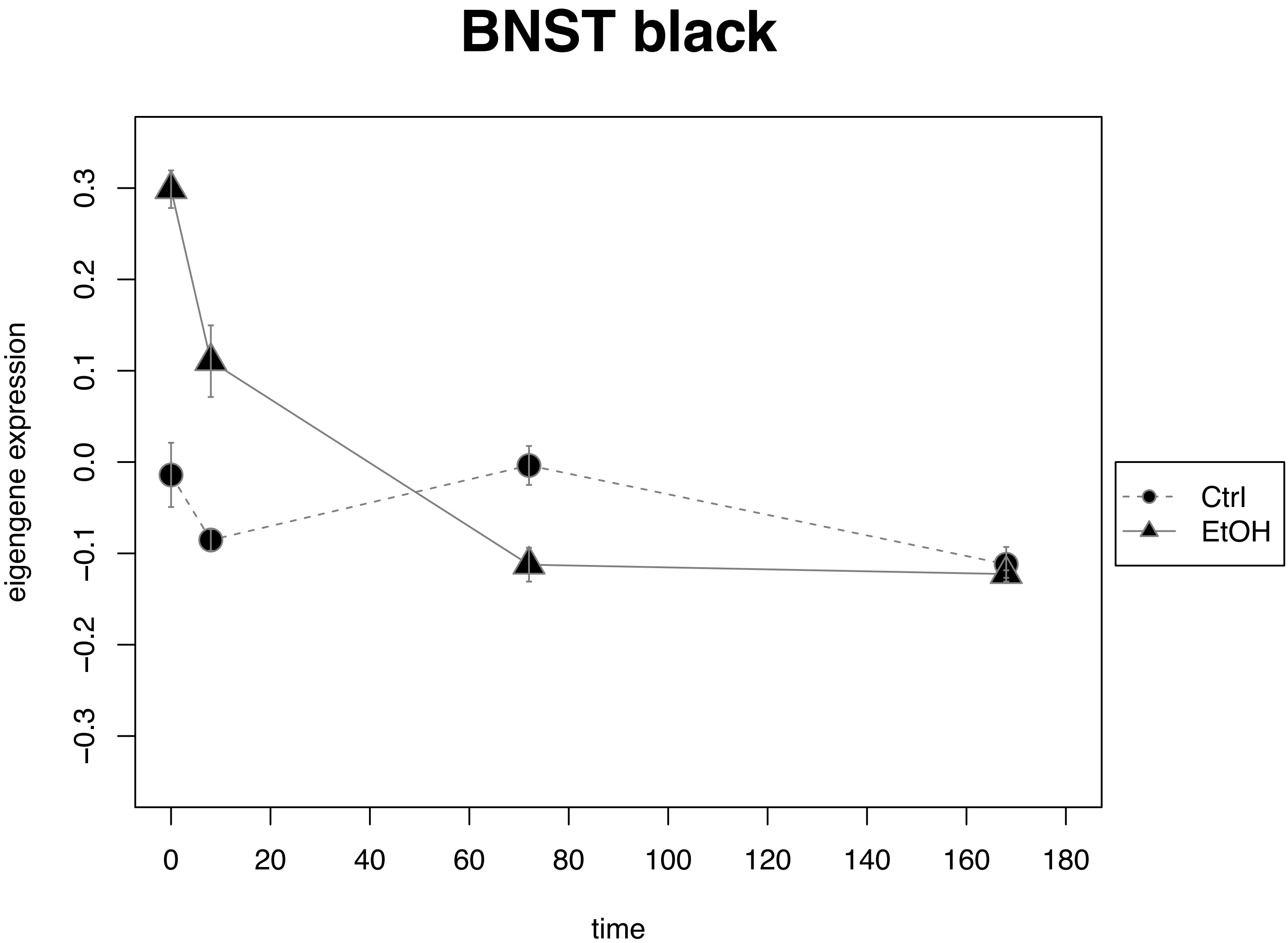
BNST black
eigengene expression
0.3
0.2
0.1
0.0
−0.1
−0.2
−0.3
0
20
40
60
80
100
120
140
160
180
time
Ctrl
EtOH

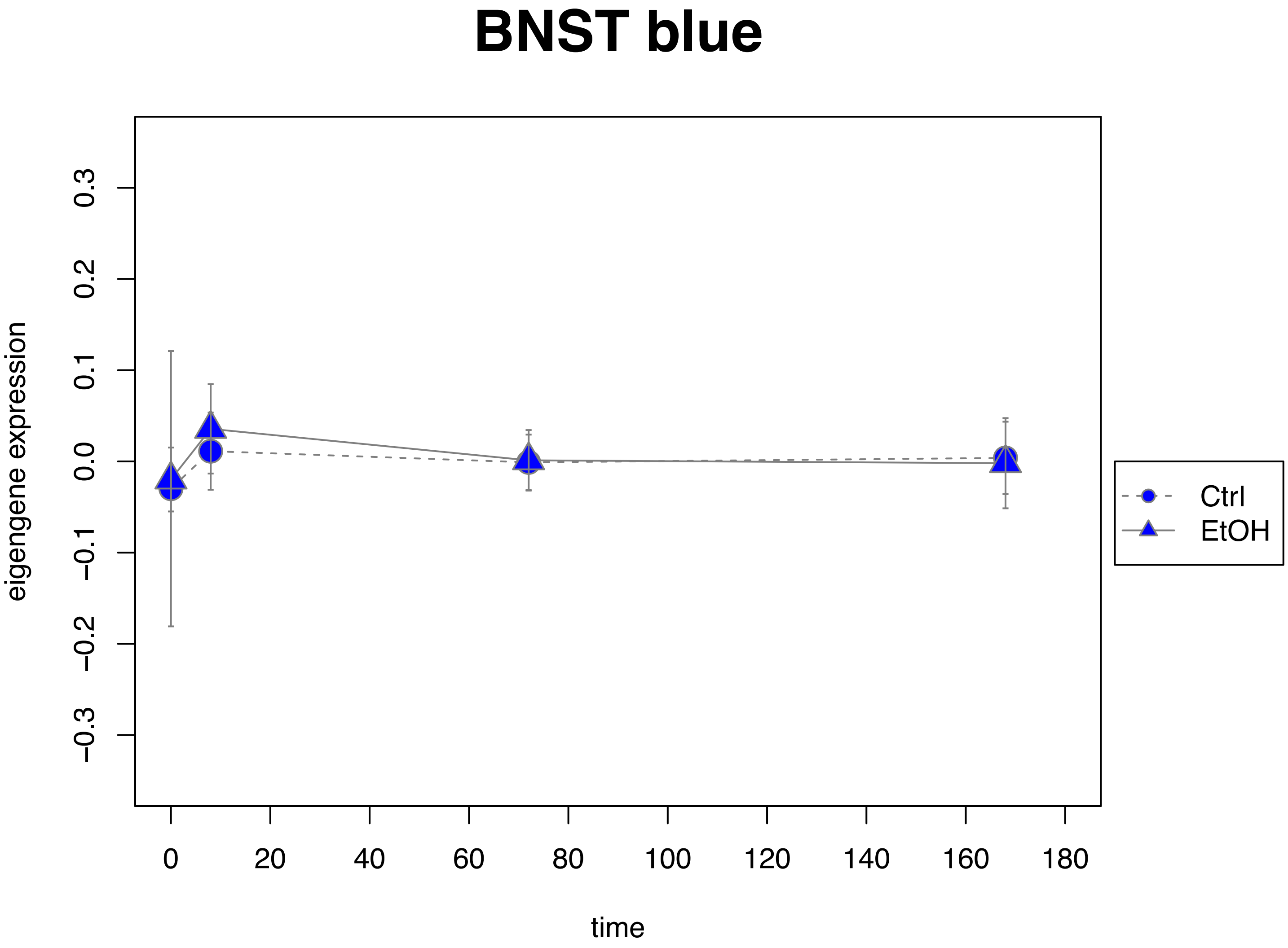
BNST blue
eigengene expression
0.3
0.2
0.1
0.0
−0.1
−0.2
−0.3
0
20
40
60
80
100
120
140
160
180
time
Ctrl
EtOH

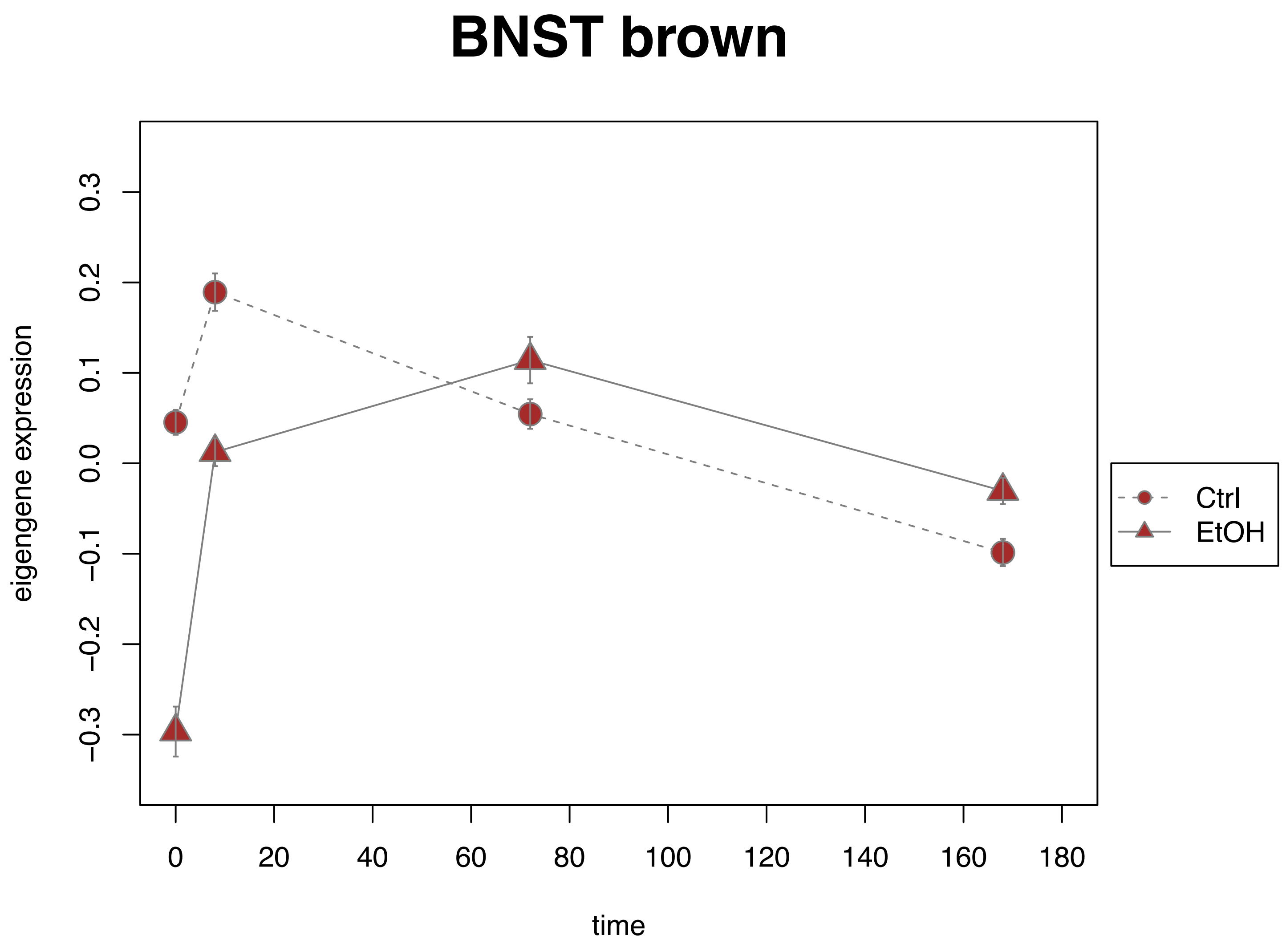
BNST brown
eigengene expression
0.3
0.2
0.1
0.0
−0.1
−0.2
−0.3
0
20
40
60
80
100
120
140
160
180
time
Ctrl
EtOH

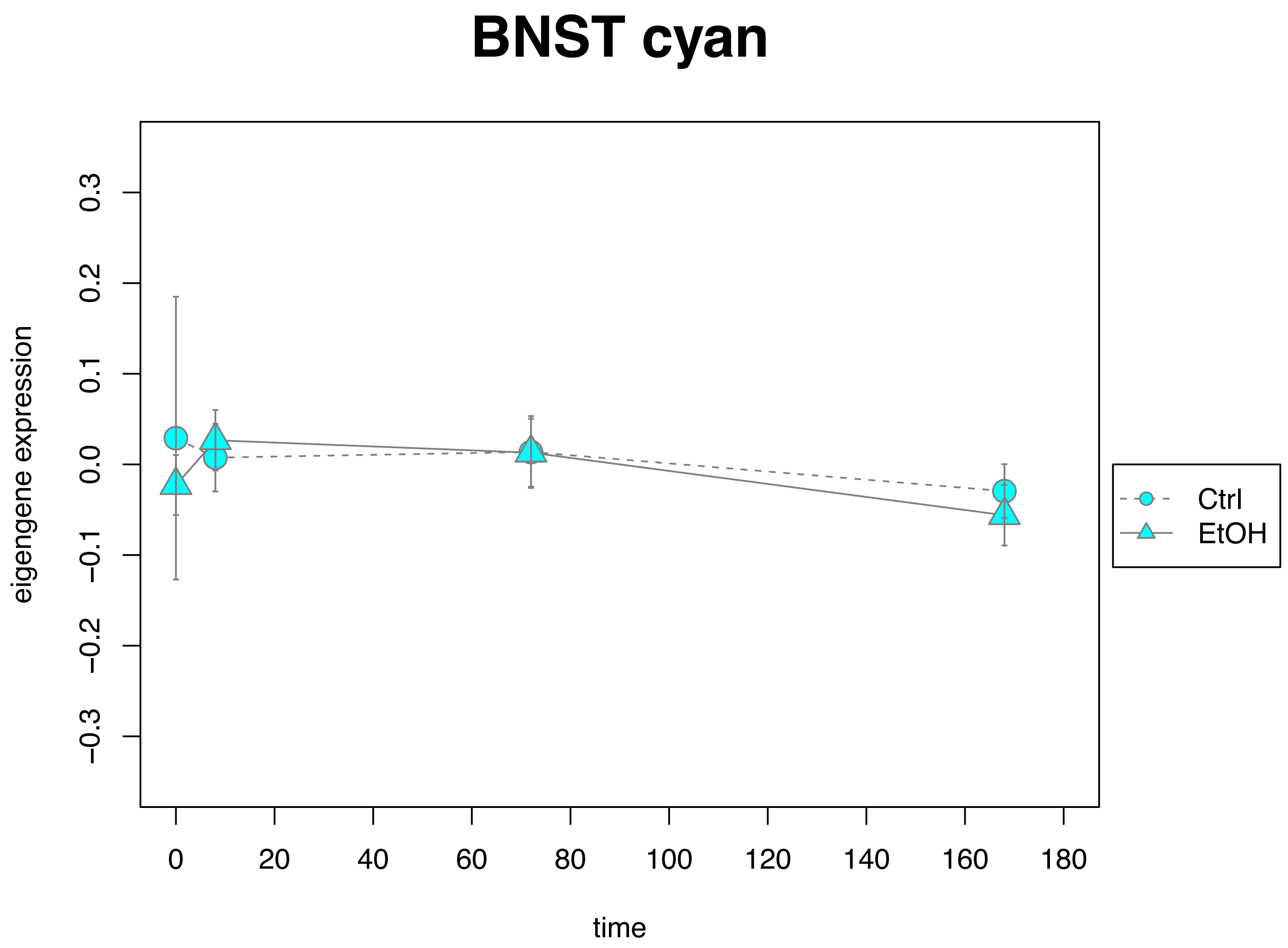
BNST cyan
eigengene expression
0.3
0.2
0.1
0.0
−0.1
−0.2
−0.3
0
20
40
60
80
100
120
140
160
180
time
Ctrl
EtOH

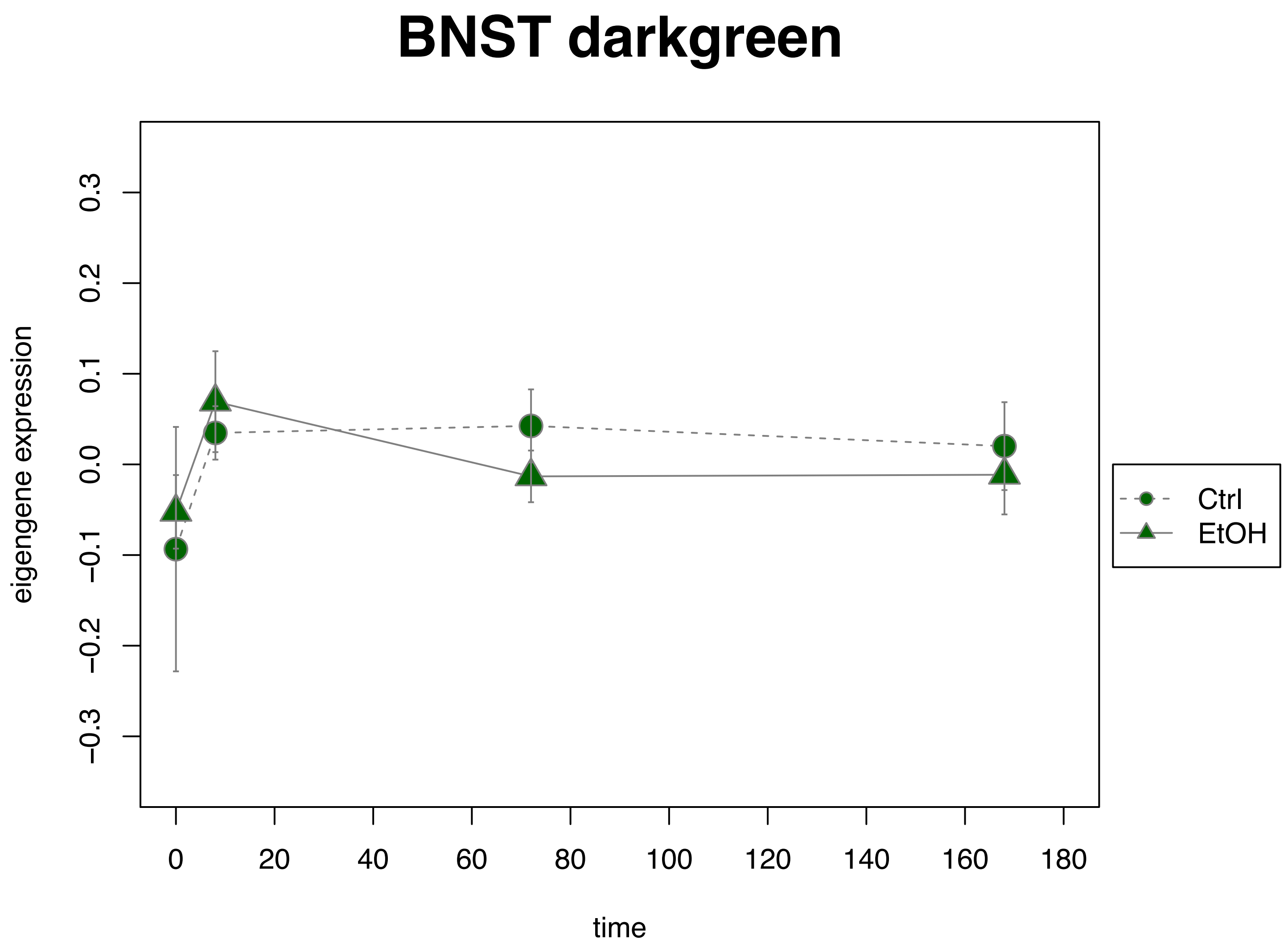
BNST darkgreen
eigengene expression
0.3
0.2
0.1
0.0
−0.1
−0.2
−0.3
time
0
20
40
60
80
100
120
140
160
180
Ctrl
EtOH

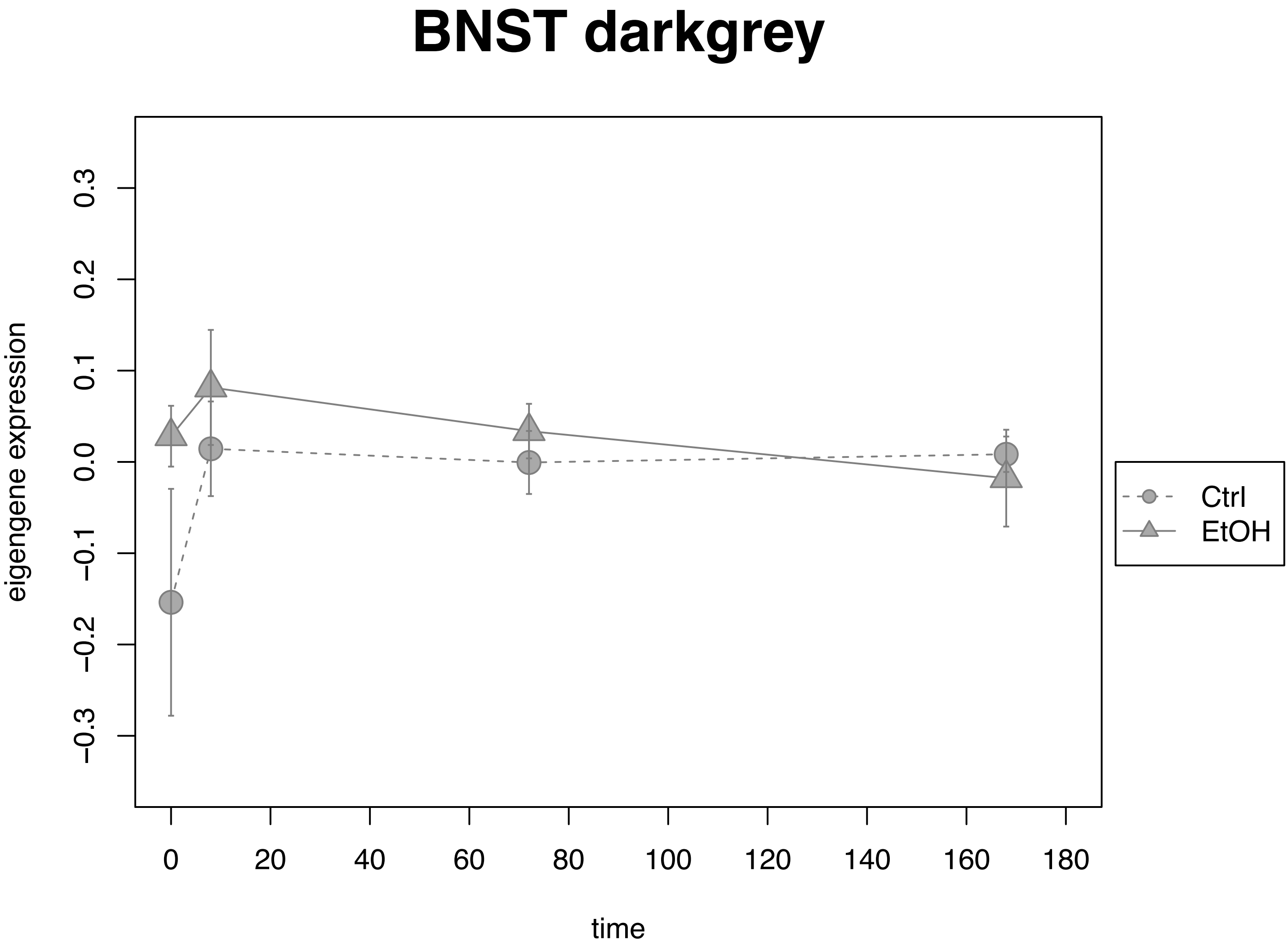
BNST darkgrey
eigengene expression
0.3
0.2
0.1
0.0
−0.1
−0.2
−0.3
time
0
20
40
60
80
100
120
140
160
180
Ctrl
EtOH

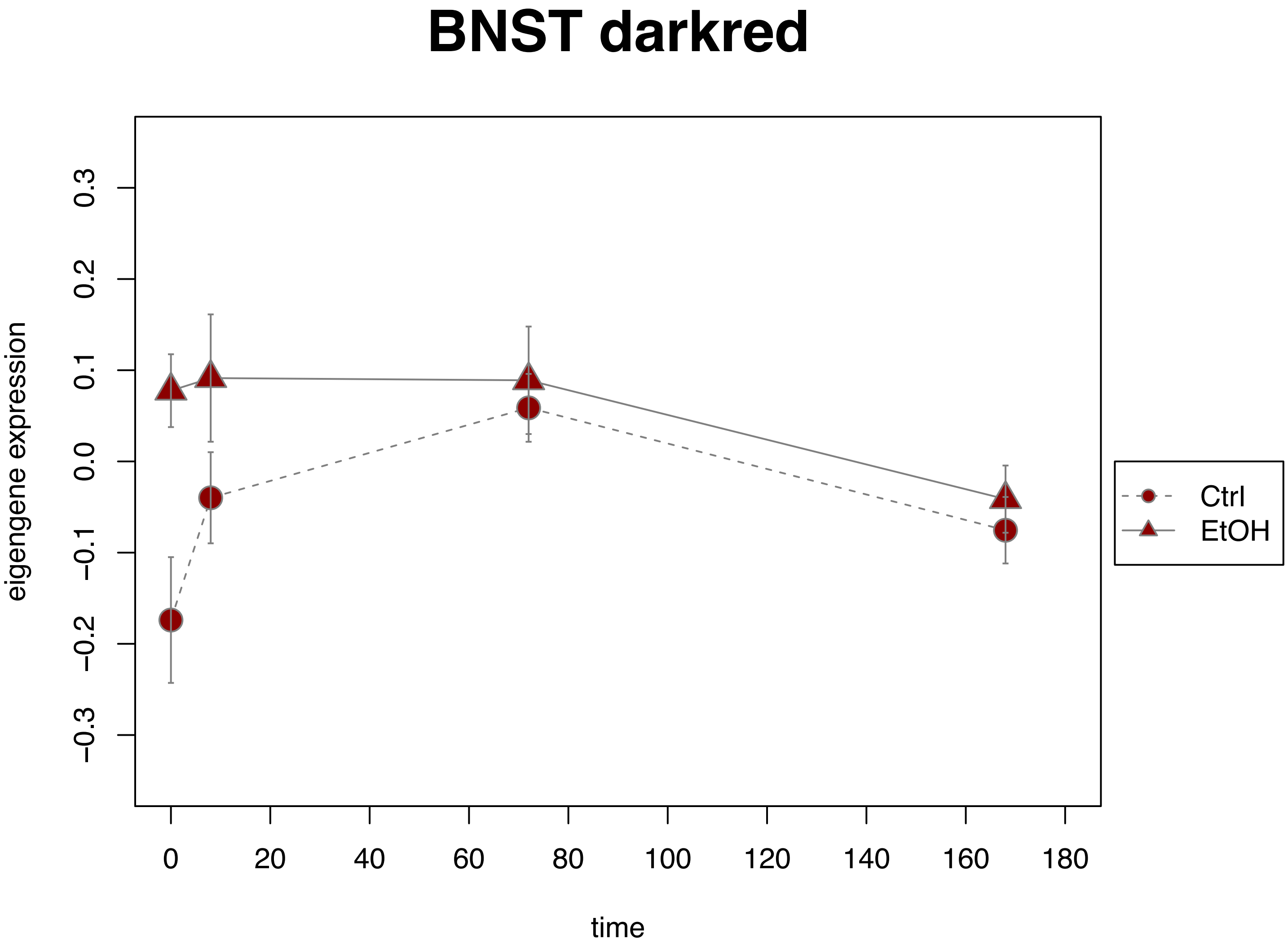

BNST darkred
eigengene expression
0.3
0.2
0.1
0.0
−0.1
−0.2
−0.3
0
20
40
60
80
100
120
140
160
180
time
Ctrl
EtOH

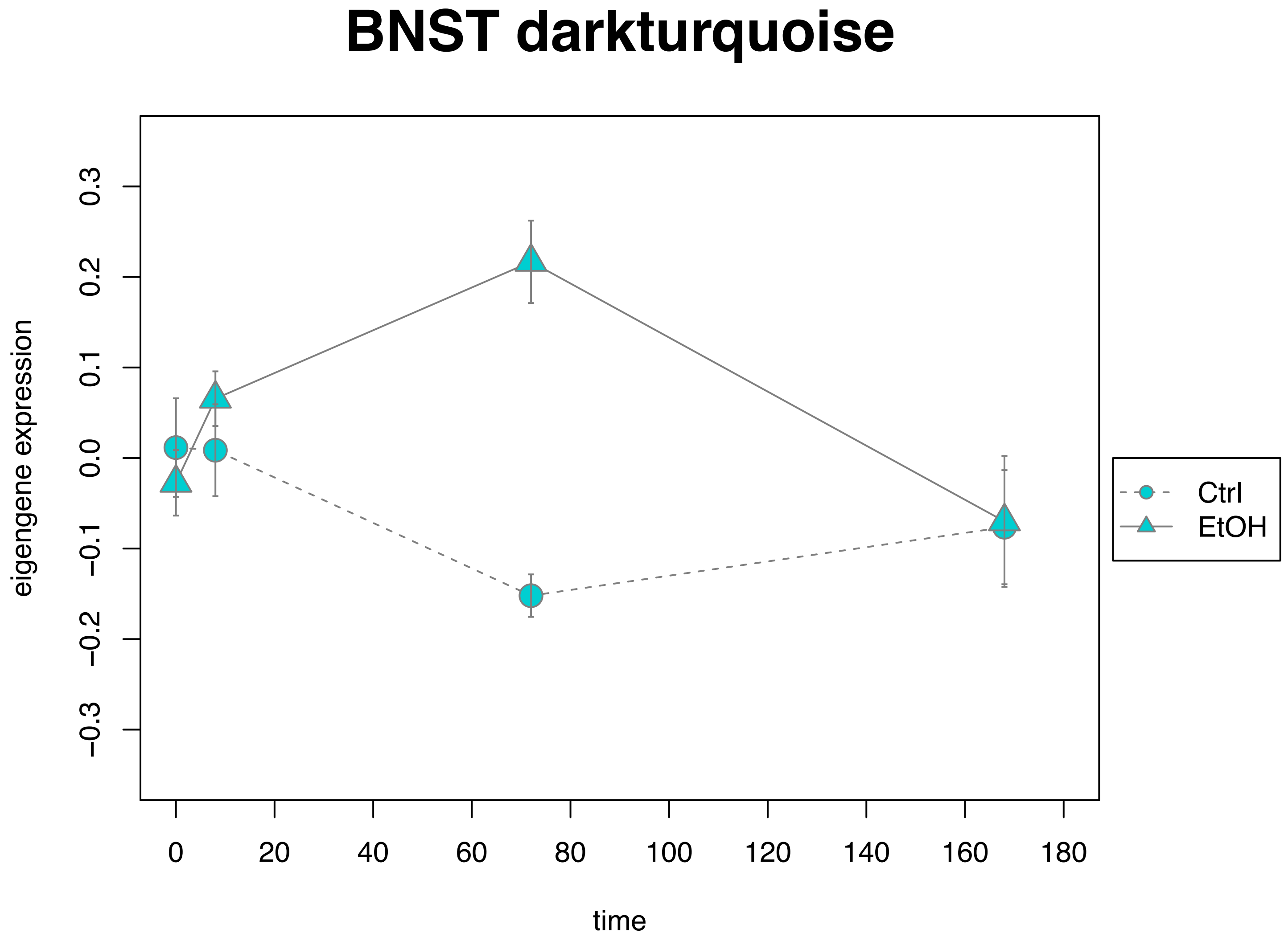
BNST darkturquoise
eigengene expression
0.3
0.2
0.1
0.0
−0.1
−0.2
−0.3
0
20
40
60
80
100
120
140
160
180
time
Ctrl
EtOH

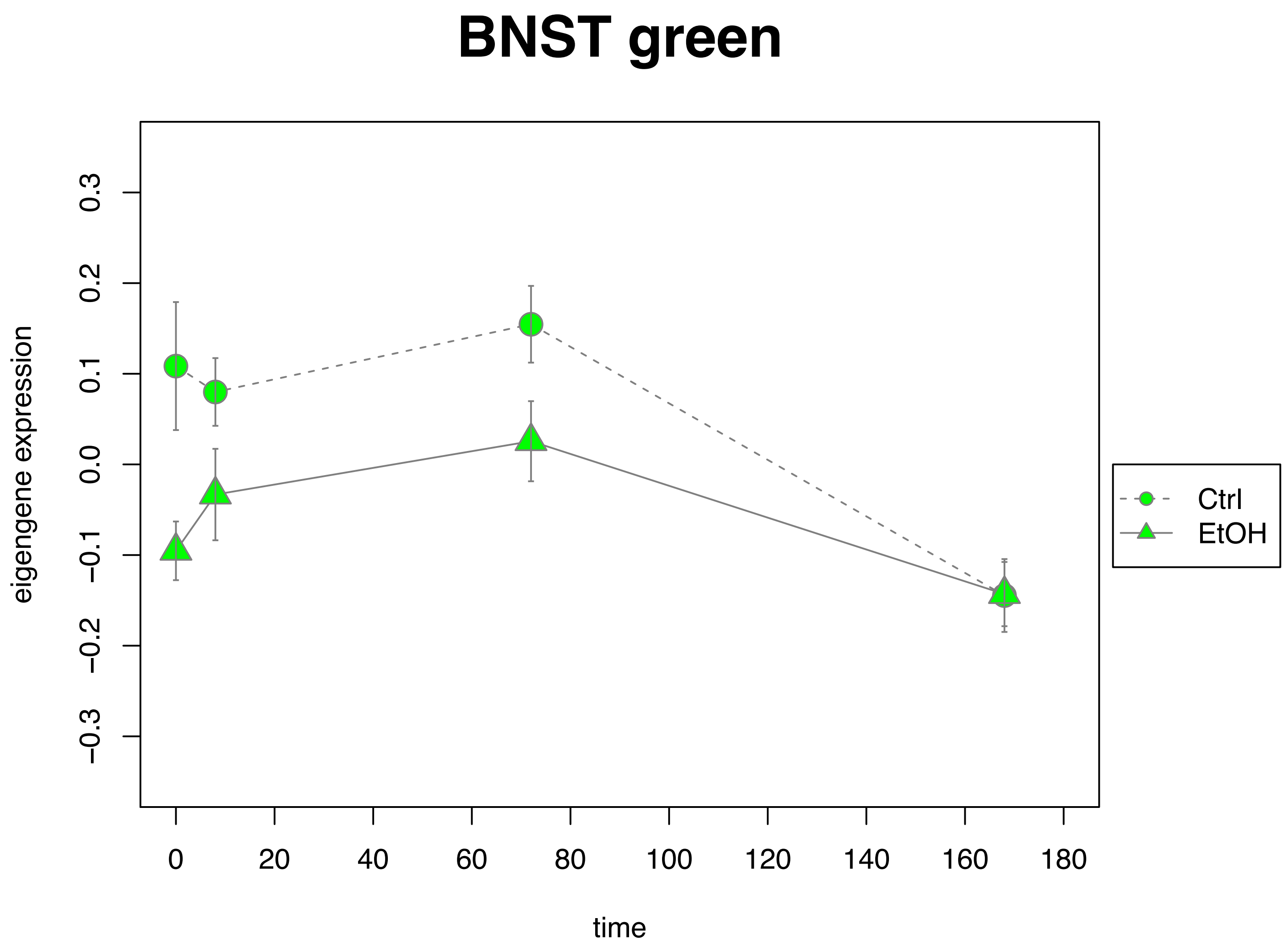

BNST green
eigengene expression
0.3
0.2
0.1
0.0
−0.1
−0.2
−0.3
0
20
40
60
80
100
120
140
160
180
time
Ctrl
EtOH

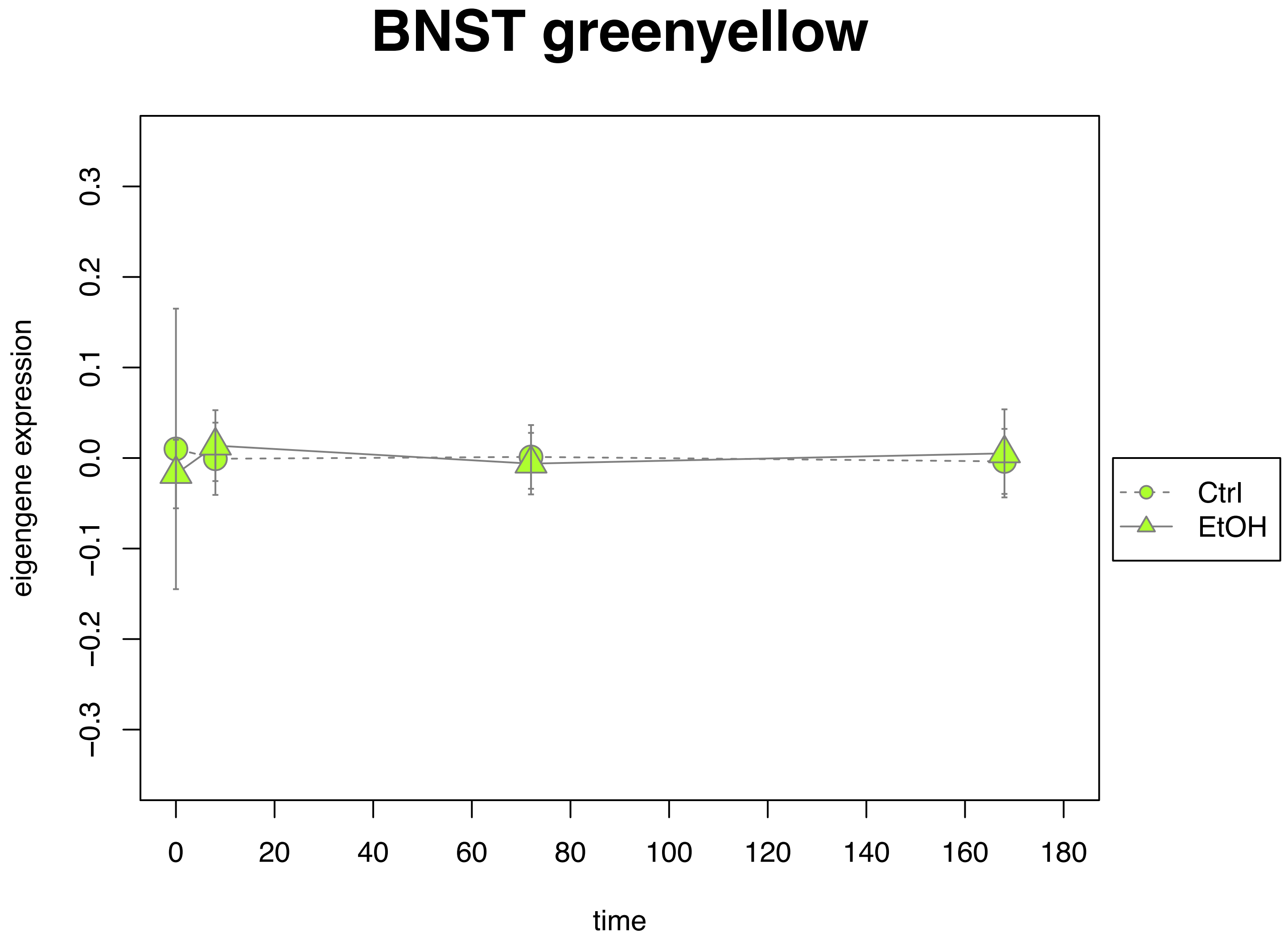

BNST greenyellow
eigengene expression
0.3
0.2
0.1
0.0
−0.1
−0.2
−0.3
time
0
20
40
60
80
100
120
140
160
180
Ctrl
EtOH

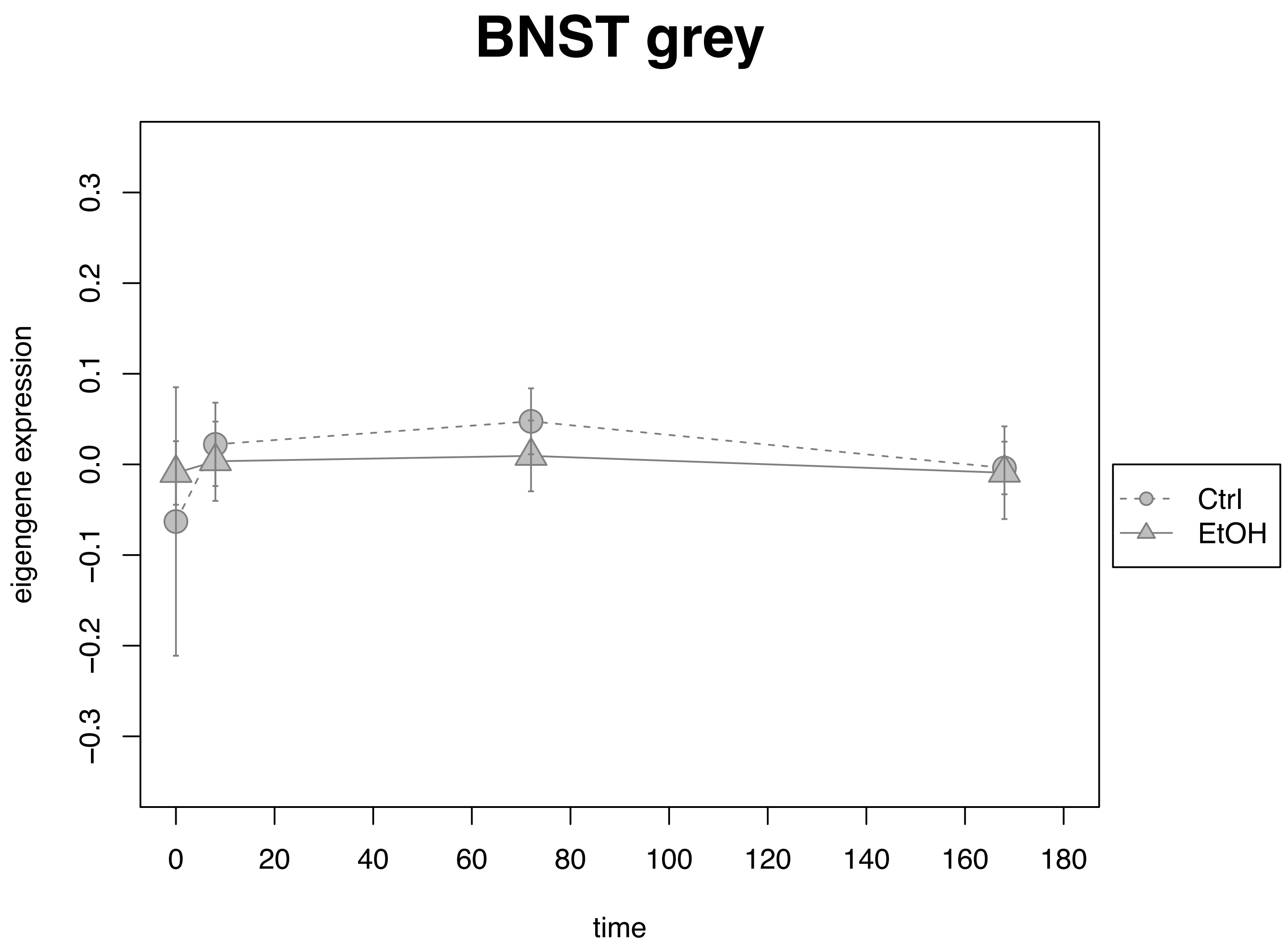
BNST grey
eigengene expression
0.3
0.2
0.1
0.0
−0.1
−0.2
−0.3
0
20
40
60
80
100
120
140
160
180
time
Ctrl
EtOH

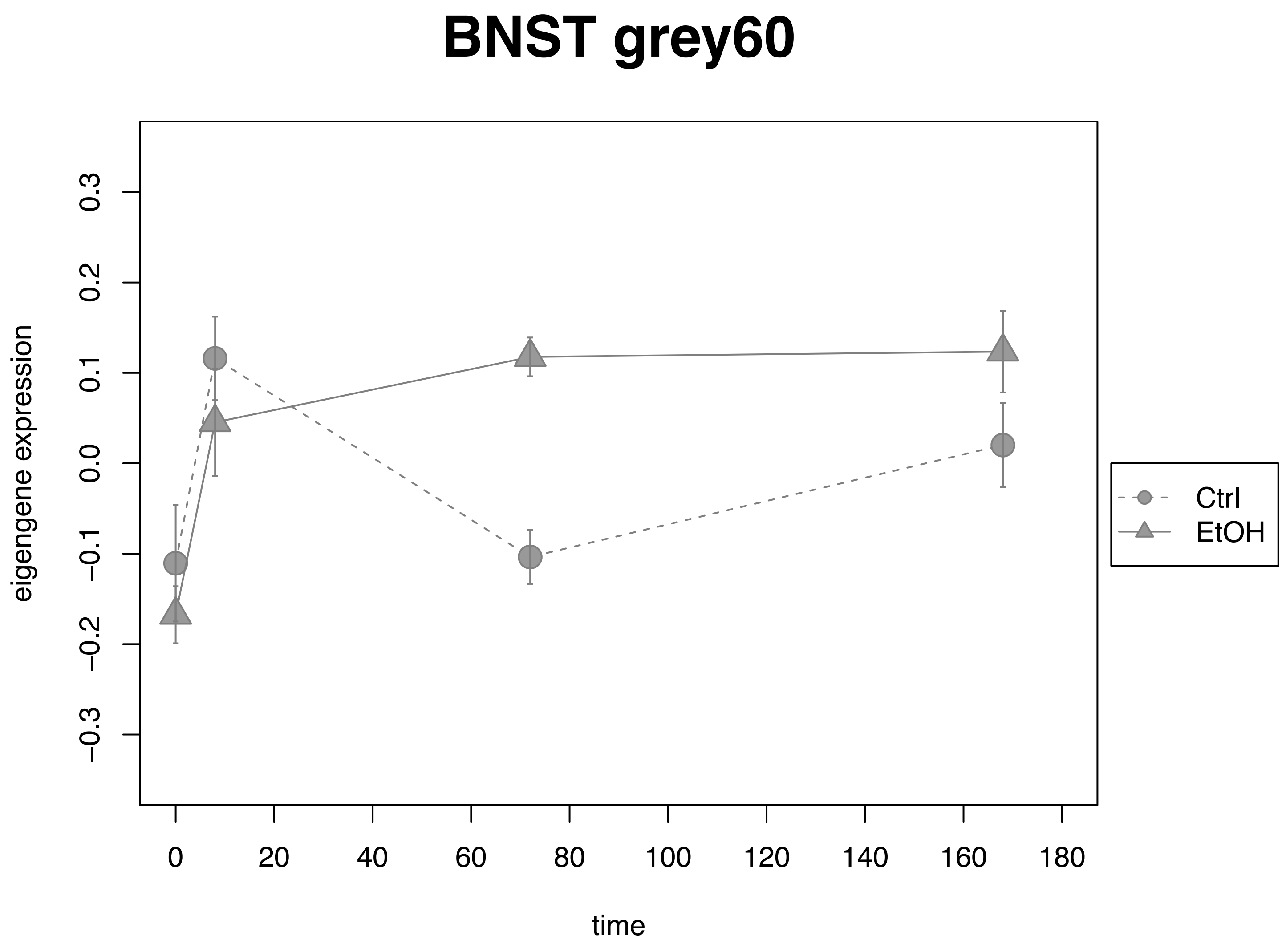

BNST grey60
eigengene expression
time
Ctrl
EtOH
0.3
0.2
0.1
0.0
−0.1
−0.2
−0.3
0
20
40
60
80
100
120
140
160
180

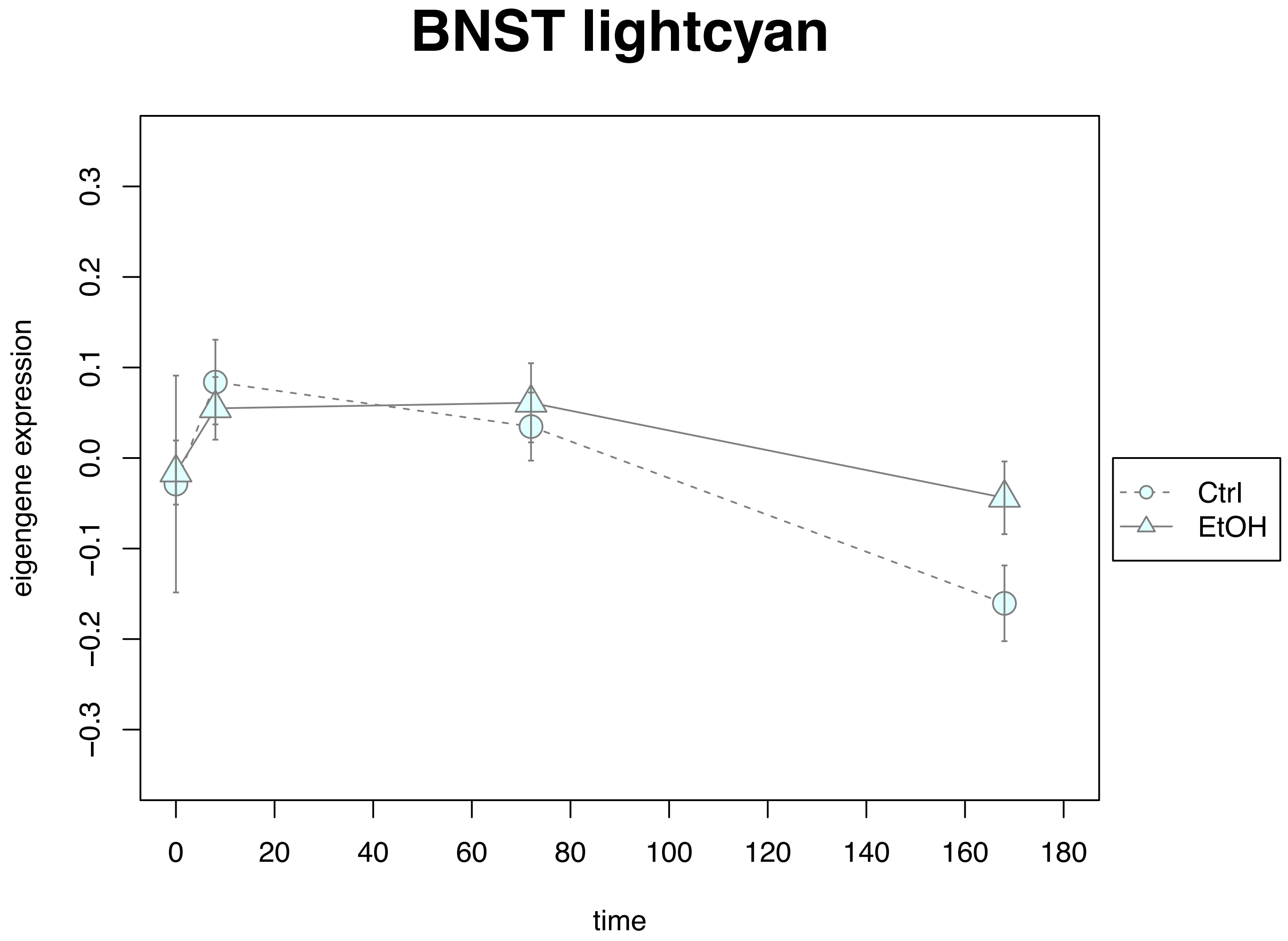
BNST lightcyan
eigengene expression
time
0.3
0.2
0.1
0.0
−0.1
−0.2
−0.3
0
20
40
60
80
100
120
140
160
180
Ctrl
EtOH

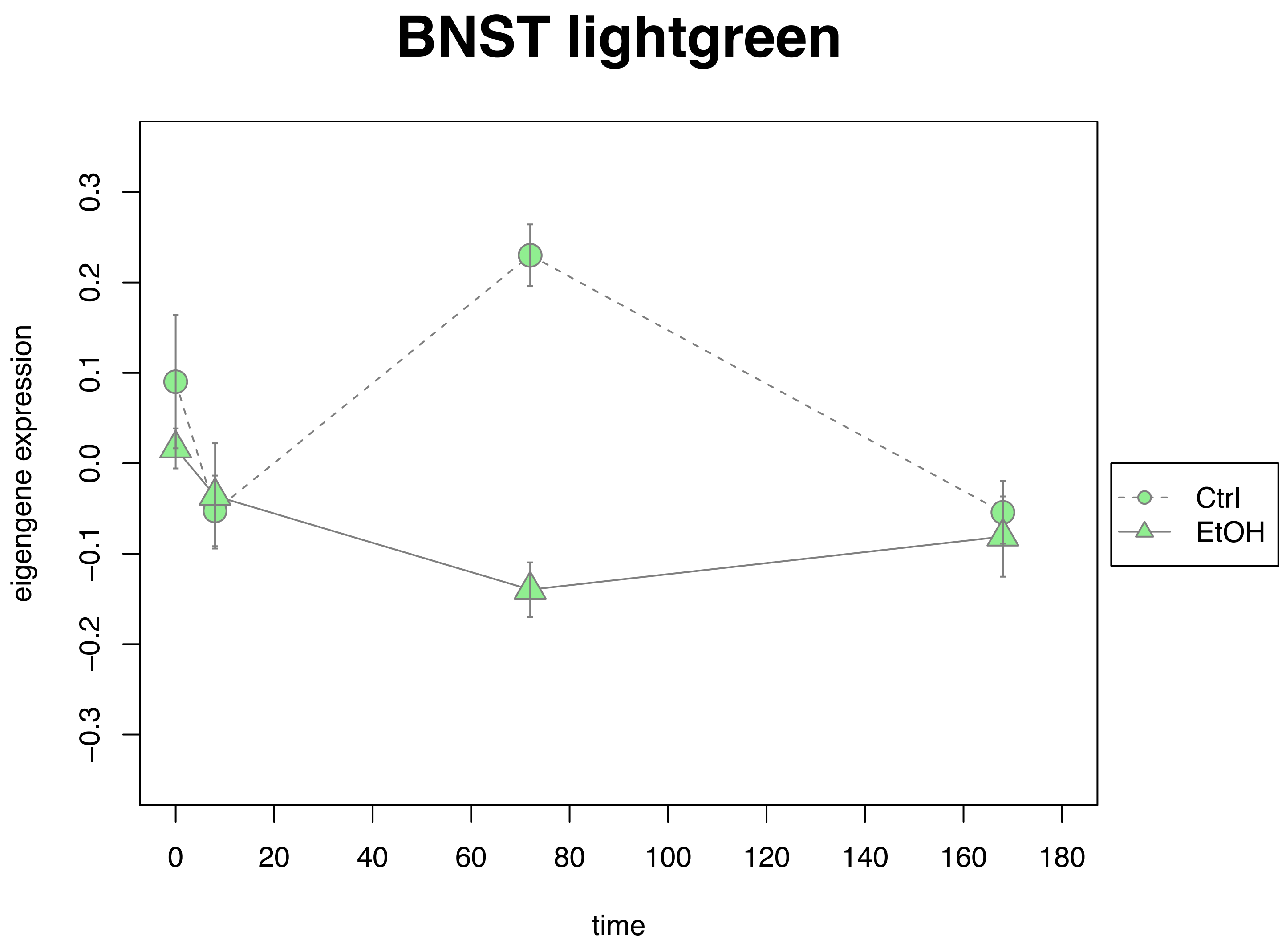

BNST lightgreen
eigengene expression
0.3
0.2
0.1
0.0
−0.1
−0.2
−0.3
0
20
40
60
80
100
120
140
160
180
time
Ctrl
EtOH

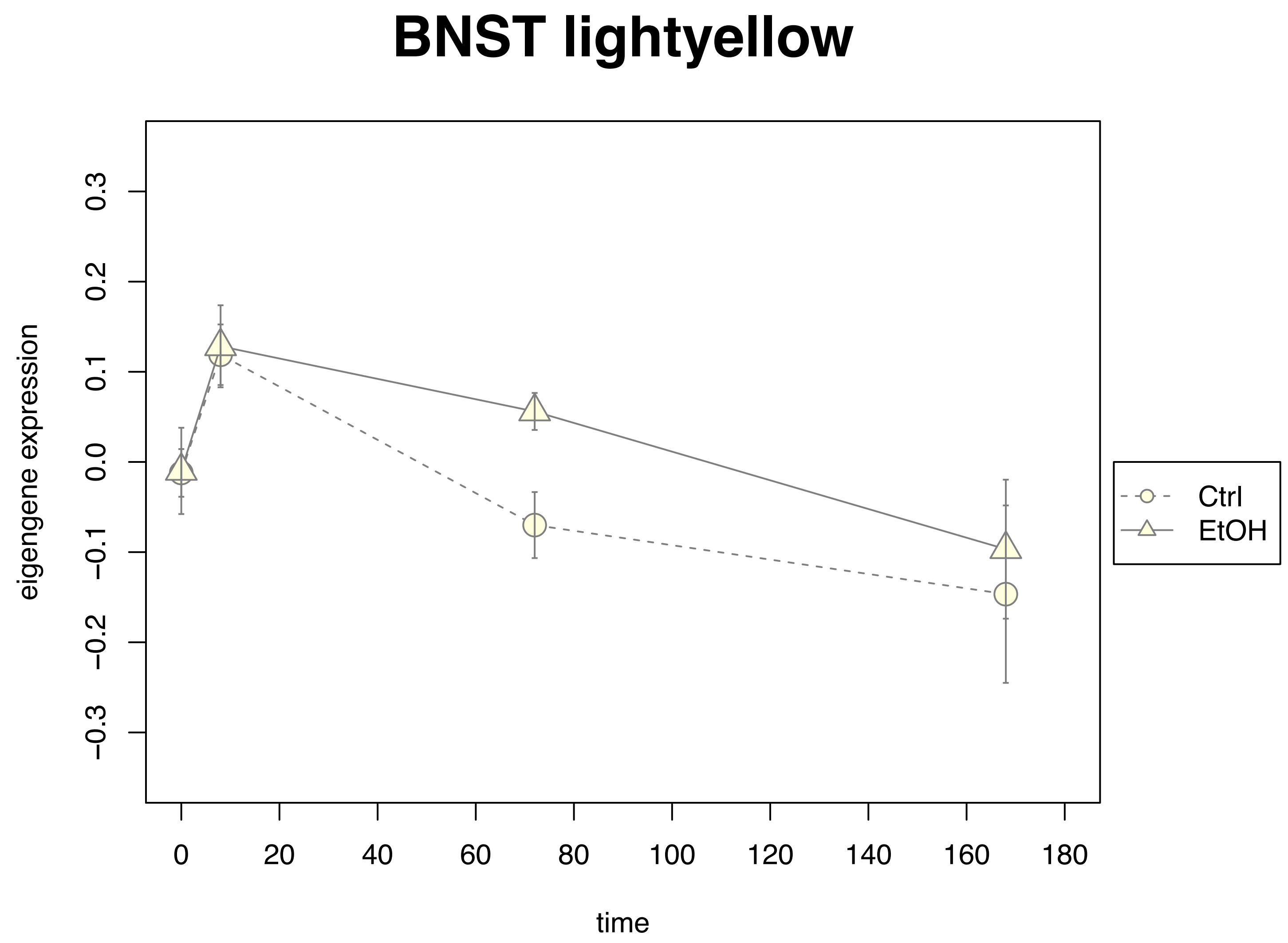

BNST lightyellow
eigengene expression
0.3
0.2
0.1
0.0
−0.1
−0.2
−0.3
0
20
40
60
80
100
120
140
160
180
time
Ctrl
EtOH

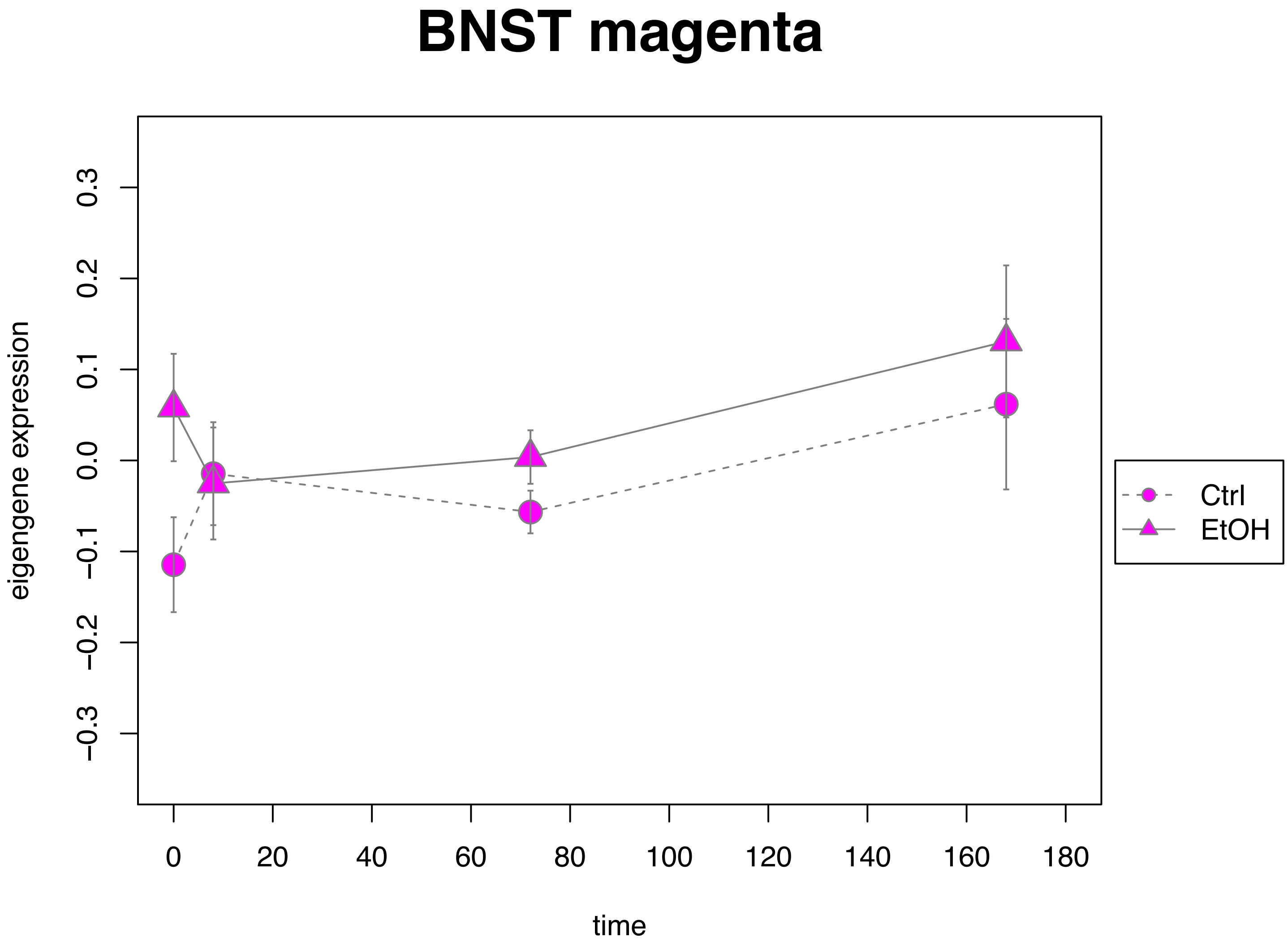

BNST magenta
eigengene expression
time
0.3
0.2
0.1
0.0
−0.1
−0.2
−0.3
0
20
40
60
80
100
120
140
160
180
Ctrl
EtOH

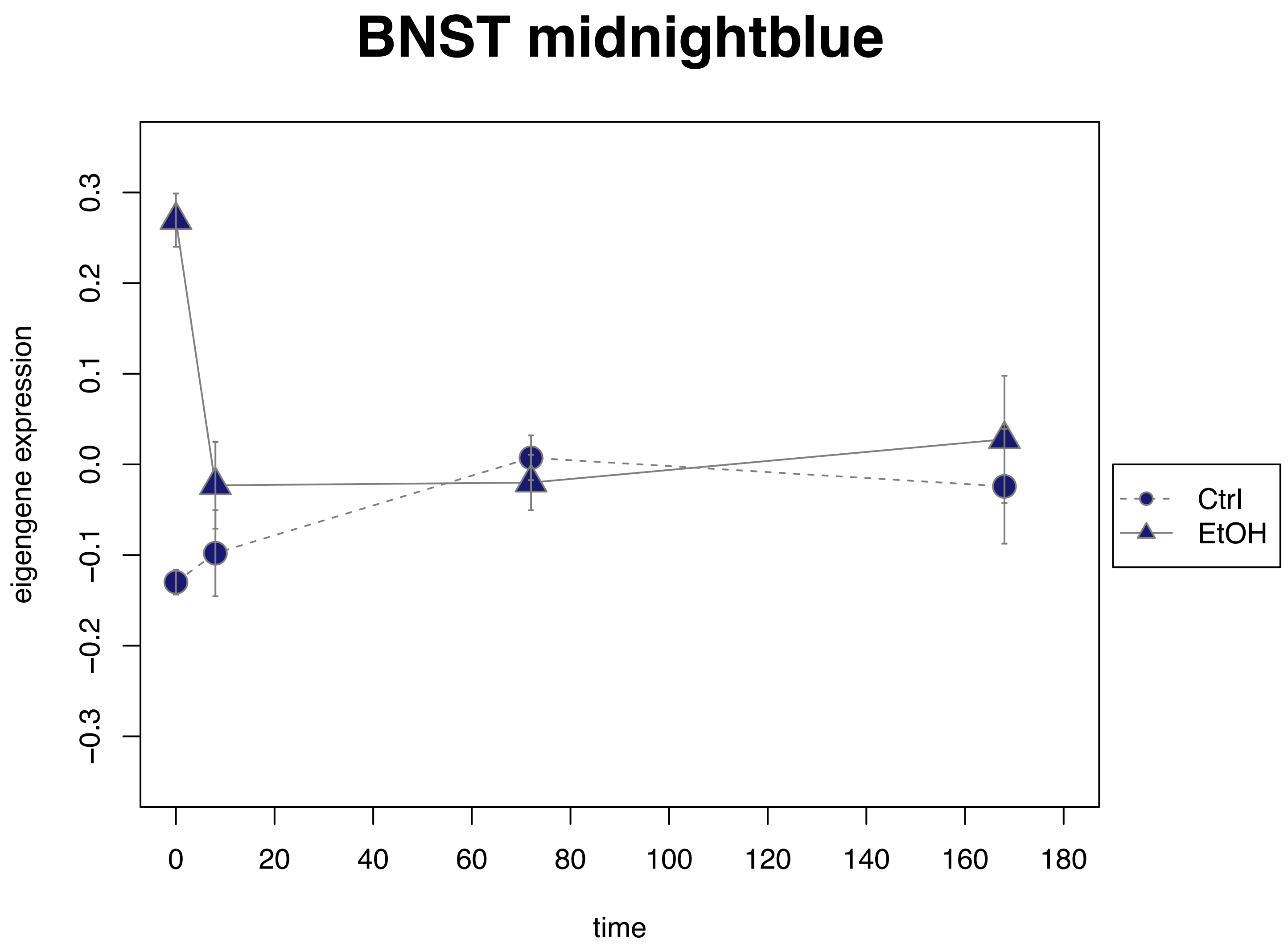
BNST midnightblue
eigengene expression
0.3
0.2
0.1
0.0
−0.1
−0.2
−0.3
0
20
40
60
80
100
120
140
160
180
time
Ctrl
EtOH

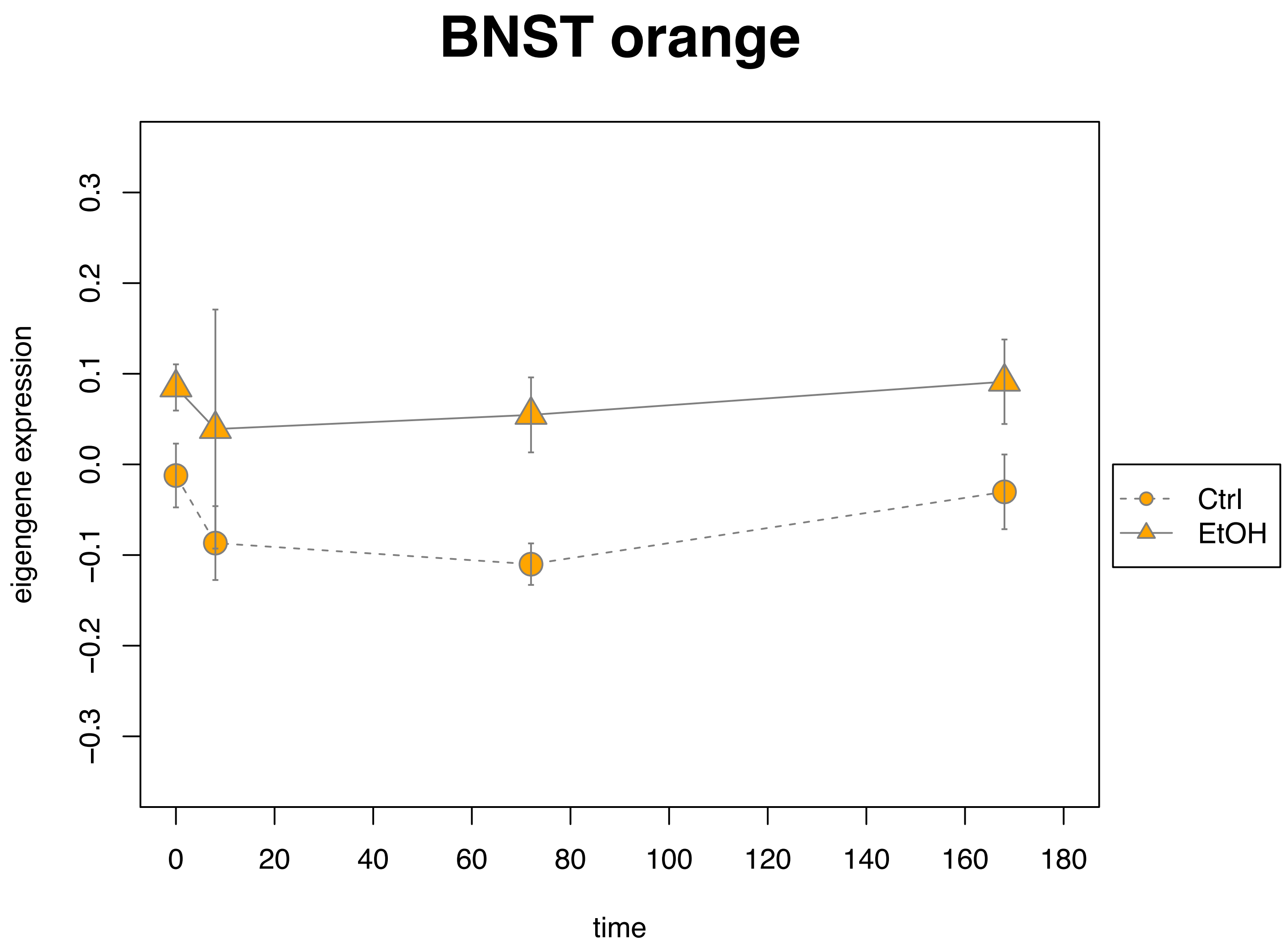
BNST orange
eigengene expression
0.3
0.2
0.1
0.0
−0.1
−0.2
−0.3
0
20
40
60
80
100
120
140
160
180
time
Ctrl
EtOH

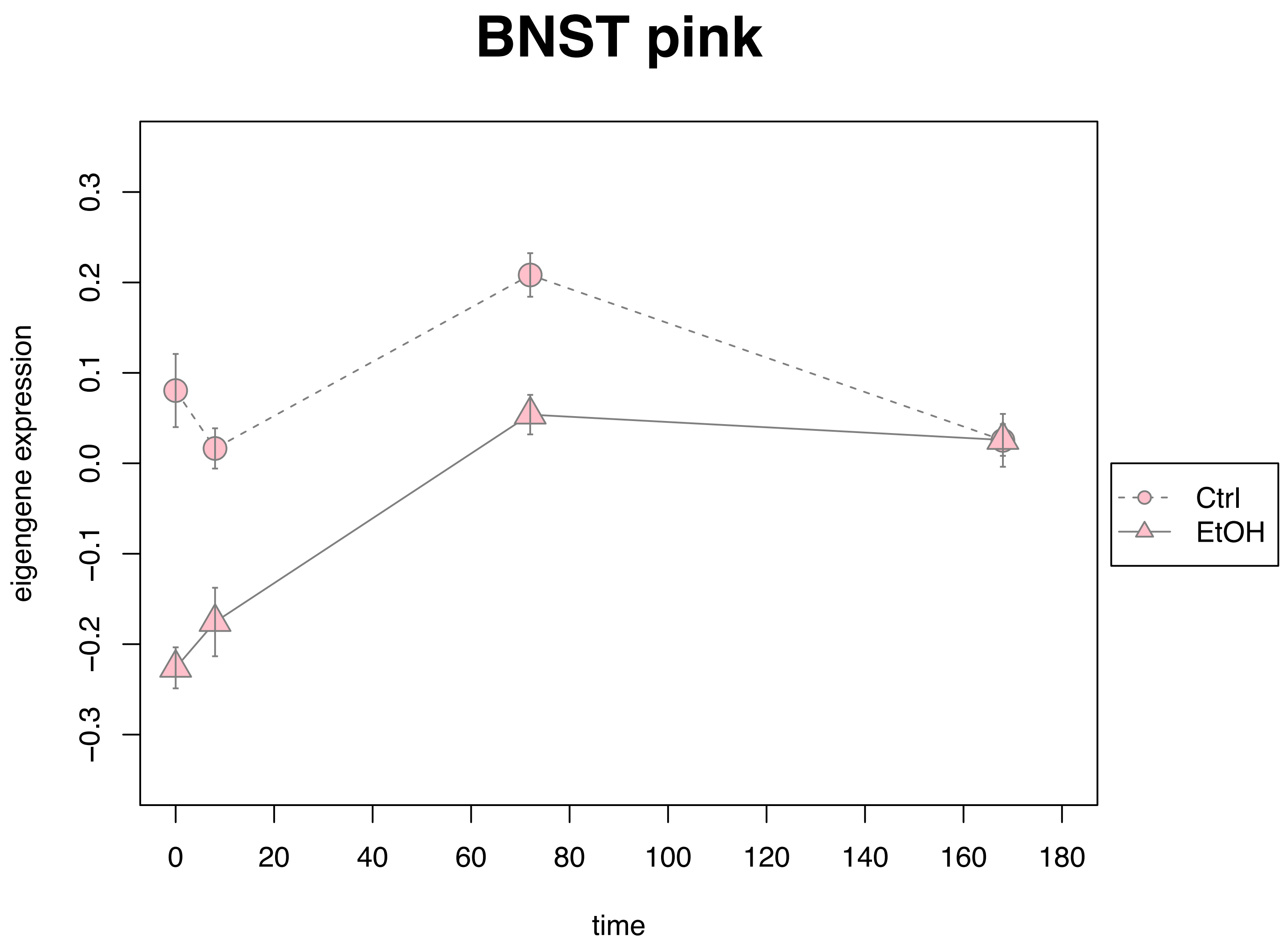
BNST pink
eigengene expression
0.3
0.2
0.1
0.0
−0.1
−0.2
−0.3
time
0
20
40
60
80
100
120
140
160
180
Ctrl
EtOH

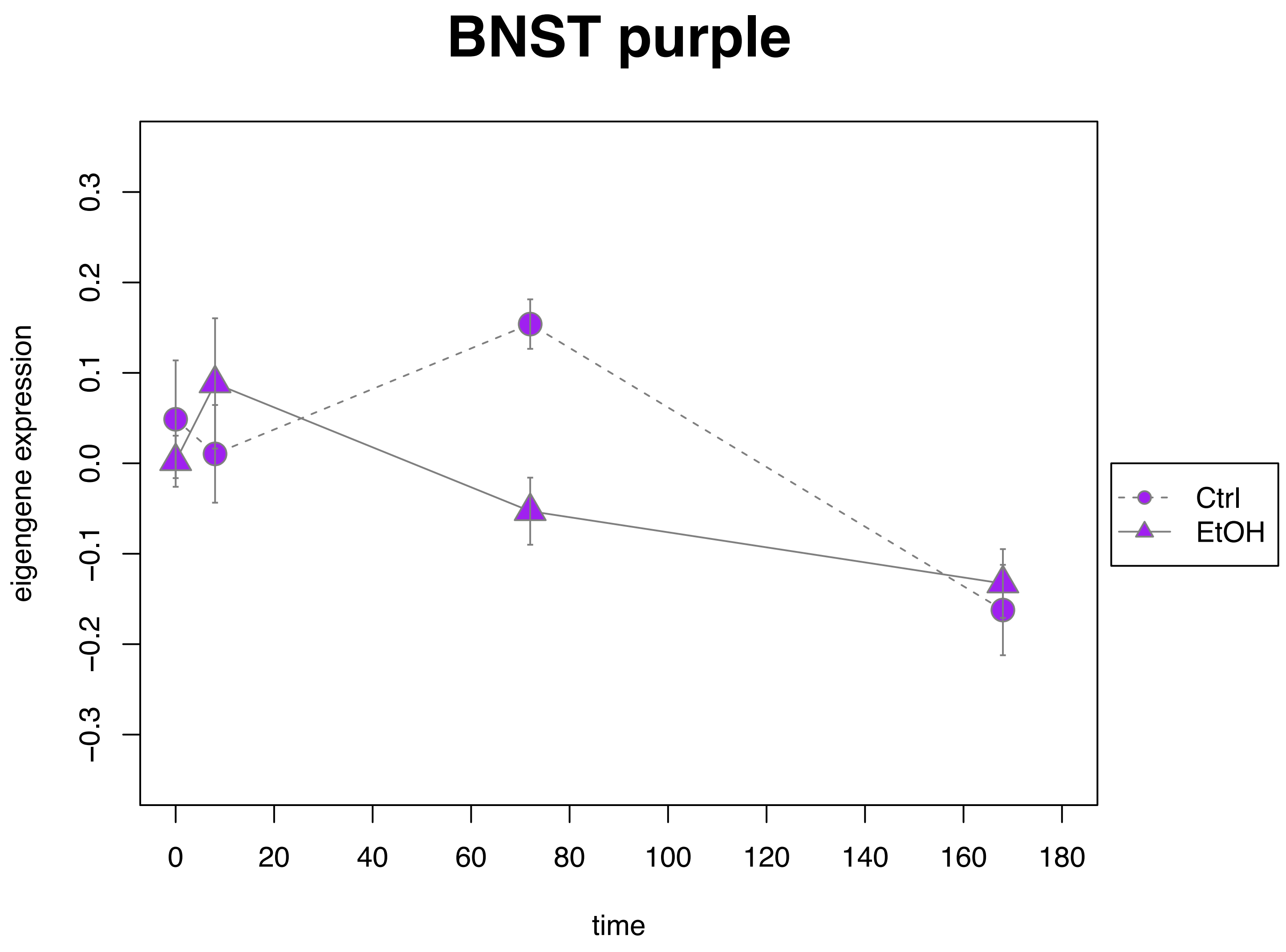
BNST purple
eigengene expression
0.3
0.2
0.1
0.0
−0.1
−0.2
−0.3
0
20
40
60
80
100
120
140
160
180
time
Ctrl
EtOH

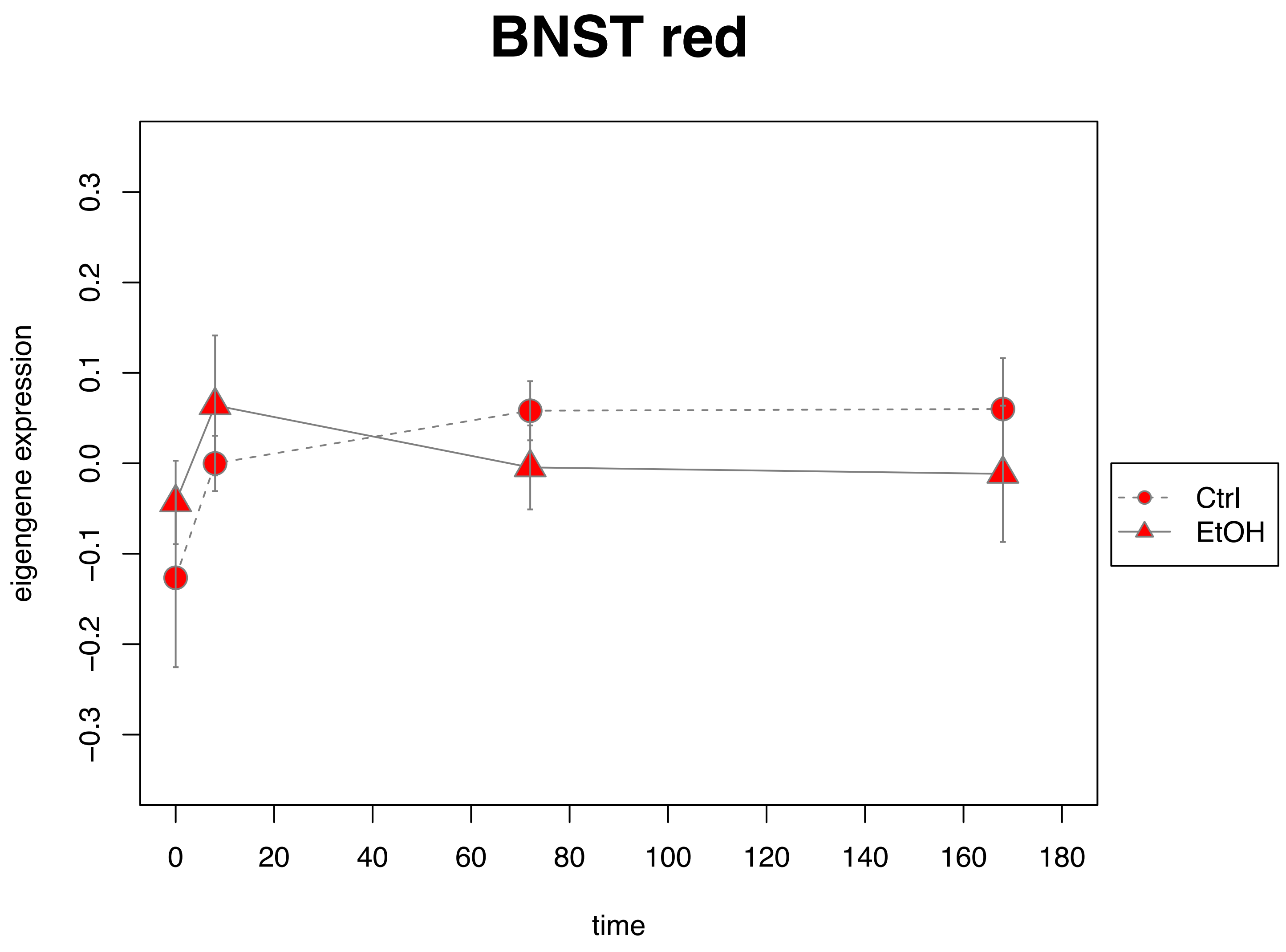
BNST red
eigengene expression
0.3
0.2
0.1
0.0
−0.1
−0.2
−0.3
0
20
40
60
80
100
120
140
160
180
time
Ctrl
EtOH

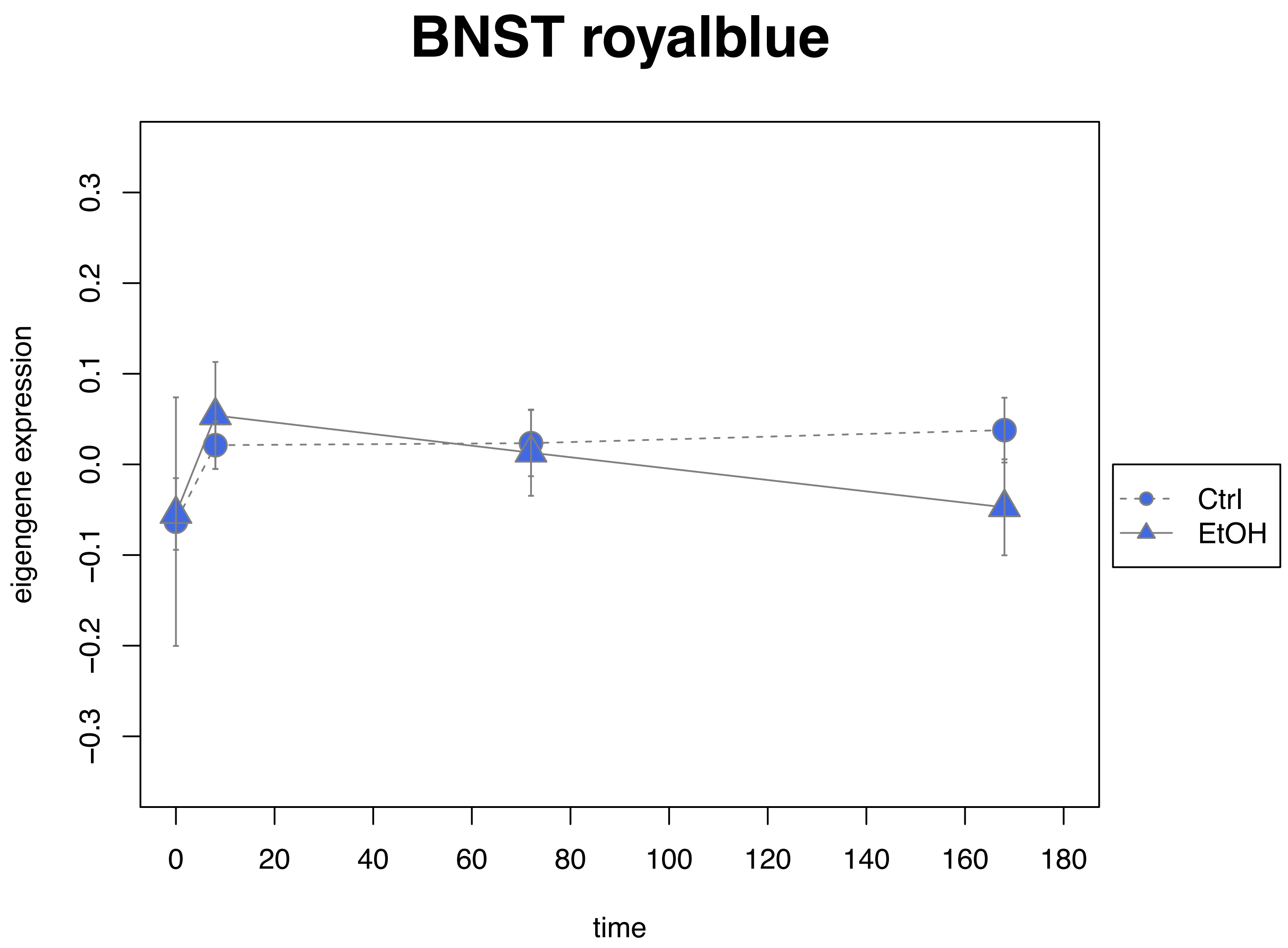

BNST royalblue
0.3
0.2
0.1
0.0
−0.1
−0.2
−0.3
eigengene expression
0
20
40
60
80
100
120
140
160
180
time
Ctrl
EtOH

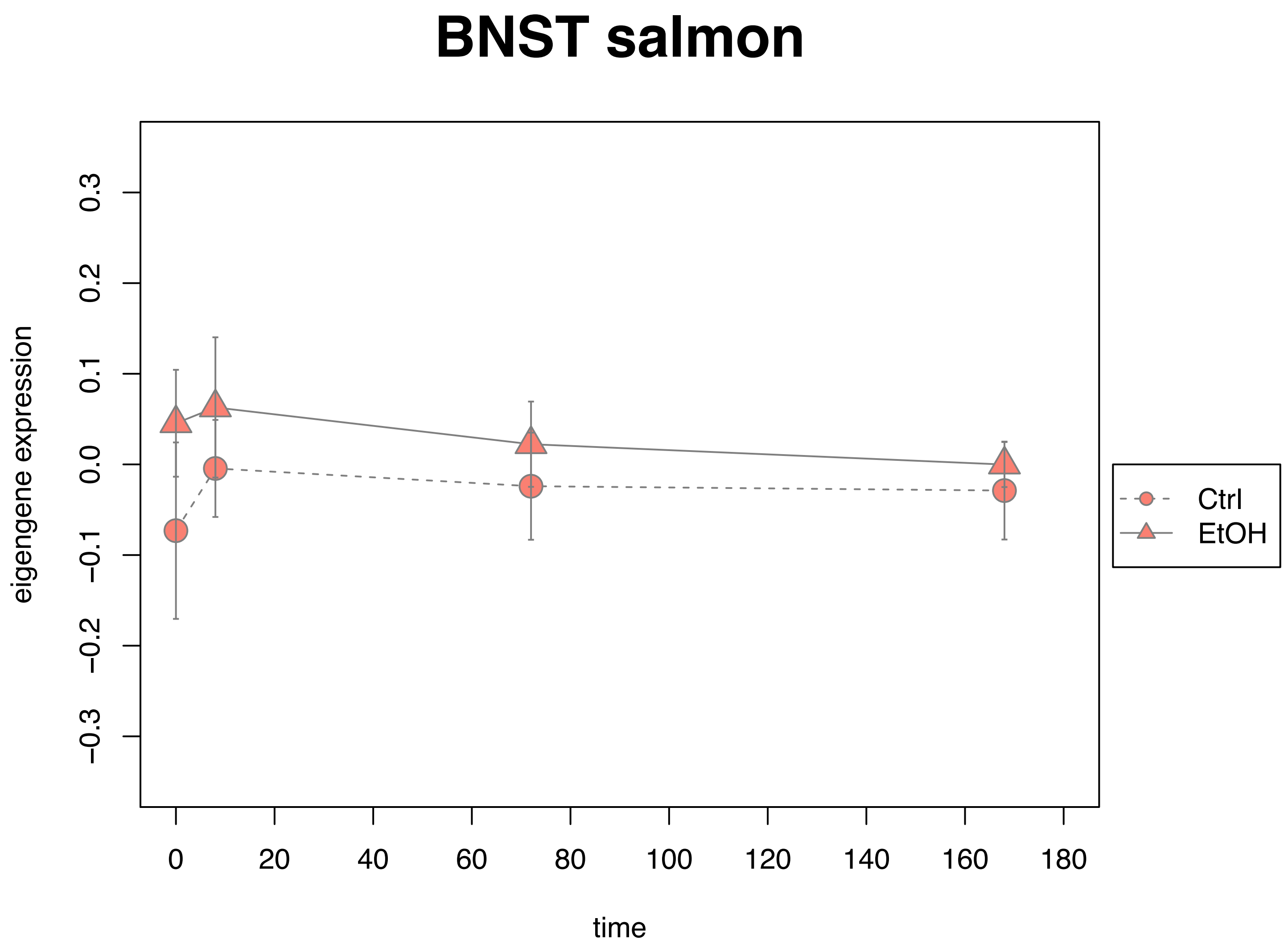
BNST salmon
eigengene expression
time
0.3
0.2
0.1
0.0
−0.1
−0.2
−0.3
0
20
40
60
80
100
120
140
160
180
Ctrl
EtOH

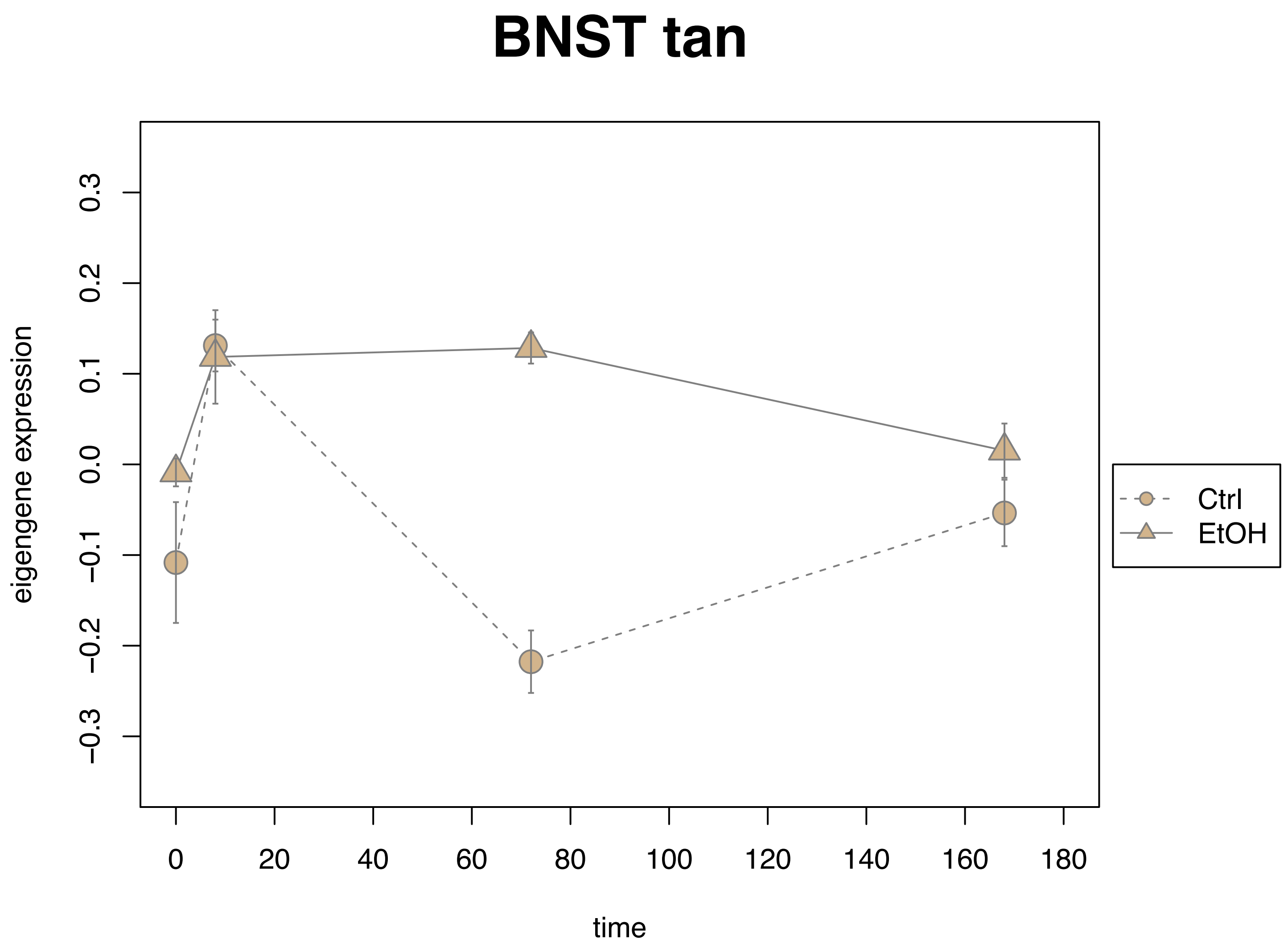

BNST tan
eigengene expression
time
0.3
0.2
0.1
0.0
−0.1
−0.2
−0.3
0
20
40
60
80
100
120
140
160
180
Ctrl
EtOH

BNST turquoise

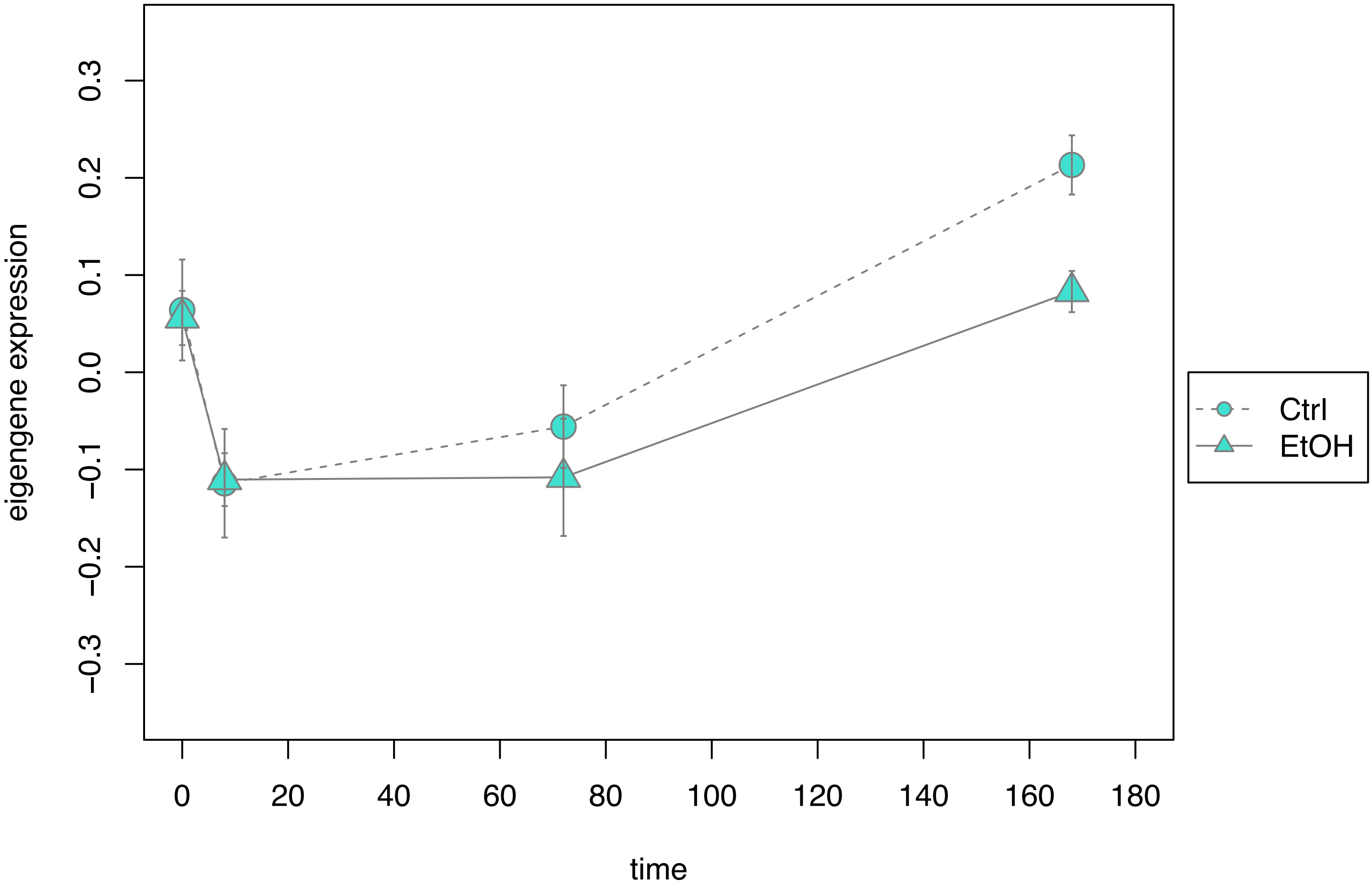

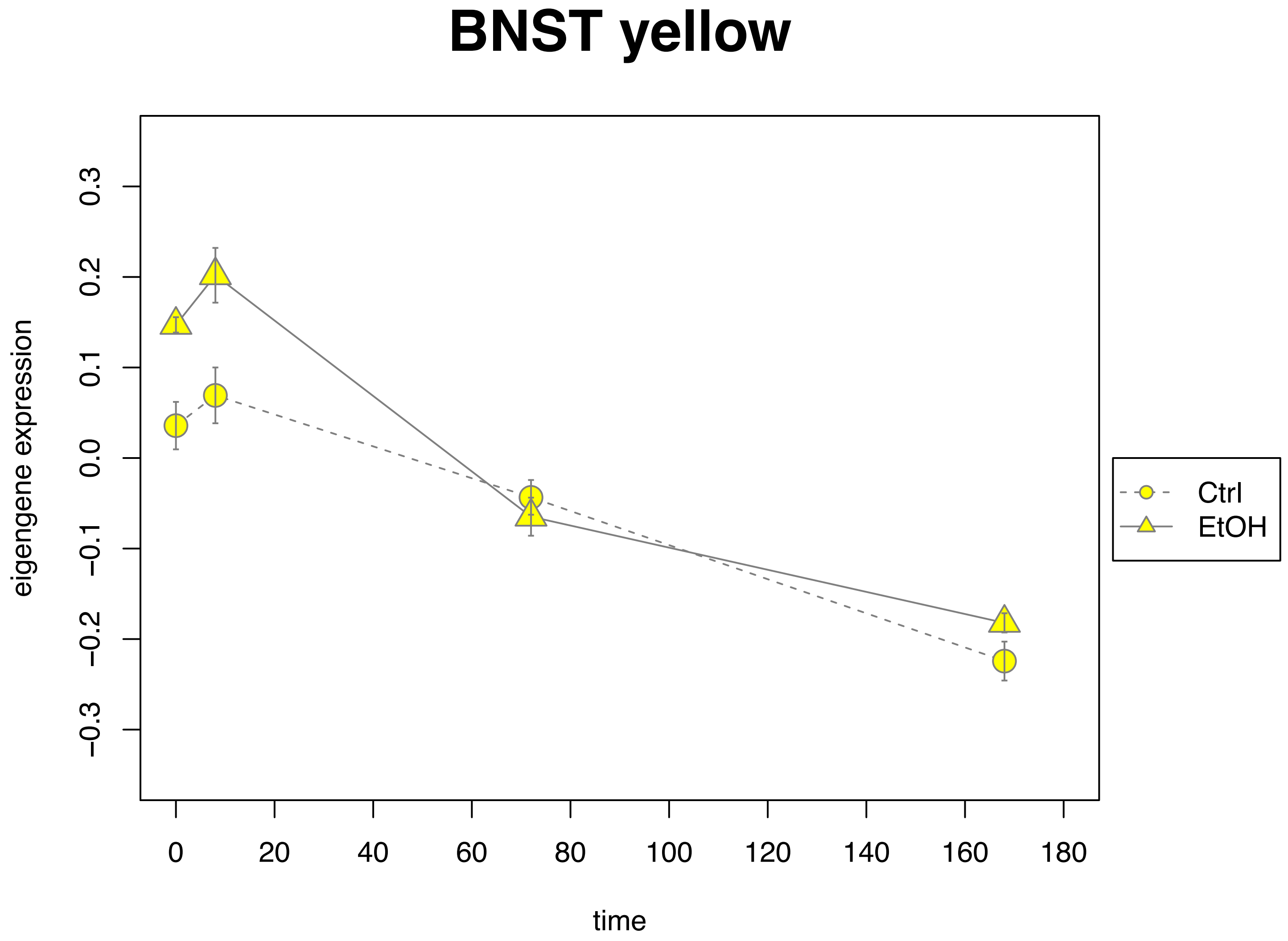
BNST yellow
eigengene expression
0.3
0.2
0.1
0.0
−0.1
−0.2
−0.3
0
20
40
60
80
100
120
140
160
180
time
Ctrl
EtOH
