# Supplementary material for: Time-Course Analysis of Brain Regional Expression Network Responses to Chronic Intermittent Ethanol and Withdrawal: Implications for Mechanisms Underlying Excessive Ethanol Consumption
Source: PLoS One. 2016 Jan 5;11(1):e0146257. doi: 10.1371/journal.pone.0146257 (PMC4701666; doi:10.1371/journal.pone.0146257)
Supplement: S5 Fig — (PDF) [file pone.0146257.s005.pdf]

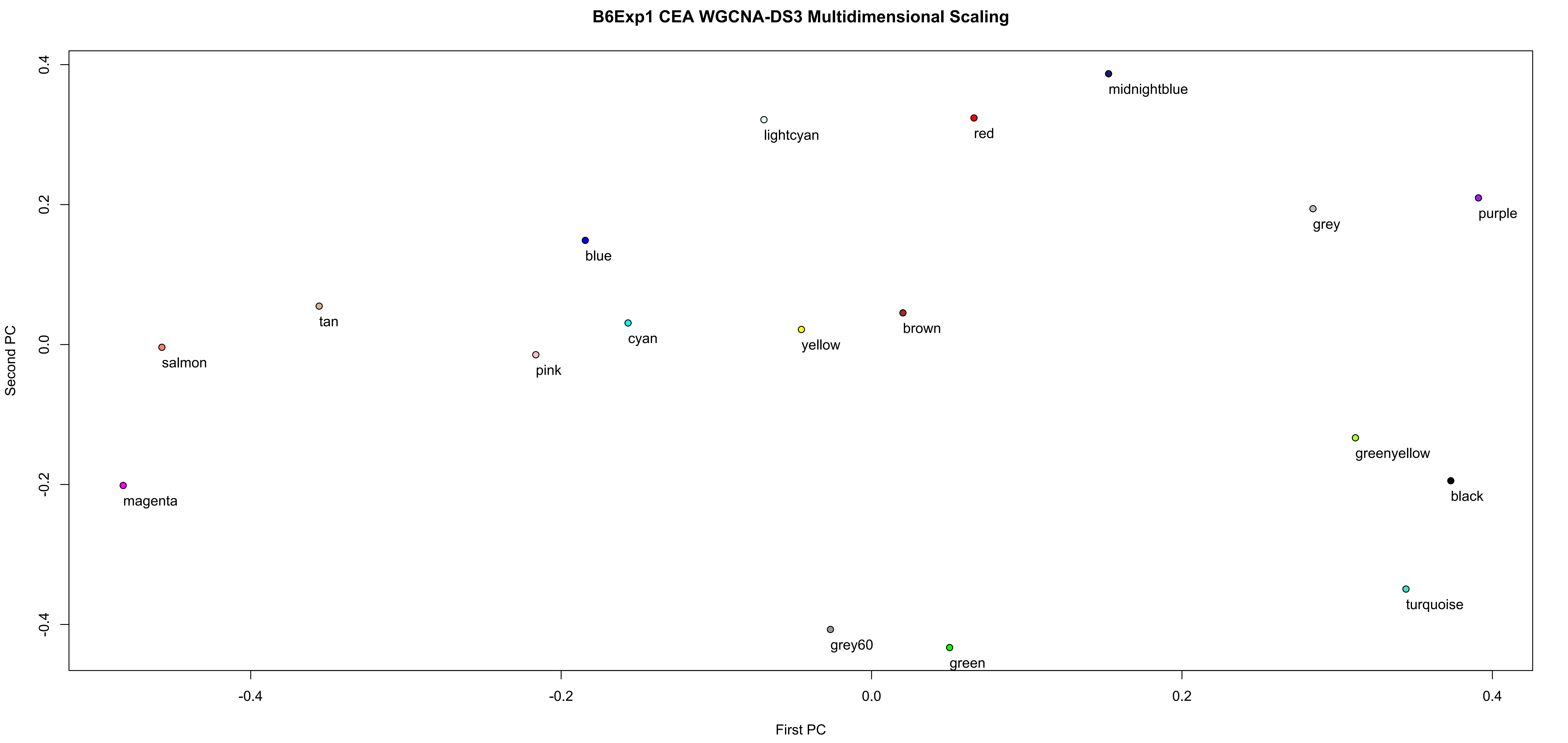
B6Exp1 CEA WGCNA-DS3 Multidimensional Scaling
0.4
0.2
0.0
-0.2
-0.4
Second PC
-0.4
-0.2
0.0
0.2
0.4
First PC
midnightblue
lightcyan
red
purple
grey
blue
tan
cyan
brown
yellow
salmon
pink
greenyellow
black
magenta
turquoise
grey60
green

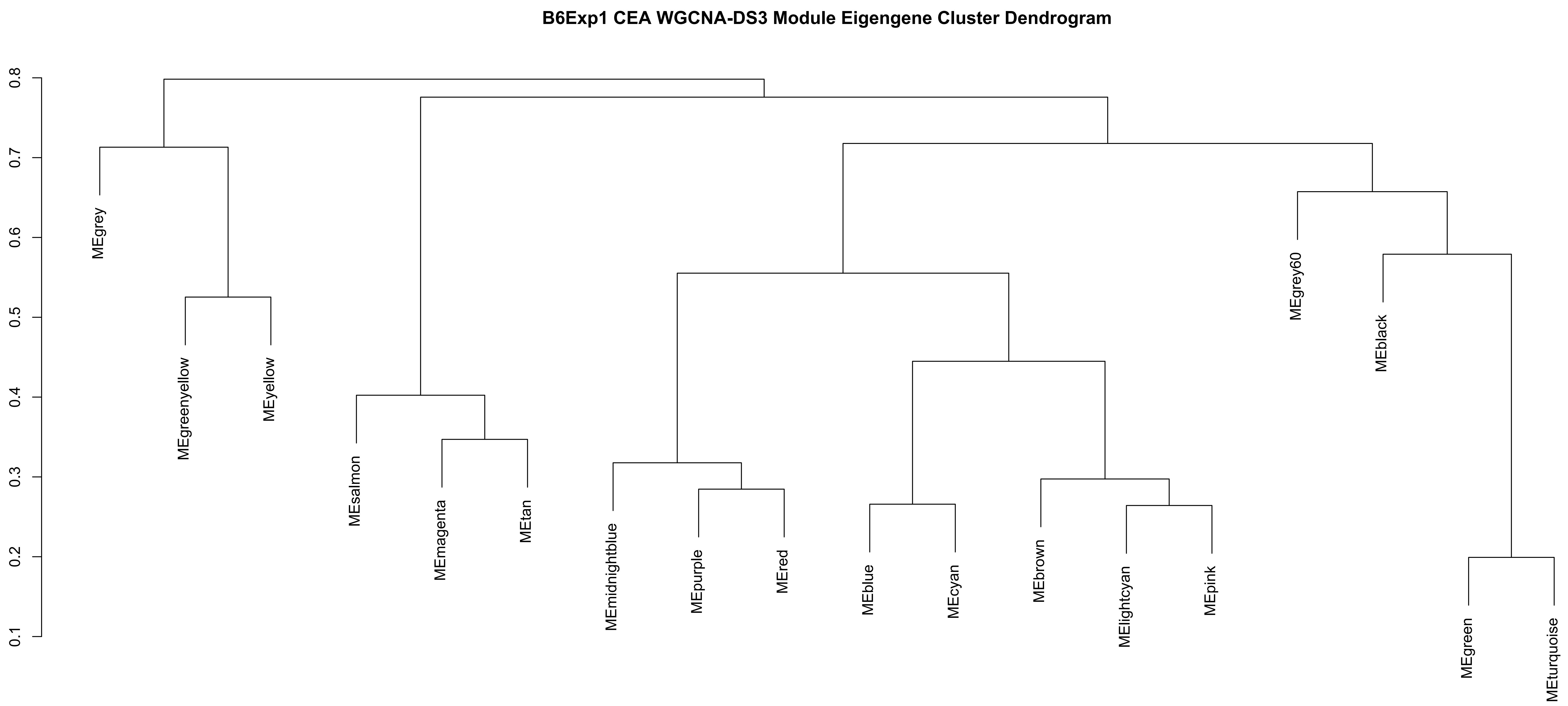
B6Exp1 CEA WGCNA-DS3 Module Eigengene Cluster Dendrogram
0.1
0.2
0.3
0.4
0.5
0.6
0.7
0.8
MEgrey
MEgreenyellow
MEyellow
MEsalmon
MEmagenta
MEtan
MEmidnightblue
MEpurple
MEred
MEblue
MEcyan
MEbrown
MElightcyan
MEpink
MEgrey60
MEblack
MEgreen
MEturquoise

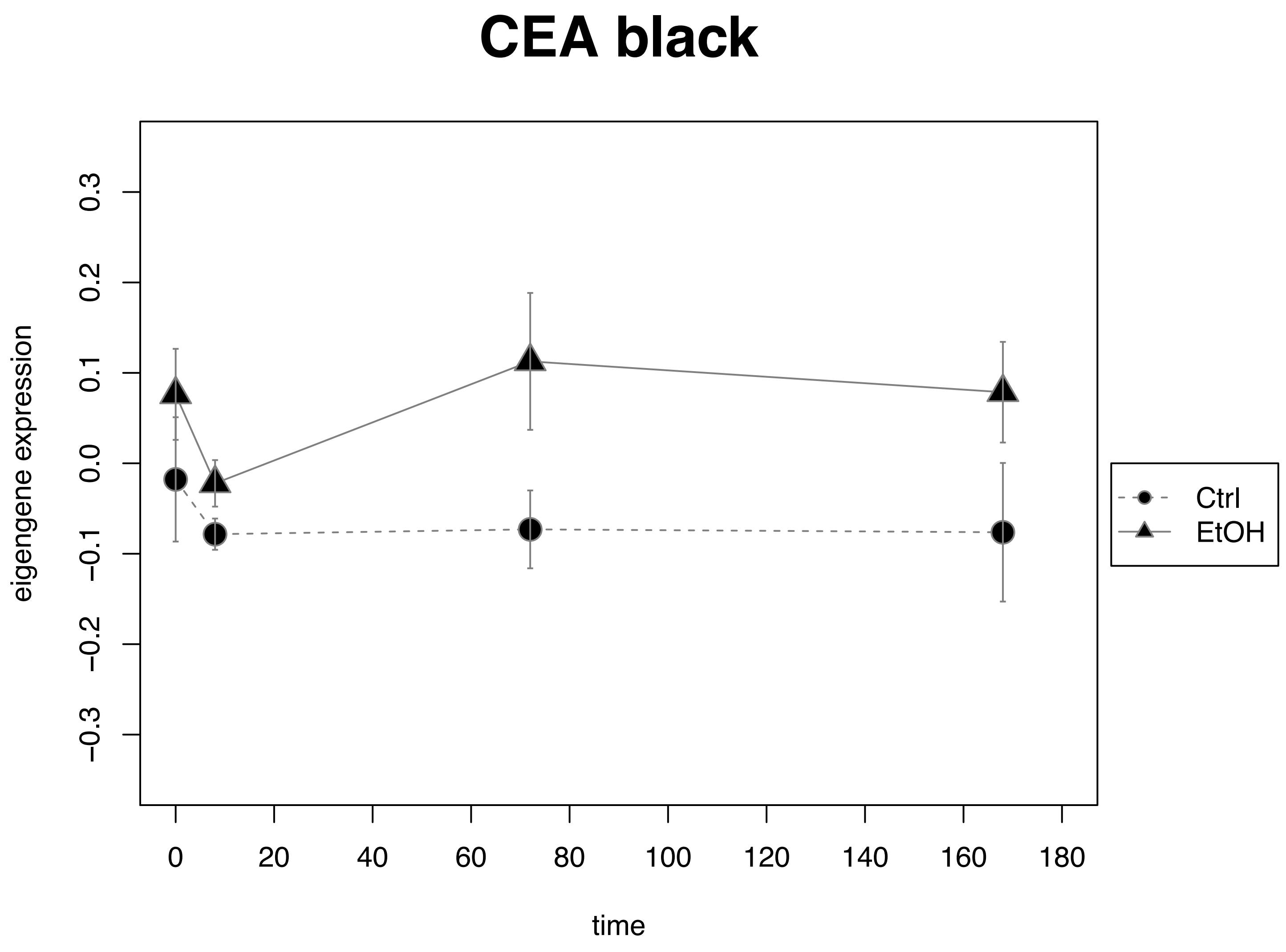
CEA black
eigengene expression
0.3
0.2
0.1
0.0
−0.1
−0.2
−0.3
0
20
40
60
80
100
120
140
160
180
time
Ctrl
EtOH

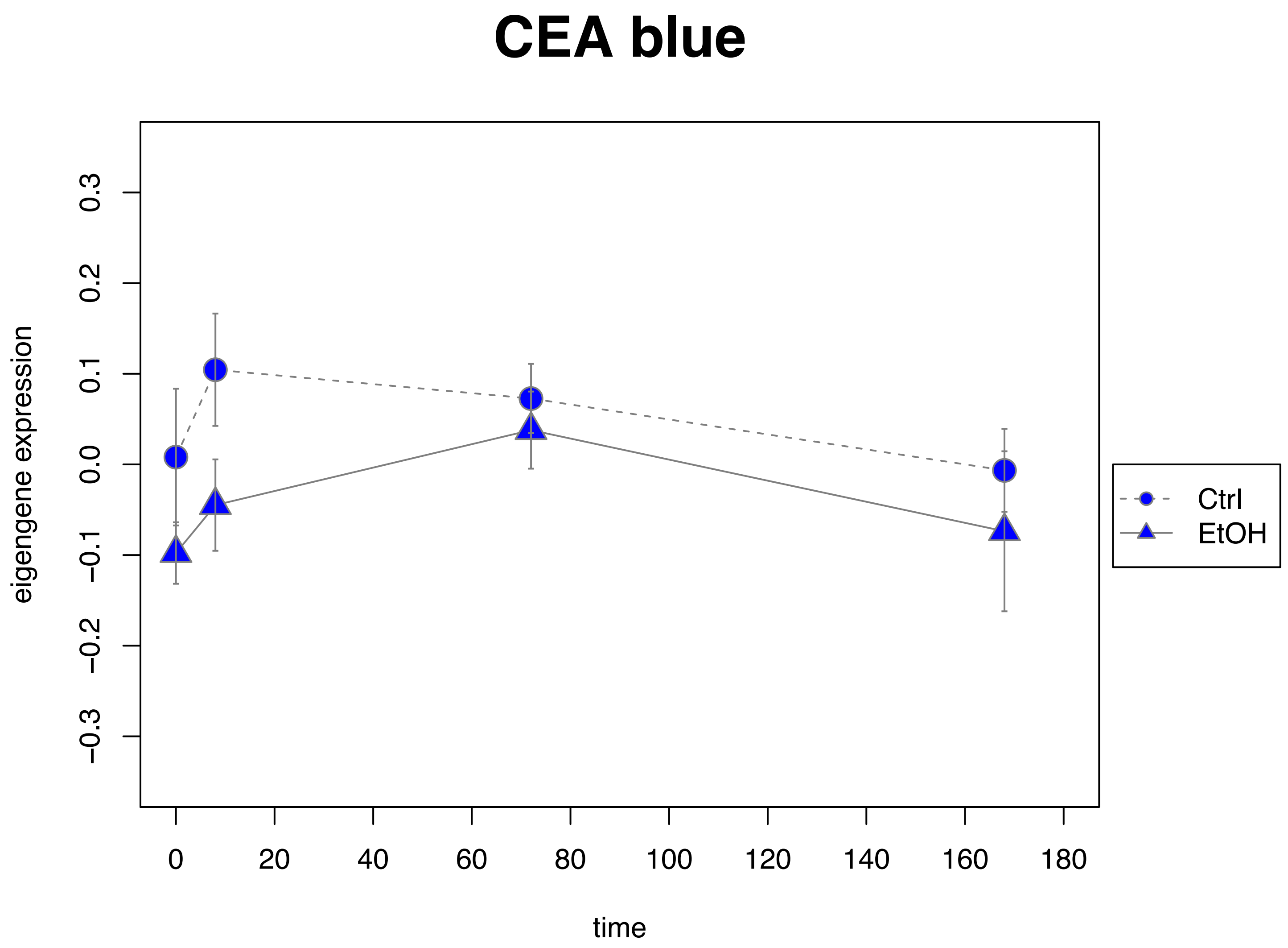
CEA blue
eigengene expression
0.3
0.2
0.1
0.0
−0.1
−0.2
−0.3
0
20
40
60
80
100
120
140
160
180
time
Ctrl
EtOH

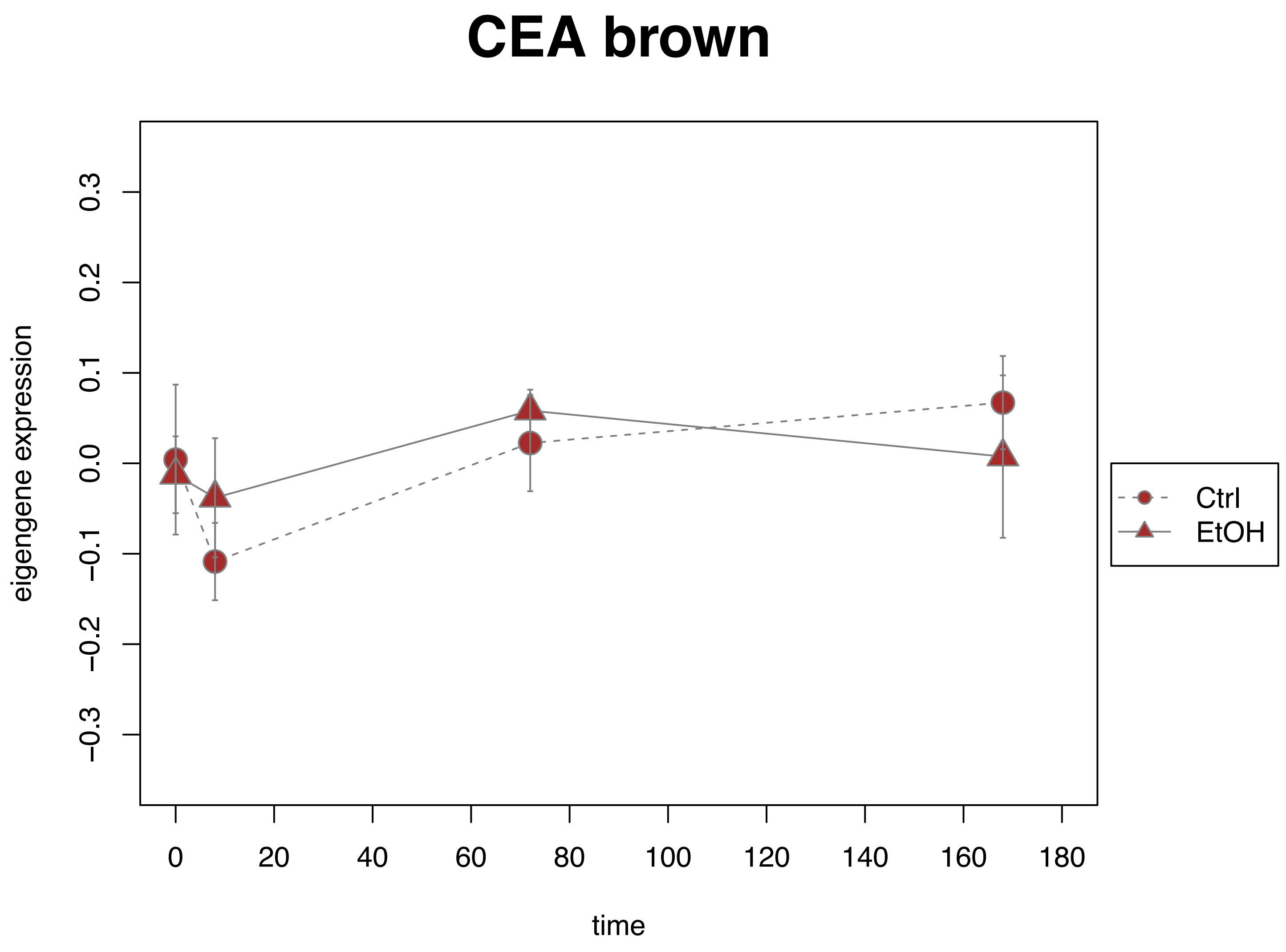
CEA brown
eigengene expression
time
Ctrl
EtOH
0.3
0.2
0.1
0.0
−0.1
−0.2
−0.3
0
20
40
60
80
100
120
140
160
180

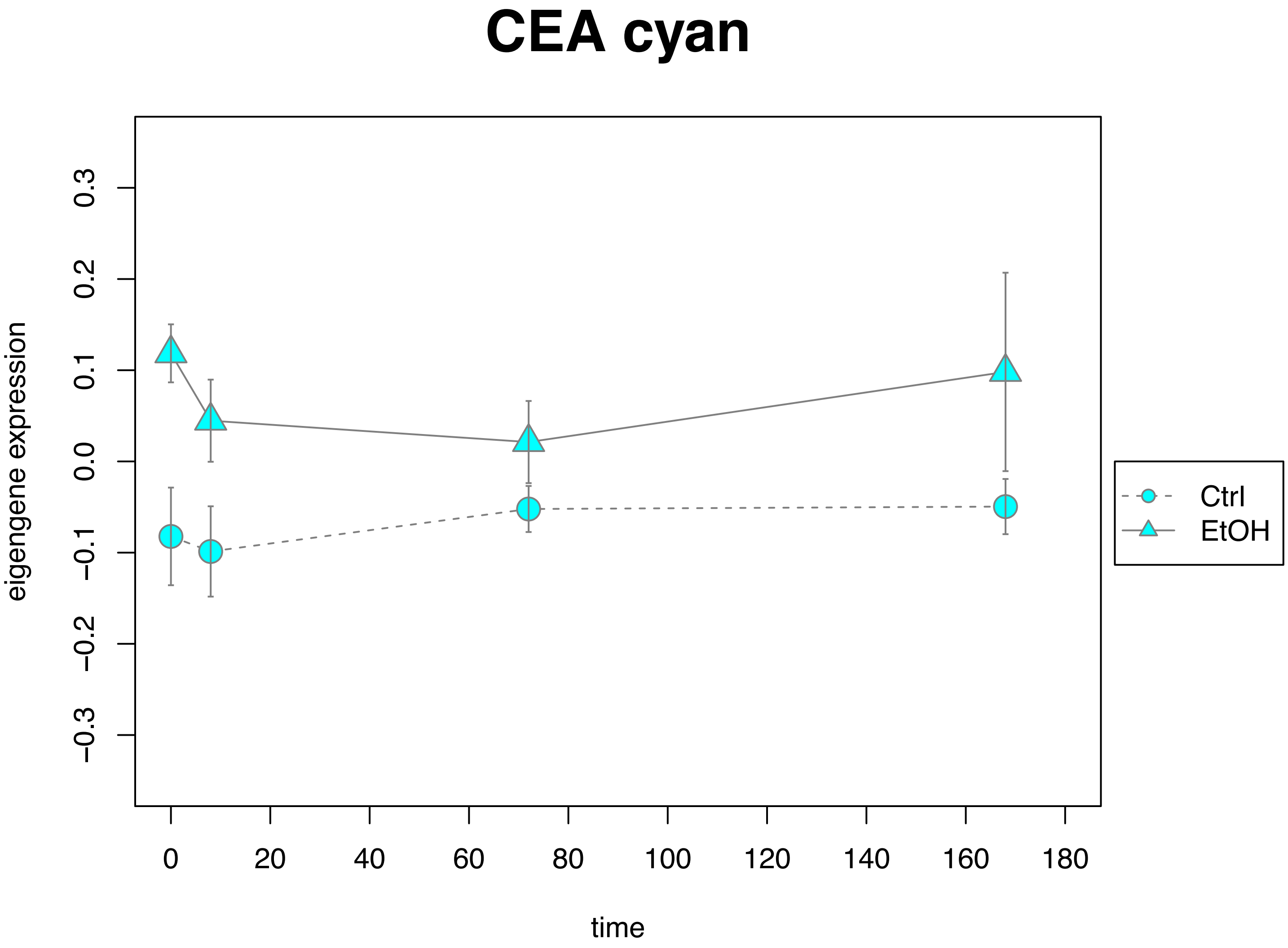

CEA cyan
eigengene expression
time
0.3
0.2
0.1
0.0
−0.1
−0.2
−0.3
0
20
40
60
80
100
120
140
160
180
Ctrl
EtOH

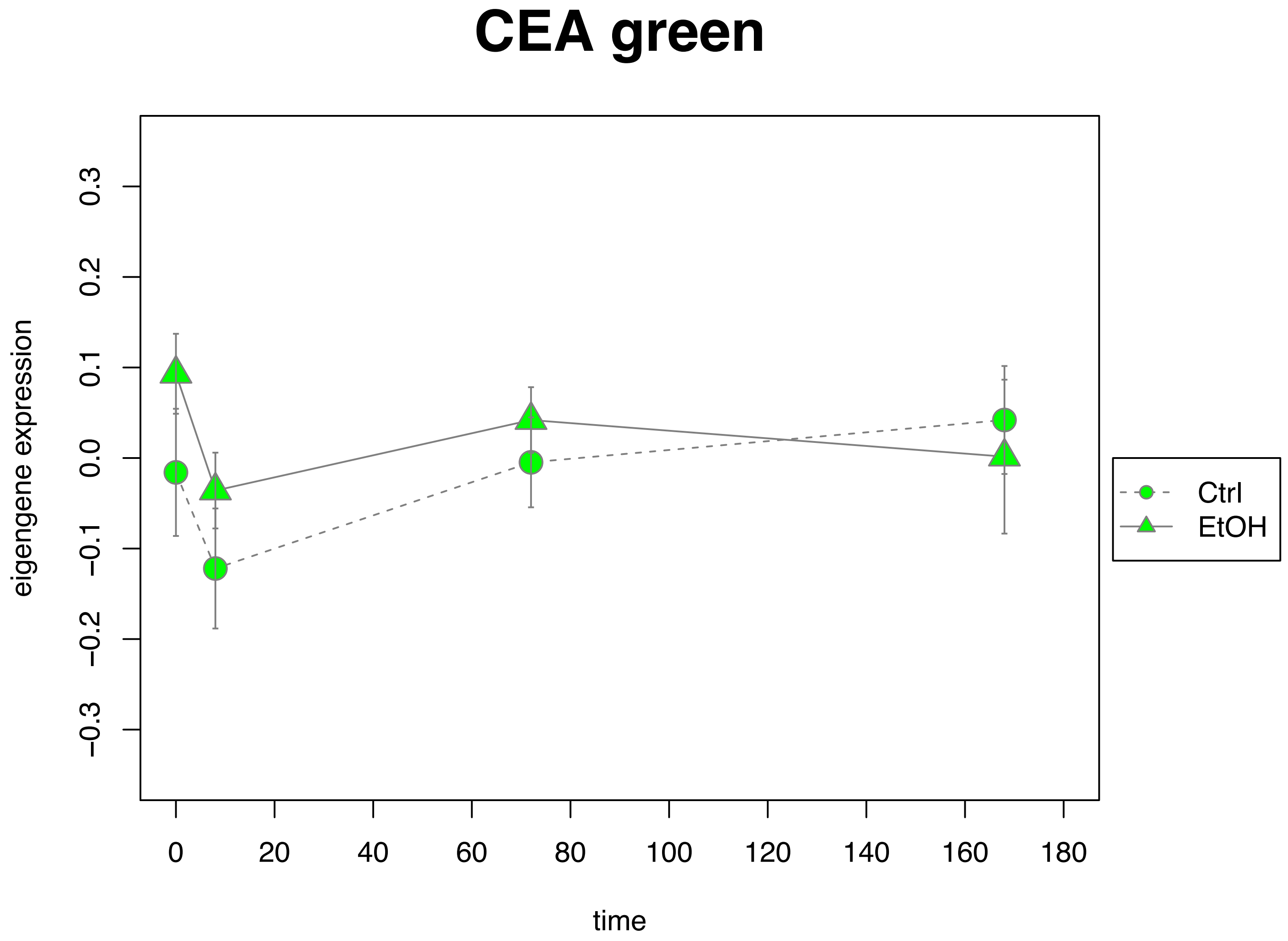

CEA green
eigengene expression
0.3
0.2
0.1
0.0
−0.1
−0.2
−0.3
0
20
40
60
80
100
120
140
160
180
time
Ctrl
EtOH

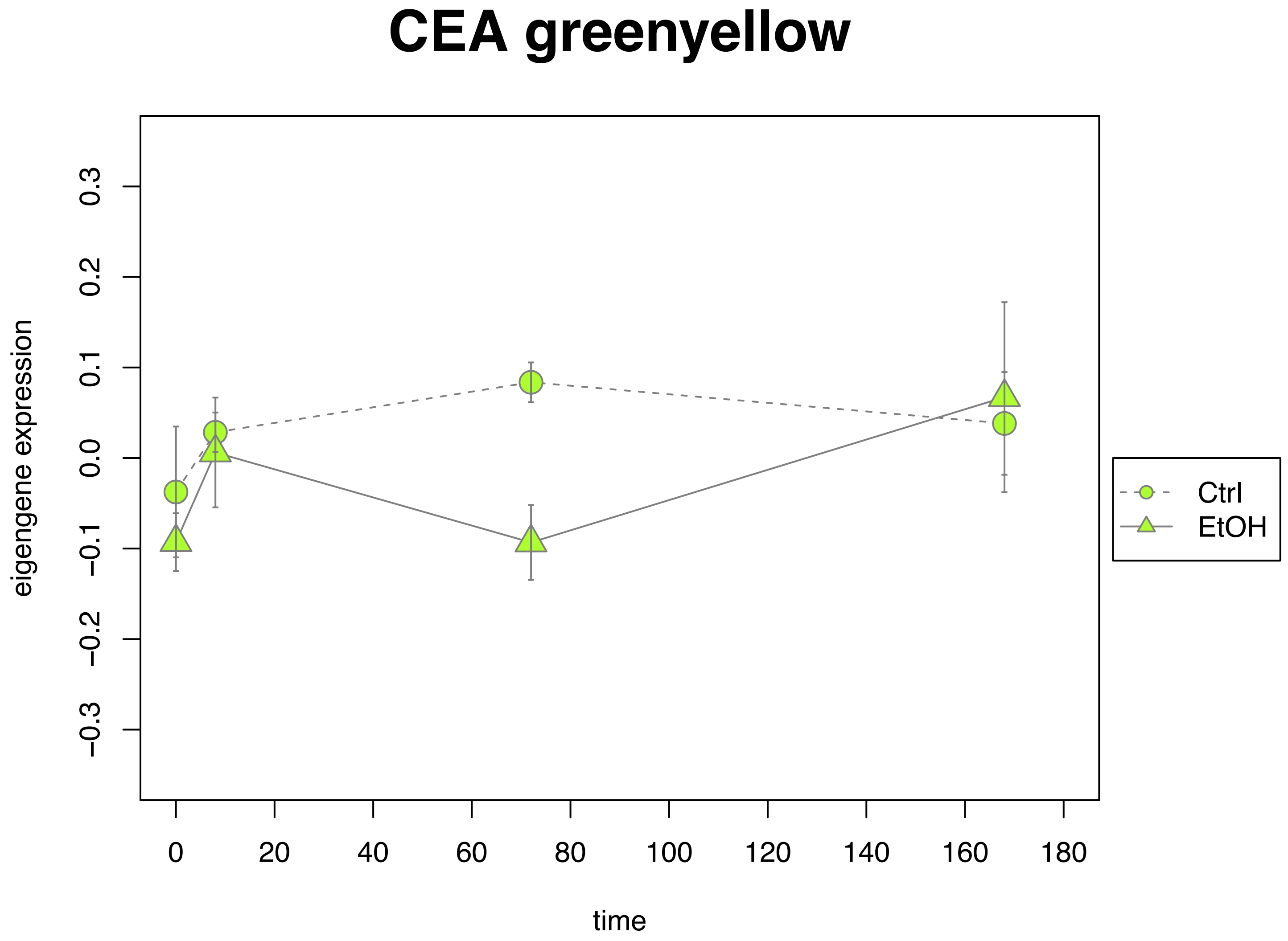

CEA greenyellow
eigengene expression
0.3
0.2
0.1
0.0
−0.1
−0.2
−0.3
0
20
40
60
80
100
120
140
160
180
time
Ctrl
EtOH

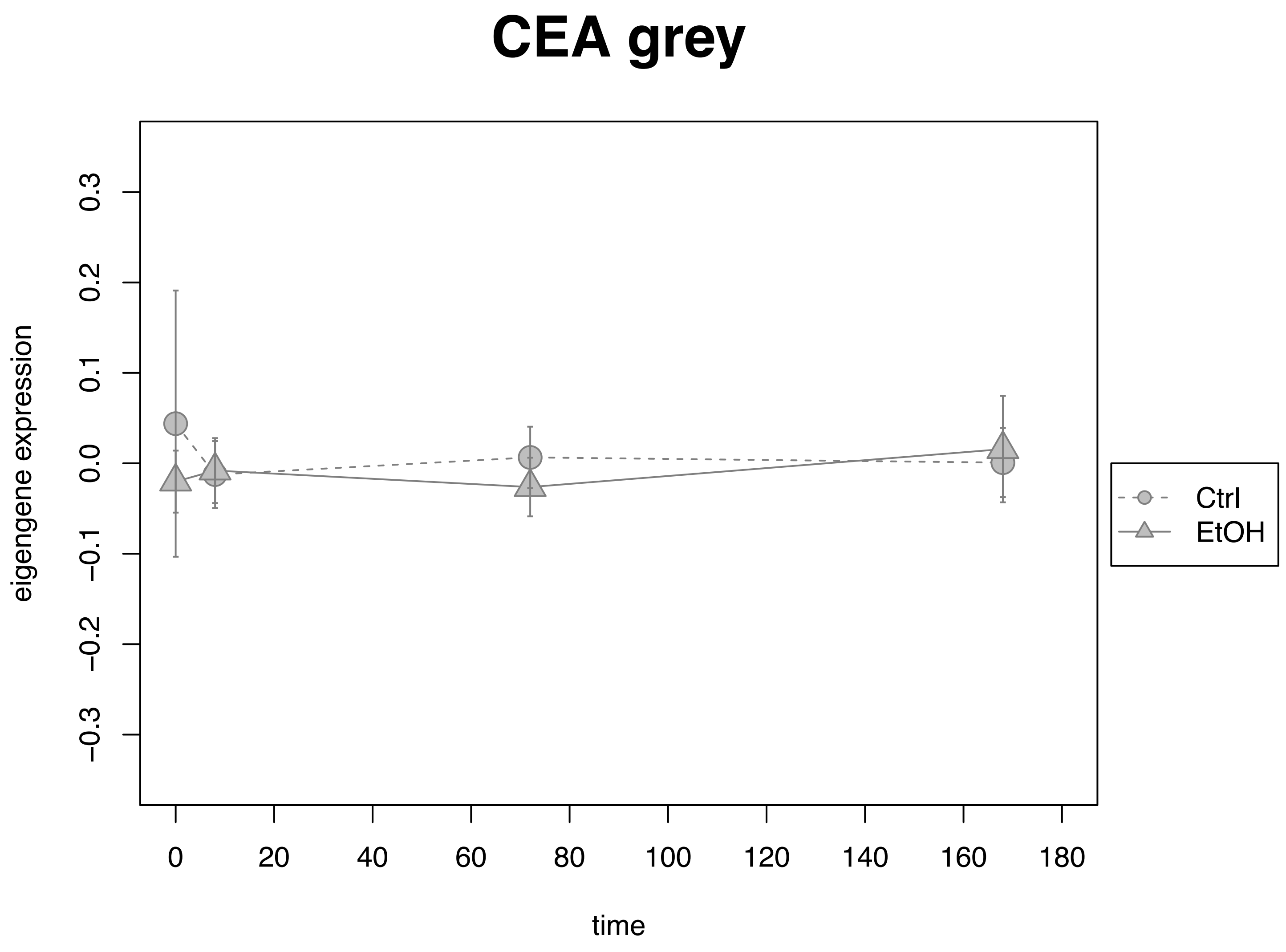
CEA grey
eigengene expression
time
0.3
0.2
0.1
0.0
−0.1
−0.2
−0.3
0
20
40
60
80
100
120
140
160
180
Ctrl
EtOH

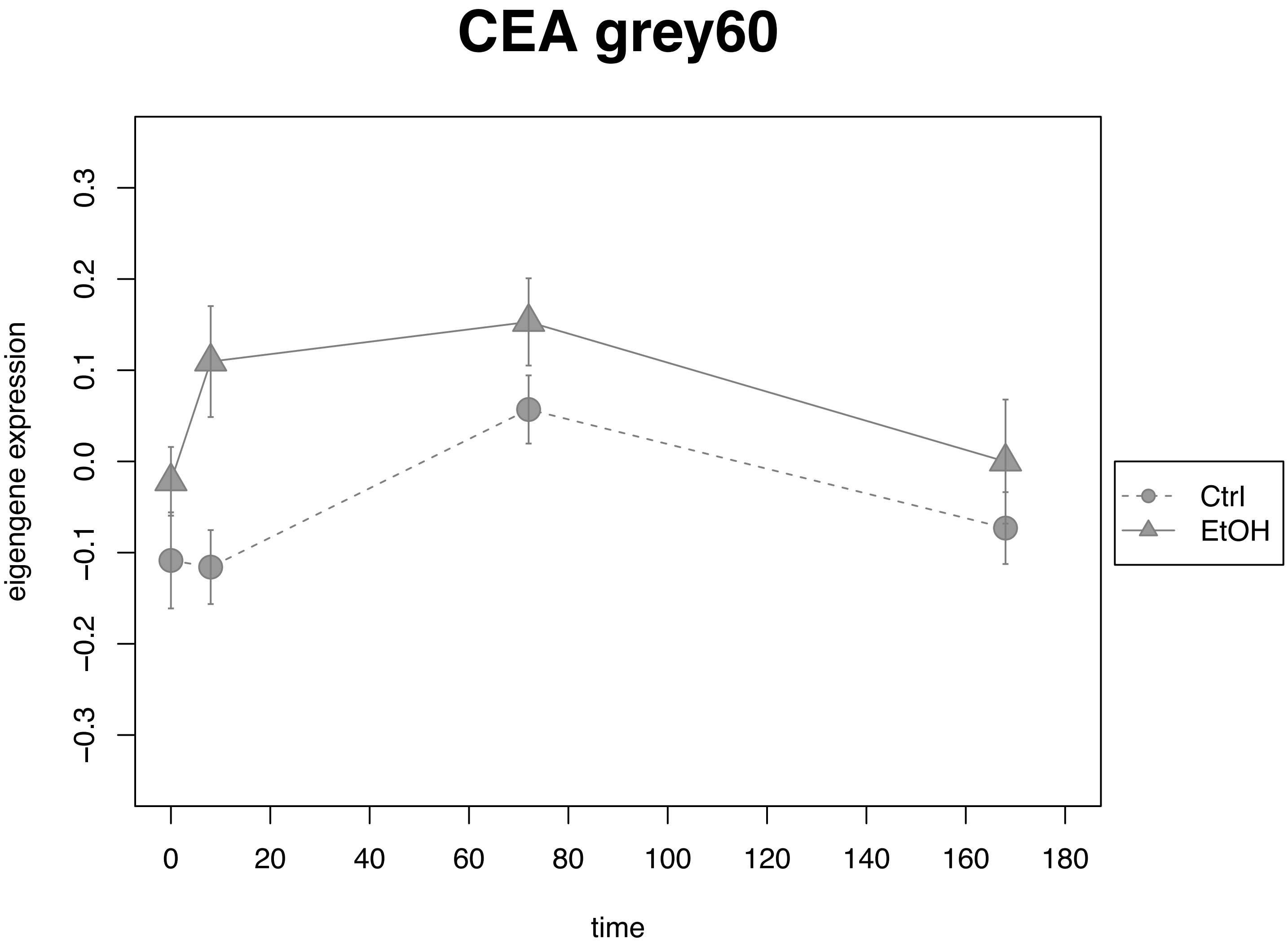

CEA grey60
eigengene expression
0.3
0.2
0.1
0.0
−0.1
−0.2
−0.3
0
20
40
60
80
100
120
140
160
180
time
Ctrl
EtOH

CEA lightcyan

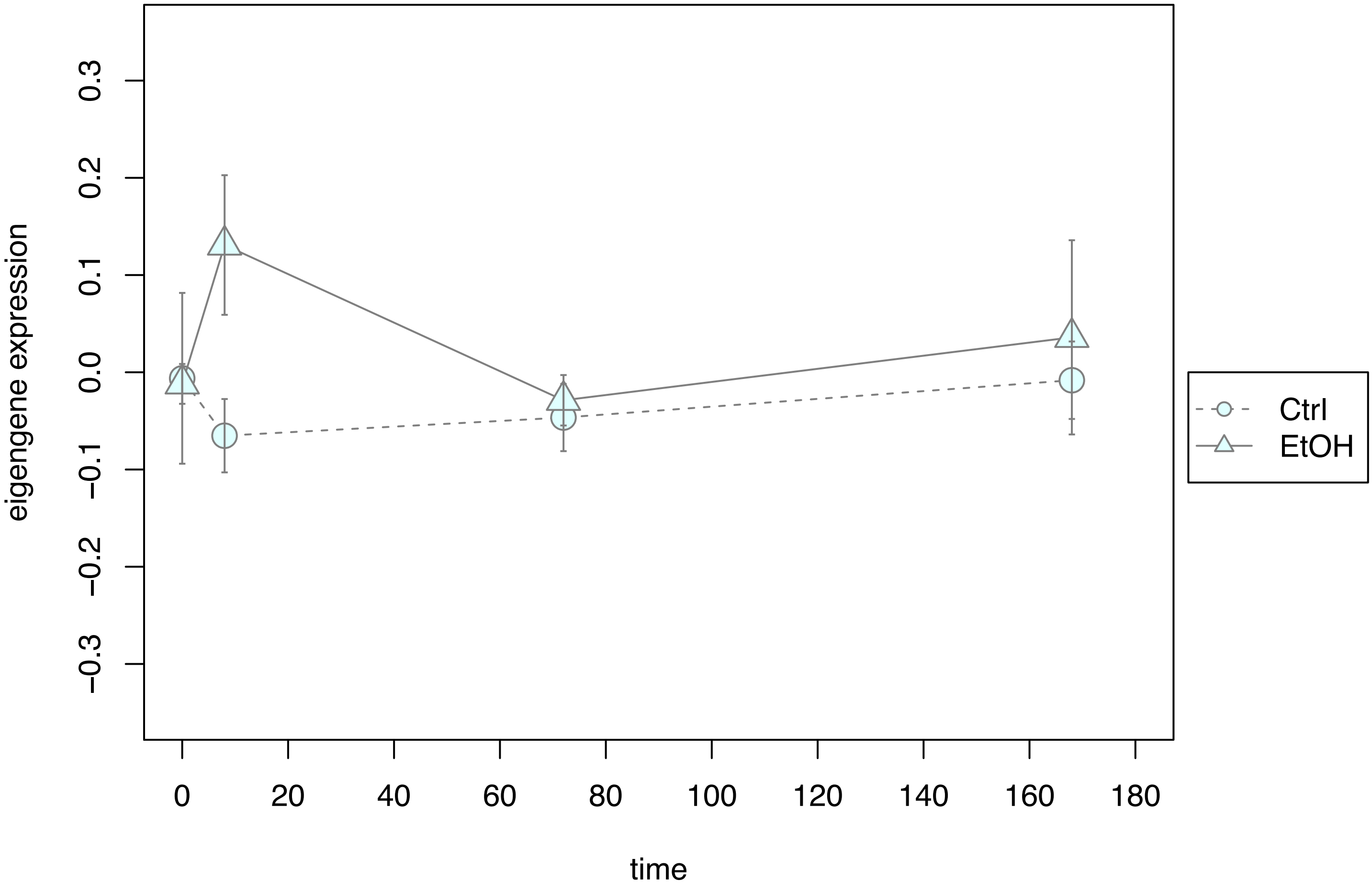

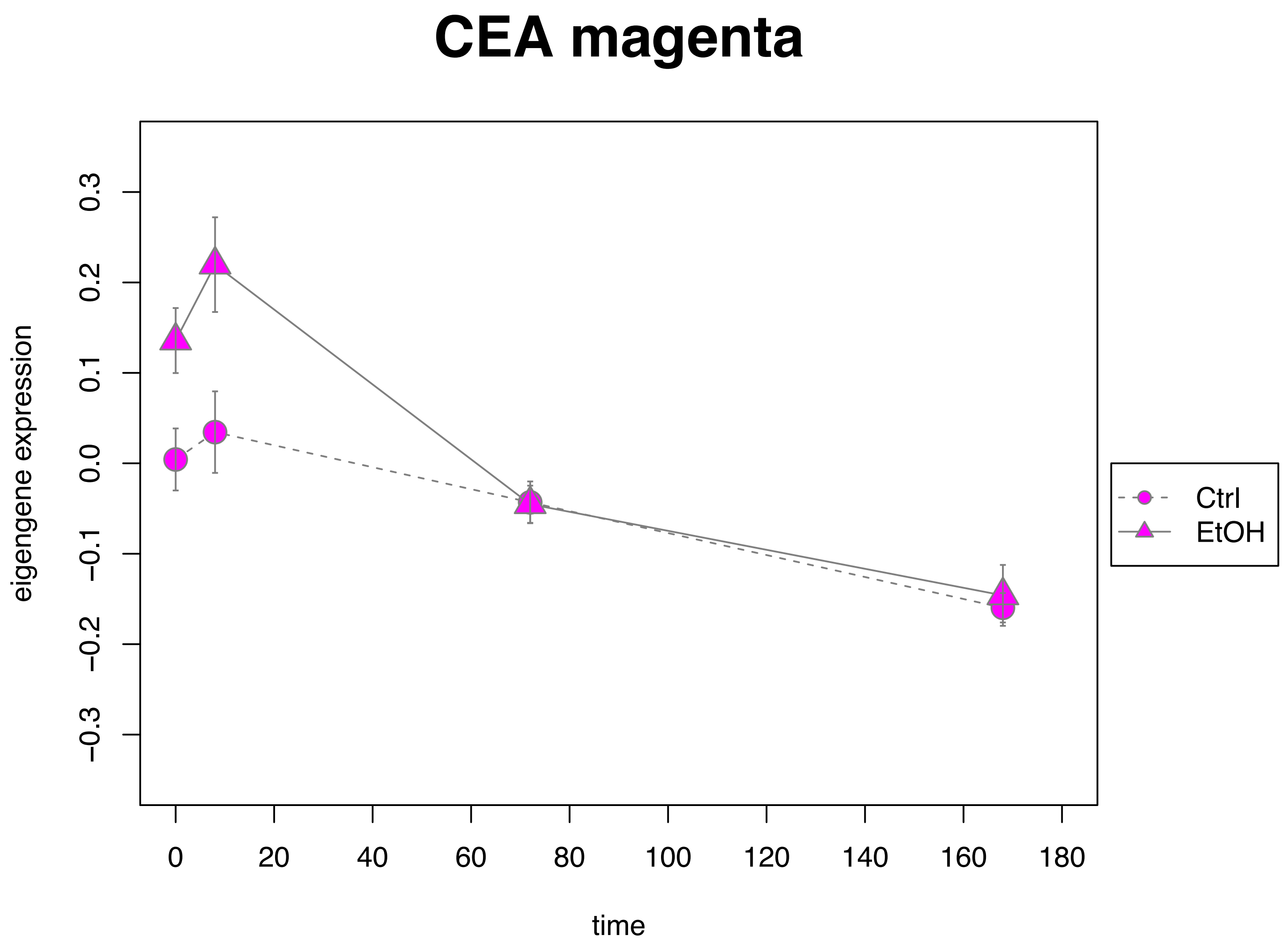
CEA magenta
eigengene expression
0.3
0.2
0.1
0.0
−0.1
−0.2
−0.3
0
20
40
60
80
100
120
140
160
180
time
Ctrl
EtOH

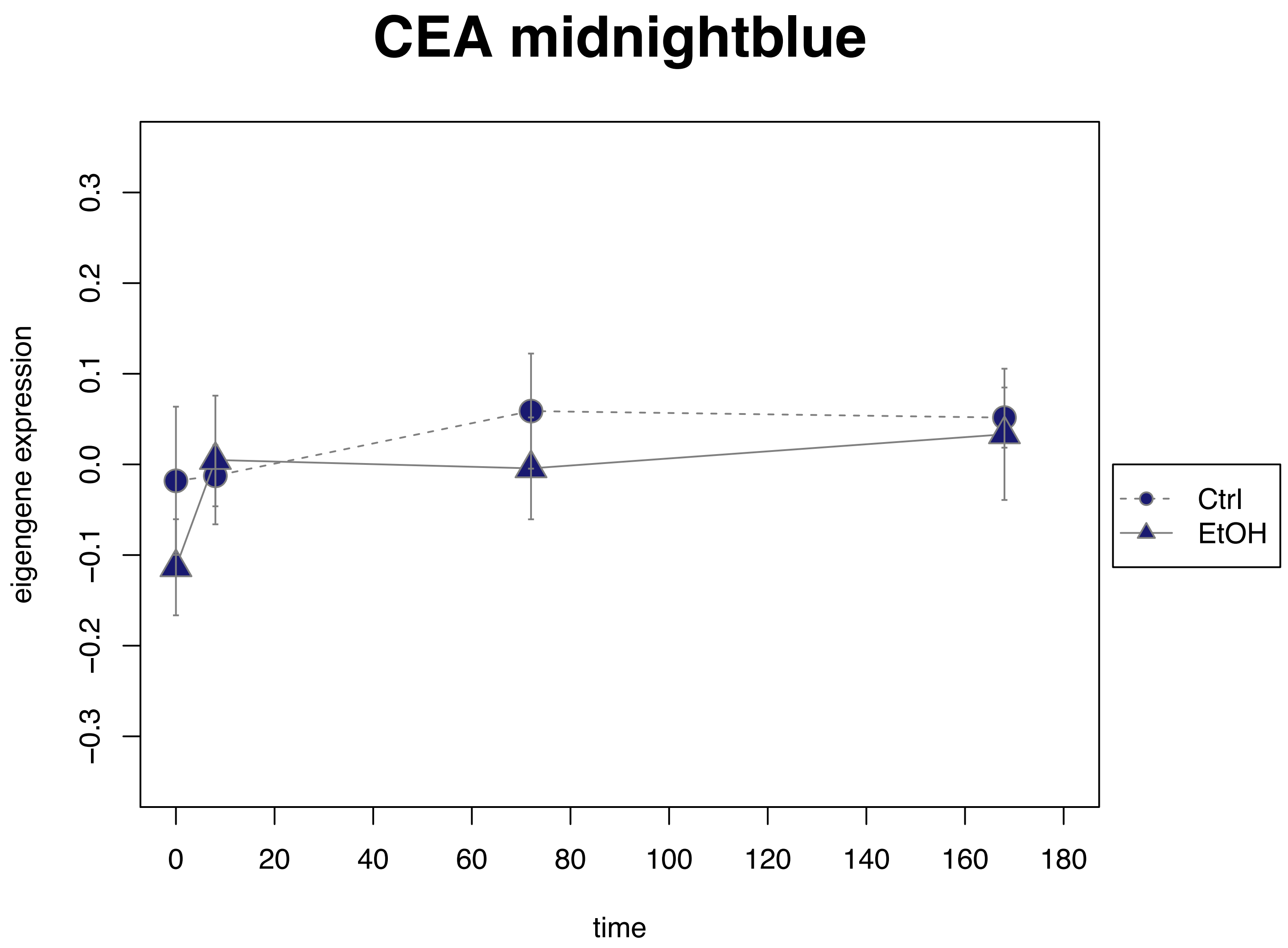
CEA midnightblue
eigengene expression
0.3
0.2
0.1
0.0
−0.1
−0.2
−0.3
0
20
40
60
80
100
120
140
160
180
time
Ctrl
EtOH

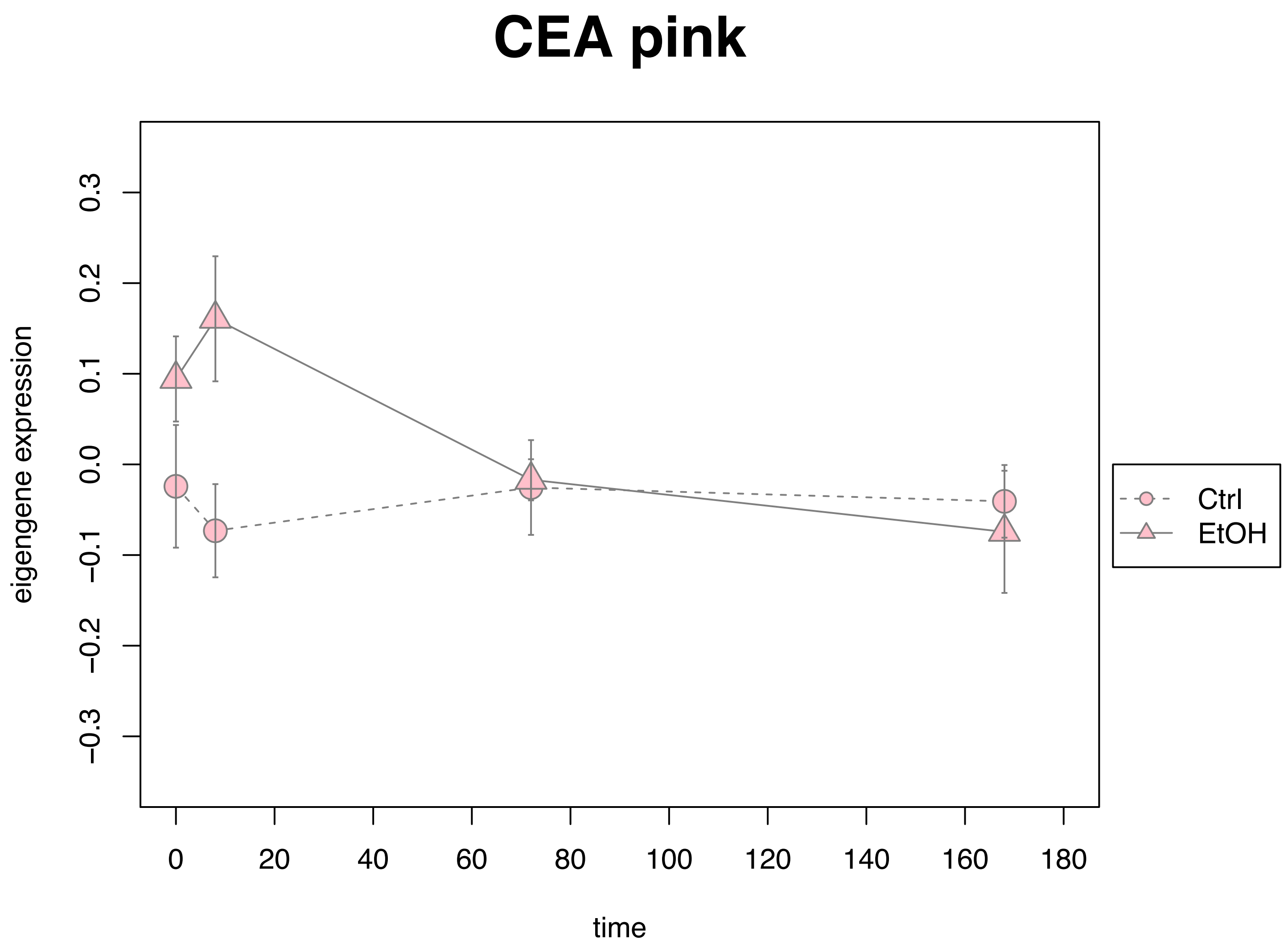
CEA pink
eigengene expression
0.3
0.2
0.1
0.0
−0.1
−0.2
−0.3
0
20
40
60
80
100
120
140
160
180
time
Ctrl
EtOH

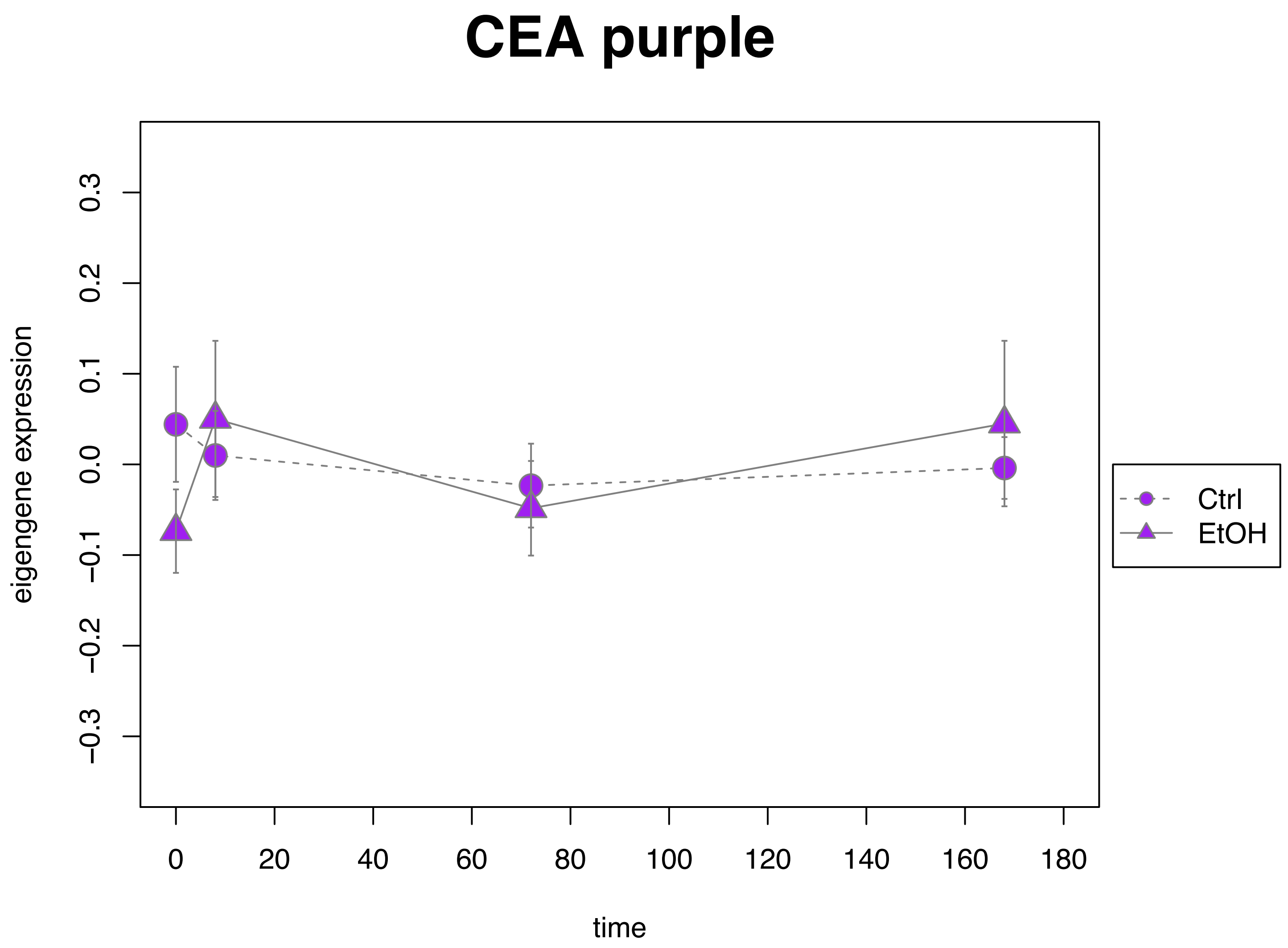
CEA purple
eigengene expression
time
Ctrl
EtOH
0.3
0.2
0.1
0.0
−0.1
−0.2
−0.3
0
20
40
60
80
100
120
140
160
180

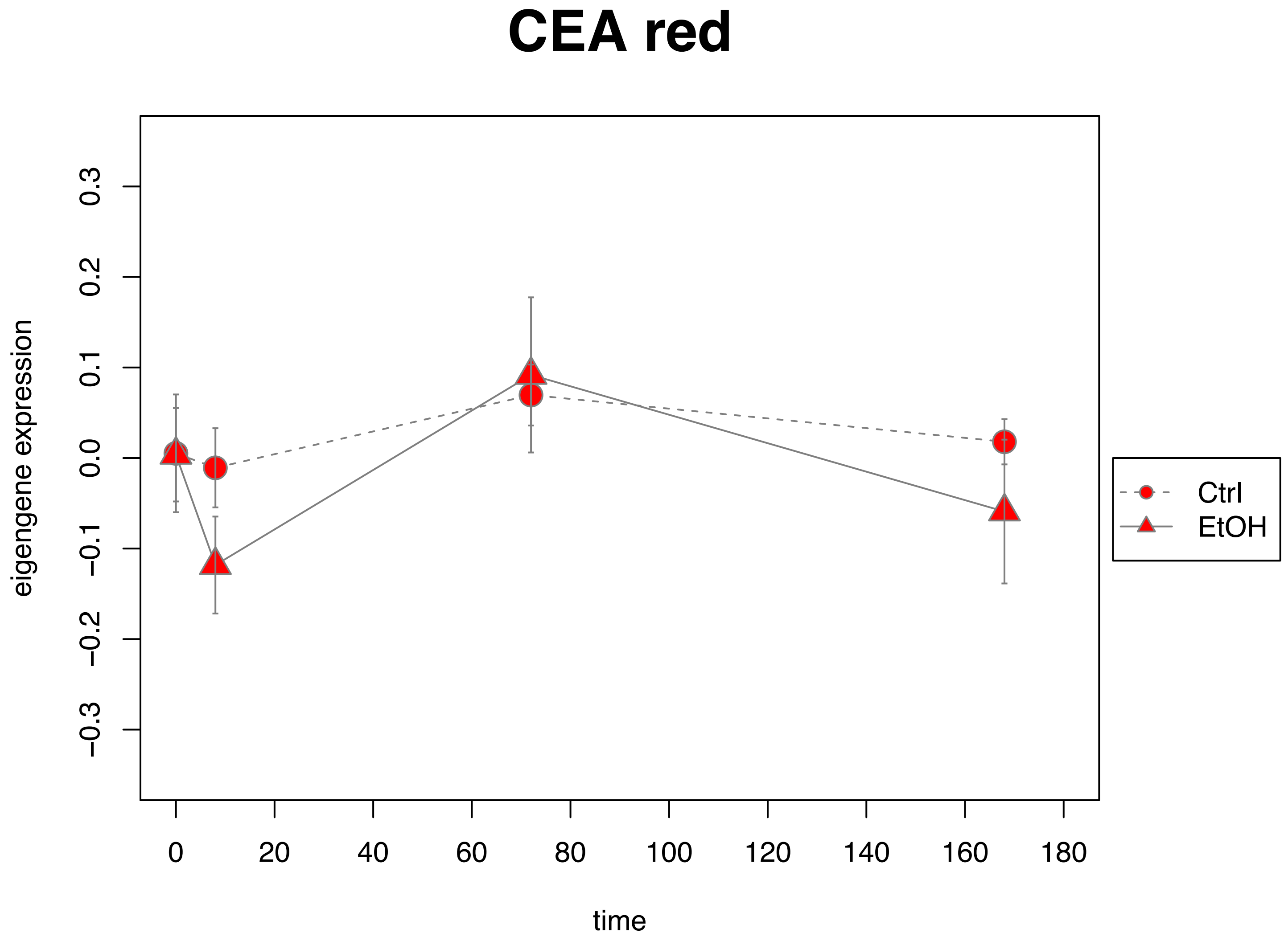
CEA red
eigengene expression
0.3
0.2
0.1
0.0
−0.1
−0.2
−0.3
0
20
40
60
80
100
120
140
160
180
time
Ctrl
EtOH

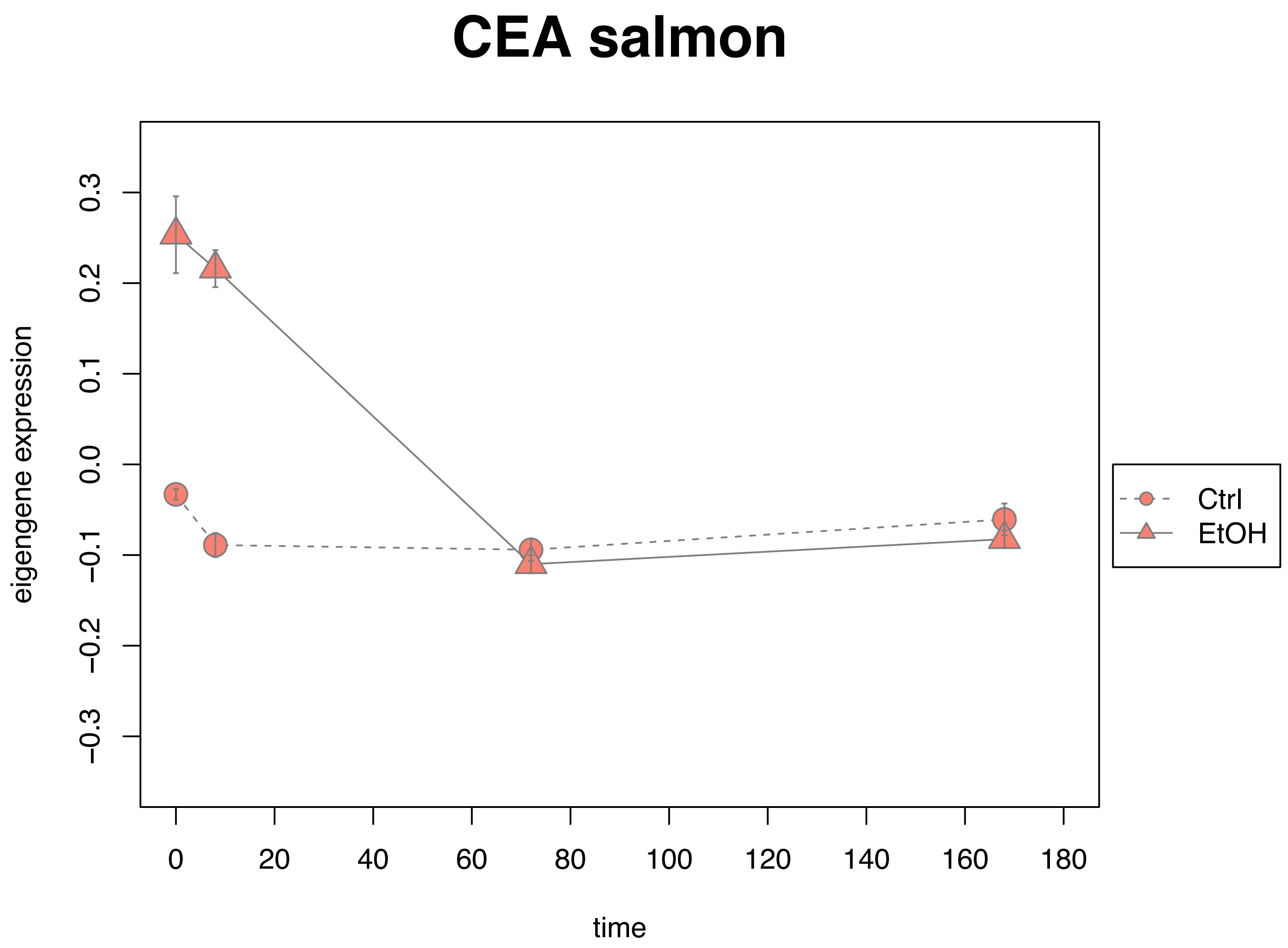

CEA salmon
eigengene expression
0.3
0.2
0.1
0.0
−0.1
−0.2
−0.3
0
20
40
60
80
100
120
140
160
180
time
Ctrl
EtOH

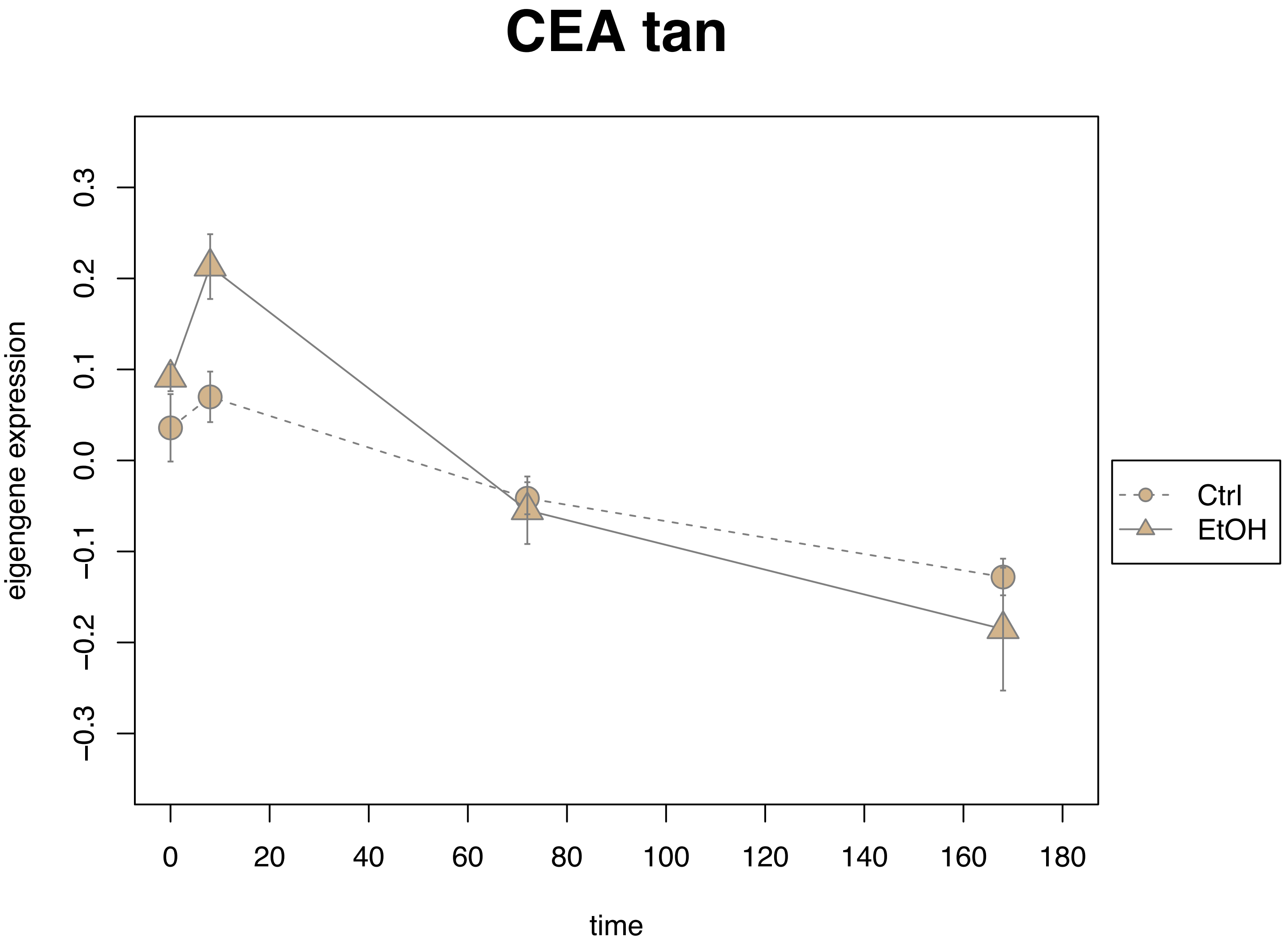
CEA tan
eigengene expression
0.3
0.2
0.1
0.0
−0.1
−0.2
−0.3
0
20
40
60
80
100
120
140
160
180
time
Ctrl
EtOH

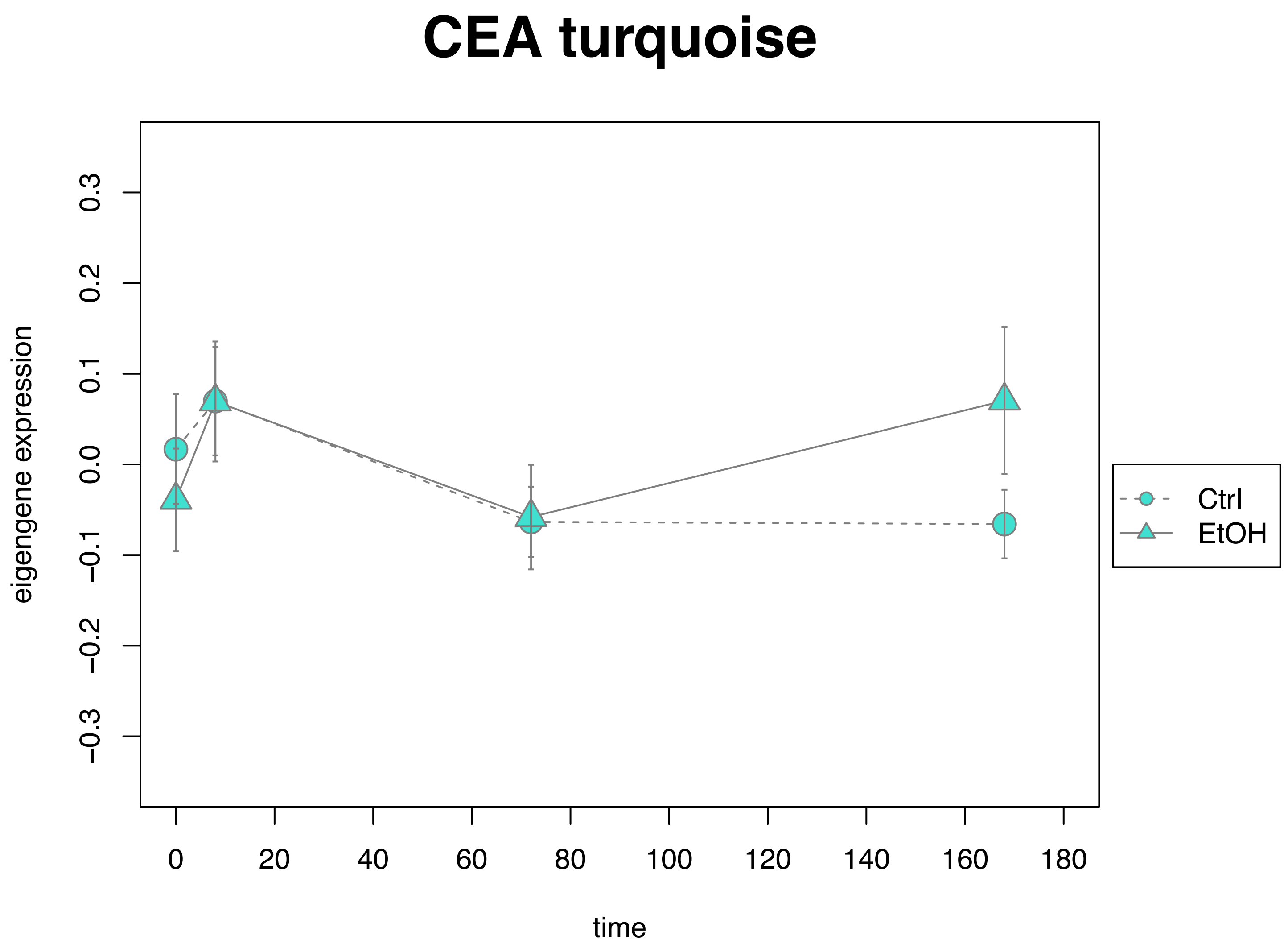

CEA turquoise
eigengene expression
0.3
0.2
0.1
0.0
−0.1
−0.2
−0.3
0
20
40
60
80
100
120
140
160
180
time
Ctrl
EtOH

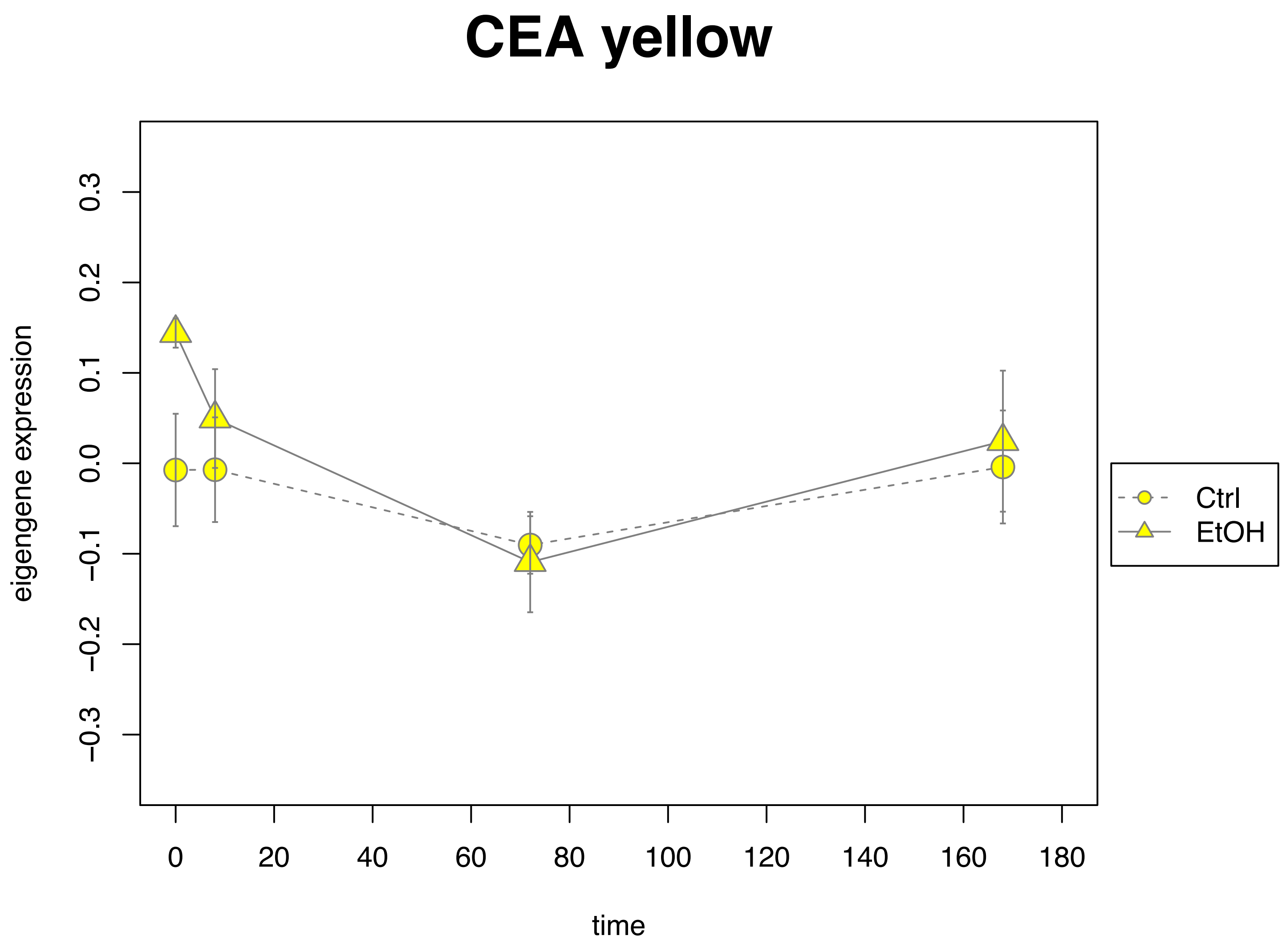

CEA yellow
eigengene expression
0.3
0.2
0.1
0.0
−0.1
−0.2
−0.3
0
20
40
60
80
100
120
140
160
180
time
Ctrl
EtOH
