# Supplementary figures and images for: Time-Course Analysis of Brain Regional Expression Network Responses to Chronic Intermittent Ethanol and Withdrawal: Implications for Mechanisms Underlying Excessive Ethanol Consumption
Source: PLoS One. 2016 Jan 5;11(1):e0146257. doi: 10.1371/journal.pone.0146257 (PMC4701666; doi:10.1371/journal.pone.0146257)

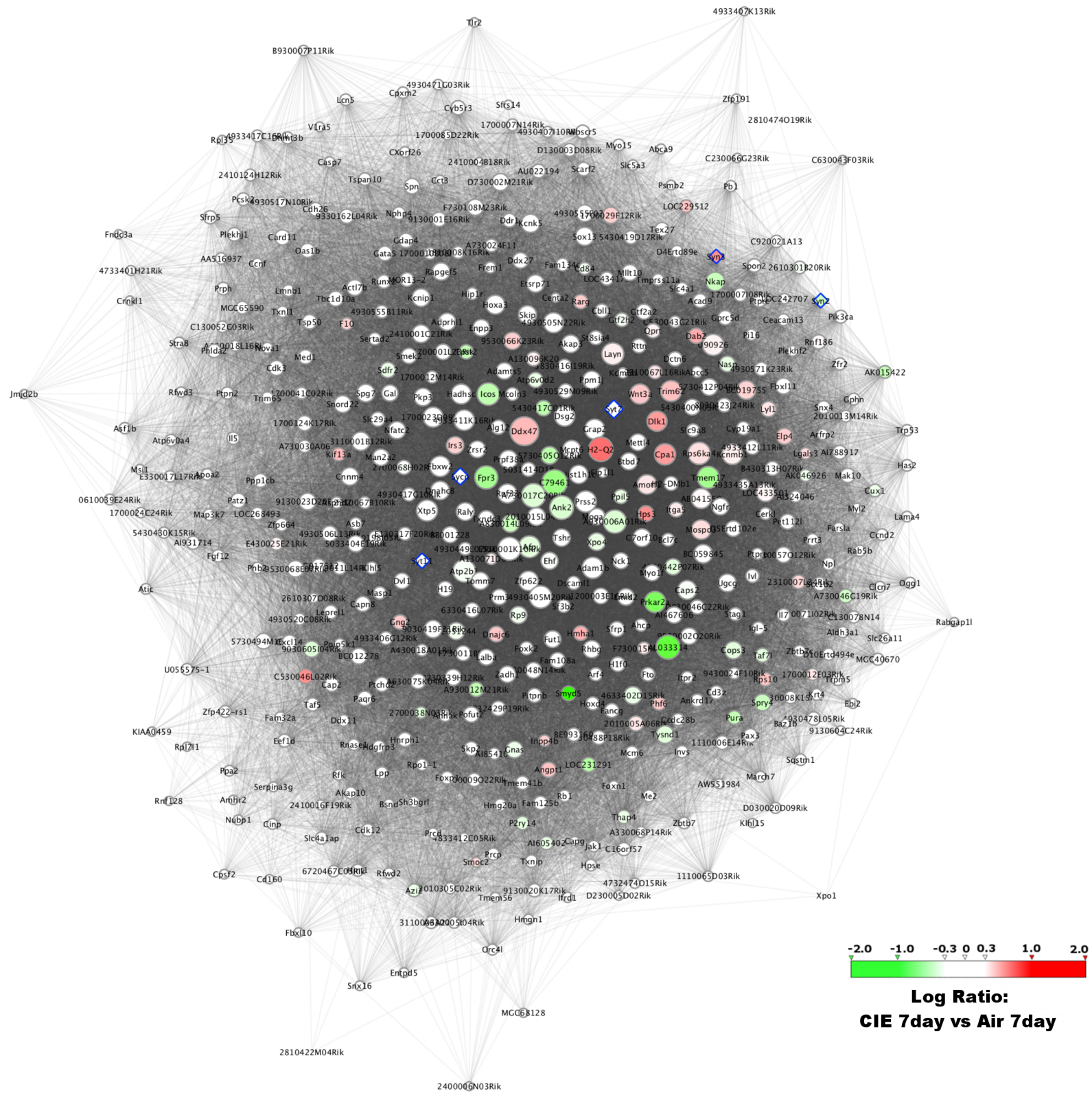

Supplement: S6 Fig — Edge transparency = Pearson correlation coefficient. Node size = within module determined by WGCNA. Node color = Log-ratio of gene expression at 5 days CIE vs. Ctrl. Genes involved in neurotransmitter release at the synapse highlighted. Network representation built using the Cytoscape resource (http://www.cytoscape.org). (PDF) [file pone.0146257.s006.pdf]

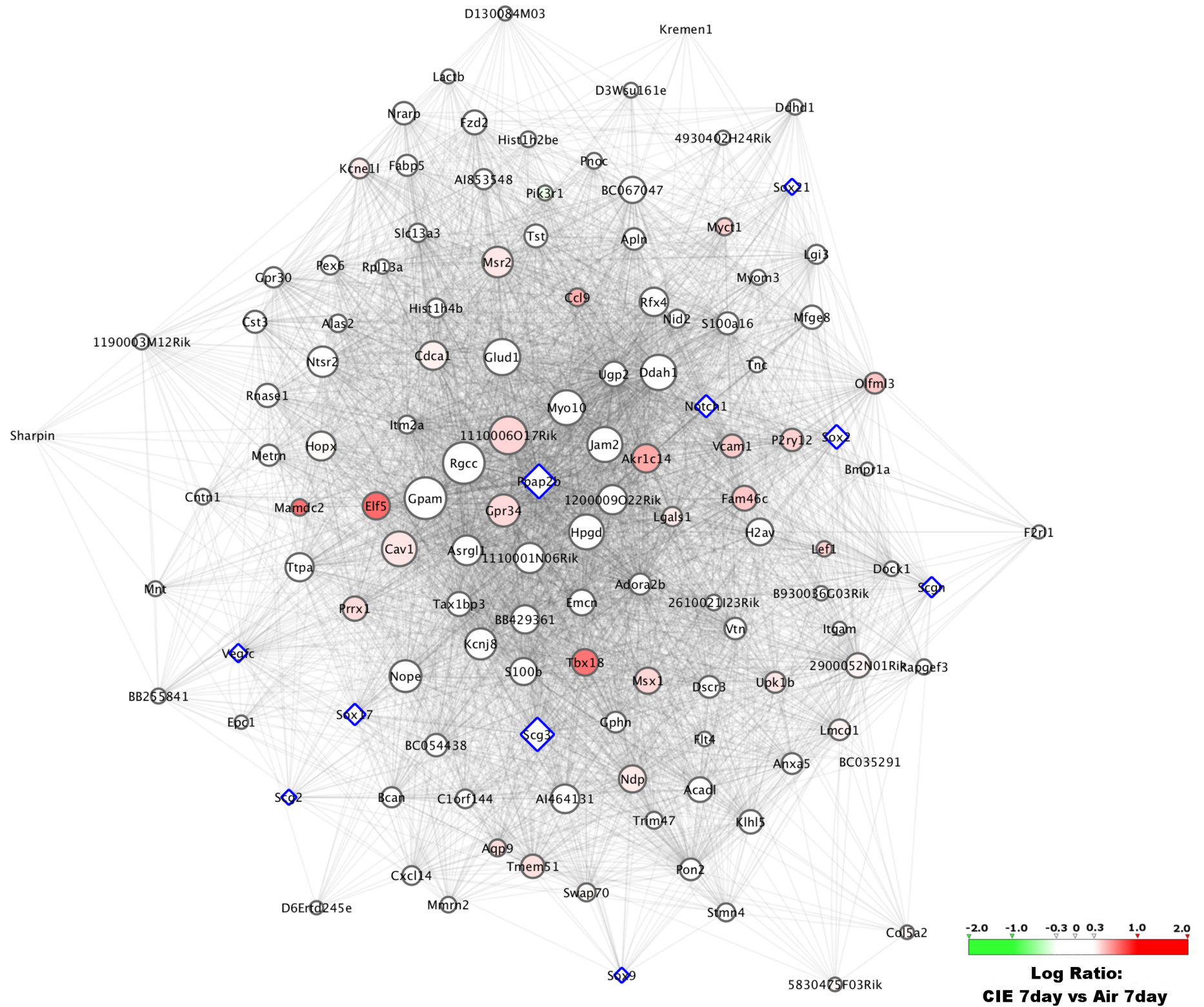

Supplement: S7 Fig — Edge transparency = Pearson correlation coefficient. Node size = within module determined by WGCNA. Node color = Log-ratio of gene expression at 5 days CIE vs. Ctrl. Highlighted genes indicate genes involved in neurodevelopment. Network representation built using the Cytoscape resource (http://www.cytoscape.org). (PDF) [file pone.0146257.s007.pdf]

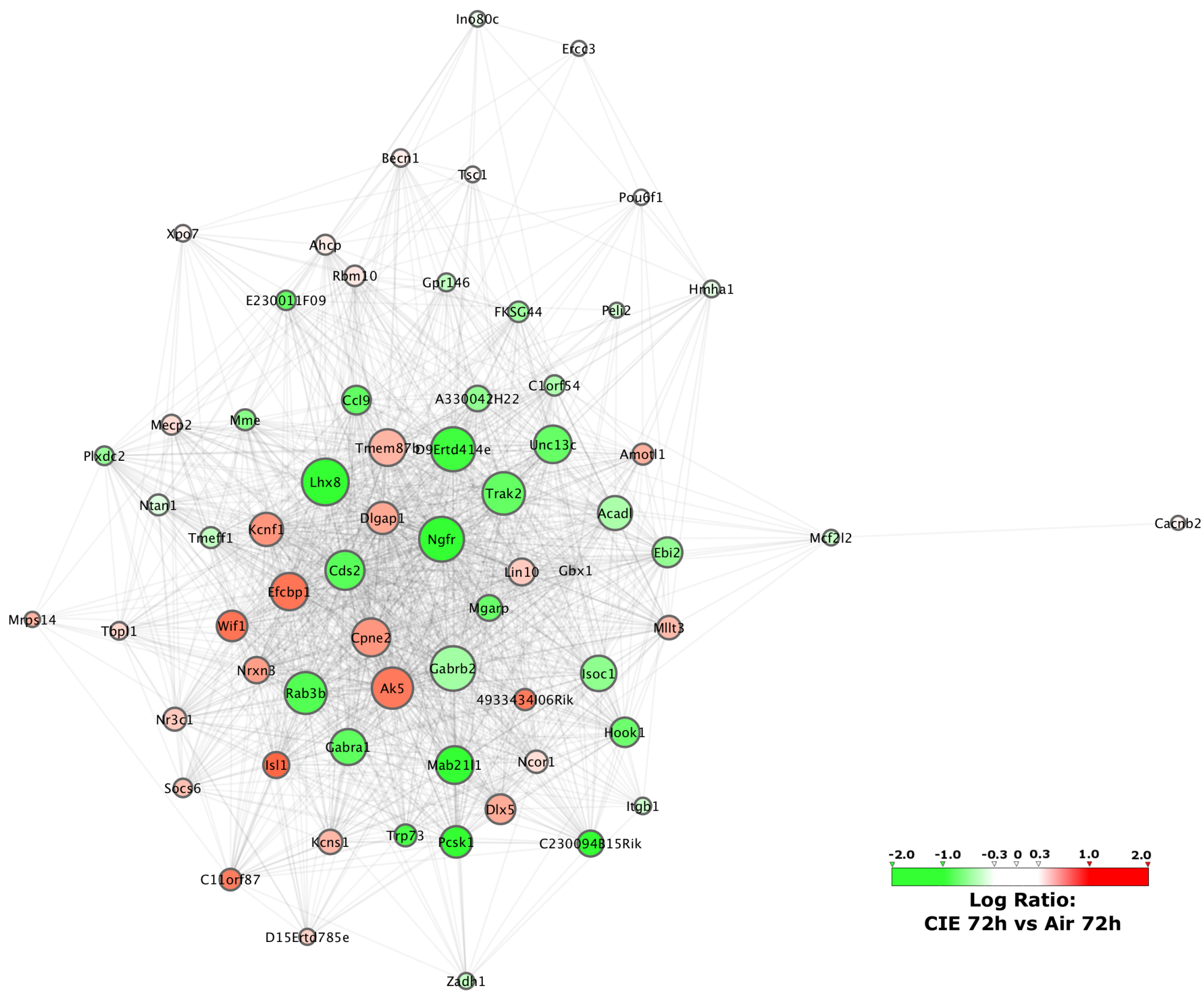

Supplement: S8 Fig — Edge transparency = Pearson correlation coefficient. Node size = within module determined by WGCNA. Node color = Log-ratio of gene expression at 72 hours CIE vs. Ctrl. Network representation built using the Cytoscape resource (http://www.cytoscape.org). (PDF) [file pone.0146257.s008.pdf]

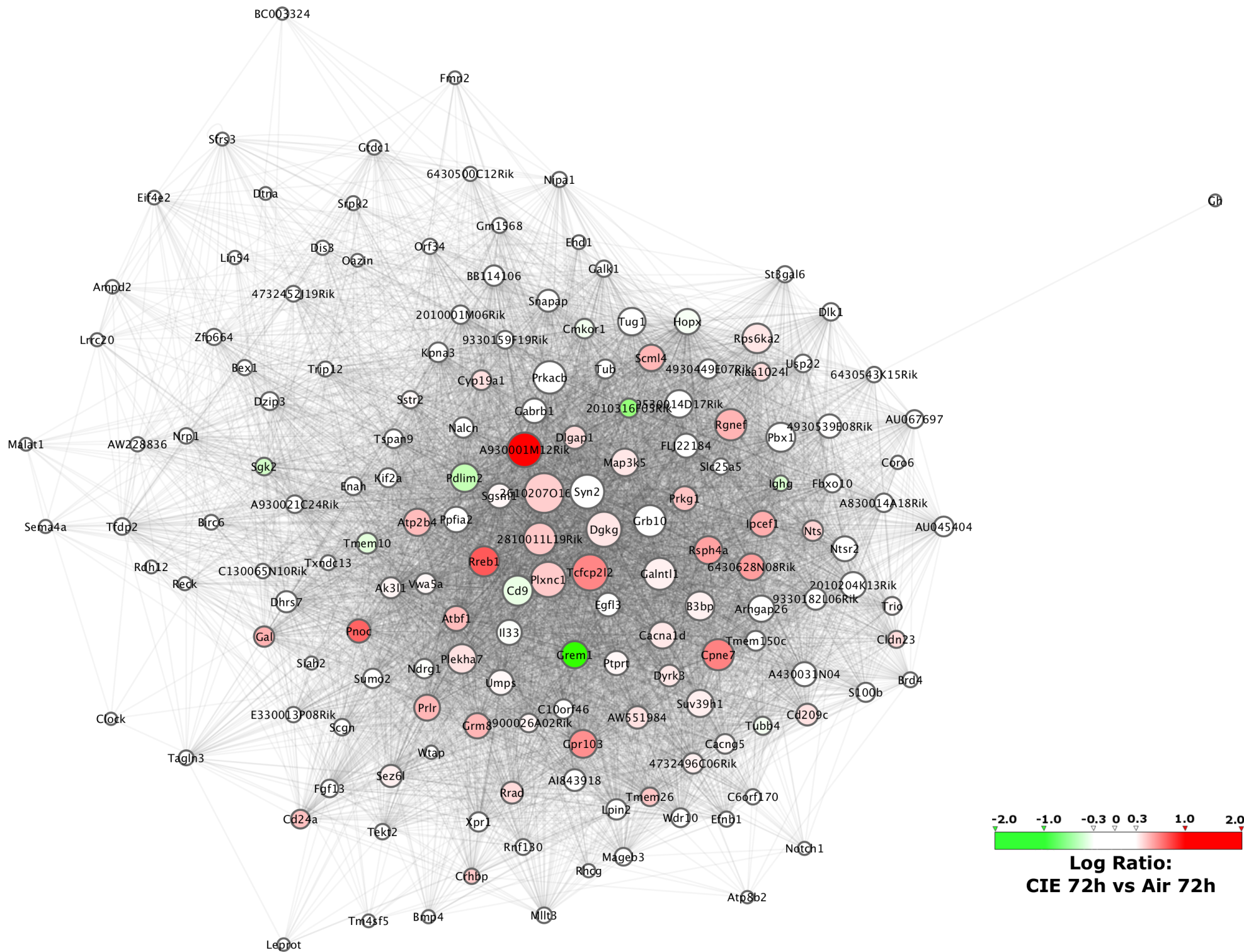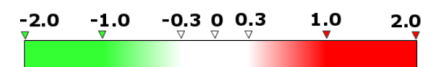

**Log Ratio:**  
**CIE 72h vs Air 72h**

Supplement: S9 Fig — Edge transparency = Pearson correlation coefficient. Node size = within module determined by WGCNA. Node color = Log-ratio of gene expression at 72 hours CIE vs. Ctrl. Network representation built using the Cytoscape resource (http://www.cytoscape.org). (PDF) [file pone.0146257.s009.pdf]

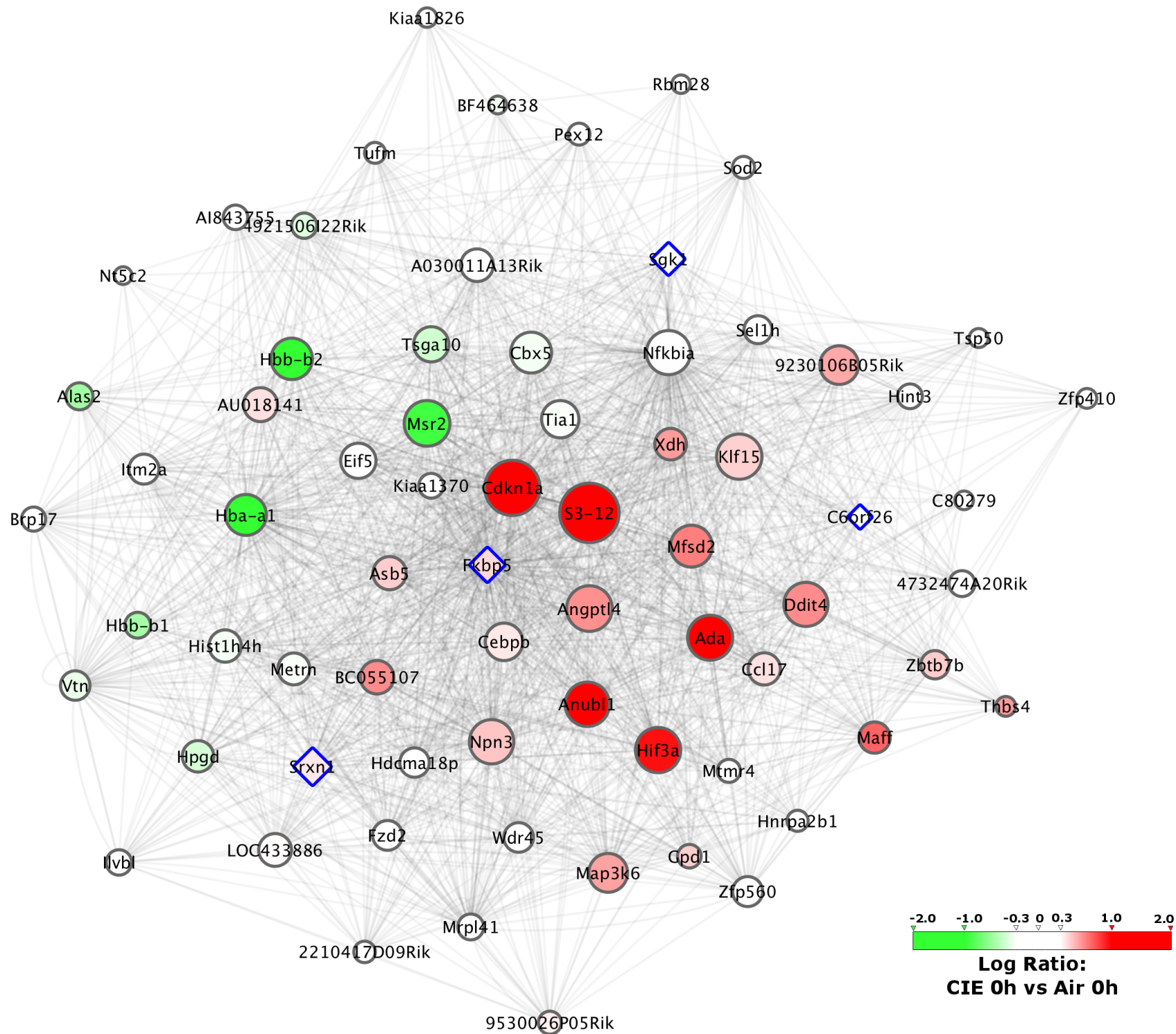

Supplement: S10 Fig — Edge transparency = Pearson correlation coefficient. Node size = within module determined by WGCNA. Node color = Log-ratio of gene expression at 0 hours CIE vs. Ctrl. Highlighted genes indicate genes shown to interact with NF-κB. Network representation built using the Cytoscape resource (http://www.cytoscape.org). (PDF) [file pone.0146257.s010.pdf]
