# Supplementary material for: Time-Course Analysis of Brain Regional Expression Network Responses to Chronic Intermittent Ethanol and Withdrawal: Implications for Mechanisms Underlying Excessive Ethanol Consumption
Source: PLoS One. 2016 Jan 5;11(1):e0146257. doi: 10.1371/journal.pone.0146257 (PMC4701666; doi:10.1371/journal.pone.0146257)
Supplement: S6 Table — Columns display the number of genes overlapping for the indicated timepoint comparisons within brain regions. (DOCX) [file pone.0146257.s016.docx]

**Suppl. Table S2: Time point comparisons of CIE-regulated genes within brain regions**

Number of probesets significantly regulated (LIMMA CIE vs. Ctrl FDR ≤ 0.01) within specified brain regions at each combination of time-points indicated in comparison column.

| **Time Comparisons** | **PFC** | **NAC** | **HPC** | **BNST** | **CEA** |
| --- | --- | --- | --- | --- | --- |
| **0hr vs. 8hr** | 197 | 11 | 139 | 116 | 23 |
| **0hr vs. 72hr** | 34 | 0 | 0 | 6 | 0 |
| **0hr vs. 7day** | 131 | 0 | 104 | 0 | 0 |
| **8hr vs. 72hr** | 56 | 0 | 0 | 3 | 0 |
| **8hr vs. 7day** | 16 | 0 | 92 | 0 | 0 |
| **72hr vs. 7day** | 71 | 0 | 1 | 0 | 0 |
